# Genome-Wide Identification and Characterization of *Isoflavone Synthase (IFS)* Gene Family, and Analysis of GgARF4-GgIFS9 Regulatory Module in *Glycyrrhiza glabra*

**DOI:** 10.3390/ijms262110435

**Published:** 2025-10-27

**Authors:** Qing Xu, Xiangxiang Hu, Shiyan Cui, Jianguo Gao, Lijie Zeng, Ziqi Li, Sheng Kuang, Xifeng Chen, Quanliang Xie, Zihan Li, Hongbin Li, Fei Wang, Shandang Shi, Shuangquan Xie

**Affiliations:** 1 Key Laboratory of Xinjiang Phytomedicine Resource and Utilization of Ministry of Education, Key Laboratory of Oasis Town and Mountain-Basin System Ecology of Bingtuan, College of Life Sciences, Shihezi University, Shihezi 832000, China; xq19901526963@163.com (Q.X.);; 2 Department of Civil, Environmental and Construction Engineering, College of Engineering and Computer Science, University of Central Florida, Orlando, FL 32816, USA

**Keywords:** *Glycyrrhiza glabra*, isoflavone synthase, gene family, isoflavonoid, auxin response factor

## Abstract

Isoflavone synthase (IFS) is the key enzyme in isoflavonoid biosynthesis and has been functionally characterized in numerous plant species. *Glycyrrhiza* species, valued for their medicinal properties, accumulate flavonoids with significant physiological activities. Among these, isoflavones play crucial roles in plant growth, development and stress responses. However, the *IFS* gene family in *Glycyrrhiza* remains poorly understood. In this study, we identified 10, 9 and 9 *IFS* genes in *G. uralensis*, *G. inflata* and *G. glabra*, respectively. Phylogenetic analysis classified these genes into four distinct clades (Clade A–D). Further characterization included chromosomal localization, gene structure, conserved motifs, *cis*-acting elements and synteny analysis. Using yeast one-hybrid (Y1H) screening, dual-luciferase assays and an electrophoretic mobility shift assay (EMSA), these results revealed that auxin response factor 4 (GgARF4) directly binds to the isoflavone synthase 9 *(GgIFS9)* promoter and activates its expression. Following indole-3-acetic acid (IAA) treatment, RNA-seq revealed that in the differentially expressed genes (DEGs), the genes involved in isoflavonoid and flavonoid biosynthesis pathways were significantly enriched. The result of quantitative reverse transcription polymerase chain reaction (qRT-PCR) revealed that *GgIFS9* was strongly induced by IAA. β-Glucuronidase (GUS) assays confirmed that IAA activates the expression of the *GgIFS9* promoter in *Nicotiana tabacum*. Our findings reveal that, through GgARF4 and its downstream-activated gene *GgIFS9,* IAA may promote flavonoid synthesis in *G. glabra*. This study provides novel insights into the auxin-mediated regulation of secondary metabolism in *Glycyrrhiza* species.

## 1. Introduction

*Glycyrrhiza glabra* L., a member of the Leguminosae family, represents one of the most valued medicinal plants in traditional Chinese medicine (TCM) [1]. The therapeutic potential of this species primarily resides in its dried roots and rhizomes, which contain diverse bioactive compounds responsible for its wide pharmacological applications [2]. Among these bioactive constituents, flavonoids and triterpenoid saponins are particularly noteworthy, with isoflavones emerging as a critical subclass of flavonoids [3]. These specialized metabolites, predominantly found in leguminous plants, play pivotal roles in plant physiology, including growth regulation, developmental processes and stress responses [4,5].

Extensive research has elucidated the multifaceted functions of isoflavones in plant systems. These compounds not only modulate root architecture but also significantly enhance the development of lateral buds, leaves and reproductive structures [6]. Through their influence on endogenous hormone homeostasis, isoflavones promote lateral bud outgrowth and branch elongation, thereby establishing robust branching patterns and optimizing photosynthetic efficiency [7]. Moreover, these metabolites precisely regulate cellular processes during leaf morphogenesis, including cell division and expansion, ultimately contributing to enhanced leaf development and photosynthetic performance [8]. The biosynthesis of isoflavones centers around isoflavone synthase (IFS), a cytochrome P450 monooxygenase that serves as the key enzyme in the phenylpropanoid pathway [9]. IFS catalyzes the conversion of flavanones into 2-hydroxyisoflavones, which subsequently undergo dehydration mediated by isoflavone dehydratase (HID) to yield 4′-hydroxyisoflavones [10]. Alternatively, 2-hydroxyisoflavones can be modified through methylation by 2-hydroxyisoflavone 4′-O-methyltransferase (HI4′OMT), producing various derivatives including biochanin A and chickpea isoflavone A [10]. Notably, the metabolic network branches at naringenin, which serves as a common precursor for multiple pathways, including those leading to flavonols and anthocyanins through the actions of flavanone 3-hydroxylase (F3H) and flavonol synthase (FLS) [11]. Additional transformations of liquiritigenin and naringenin yield various defensive compounds such as flavones and quercetin, significantly contributing to plant adaptation mechanisms [12].

The phytohormone auxin stands as a fundamental regulator of plant growth and development [13,14]. The auxin signaling pathway, mediated by auxin response factors (ARFs), plays a particularly significant role in root system development and the biosynthesis of secondary metabolites, including isoflavones. Integrated transcriptomic and metabolomic analysis in *Pueraria lobata* have demonstrated a positive correlation between auxin/ARF expression, root expansion, and the accumulation of flavonoids and isoflavones in root tubers [15]. Similar investigations in peanut have revealed the involvement of auxin in nodulation processes and flavonoid accumulation [16]. Furthermore, exogenous IAA application in *Cicer arietinum* has been shown to elevate levels of antioxidant enzymes (catalase and glutathione) along with secondary metabolites including flavonoids and phenols [17]. However, there are few reports of *IFS* gene family and regulation mechanism in *G. glabra*.

Therefore, in the present study, we conducted an analysis of the *IFS* gene family across three *Glycyrrhiza* species (*G. uralensis*, *G. inflata* and *G. glabra*). Molecular characterization revealed that GgARF4 directly bound to and transcriptionally activated the *GgIFS9* promoter. RNA-seq analysis highlighted significant enrichment of phenylpropanoid biosynthesis pathways among DEGs. The expression patterns of *GgIFS* genes indicated that *GgIFS9* was induced by IAA in *G. glabra*. Functional validation demonstrated that IAA treatment enhanced *GgIFS9* promoter activity. These findings provide crucial insights into the molecular mechanisms governing isoflavone biosynthesis and establish a foundation for future efforts to develop *G. glabra* cultivars with enhanced isoflavone content through targeted genetic approaches.

## 2. Results

### 2.1. Identification and Classification of IFS Gene Family Members

A total of 9, 9, 10 members of *GgIFS*, *GiIFS*, *GuIFS* were obtained from the genome of *G. glabra, G. inflata* and *G. uralensis*. The ID of IFS members was named according to the location of chromosomes. The coding sequences (CDSs) and protein sequences are listed in Table S1. Subsequently, we predicted the physicochemical properties of IFS proteins in three *Glycyrrhiza* species, including the number of amino acids (aa), molecular weight (MV), theoretical pI, instability index, aliphatic index and grand average of hydropathicity (GRAVY) (Table S2). The molecular weight ranged from 38.817 to 60.610 kDa and the pI spanmed from 6.07 to 8.05. The GRAVY values were negative, indicating that they were hydrophilic. Most of the instability indexes of the IFS proteins were above 40, showing that they were unstable. The prediction of subcellular localization revealed that most IFS proteins were located in the cytoplasm, while others were located in chloroplast, membrane, nucleus and endosome (Table S2).

### 2.2. Phylogenetic Relations of the IFS Gene Family

**Figure 1:**
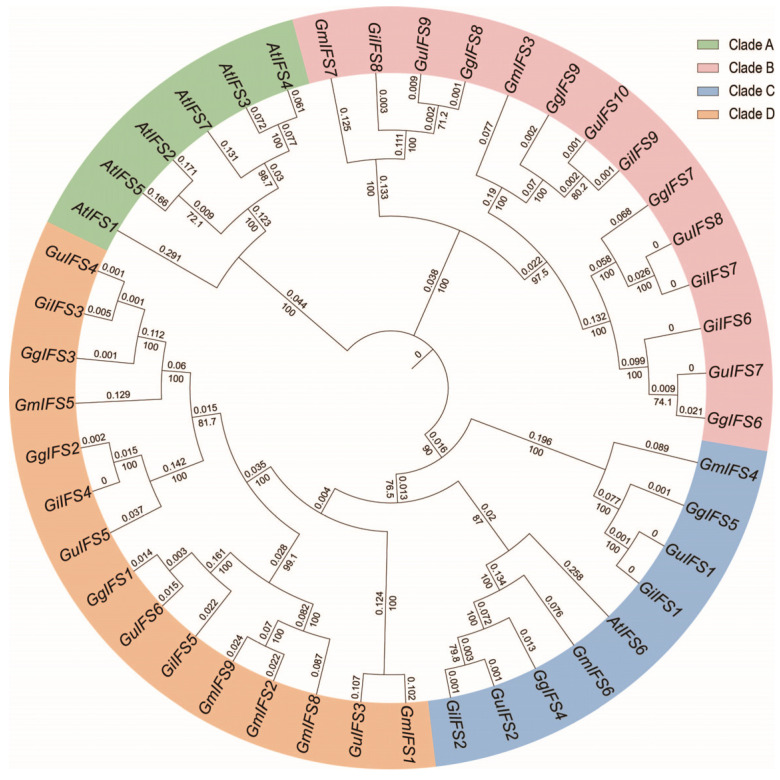
Phylogenetic analysis of the *IFS* gene family members in *G. uralensis*, *G. inflata*, *G. glabra*, *G. max* and *A. thaliana*. Gu, Gi, Gg, Gm and At represent *G. uralensis*, *G. inflata*, *G*. *glabra*, *G. max* and *A. thaliana*, respectively. The phylogenetic tree is divided into four branches: Clade A, Clade B, Clade C and Clade D. The namex of *IFS* genes on the branches represent different *IFS* gene members of each species, and the phylogenetic tree shows the degree of genetic relationship among these gene members.

In order to further elucidate the evolutionary relationships in the *IFS* gene family, we constructed a phylogenetic tree using the protein sequences of the *IFS* gene family. The 44 members were divided into four branches (Clade A–D) (Figure 1). Clade A only contained six *AtIFS* genes, comprising the smallest size group. The Clade B contained 15 gene members, comprising the largest group. Clade C and Clade D had 9 and 14 members, respectively.

### 2.3. Chromosome Location of IFS Genes in Three Glycyrrhiza Species

**Figure 2:**
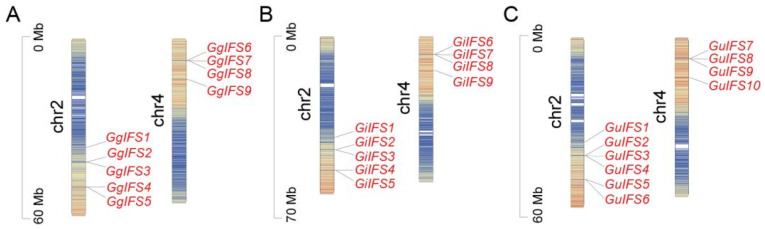
Distribution map of *IFS* family genes on the chromosomes of three *Glycyrrhiza* species. (**A**–**C**) corresponds to *G. glabra*, *G. inflata* and *G. uralensis*, respectively. Chr2 and chr4 represents different chromosomes and different colors indicate different characteristics of the chromosomes. The *IFS* genes are marked in red and the arrows indicate their positions on the chromosomes. The scale on the left side of the chromosome represents the chromosome length (in Mb).

To investigate the distribution of *IFS* genes on the chromosomes, 28 *IFS* genes were mapped (Figure 2). The 28 *IFS* genes were unevenly distributed on chromosome 2 and 4 in three *Glycyrrhiza* species. For example, five members were located on chromosome 2 and four members on chromosome 4 in *G. glabra* (Figure 2A).

### 2.4. Analysis of Collinearity Relationships Among IFS Gene Families in Three Glycyrrhiza Species

**Figure 3:**
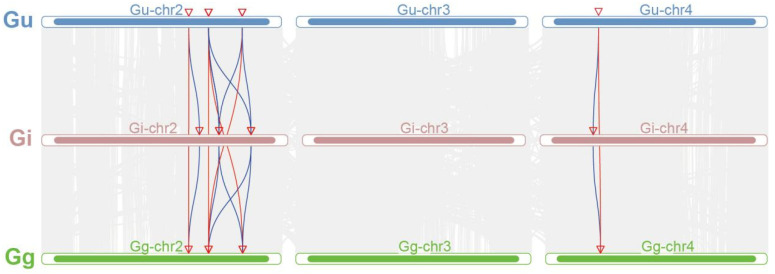
Collinearity relationship diagram of *IFS* family genes among chromosomes of *G. uralensis*, *G. inflata* and *G. glabra*. Gu, Gi and Gg represent *G. uralensis*, *G. inflata* and *G. glabra*, respectively. Gg-Chr2–Gg-Chr4, Gi-Chr2–Gi-Chr4, and Gu-Chr2–Gu-Chr4 represent different chromosomes of the corresponding *Glycyrrhiza* species. The arrows represent gene location.

To explore the gene duplication events of *IFS* genes, the collinearity relationship was analyzed (Figure 3). In the figure, different colored lines connect the chromosomes of different species, with each line representing a collinear region between chromosomes, indicating the conserved order and orientation of genes in these regions. We found four collinear gene pairs among *G. uralensis*, *G. inflata* and *G. glabra*. The *IFS* genes between two *Glycyrrhiza* species all have five gene pairs’ collinearity relationships, and one *IFS* gene was homologous to two *IFS* genes in three *Glycyrrhiza* species (Table S3). In *G. uralensis*, *G. inflata* and *G. glabra*, there was one segmental duplicated gene pair (Figure S1). These results suggested that these *IFS* genes may play an important role in the evolution of the *IFS* gene family in licorice.

### 2.5. Analysis of Promoter Cis-Acting Elements, Conserved Motifs and Gene Structures of IFS Gene Family

**Figure 4:**
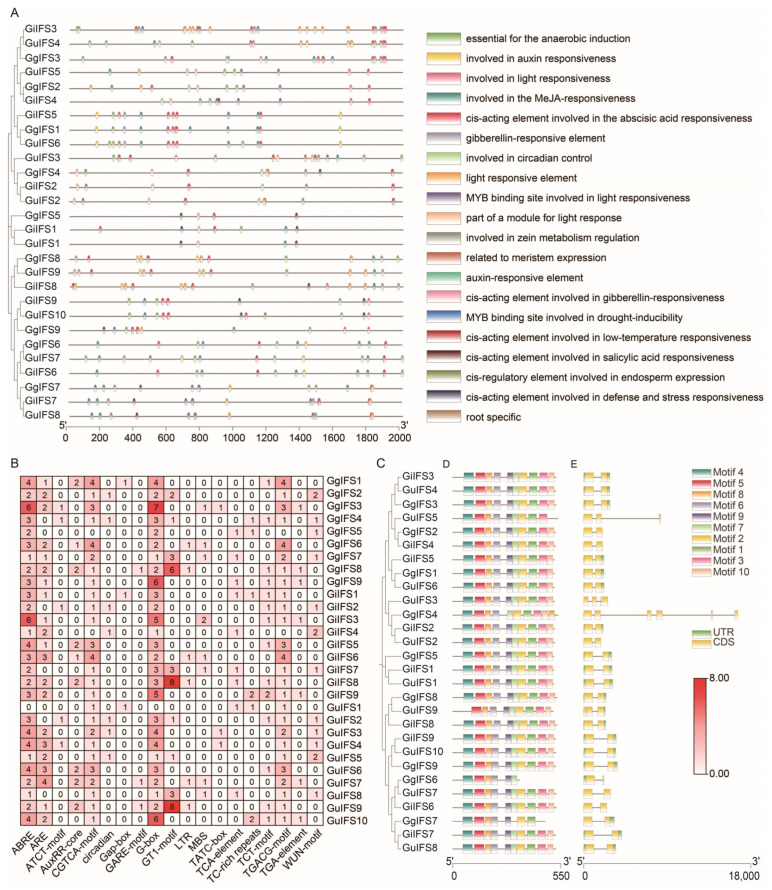
Analysis of promoter *cis*-acting elements, conserved motifs and gene structure of *IFS* family genes in *G. uralensis*, *G. inflata* and *G. glabra*. (**A**) Analysis of the distribution of *cis*-acting elements in the promoter region of *IFS* genes. Different-colored ellipses represent different types of elements. The horizontal axis represents the base positions from the 5′ to the 3′ end of the promoter. (**B**) The heatmap of the number of different *cis*-acting elements in each *IFS* gene, which is reflected by the depth of color. The specific values are marked within the squares. (**C**) Phylogenetic tree-based evolutionary relationship analysis of *IFS* genes from different species. The branches reflect the degree of genetic relatedness. (**D**) Conserved motif analysis of the *IFS* genes from different species. Squares of different colors represent different motifs, visually presenting the types and positions of motifs contained in the genes. (**E**) Gene structure analysis of the *IFS* genes. The black lines and green bars represent the introns and exons, respectively.

We analyzed the *IFS* gene family from different aspects, including *cis*-acting elements, conserved motifs and exon–intron structure (Figure 4). The evolutionary relationship of closely related genes exhibited similar *cis*-acting element distribution patterns (Figure 4A). The 19 *cis*-acting elements were identified, and they can be divided into three categories: hormone-responsive (auxin, ABA, MeJA, salicylic acid and gibberellin) elements, stress-responsive elements and development-responsive elements. The stress-responsive elements, such as the MYB binding site (MBS) and low-temperature reactivity (LTR), also existed in *IFS* genes (Figure 4B). The heatmap of *cis*-acting elements shows that many elements were hormone-responsive, such as ABA (ABRE), auxin (AuRR-core and TGA-element), gibberellin (GARE-motif, TATC-box and P-box), MeJA (CGTCA-motif and TGACG-motif) and salicylic acid (TCA-element) (Figure 4B). A total of ten motifs were identified, namely motif 1–10 (Figure 4D). The number of motifs ranged from 5 to 10; most of IFS proteins had 10 motifs. The results of gene structure analysis indicated that genes closely related in terms of their evolutionary relationship exhibited similar gene structures (Figure 4C,E).

### 2.6. The GgARF4 Protein Positively Regulates Isoflavone Synthase GgIFS9 in G. glabra

**Figure 5:**
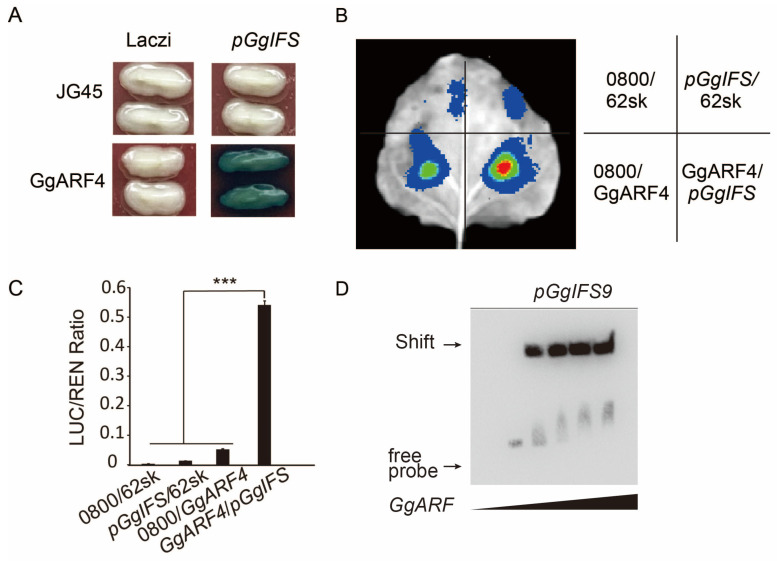
The GgARF4 protein binds directly to the promoter of the *GgIFS9* gene and activates its expression. (**A**) Interaction between *GgIFS9* and GgARF4 in the yeast one-hybrid (Y1H) assay. The *placzi* and *JG45* empty vectors were used as negative controls. (**B**) Dual-luciferase assay conducted to determine the interaction between *GgIFS9* and GgARF4 in tobacco in vivo. The *pGreenⅡ 62-SK* and *pGreenⅡ 0800-LUC* empty vectors were used as controls. (**C**) Quantification of *LUC* expression. The *pGreenⅡ 62-SK* and *pGreenⅡ 0800-LUC* empty vectors (0800/62sk), the *pGreenⅡ 62-SK* and *pGreenⅡ0800-LUC-GgIFS* (*pGgIFS*/62sk) empty vectors, the *pGreenⅡ 62-SK-GgARF4* and *pGreenⅡ 0800-LUC* empty vectors (0800/GgARF4) were used as controls. The data have been normalized according to the LUC/REN standard. Statistical tests were conducted via one-way analysis of variance (ANOVA, *** *p*  <  0.001). (**D**) EMSA of GgARF4 and *GgIFS9*. The EMSA was performed as described in the Methods section. Cold and mutant competitor controls were not included in this experiment. Error bars represent the SD (*n* = 3). Statistical tests were conducted via one-way analysis of variance (ANOVA, *** *p*  <  0.001).

In order to identify the transcription factors that interact with the *GgIFS9* gene, we conducted yeast library screening. The candidate TFs are listed in Table S4. To clarify whether the candidate TFs can directly regulate the transcription of the *GgIFS9* gene, we conducted a Y1H assay (Figure S2). The results showed that only GgARF4 could bind to the promoter of *GgIFS9* (Figure 5A). The result of a transient in vivo expression assay in *N. tabacum* showed that, compared with that of the control, the LUC reporter activity of GgARF4 with the co-transfected *GgIFS9* promoter was significantly enhanced, with about a six-fold change (Figure 5B,C). The results of the EMSA revealed that GgARF4 had binding affinity for the promoter of *GgIFS9* (Figure 5D). Based on the results, this study confirmed that GgARF4 can directly bind to the promoter sequence of the *GgIFS9* gene and activate its expression.

### 2.7. Transcriptome Sequencing Analysis and qRT-PCR Analysis of GgIFS Genes After IAA Treatment

**Figure 6:**
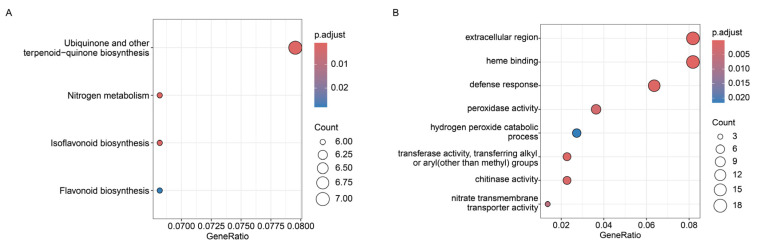
Transcriptomic analysis between the wild-type and IAA-treated *G. glabra*. (**A**) KEGG annotation of DEGs. (**B**) GO annotation of DEGs. Three independent biological replicates were conducted in the experiment.

To characterize the molecular mechanism, we performed RNA-seq on the wild-type and IAA-treated plant of *G. glabra*. In DEGs, up-regulated genes and down-regulated genes were identified (Figure S3). The KEGG enrichment analysis showed that the biosynthesis of nitrogen metabolism, isoflavonoid biosynthesis and flavonoid biosynthesis, ubiquinone and other terpenoid–quinones were enriched in the DEGs (Figure 6A). The results of Gene Ontology (GO) revealed that the extracellular region, heme binding, defense response, peroxidase activity, the hydrogen peroxide catabolic process, transferase activity, transferring alkyl or aryl (other than methyl) groups, chitinase activity and nitrate transmembrane transporter activity were enriched (Figure 6B). To identify the candidate gene, we conducted qRT-PCR to analyze the expression of *GgIFS* genes under normal conditions and with IAA treatment. The qRT-PCR results revealed that a root-specific gene, *GgIFS9*, was most significantly up-regulated after IAA treatment (Figure S4).

### 2.8. Expression Pattern of Up-Regulated Genes in the Transcriptome After IAA Treatment

**Figure 7:**
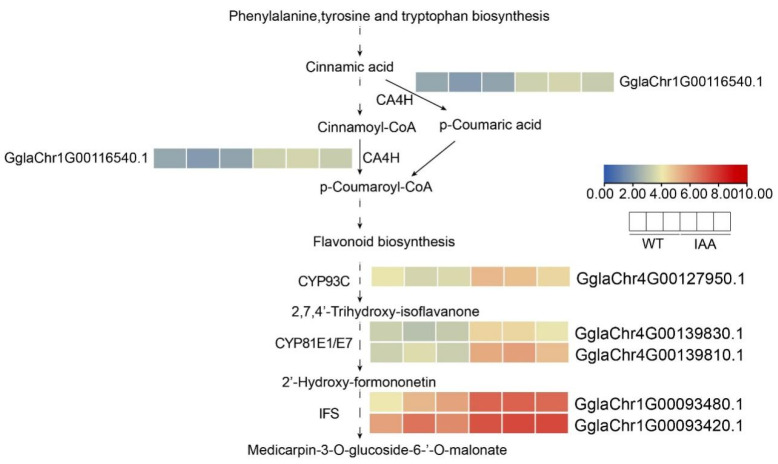
Expression analysis of DGEs in the phenylpropanoid metabolic pathways. Dashed arrows indicate that intermediate steps are involved in the reaction, whereas solid arrows denote that the reaction proceeds in a single step without intermediates.

In the DEGs, some up-regulated genes were found in the phenylpropanoid metabolic pathways (Figure 7). These genes included *cytochrome P450 monooxygenase 93C (CYP93C)*, *cytochrome P450 monooxygenase 81E1/E7 (CYP81E1/E7)*, *trans-cinnamate 4-monooxygenase (CA4H)* and *2-hydroxyisoflavanone synthase (IFS)*. These results revealed that IAA treatment can enhance flavonoid accumulation via the phenylpropanoid metabolic pathway.

### 2.9. GUS Staining and GUS Activity of the GgIFS9 Promoter After IAA Treatment

**Figure 8:**
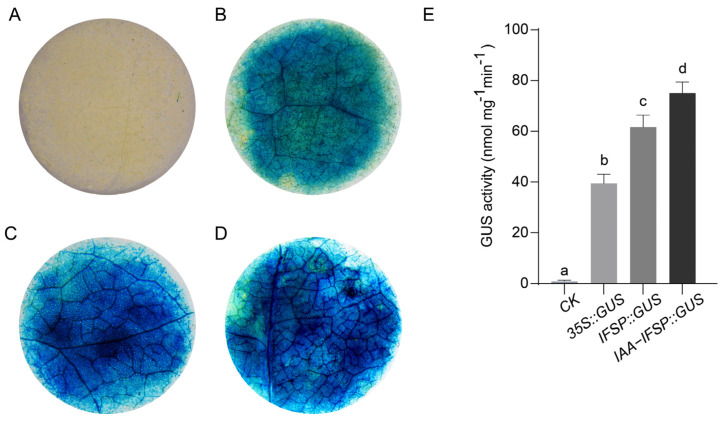
GUS staining and GUS activity of the *GgIFS9* promoter. (**A**) The negative control (the *pCAMBIA1304* empty vector without the 35S promoter) (CK) subject to GUS staining. (**B**) The positive control (the *pCAMBIA1304* empty vector with the 35S promoter) (35S::GUS) subject to GUS staining. (**C**) GUS staining of the *pCAMBIA1304*-*GgIFS9* (IFSP::GUS) promoter under normal conditions. (**D**) GUS staining of the *pCAMBIA1304*-*GgIFS9* (IAA_IFSP::GUS) promoter after IAA treatment. (**E**) GUS activity of the *pCAMBIA1304* empty vector without the 35S promoter (CK), the *pCAMBIA1304* empty vector with the 35S promoter (35S::GUS), the *pCAMBIA1304*-*GgIFS9* promoter under normal conditions (IFSP::GUS) and the *pCAMBIA1304*-*GgIFS9* promoter after IAA treatment (IAA_IFSP::GUS). The a, b, c, d represent significant differences among them. Scale bar = 2 mm. Error bars represent the SD (*n* = 3).

In order to investigate the activity of the *GgIFS9* promoter, we conducted GUS staining on the negative control, positive control, and *pCAMBIA1304-GgIFS9* promoter under normal conditions and under IAA treatment in *N. tabacum*. The leaf of the negative control was not blue, and the leaf of the positive control was blue (Figure 8A,B). After IAA treatment, the blueness of the leaf was darker than that under normal conditions (Figure 8C,D), indicating that IAA treatment enhanced the activity of the *GgIFS9* promoter. Then, we quantitatively analyzed the GUS activity, with the results showing that the GUS activity of the negative control (CK) was the lowest (Figure 8E). The GUS activity of the *pCAMBIA1304-GgIFS9* promoter was higher than the GUS activity of the *pCAMBIA1304* empty vector with the 35S promoter, indicating that the *GgIFS9* promoter was more active than the 35S promoter. After IAA treatment, the GUS activity of the *pCAMBIA1304*-*GgIFS9* promoter was higher than that under the normal condition, further illustrating the IAA treatment enhanced the activity of the *GgIFS9* promoter.

## 3. Discussion

*G. glabra* is an important medicinal plant rich in medicinal components, such as triterpene saponins and flavonoids [18]. Isoflavone has many functions, such as anti-tumor, anti-cardiovascular disease, immune regulation, antibacterial and anti-inflammatory properties [19,20]. IFS can convert flavonoids into isoflavones, and the *IFS* gene is a key gene in deciding the concentration of isoflavonoids [21]. In recent years, researchers have identified *IFS* genes in many plants species, such as *Trifolium pratense*, *Cicer arietinum L.*, *Lotus japonicus*, soybean and other legumes [22,23,24,25,26]. However, research on the three *Glycyrrhiza* species and investigations of the molecular mechanism of isoflavonoids are limited. In this study, we identified 9 *GgIFS* genes, 9 *GiIFS* genes and 10 *GuIFS* genes. These members could be divided into four clades in the phylogenetic tree (Clade A–D), in contrast to the six clades of 139 IFS proteins [27].

Interacting with the promoter of genes, transcription factors play pivotal roles in their regulatory networks [28]. Previous studies have indicated that MYB and bHLH TFs are related to the synthesis of isoflavones [29,30]. This research revealed that *GmMYB29* and *GmMYB133* regulate the GmIFS2 promoter, and the expression of *GmIFS2* is activated, participating in the synthesis of isoflavone [31]. In our current investigation, through Y1H, dual-luciferase assays and an EMSA (Figure 5), we found that the auxin response factor GgARF4 interacted with *GgIFS9*.

There are various hormone-responsive elements in the promoter region of *GgIFS* genes (Figure 4A,B), which suggests that hormones may affect the expression of *GgIFS* genes. The results of qRT-PCR revealed that, after IAA treatment, some *GgIFS* genes were highly expressed, especially the *GgIFS9* gene (Figure S4), which reflects the results of previous research showing that *IFS* genes are differentially regulated under IAA treatment in soybean [32].

In recent years, RNA-seq has become an important technique for profiling targets of DEGs under particular conditions [33]. The ARF is an important transcription factor in the plant auxin signaling pathway [34]. The research revealed that ARF2 positively regulated flavonols biosynthesis in *A. thaliana* [35]. Through the Aux/IAA-ARF signaling pathway, auxin regulates anthocyanin biosynthesis in apple [36]. The results indicated that auxin affected flavonol accumulation through the ARF by regulating the flavonoid biosynthesis genes [37]. The flavonoid biosynthesis pathway is impacted by several plant hormones, transcription factors and non-coding RNAs [38]. Through the aux/IAA-ARF signaling pathway, flavonoid biosynthesis is regulated by auxin (indole-3-acetic, IAA) [39]. In anthocyanin biosynthesis, auxin negatively regulates flavonoid biosynthesis [40]. Therefore, we conducted RNA-seq analysis of the wild-type and IAA-treated *G. glabra*. Compared to the control, the isoflavonoid biosynthesis and flavonoid biosynthesis pathways were enriched after IAA treatment (Figure 6), indicating that IAA may modulate isoflavonoid biosynthesis and flavonoid biosynthesis. Compared to that under normal conditions, the promoter activity of GgIFS9 was significantly enhanced under IAA treatment (Figure 8), suggesting that IAA may act as a positive regulator of isoflavone biosynthesis. Further work is needed to determine the content of flavonoids and for metabolite quantification under IAA treatment and normal conditions by using LC-MS/HPLC in *G. glabra*.

Collectively, these findings have deepened our understanding of the molecular mechanism by which ARF mediates the IAA signaling pathway to regulate the *IFS* genes in *G. glabra*, perhaps influencing the synthesis and accumulation of isoflavones. This provides valuable insights for future research and potential applications in crop improvement.

## 4. Materials and Methods

### 4.1. Identification and Characterization of IFS Genes

The protein sequences of AtIFS were downloaded from the TAIR database (https://www.arabidopsis.org, accessed on 4 August 2024) [41]. We used the IFS sequences of *A. thaliana* as the query to blast the genomic data of *G. uralensis*, *G. inflata*, *G. glabra* and *G. max* with an E-value of 1 × 10^−5^. Then, the Pfam database (http://pfam.xfam.org/, accessed on 6 August 2024) and NCBI-CDD (https://www.ncbi.nlm.nih.gov/cdd/, accessed on 6 August 2024) were used to verify the domain [42]. Proteins without the domain were removed. We used the database ExPASy (https://web.expasy.org/protparam/, accessed on 7 August 2024) to analyze the physicochemical properties, including the number of amino acids, molecular weight (MW), isoelectric point (pI), instability index, aliphatic index and grand average of hydropathicity (GRAVY) [43]. We used the website for Euk-mPLOC (http://www.csbio.sjtu.edu.cn/bioinf/euk-multi-2/, accessed on 8 August 2024) to predict the subcellular localization of IFS proteins [44].

### 4.2. Phylogenetic Tree Construction

We used the IFS protein sequences to perform phylogenetic analysis in *G. uralensis*, *G. inflata*, *G. glabra*, *G. max* and *A. thaliana*. Using the MEGA X software (version 10.2.6, www.megasoftware.net, accessed on 15 August 2024), we constructed the phylogenetic tree using the Neighbor-Joining (NJ) method with 1000 bootstrap repeats [45]. The phylogenetic tree was visualized on the online tool Evolview (https://evolgenius.info/, accessed on 20 August 2024) [46].

### 4.3. Chromosome Distribution of IFS Genes in Three Glycyrrhiza Species

Using the genomic data and the gff file, we determined the position of *IFS* genes on the chromosome in three *Glycyrrhiza* species. Using TBtools software (Version 2.056), we drafted a chromosome distribution map [47].

### 4.4. Gene Structure and Motif Analysis of the IFS Gene Family Members in Three Glycyrrhiza Species

We used the online tool Gene Structure Display Server 2.0 to visualize the gene structure of *IFS* genes [48]. The online tool MEME (http://genocat.tools/tools/meme.html, accessed on 20 August 2024) was used to obtain the motifs, and the results were presented using TBtools software (Version 2.056) [49].

### 4.5. The Analysis of IFS Promoter Genes in Three Glycyrrhiza Species

The 1500 bp upstream genomic sequences of the *IFS* genes were analyzed using PlantCARE (http://bioinformatics.psb.ugent.be/webtools/plantcare/html/, accessed on 25 August 2024) to search for the distribution of *cis*-regulatory elements [50]. We used the TBtools application (Version 2.056) to draft a heatmap of *cis*-regulatory elements.

### 4.6. Synteny and Duplication Analysis

We used the Multiple Collinearity Scan toolkit to analyze the duplication events [51]. The collinearity analysis was mapped using the TBtools application (Version 2.056). The collinear gene pairs of *IFS* gene family members among the three *Glycyrrhiza* species were listed in Table S3. We calculated the synonymous (Ks), non-synonymous (Ka), and Ka/Ks ratios using the KaKs Calculator (https://sourceforge.net/projects/kakscalculator2/, accessed on 25 August 2024).

### 4.7. Yeast Library Screening and Y1H Assay

The 1500 bp promoter fragments of *GgIFS9* were cloned from *G. glabra* and then inserted into the *pHIS2* vector (Clontech, Mountain View, CA, USA). The *pHIS2* vector was linearized and transformed into the yeast strain Y187. The transformed yeast cells were evenly plated on SD-Trp/-Leu with different concentrations of 3-aminotriazole (3-AT) to determine the minimal inhibitory concentration of 3-AT, followed by incubation at 30 °C for 3–5 days. The minimal 3-AT concentration that could significantly inhibit yeast strain growth was selected for yeast library screening. Through yeast library screening, candidate transcription factors (TFs) were identified (Table S4). The coding sequences of TFs were cloned and then inserted into the *pGADT7* vector (Clontech, Mountain View, CA, USA). The promoter of *GgIFS9* was inserted into the *placzi* vector (Clontech, Mountain View, CA, USA). The yeast strain EGY48 was co-transformed with JG45 prey vectors containing candidate TFs and the promoter of *GgIFS9*, followed by incubation at 30 °C for 3–5 days.

### 4.8. In Vivo Dual-Luciferase Assay

In the dual-luciferase (LUC) reporter assay, 1500 bp promoter fragments upstream of the *GgIFS* gene from genomic DNA were amplified and individually cloned into the *pGreenII 0800-LUC* vector before the firefly luciferase LUC reporter gene. The *GgARF4* gene was inserted into the *pGreenII 62-SK* vector. These vectors were then introduced into *Agrobacterium tumefaciens* strain GV3101 and co-infiltrated into the epidermal cells of *N. tabacum* leaves. After 60 h of incubation, the leaves were daubed with D-Luciferin potassium, and then the signals of the leaves were visualized in the IVScope 7000 plant in vivo imaging system (Shanghai Qinxiang Scientific Instrument Co., Ltd., Shanghai, China). The activities of LUC and REN (Renilla luciferase) were quantified using the Dual-Luciferase Reporter Assay System (Promega Corp., Madison, WI, USA). Three independent biological replicates were conducted in the experiment.

### 4.9. EMSA

We used the Thermos Scientific Lightshift Kit (ThermoFisher, Shanghai, China) to perform the EMSA. The promoter fragment of the *GgIFS9* gene was labeled with biotin of the probe. The probe sequence was listed in Table S6. Three independent biological replicates were conducted in the experiment.

### 4.10. RNA-Seq Analysis and qRT-PCR Analysis of GgIFS Genes 

With a temperature of 25 °C, a 16 h/8 h night cycle a day, and 60–70% relative humidity, we grew *G. glabra*. Using 2 µM IAA and water, we applied the treatment to *G. glabra*. The roots of *G. glabra* were frozen in liquid nitrogen and then stored at −80 °C until use. Using the primer premier software, we designed the qRT-PCR primers, and the total RNA was extracted using the RNAprep Pure Plant Kit (TIANGEN, Beijing, China) [52]. Using agarose gel (1.0%) electrophoresis, we assessed the quality of the extracted RNA, and using NanoDrop One (Thermo Fisher Scientific, USA, Waltham), we measured the concentration and purity (OD_260_/OD_280_). An amount of 1 µg of total RNA was reversely transcribed into cDNA using the FastKing One Step RT-PCR Kit (TIANGEN, Beijing, China). Using SuperReal PreMix Plus (SYBR Green) (TIANGEN, Beijing, China), we performed qRT-PCR. The mixture contained 20 µL: 2 × 10 µL SuperReal PreMix Plus, 0.6 µLforward primer, 0.6 µL reverse primer, 2 µL cDNA, and 6.8 µL RNase-free H_2_O. The primers are listed in Table S5. The qRT-PCR reaction conditions were set at 95 °C for 15 min, followed by 40 cycles of 95 °C for 10 s, 60 °C for 20 s, and 72 °C for 20 s, followed by 95 °C for 15 s, 60 °C for 1 min and 95 °C for 15 s. The expression of genes was calculated by the 2^−△△CT^ method, and three biological replicates were taken for each.

Three biological replicates were set for each control. The raw sequencing data were processed by using FastQC (Cambridge, Cambridgeshire, United Kingdom) and Trimmomatic (Forschungszentrum Jülich & University of Düsseldorf, North Rhine-Westphalia, Germany), and low-quality reads were removed to obtain clean reads. The clean reads were mapped onto the *G. glabra* genome using HISAT2. The levels of gene expression were normalized using per kilobase of transcript per Million mapped reads (FPKM). DESeq2 software (Version 1.42.x) was used for screening of the DEGs. The threshold parameters used for screening the DEGs were an FDR < 0.05 and a fold change (FC) ≥ 2. Using the online website for Gene Ontology (GO) (http://geneontology.org/, accessed on 15 September 2024) and KEGG (http://www.genome.jp/kegg/pathway.html, accessed on 25 September 2024), we performed enrichment analysis to identify functional annotations related to metabolic pathways. The DEGs of the RNA-Seq were listed in Table S7.

### 4.11. β-Glucuronidase (GUS) Staining and GUS Activity

The 1500 bp promoter of *GgIFS9* was amplified using genomic DNA as the template. The fragment was inserted into the *pCAMBIA1304* empty vector. The *pCAMBIA1304::GgIFS9* plasmid was inserted into *Agrobacterium tumefaciens* strain GV3101 and co-infiltrated into the epidermal cells of leaves in *N. tabacum*. We used the GUS Straining Kit (Coolaber, Beijing, China) to detect the GUS activity. Three independent biological replicates were conducted in the experiment.

### 4.12. Statistical Analysis

Mean values and standard errors were calculated using Microsoft Excel software. Student’s t test was conducted with the SPSS program (Version 23.0) to determine the significance of differences between the control and treated samples or between time points. The significance threshold was set at *p* < 0.01.

## 5. Conclusions

Through genome-wide identification and analysis, this study comprehensively identified 9, 9, and 10 *IFS* genes in *G. uralensis*, *G. inflata* and *G. glabra*, elucidating the pivotal role of *GgIFS9* in isoflavone biosynthesis in *G. glabra*. This study is the first to identified the molecular mechanism by which auxin response factor GgARF4 regulates *GgIFS9* expression. These findings provide crucial theoretical insights into the regulatory network of plant isoflavone biosynthesis.

## Data Availability

All data are presented in the article and Supplementary Materials. All authors agree with the MDPI Research Data Policies.

## Supplementary Materials

The following are available online at https://www.mdpi.com/article/10.3390/ijms262110435/s1.

## Abbreviations

IFS: Isoflavone synthase; GgARF4: auxin response factor 4 in *Glycyrrhiza glabra*; DEGs: differentially expressed genes; GUS: β-Glucuronidase; qRT-PCR: quantitative reverse transcription chain reaction.
