# Supplementary material for: Genome-Wide Identification and Characterization of Isoflavone Synthase (IFS) Gene Family, and Analysis of GgARF4-GgIFS9 Regulatory Module in Glycyrrhiza glabra
Source: Int J Mol Sci. 2025 Oct 27;26(21):10435. doi: 10.3390/ijms262110435 (PMC12608901; doi:10.3390/ijms262110435)
Supplement: Supplementary file 1 [file ijms-26-10435-s001.zip › ijms-3904395-supplementary.pdf]

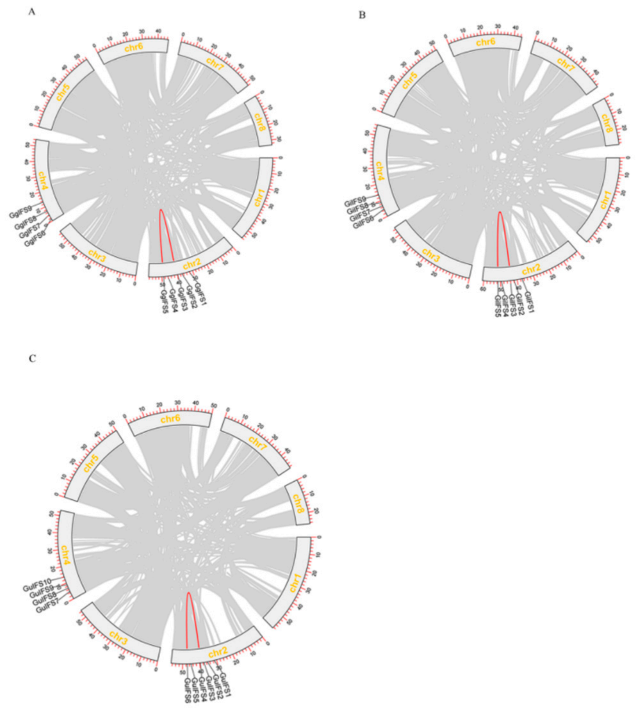

Figure S1. Chromosomal localization and covariance analysis of three *Glycyrrhiza* species. A, B, C corresponds to *G. glabra*, *G. inflata* and *G. uralensis*.

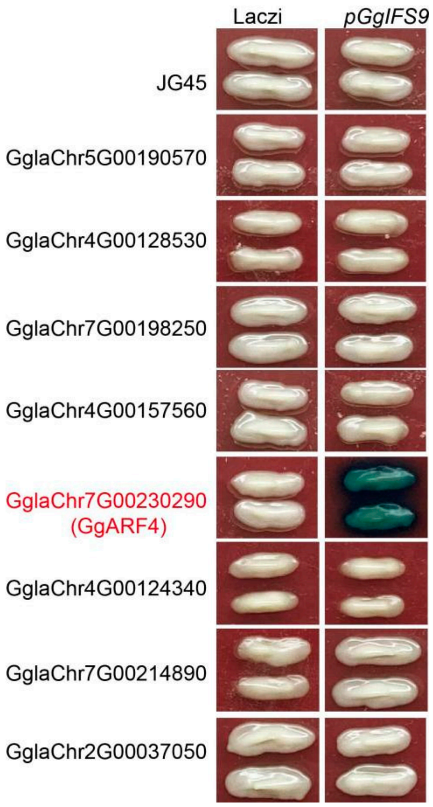

Figure S2. The results of yeast screening and Y1H assay.

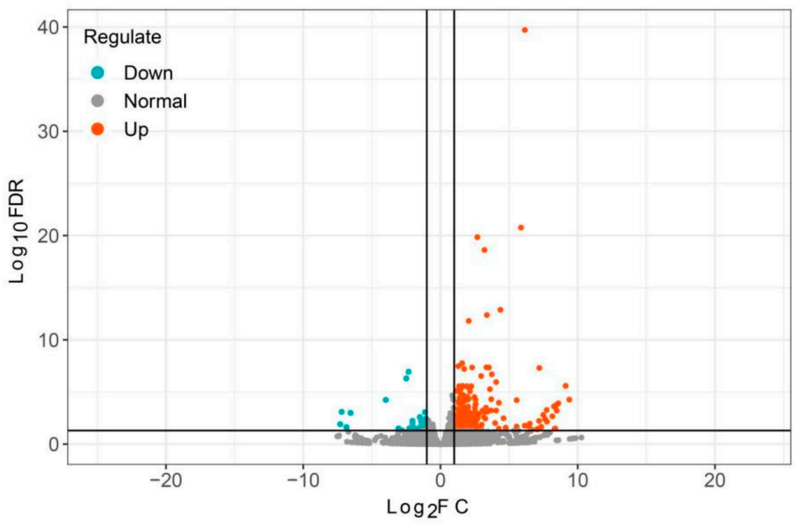

Figure S3. Volcano plot of DEGs.

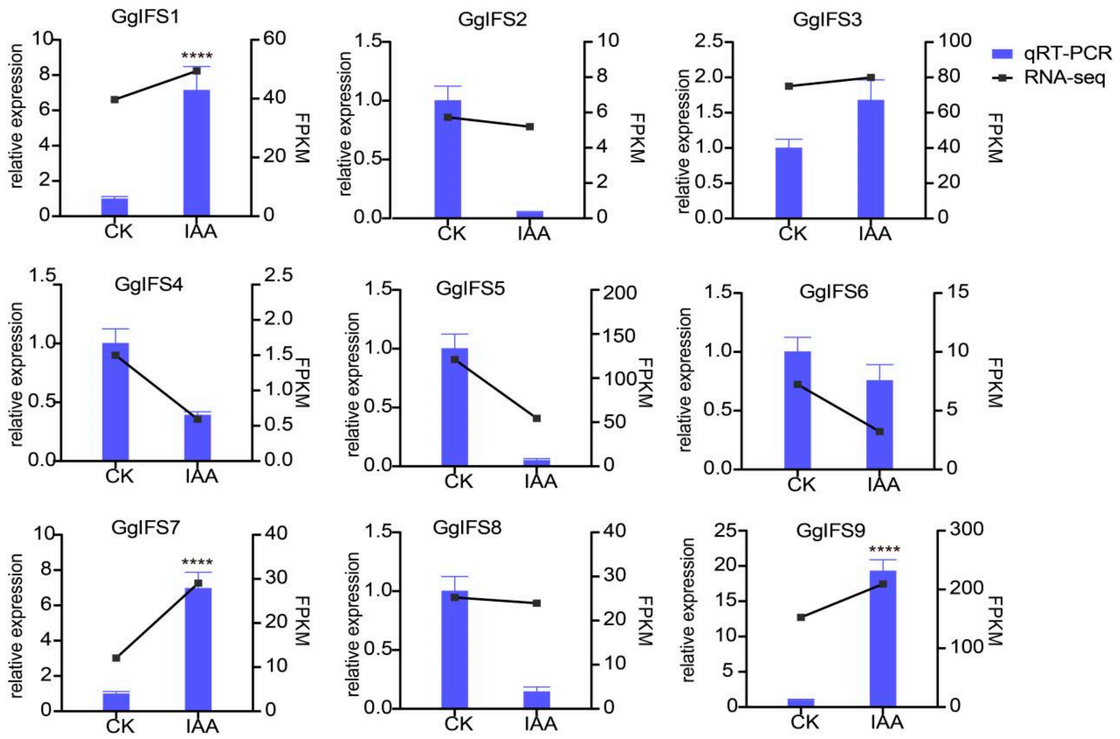

Figure S4. qRT-PCR analysis of GgIFS genes between the wild type and IAA treatment in *G. glabra*. Three independent biological replicates were conducted in the experiment. The t-test was used for significant difference analysis. *, **, and *** denoting significant differences at $p < 0.05$, 0.01, and 0.001 levels, respectively.

Table S1. The protein sequences and CDS sequences of the *IFS* genes in the three *Glycyrrhiza* species.

ID	pep sequence	CDS sequence
GgIFS1	MADFQDYLQIFVICVLSTI<br>LVGSILWRKQNKHPHPP<br>GPLALPIIGHFHLLALLSP<br>LLHSAFHKLSIRYGPIILLF<br>MGSVPCIVVSTAEAAKEF<br>LKTHETSFSNRARTVATE<br>TYSYGLQGIIFTPYGDYW<br>RFIKKLCMSELLGQNILN<br>RFLAVRQQETKRFIKLVL<br>NKGVEGEAVDFGEQFM<br>KLANDIMSRMTISQTSSK<br>NDDEANEMMKMVADIT<br>EILGEFNISEFIWFLKRFN<br>LQGHNKRLKEVHDKFDT<br>RLNRVIKEHEEERRKRKE<br>MGGSGTHQSKDILDVLL<br>DIYEDKSSEMKLTKENIK<br>AFILDIFLAGTDTSALALE<br>WAIAELINNPHVMKKAR<br>QEIDAVVGKSRMVEESD<br>VMNLPYLQAIVKETLRL<br>HPVVPMIFRESSRRDIVG<br>GYDIPAKSKVFINIWAIGR<br>DPNHWENPLEFRPERFIG<br>EDNDQMDVRGQHYHLI<br>PFGSGRRVCPGTSLALQV<br>LHVNLGAVIQCFELKVE<br>GGNGTIDMAEKPGMTM<br>RRAQPLICVPLPRLNPFPS<br>M*	ATGGCTGATTTTCAAGACTATCTTCAAATCTTCGTCATTTGTGTTCTATCCACCAT<br>CCTAGTTGGTTCCATACTATGGAGAAAACAAAACAAGCACCCTCATCCACCAGG<br>TCCCTTAGCCCTACCCATCATTGGTCACTTCCACCTTTTGGCCCTTCTTTCCCCACT<br>ACTTCACTCTGCTTTTCACAAACTCTCAATCCGCTATGGCCCCATAATACTCCTTT<br>TCATGGGCTCTGTCCCTTGTATTGTAGTTTCCACCGCAGAAGCTGCCAAAGAGTT<br>TCTCAAAACTCATGAAACCTCCTTTTCCAACCGTGCAAGAACTGTTGCCACTGAG<br>ACCTATAGCTATGGTCTTCAAGGCATAATCTTTACACCCTATGGAGACTATTGGA<br>GGTTTATTAAGAAATTGTGCATGTCTGAACTTCTTGGTCAAAATATATTGAATCG<br>ATTCCTTGCTGTGAGGCAGCAAGAGACAAAGAGGTTCATCAAACTTGTGCTCAA<br>TAAAGGGGTGGAAGGTGAGGCTGTGGATTTTGGGGAACAATTCATGAAGCTTGC<br>AAATGATATCATGTCAAGAATGACCATTAGTCAAACAAGTTCTAAGAATGATGA<br>TGAAGCAAACGAGATGATGAAGATGGTGGCAGATATTACAGAGATCTTAGGGG<br>AGTTCAATATATCGGAATTCATTTGGTTCTTGAAGAGGTTCAATCTACAGGGACA<br>CAACAAGAGGCTAAAGGAAGTTCATGACAAGTTTGATACCAGGCTGAACAGAG<br>TGATAAAAGAGCATGAAGAGGAAAGAAGGAAGAGAAAGGAAATGGGTGGAAG<br>TGGAACTCATCAATCTAAGGATATACTTGATGTGTTATTAGATATATATGAAGAT<br>AAAAGTTCAGAAATGAAATTGACTAAAGAGAACATAAAGGCCTTCATCTTGGAC<br>ATATTTCTTGCTGGGACTGACACGTCAGCATTGGCCCTAGAATGGGCTATAGCAG<br>AATTGATCAACAACCCACATGTGATGAAGAAAGCAAGGCAAGAGATAGATGCA<br>GTGGTTGGAAAGAGTAGAATGGTGGAAGAATCTGATGTTATGAATCTTCCTTACT<br>TACAAGCCATAGTCAAAGAAACATTAAGGCTTCACCCTGTAGTTCCGATGATTTT<br>CAGGGAGTCATCTAGAAGGGATATAGTTGGTGGTTATGATATTCCAGCCAAGTCT<br>AAAGTATTTATCAATATTTGGGCTATTGGTAGGGACCCCAATCACTGGGAGAAC<br>CCACTTGAGTTTAGGCCAGAAAGGTTTATAGGTGAAGATAATGATCAAATGGAT<br>GTTAGGGGACAACATTATCATTTGATTCCATTTGGGAGTGGAAGAAGAGTGTGTC<br>CTGGAACCTCTTTGGCATTGCAGGTTTTGCATGTGAACCTGGGTGCTGTGATTCA<br>ATGTTTCGAGTTGAAGGTTGAAGGTGGCAATGGCACGATTGACATGGCAGAGAA<br>GCCTGGCATGACTATGCGCAGGGCTCAACCTCTCATCTGTGTCCCTCTTCCAAGG<br>CTTAATCCATTTCCTTCTATGTGA
GgIFS2	MADYQSYTLLFILWLVSII<br>VVRTILTRKQNKAHVPPS<br>PLALPIIGHLHLLAPIPHQ<br>ALHKLSTRYGPIMQLSLG<br>SVPCLVASTPESAKEFLKI<br>HETKFSNRPQSSAVHYLT<br>YGSQDFSFAPYGPYWKF<br>MKRICMSELLGGHTLTLL<br>LPVRKQETTRFLRLLLKK<br>GNAGETVDVGGELLTLS<br>NNIVSRMIMSQTCSEEDD<br>GEGEEVVRKLVQDTVLL	ATGGCTGATTACCAAAGCTACACCCTACTTTTTATCCTATGGCTAGTGTCCATAAT<br>CGTGGTTCGAACCATTCTCACCAGAAAACAGAACAAGGCTCATGTACCACCAAG<br>CCCACTAGCCTTACCCATAATTGGACACCTTCACCTTCTTGCACCAATACCCCAT<br>CAAGCTCTTCACAAGCTATCAACACGCTATGGACCCATAATGCAACTTTCCCTTG<br>GTTCCGTCCCTTGCCTCGTGGCTTCAACACCAGAGTCCGCCAAAGAGTTCCTTAA<br>AATCCACGAGACCAAATTCTCCAACCGTCCTCAAAGCTCTGCTGTTCATTACCTA<br>ACATATGGTTCCCAAGACTTCTCCTTTGCCCCATATGGACCTTATTGGAAATTCAT<br>GAAGAGAATATGCATGTCTGAACTTCTTGGGGGTCACACACTCACACTGCTTCTT<br>CCCGTGAGAAAACAAGAGACGACAAGGTTTCTTAGACTCTTGCTAAAGAAAGG<br>GAATGCAGGTGAGACTGTTGATGTTGGAGGGGAACTTCTGACACTGTCGAATAA<br>CATTGTTTCGAGGATGATTATGAGCCAAACTTGCTCAGAAGAAGATGATGGGGA<br>AGGGGAAGAAGTGGTGAGGAAGTTGGTGCAAGACACGGTGCTTCTCACGGGGA

	TGKFNVSDFVWFFKNWD	AGTTTAATGTCTCGGACTTTGTTTGGTTCTTTAAGAATTGGGATTTGCAGGGGTTT
	LQGFGKRLREIRERFDTM	GGTAAAAGGCTTAGGGAGATTCGGGAGAGGTTTGATACCATGATGGAGAAGGT
	MEKVIKEHEEERRKRKEV	GATCAAGGAGCATGAAGAGGAGAGGAGGAAGAGAAAAGAAGTTGGTGGAGGA
	GGGDGRVKDLLDILLDIA	GATGGTCGAGTCAAGGATCTACTCGATATATTGTTGGATATAGCTGAAGATGAG
	EDESSDIKLTMENIKAFIL	AGCTCTGACATAAAATTGACAATGGAGAACATAAAGGCCTTCATCTTGGACATA
	DIFMAGTDTSALTTEWA	TTTATGGCAGGAACAGACACTTCAGCGCTAACCACTGAATGGGCTCTGGCAGAG
	LAELINHPNVMERARKEI	TTGATTAACCACCCAAATGTGATGGAGAGAGCAAGAAAAGAGATTGATGATGT
	DDVVGKTRIVEESDLVNL	AGTTGGGAAAACTAGAATAGTAGAAGAATCAGACCTTGTCAACCTTCCTTATCT
	PYLQAIVKETLRIHPTGPL	ACAAGCTATAGTCAAAGAAACACTGAGGATTCACCCCACAGGTCCTTTAATTGT
	IVRESSEKCTIWGYEIPAK	TAGAGAATCATCTGAAAAATGCACCATATGGGGCTATGAGATTCCAGCAAAGAC
	TQLFVNVWSIGRDPNHW	ACAATTGTTTGTTAATGTGTGGTCTATTGGCAGGGACCCCAATCACTGGGAGAAC
	ENPLEFKPERFIGEEGSGK	CCACTTGAGTTCAAACCAGAGAGATTTATTGGTGAGGAGGGAAGTGGAAGAGT
	SQLDVRGQHFHLIPFGSG	CAGTTAGATGTGAGGGGACAACATTTTCACCTAATTCCATTTGGGAGTGGAAGA
	RRGCPGTSLALQVVQTN	AGAGGGTGCCCTGGAACCTCACTGGCTTTGCAGGTTGTGCAGACAAACCTTGCT
	LAAMIQCFEWKVNNNA	GCTATGATTCAGTGTTTTGAATGGAAGGTTAATAATAATGCAGGCAATAATTATG
	GNNYETVVNMEEKPGLT	AGACTGTAGTTAACATGGAAGAGAAACCTGGGTTAACACTTTCAAGGGCTTATC
	LSRAYPLICVPMPRLSPFP	CGTTAATATGTGTCCCAATGCCTAGGCTTAGTCCTTTTCCTTCAATGTGA
	SM*	
	MAEIQDFIQLFLIWLLSTI	ATGGCTGAAATCCAAGACTTTATCCAACTTTTCCTAATTTGGCTACTCTCCACCAT
	AIRAILTRKQNNKEGHSQ	TGCAATTCGAGCCATACTAACCCGAAAACAGAACAACAAGGAGGGTCATAGCC
	SHPPSPLALPIIGHLHLIS	AAAGCCACCCACCAAGCCCTCTAGCACTACCCATAATTGGACACCTTCACCTTA
	QLPHQSFHNLSTRYGPIM	TTTCTCAATTACCCCACCAAAGCTTCCACAACCTCTCAACTCGCTATGGACCCAT
	QIFLGSVPCVVASTPEIAK	CATGCAAATCTTCCTAGGCTCTGTCCCTTGTGTAGTAGCTTCCACCCCAGAAATC
	EFLKTNEASFSNRFRSAA	GCCAAGGAGTTCCTCAAAACCAATGAAGCATCCTTCTCCAACCGTTTCAGAAGT
	VHYLSYGSKGFLFAPYGE	GCTGCGGTTCACTATTTATCGTATGGCTCAAAGGGGTTCTTGTTTGCGCCTTATGG
	YWKFMKKLCVSELLGGR	AGAGTACTGGAAGTTCATGAAGAAGCTCTGTGTATCTGAGCTTCTCGGTGGAAG
	TLDQLSPLRKQETVRFLR	AACACTCGATCAGCTAAGTCCACTGAGGAAACAAGAGACTGTGAGGTTTCTGAG
	LMQSKGEAGVAIDVGGE	GCTTATGCAGAGTAAAGGAGAAGCTGGTGTTGCTATTGATGTTGGTGGGGAGCT
	LLTLANSVITRMTMSKTC	ATTGACACTTGCAAATAGTGTCATAACGAGGATGACTATGAGCAAAACGTGTTT
	FENDGDVEDIRKMVKDT	TGAAAATGATGGTGATGTGGAAGATATTAGGAAGATGGTGAAGGACACTGCGG
GgI	AELAGKFNVSDFIWFCK	AGCTTGCTGGGAAGTTTAATGTGTCTGACTTTATTTGGTTCTGTAAGAATTTGGAT
FS3	NLDLQGMNKRLKEILDR	CTGCAGGGGATGAACAAGAGGCTTAAGGAGATTCTTGATAGGTTTGATACCATG
	FDTMMERVIREHEVERK	ATGGAGAGGGTGATAAGGGAGCATGAAGTGGAAAGGAAGAGAAGGAAGGAAA
	RRKERGEEGANQVMDLL	GGGGTGAAGAAGGAGCTAATCAAGTTATGGACTTGCTTGATATTTTGTTGGAAAT
	DILLEIQGDERTEMKLTR	ACAGGGTGATGAGAGAACTGAGATGAAACTGACCAGAGAGAATGTCAAGGCTT
	ENVKAFILDIFMAGTDTS	TCATCTTGGACATATTTATGGCAGGAACTGACACGTCAGCTATAACGATCGAAT
	AITIEWALAELINHPHVM	GGGCTCTTGCTGAGTTAATCAACCACCCGCATGTGATGGAGAAAGCAAGACAAG
	EKARQEIDSVIGKNRLIEE	AAATCGATTCAGTAATAGGAAAGAATAGATTAATAGAAGAGTCAGATCTTCCCA
	SDLPNLPYLRAIVKETLRL	ACCTTCCTTACTTGCGAGCTATAGTTAAAGAAACATTAAGGCTTCACCCTGCAGC
	HPAAPLLGRESSESCNVC	ACCACTTCTAGGGAGAGAATCATCTGAAAGTTGCAATGTTTGCGGGTATGAAAT
	GYEIPAKTVVFVNLWSM	TCCAGCGAAGACAGTTGTATTTGTTAATTTGTGGTCAATGGGTAGGGACCCAAAC
	GRDPNMWENPLDFRPER	ATGTGGGAGAACCCACTTGATTTCAGGCCAGAGAGGTTTATAATGGGTGAAGAG
	FIMGEESVQLDVRGQNF	AGTGTTCAGTTAGATGTGAGGGGACAGAATTTCCAACTAATGCCATTTGGGACTG
	QLMPFGTGRRACPGASL	GAAGAAGGGCATGTCCTGGTGCCTCACTTGCACTTCAGATGGTTCCCACTAACCT

	ALQMVPTNLAAMVQCF	TGCTGCTATGGTTCAGTGCTTTGAATGGAAGGTTGTTGATGATGGTGGAGATGGT
	EWKVVDDGGDGNNGK	AATAATGGGAAAGTTAGCATGGAAGAGAAACCAGGCATGACCCTTCCAAGGGC
	VSMEEKPGMTLPRAHPL	TCATCCTTTGACGTGTGTCCCTGTGCCTCGTTTTGATTGCATTCCTTCTTGTGTATA
	TCVPVPRFDCIPSCV*	G
	MADFGNYFGLLLIVLSSIL	ATGGCTGATTTTGGTAACTATTTTGGGCTACTTCTCATTGTTCTATCCTCAATCCTC
	LFRVIFTNNKACLPPSPR	TTGTTCCGAGTGATCTTCACCAACAATAAAGCATGTCTTCCACCAAGCCCTCGTG
	ALPVLGHLYLLTNLPHQ	CCTTACCCGTTCTGGGACACCTCTACCTTCTCACAAACCTCCCTCACCAAGCATT
	AFQKISSRYGPLVYLLFGS	CCAAAAAATCTCATCCCGTTATGGCCCTTTGGTCTACCTCTTGTTTGGTTCCAAAC
	KHCVLVSSPEMAKQCLK	ACTGTGTTCTAGTCTCTTCCCCTGAAATGGCCAAACAATGCCTTAAAACCAATGA
	TNESCFLNRPKRTNVDYI	GTCCTGTTTCCTAAACCGACCCAAGAGAACCAACGTGGACTACATCACATACGG
	TYGSSDFVMAPYGPYWR	TTCCTCAGATTTTGTAATGGCACCCTATGGACCCTATTGGAGGCTCATGAAGCGG
	LMKRLCMNELLGGRILH	CTTTGCATGAATGAACTCCTTGGTGGTCGAATACTTCACCAGCACCTTCCCATTA
	QHLPIRAEEIKLFLKGMM	GAGCTGAAGAGATTAAGCTTTTCTTGAAGGGTATGATGAAAAGGTCTGATTTTGG
	KRSDFGERVNVGEELSLL	AGAGAGGGTGAATGTTGGGGAGGAACTTTCTTTGCTTTCGAATAACATCATCACA
	SNNIITRMALRRRCSDVE	AGGATGGCTTTGAGAAGAAGGTGTAGTGATGTTGAAGGGGAAGGGCACCAATT
	GEGHQLIELVREMTELGG	GATTGAGCTTGTGAGGGAGATGACTGAGCTAGGTGGGAAGTTCAACTTGGGAGA
	KFNLGDMLWFVKRLDL	CATGTTGTGGTTTGTTAAGAGGCTTGATTTGCAAGGGTTTGGTAAGAGGTTGGAG
	QGFGKRLESVRSRYDAIM	AGTGTTAGGAGTAGGTATGATGCTATAATGGAGAGGATCATGAAGGAACATGA
	ERIMKEHEDARMVKKK	AGATGCAAGGATGGTGAAGAAGAAAAATAAGGATAGTTGTAATGGAGATGAAG
	NKDSCNGDEAVRDLLD	CAGTGAGGGATTTACTTGATAACATGTTTGGTGCTGGAACTGAGACATCAGCATG
	NMFGAGTETSACTIEWA	TACAATAGAATGGGCTTTGGCCGAGCTAATAAACCATCCAGACATGATGGAAAG
	LAELINHPDMMERARQE	AGCAAGACAAGAGATTGATTCAGTGGTTGGTAAGAGCAGATTGGTGGAGGAAT
	IDSVVGKSRLVEESDIPNL	CAGATATTCCCAACCTTCCCTATGTTCAATCCATAGTGAAAGAAACAATGAGGC
GgI	PYVQSIVKETMRLHPTGP	TTCACCCAACTGGGCCTCTAATAGTGAGGCAATCAACTGAGGATTGCAATGTTG
FS4	LIVRQSTEDCNVDGYHIP	ATGGGTATCACATTCCAGCAAGGACTTCTGTGTTTGTTAATGTGTGGGCCATTGG
	ARTSVFVNVWAIGRDPK	TAGGGACCCAAAATACTGGGAAAGCCCACTTGAGTTTAAGCCAGAGAGGTTCCT
	YWESPLEFKPERFLNEEG	CAATGAGGAGGGACAGAGCATGTTGGATCTGAAGGGGCAGAATTTTGAACTGTT
	QSMLDLKGQNFELLSFG	GTCTTTTGGTGCTGGAAGGAGAAGTTGCCCTGGTGCTTCACTAGCCTTGCAGATT
	AGRRSCPGASLALQIIYTT	ATTTATACAACACTGGCTGGTATGGTTCAGTGTTTTGAATGGAAGGTTGGTGAAG
	LAGMVQCFEWKVGEEG	AAGGGAATAATAATGGAACCATTGACATGGAGGAGGGTCCTGGAATGGCACTT
	NNNGTIDMEEGPGMALP	CCTAGGGCAAATCCCCTGATATGCACCCCTGTGATTAAAAAGAAAGCTGAAGCA
	RANPLICTPVIKKKAEAL	CTTGAGGCTGTTAATGTTACTGATGAGCTATTGAAGCTAACAAACAGTGTGGTAA
	EAVNVTDELLKLTNSVV	TGAGAATGGCAATAAGCAAGAGCTGTTTCAACAGGGATGATGAGGCTCACAAG
	MRMAISKSCFNRDDEAH	GTGACAGAGAGGGTGAGAGAGTCTTCCATGTTGAGTGGAATGTTTAACCTTGCG
	KVTERVRESSMLSGMFNL	GATTACTTTTGGTTTTGCAAAAAGTTGGATCTTCAGGGAATGGGGAAGAGGCTA
	ADYFWFCKKLDLQGMG	AAGGAGGTTCATGACAGGCTTGACACCATGATGGAGAGTATCATTCAGGAGCAT
	KRLKEVHDRLDTMMESII	GAAGAGGCAAGAAGAGGTGAATCGACCAGAAATAAGGATGCTACAAAGGATG
	QEHEEARRGESTRNKDA	TGCTTGATGCTCTTTTGAGTATTTATGAAGATCAAAGCTCAGAGGTCAAAATAAC
	TKDVLDALLSIYEDQSSE	CAGAGACAACATTAAAGCCTTCTTGGTGGATATGTTCACAGGTGGGACAGATAC
	VKITRDNIKAFLVDMFTG	AACTGCTGTGACTCTAGAATGGTCATTGGCAGAACTAATTAACCACCCAACAGT
	GTDTTAVTLEWSLAELIN	GATGGAGAAAGCAAGGAAGGAAATTGACTCTGTGATTGGCAAGGACAGAACAG
	HPTVMEKARKEIDSVIGK	TAGTGGAATCAGACATAGCTGATCTTCCTTATCTCCAAGCCATAGTGAAGGAGA
	DRTVVESDIADLPYLQAI	CACTAAGGCTTCACCCTCCATCTCCATTTATATTGAGAGAATCAACTGAGGACTG
	VKETLRLHPPSPFILREST	CACCGTTGCTGGGTTTGATATCCCATCAAAAACTCAGATCTTCACTAATGTGTGG

	EDCTVAGFDIPSKTQIFTN	GCCATTGGAAGGGATCCAAAGCAATGGGATAACCCTCTTGAGTTTAGGCCAGAA
	VWAIGRDPKQWDNPLEF	AGGTTTCTTAGAAGGGAGAATCAAGTTGAATTAAGAGGGCAACATTATCAGCTT
	RPERFLRRENQVELRGQ	TTGCCTTTTGGGAGTGGAAGAAGAAGGTGCCCTGGAACTTCACTAGCACTAAAT
	HYQLLPFGSGRRRCPGTS	GTTGCCCACACCACCCTTGCTGCTATGATTCAATGCTTTGAATGGAAGGGTGATC
	LALNVAHTTLAAMIQCF	AAGATGGAGGAGGGGATGGTAGTGTTGACATGAAAGAGGGTCCTTCATTCATTC
	EWKGDQDGGGDGSVDM	TTTCAAGAGCTCAGCCTTTGATTTGTGTCCCAAAACTCAGATTTATGCCATTTCCC
	KEGPSFILSRAQPLICVPK	TCCGTGATCCGTTCCTTGCATCGTAACTTCCAACCCAGAAACCGCCAAAGAGAC
	LRFMPFPSVIRSLHRNFQP	GAAGAGTTCCTCAAAACCCACGAAGCATGCTTCTCCAACCGCGTCAAAGGTTCC
	RNRQRDEEFLKTHEACFS	GCAATTCACTACCTATCATCAAGGAGGTTCTTGTTCGCTCCTTATGGAGAGTACT
	NRVKGSAIHYLSSRRFLF	GGAAGTTCATGAAGAAACTCTGCATGTCTAAGCTTCTCGGTGGCAAAACCATTG
	APYGEYWKFMKKLCMS	AGGAACACAACCTGTTGAGGAAACAAGAGACTTTGAGGTTTCTGAGGCATATGC
	KLLGGKTIEEHNLLRKQE	AGAGTAAAGGGGAAGCCTTTGAGGCCGTTGATGTTGGTGGGGAGTTACTGACTC
	TLRFLRHMQSKGEAFEA	TTAGCAATAATGTTATAACGAGAATGGCTATGAGTAGAACGTGTTCTGAAAATG
	VDVGGELLTLSNNVITR	ATGGCGATGTGGAAGAGATCAAGAAGATGGTGATAGATACTGCGGATCGAGCT
	MAMSRTCSENDGDVEEI	CGTGGGGAAGTTTAA
	KKMVIDTADRARGEV*	
	MLDIQGYLVLFLLWFISTI	ATGCTTGACATCCAAGGCTACCTCGTGCTATTCCTCTTATGGTTCATCTCAACCAT
	LIRSIFKKSQCYKLPPGPPI	TTTGATTAGATCCATCTTCAAAAAATCCCAGTGTTATAAACTACCACCAGGACCC
	SLPLIGHAPYLRSLLHQA	CCAATCTCTTTACCACTCATTGGACACGCTCCTTACCTTAGATCACTCCTCCACCA
	LYKLSTRYGPLMHVLIGS	AGCACTCTACAAACTCTCCACCCGATATGGGCCCTTGATGCACGTCCTCATCGGT
	QHVIVASSAEMAKQILKT	TCGCAACATGTCATCGTTGCGTCCTCTGCAGAAATGGCTAAGCAAATCCTAAAA
	YEESFCNRPIMIASENLTY	ACCTACGAGGAGTCGTTTTGCAACCGTCCGATCATGATCGCGAGCGAGAATTTG
	GAADYFFIPYGTYWRFLK	ACTTACGGCGCCGCCGATTACTTCTTCATTCCATACGGAACCTACTGGAGGTTCC
	KLCMTELLSGKTLEHFVN	TTAAGAAGCTATGCATGACAGAGCTTCTTAGTGGGAAGACGCTTGAGCACTTTGT
	IREDEIKCFLRNVLEISKT	CAATATCCGCGAGGATGAGATTAAGTGCTTCTTGAGGAATGTTCTGGAGATATCG
	GKGVEMRHELIRHTNNII	AAGACCGGGAAAGGGGTGGAGATGAGGCATGAGTTGATAAGGCACACAAACA
	SRMTMGKKSNGTNDEV	ACATCATTTCTAGGATGACTATGGGGAAGAAGAGTAATGGGACAAATGATGAG
	GQVRKLVREIGELLGAFN	GTTGGTCAGGTGAGGAAGTTGGTTAGGGAGATTGGGGAACTTCTTGGTGCTTTTA
	LGDIIGFMRPFDLQGFGK	ACTTGGGTGACATTATTGGGTTCATGAGGCCTTTTGATCTGCAAGGGTTTGGGAA
GgI	KNRDAHHNMDVMMEK	GAAGAATAGGGATGCACACCATAATATGGATGTGATGATGGAGAAGGTGCTCA
FS5	VLKEHEEARAKEKGGAE	AGGAGCATGAAGAGGCCAGGGCAAAGGAGAAGGGTGGTGCTGAGAGTGATAG
	SDRKKDLFDILLNLIEAD	GAAGAAGGATCTATTTGACATTCTGTTGAACTTGATTGAAGCTGATGGTGCTGAT
	GADNKLTRESAKAFALD	AATAAACTCACTAGAGAAAGTGCCAAAGCCTTTGCACTGGACATGTTCATTGCT
	MFIAGTNGPASVLEWSL	GGGACAAACGGACCAGCAAGTGTGTTAGAATGGTCACTGGCAGAGCTGATTAG
	AELIRNPQVFKKAREEID	AAATCCACAAGTTTTCAAGAAGGCAAGAGAAGAGATTGACTCTGTTGTTGGAAA
	SVVGKERLVKESDIPNLP	GGAAAGGCTAGTCAAAGAATCAGACATACCAAACCTACCTTACCTCCAAGCAGT
	YLQAVVKETLRMHPPTPI	GGTCAAAGAAACCCTGAGGATGCACCCACCAACCCCAATCTTTGCAAGGGAAG
	FAREAIRGCQVDGYDIPA	CCATAAGGGGTTGCCAAGTTGATGGCTATGACATCCCAGCAAACTCAAAGATCT
	NSKIFINAWAIGRDPKY	TCATCAATGCATGGGCCATTGGAAGGGATCCAAAGTATTGGGACAACCCACAAG
	WDNPQVYSPERFLITDEP	TTTATAGCCCTGAAAGGTTCTTAATCACTGATGAACCTGGAAAGAGTAAAATTG
	GKSKIDVRGQYYQLLPFG	ATGTTAGGGGGCAATACTACCAATTGTTGCCATTTGGGAGTGGGAGAAGAAGCT
	SGRRSCPGSSLALLVIQAT	GCCCTGGATCCTCACTTGCATTGCTTGTCATCCAAGCAACACTTGCAAGTTTGGT
	LASLVQCFDWVVNDGK	ACAGTGCTTTGATTGGGTTGTGAATGATGGTAAGAATAGTGAGATTGACATGTCA
	NSEIDMSEEGRVTVFLAK	GAGGAAGGAAGGGTAACTGTGTTTTTGGCTAAGCCTCTCAAGTGCAAGCCTGTTC

	PLKCKPVPRFVPFSA*	CTCGTTTTGTTCCATTCTCTGCCTGA
	MESQLMAVSVVLVSALIC	ATGGAATCTCAACTCATGGCGGTGTCTGTAGTGTTGGTTTCAGCACTTATTTGCTA
	YFLFRPYFHRHGKNLPPS	CTTCCTTTTCCGGCCATATTTTCACCGCCACGGAAAAAACCTCCCACCATCTCCTC
	PLFRLPIIGHMHMLGPLL	TGTTTCGGCTTCCAATAATTGGGCACATGCACATGTTGGGTCCCCTTCTCCACCA
	HQSLHNLSHRYGPLFSLN	GTCCCTTCACAATCTCTCTCACCGTTACGGTCCTCTGTTTTCTCTTAACTTTGGCTC
	FGSVLCVVASSPHFAKQL	TGTTCTCTGTGTCGTTGCTTCCTCCCCTCACTTCGCCAAACAACTCCTTCAAACCA
	LQTNELAFSSRIETTAVKR	ACGAACTTGCATTTAGCAGTCGCATTGAAACCACCGCCGTTAAACGCCTCACTTA
	LTYESSLAFAPCGDYWRF	CGAATCCTCATTGGCCTTCGCACCCTGTGGGGATTATTGGAGGTTCATCAAGAAG
	IKKLSMNEFLGSRGMNN	CTTAGCATGAACGAGTTCTTGGGCTCACGTGGCATGAACAACTTCCTGCACGTGC
	FLHVRAHETHRLLRLLSN	GTGCACACGAGACTCATCGGTTGTTACGGCTTTTGTCCAACAGGGCAAAAACGT
GgI	RAKTCEAVNLTEELLRLT	GTGAGGCCGTTAATCTCACCGAGGAACTGCTTAGGTTGACCAATAACGTTATTTC
FS6	NNVISRMMLGEAEEARD	TAGGATGATGTTGGGGGAGGCAGAGGAGGCCAGAGATGTAGTGCGTGGTGTGA
	VVRGVSQIFGEFNVSDFI	GCCAGATATTCGGAGAGTTTAATGTCTCGGATTTCATTTGGCTGTTTAAGAAGAT
	WLFKKMDLQGFGKRIED	GGATTTGCAGGGGTTTGGGAAGAGGATAGAGGATTTGTTTCTGAGGTTCGATAC
	LFLRFDTLVERIVCKRVQ	GTTGGTGGAGAGGATTGTGTGCAAACGGGTGCAGATGAGGAAAGAGAAACGAA
	MRKEKRMGNGGSGKQQ	TGGGAAATGGAGGGAGTGGCAAGCAGCAGGGTACTGATGAGGTCAAAGATTTT
	GTDEVKDFLDLLLDCAE	CTTGATCTTTTGCTTGATTGCGCTGAGGATGAGAACTGCGAGGTGAAGATTCGAA
	DENCEVKIRKTQIKALIM	AGACTCAAATTAAGGCCTTGATTATGGATTTCTTCACTGCTGGGACAGATACCAT
	DFFTAGTDTMATSTEWA	GGCGACTTCAACAGAGTGGGCATTAGTGGAGCTTATCAAGAATCCCTTGTTACTA
	LVELIKNPLLLQKAREEID	CAAAAAGCTCGTGAAGAGATAGACAAAGCGTAG
	KA*	
	MEPQLVAVSVLVSALICY	ATGGAACCTCAACTCGTAGCAGTGTCTGTGTTGGTTTCAGCACTTATTTGCTACTT
	FFFRPYFHRYGKNLPPSPF	CTTTTTCCGGCCATATTTCCACCGTTACGGAAAAAACCTCCCACCGTCTCCTTTTT
	FRLPIIGHMHMLGPLLH	TCCGGCTTCCAATAATTGGCCACATGCACATGTTAGGTCCCCTTCTTCACCAATC
	QSFHNLSHRYGPLFSLNF	CTTCCACAACCTCTCTCACCGTTACGGTCCTCTGTTTTCACTTAACTTTGGCTCTGT
	GSVLCVVASTPHFAKQLL	TCTCTGTGTCGTTGCTTCCACCCCTCATTTTGCCAAACAACTCCTTCAAACCAACG
	QTNELAFNCRIESTAVKK	AACTCGCCTTTAACTGTCGCATTGAATCAACCGCCGTTAAAAAACTCACTTACGA
	LTYESSLAFAPYGDYWRF	GTCTTCCTTGGCCTTCGCACCTTACGGTGATTACTGGAGGTTCATTAAGAAGCTG
	IKKLSMNELLGSRSINNF	AGCATGAACGAGCTTTTGGGCTCTCGTAGCATAAACAACTTCCAACACCTGCGA
	QHLRAQETHQLLRLLSN	GCACAAGAGACTCATCAATTGTTAAGGCTTTTGTCCAACAGGGCAAGAGCGTTT
	RARAFEAVNITEELLKLT	GAGGCCGTGAATATCACCGAGGAGCTTCTTAAGTTGACCAACAACGTTATTTCTA
	NNVISIMMVGEAEEARD	TAATGATGGTTGGGGAGGCAGAGGAGGCAAGGGATGTGGTGCGTGATGTGACG
GgI	VVRDVTEIFGEFNVSDFI	GAGATCTTTGGAGAGTTTAATGTTTCGGATTTTATTTGGTTGTTTAAGAAGATAGA
FS7	WLFKKIDLQGFGKRIEDL	CTTGCAGGGGTTTGGGAAGAGGATTGAGGATCTGTTTCAGAGGTTTGATACGTTG
	FQRFDTLVERIISKREQTR	GTGGAAAGGATTATTAGCAAGCGGGAGCAGACGAGGAAAGACAGAAGGAGGA
	KDRRRSGKKGEQGSDDG	GTGGGAAGAAGGGTGAGCAGGGGAGTGATGATGGGATCAGAGACTTTCTTGAT
	IRDFLDILLDCTEDENSEI	ATCTTGCTTGACTGTACTGAGGATGAGAATTCCGAGATTAAAATCCAAAGGGTTC
	KIQRVHIKALIMVRFLHC	ACATTAAGGCCTTGATTATGGTAAGATTTCTTCACTGCAGGGACGGATACCACAG
	RDGYHSDFNRVGISGAR	CGATTTCAACAGAGTGGGCATTAGTGGAGCTCGTCAAGAAACCCTCCTTGCTAC
	QETLLATKSPEEIDNVVG	AAAAAGTCCGGAAGAGATAGACAATGTCGTAGGAAAAGACAGACTTGTTGAGG
	KDRLVEESDCPNLPYLQA	AATCTGATTGTCCCAATCTCCCATATCTCCAAGCCATTCTTAAAGAAACATTCCG
	ILKETFRLHPPVPMVTRR	TTTGCACCCACCGGTTCCTATGGTTACAAGAAGATGCGTGGCAGAGTGCAGGGT
	CVAECRVENYVIPEDSLL	AGAGAATTACGTCATCCCAGAAGACTCACTTCTCTTTGTGAATGTTTGGTCCATT
	FVNVWSIGRNPNFWDNP	GGGAGAAACCCAAACTTTTGGGACAACCCATTGGAGTTTCGCCCCGAACGATTC

	LEFRPERFLKLEGDSSGAI	TTAAAACTAGAAGGAGATTCCAGTGGAGCTATTGATGTGAGGGGAAGCCATTTT
	DVRGSHFQLLPFGSGEDV	CAGCTTCTGCCATTTGGGTCTGGGGAGGATGTGTCCTGGTGTGTCCTTGGCTATGC
	SWCVLGYARGASTNWC	AAGAGGTGCCAGCACTAATTGGTGCTATAATCCAGTGCTTTGA
	YNPVL*	
	MISDHQSILLSLSLLFFAFL	ATGATTTCTGATCATCAGTCCATTTTATTATCCCTCAGTCTGTTGTTCTTTGCTTTC
	LHILSLKRNNKPKPHFRE	CTTCTCCATATCCTCTCGTTGAAGAGAAACAACAAACCAAAGCCCCATTTTCGTG
	PPSPPALPIIGHLHLLKPL	AGCCACCAAGCCCACCAGCACTACCCATAATTGGCCATCTCCATCTCCTCAAAC
	VHQAFRDLSEQHGPLVL	CACTCGTCCATCAAGCCTTCCGCGACCTCTCCGAACAACACGGCCCTCTCGTGTT
	LRLGSVRFVVASTPSLAR	GCTTCGGCTCGGCTCGGTCCGGTTCGTCGTTGCCAGCACCCCATCACTCGCGAGA
	EFLKTHELAYSFRKTNTA	GAGTTTCTCAAGACACACGAGCTAGCATACTCTTTCCGCAAAACGAACACGGCC
	INTVIYDNATFAFSPHGD	ATCAACACGGTCATCTACGACAACGCCACTTTTGCTTTCTCCCCTCATGGGGACT
	YWKFIKKLSTTELLGNRTI	ACTGGAAGTTCATCAAGAAGCTGAGCACCACAGAACTCTTGGGCAACCGAACT
	GQFLPIRAREVHEFIWAL	ATTGGACAATTCCTACCCATTCGGGCCCGTGAGGTTCATGAATTCATTTGGGCCT
	ENKSKAQESVNLTQELL	TAGAGAACAAGTCTAAGGCCCAAGAGAGCGTGAACCTCACTCAGGAGCTCCTC
	KLSNNIISKMMLSIKSSGT	AAGCTTTCCAACAACATAATATCGAAGATGATGCTGAGCATCAAGAGCTCCGGG
	DGQAEQARALVREVTEIF	ACAGACGGCCAGGCGGAGCAGGCGAGGGCTTTGGTTCGCGAGGTGACGGAGAT
	GEFNVSDFIGIFKNLDLQ	CTTCGGGGAATTCAACGTCTCCGATTTCATAGGAATCTTCAAGAACTTGGACCTG
	GFKKRAMHIQKRYDALL	CAGGGTTTTAAAAAGAGAGCCATGCACATACAAAAGAGGTACGATGCTCTGTTG
GgI	EKIISDREESRRKEEGRCEI	GAGAAGATCATCTCCGACCGCGAGGAATCGAGAAGGAAGGAAGAGGGACGTTG
FS8	NGGEEKVRDFLDILLDFS	TGAGATTAACGGAGGAGAAGAGAAAGTGAGGGATTTTCTAGACATTTTGCTTGA
	EEKDCEVSLTRNHIKSVIL	TTTCTCTGAGGAGAAAGATTGTGAAGTCAGCTTAACTAGAAACCACATCAAATC
	DYFTAATDTTAISVEWAI	AGTCATATTGGATTACTTTACAGCAGCTACGGACACAACAGCCATCTCAGTGGA
	AELFNNPRVLKKAQEEV	ATGGGCAATAGCGGAACTGTTCAACAATCCAAGGGTGCTGAAGAAAGCGCAAG
	DRVIGNKRLVCEADSPN	AGGAGGTAGACAGAGTAATTGGGAACAAAAGACTAGTGTGTGAAGCAGACAGT
	LPYIHAIIKETMRLHPPIP	CCAAACCTTCCTTACATACATGCCATCATAAAAGAGACGATGAGGCTTCACCCA
	VIMRKGIEDCVVDGYMIP	CCGATACCGGTGATCATGAGGAAGGGGATAGAGGACTGCGTGGTTGATGGGTAC
	KGSVVGVNIWAMGRDP	ATGATACCCAAAGGCTCAGTAGTCGGTGTGAACATTTGGGCCATGGGAAGGGAC
	KVWESPLEFRPERFLEGE	CCAAAAGTGTGGGAAAGCCCATTGGAGTTCAGGCCAGAGAGGTTTCTAGAAGG
	KGREIDLKGHHFELLPFG	CGAAAAAGGGAGAGAGATAGATCTCAAAGGCCATCACTTTGAGTTGTTGCCGTT
	SGRRGCPGMNLAMRELP	TGGTTCTGGGAGGAGGGGTTGCCCTGGAATGAATTTGGCCATGCGGGAATTGCC
	AIIGALVQCFEWKMLDSE	CGCAATAATTGGAGCATTGGTACAGTGCTTTGAGTGGAAGATGCTTGATTCCGAA
	RKILDRGRKIDMDERPGL	CGTAAGATCTTAGACCGTGGTAGAAAAATTGACATGGATGAACGGCCAGGATTG
	TVPRATDLICIPVARLNN	ACTGTTCCTCGGGCCACTGATCTTATTTGTATTCCAGTCGCACGATTGAATAATCC
	PIPFLQV*	CATTCCTTTTCTTCAAGTGTAG
	MLVELAITLLVIALFIHLR	ATGTTGGTGGAACTTGCAATTACTCTGTTGGTGATAGCCCTGTTCATACACCTGC
	PTPSAKSKSLRHLPNPPSP	GTCCCACACCAAGTGCAAAATCAAAGTCCCTTCGCCACCTTCCAAACCCTCCAA
	KPRLPFVGHLHLLDKPLL	GTCCAAAACCCCGTCTCCCATTCGTGGGTCACCTTCACCTTTTAGACAAACCCCT
	HNSLIDLSKRYGPLYSLYF	TCTCCACAACTCCCTCATCGATCTAAGCAAACGCTATGGTCCCCTTTACTCCCTCT
GgI	GSMPTVVVSTPELFKLFL	ACTTCGGTTCCATGCCAACCGTTGTAGTCTCCACCCCTGAACTTTTCAAACTCTTC
FS9	QTHEASSFNTRFQTSAIR	CTCCAAACCCACGAGGCCTCTTCCTTCAACACCAGGTTCCAAACCTCTGCCATTA
	RLTYDNSVAMVPFGPYW	GGCGCCTAACCTACGACAACTCTGTTGCCATGGTTCCCTTTGGTCCTTACTGGAA
	KFIRKLIMNDLLNATTVN	GTTCATTAGGAAGCTTATCATGAACGATCTCCTCAATGCCACAACTGTGAACAA
	KLRPLRSQEIRKVLRVMA	GTTGAGGCCTTTAAGGAGCCAGGAAATCCGAAAGGTCCTTAGGGTGATGGCACT
	LSAESQVPLNVTEELLKW	GAGTGCAGAGTCTCAAGTTCCTCTTAATGTTACCGAGGAGCTTCTCAAGTGGACC

	TNSTISRMMLGEAEEIRDI	AACAGCACCATCTCGAGGATGATGCTTGGGGAAGCAGAGGAAATCAGGGACAT
	ARDVLKIFGEYSLTDFIWP	AGCACGTGACGTGCTTAAGATCTTTGGGGAGTATAGTCTCACCGACTTCATCTGG
	LKKLKVGQYEKRIDDIFN	CCCTTGAAGAAACTCAAGGTTGGGCAATACGAGAAGAGGATTGACGATATATTC
	RFDPVIERVIKKRQEIRKK	AACAGGTTTGACCCCGTCATTGAGAGGGTCATCAAGAAAAGACAGGAGATTAG
	KKERNGEVEEGEQSVVFL	GAAGAAGAAGAAGGAGAGGAATGGTGAGGTCGAGGAGGGTGAACAGAGTGTG
	DTLLDFAEDETMEIKITKE	GTTTTTCTCGACACTTTGCTCGATTTTGCTGAGGATGAGACCATGGAGATCAAAA
	QIKGLVVDFFSAGTDSTA	TCACCAAGGAACAAATCAAGGGTCTTGTTGTGGATTTCTTCTCAGCAGGGACGG
	VATEWALSELINNPRVL	ATTCCACGGCCGTGGCAACAGAATGGGCTCTGTCAGAGCTCATCAACAACCCCA
	QKAREEVDAVVGKDRLV	GGGTGCTTCAAAAGGCACGAGAGGAGGTCGATGCGGTTGTGGGAAAAGACAGA
	DEADVQNLPYIRSIVKET	CTCGTTGACGAGGCAGATGTCCAGAACCTTCCTTACATTAGATCCATCGTGAAGG
	FRMHPPLPLVKRKCVQE	AGACGTTCCGCATGCACCCACCACTACCCTTGGTCAAAAGAAAGTGCGTGCAGG
	CEIDGYAIPEGALILFNV	AGTGTGAGATCGACGGTTATGCGATCCCAGAGGGAGCATTGATCCTTTTCAATGT
	WAVGRDPKYWDRPTEFR	TTGGGCCGTCGGAAGAGACCCAAAATACTGGGACAGGCCCACTGAGTTCCGTCC
	PERFLENVGEGDQAVDL	CGAAAGGTTCTTAGAAAATGTGGGTGAAGGGGATCAAGCCGTTGACCTTAGGGG
	RGQHFQLLPFGSGRRMC	TCAACATTTCCAACTTCTTCCATTTGGGTCTGGAAGGAGGATGTGCCCTGGTGTG
	PGVNLATAGMATLLASV	AATTTGGCCACTGCGGGAATGGCCACACTGCTTGCGTCAGTTATCCAGTGCTTTG
	IQCFDLSVVGPQGKILKG	ATCTCAGCGTGGTGGGCCCACAGGGAAAGATATTGAAGGGCAATGATGCCAAG
	NDAKVSMEESAGLTVPR	GTTAGCATGGAAGAGAGTGCTGGACTCACGGTTCCAAGGGCACATAACCTCGTG
	AHNLVCVPVARSSAVPK	TGTGTCCCGGTTGCAAGATCAAGTGCCGTACCTAAACTCTTTTCCTCGTGA
	LFSS*	
	MLDIQGYLVLFLLWFISTI	ATGCTTGACATCCAAGGCTACCTCGTGCTATTCCTCTTATGGTTCATCTCAACCAT
	LIRSIFKKSQCYKLPPGPPI	TTTGATTAGATCCATCTTCAAAAAATCCCAGTGTTATAAACTACCACCAGGACCC
	SLPLIGHAPYLRSLLHQA	CCAATCTCTTTACCACTCATTGGACACGCTCCTTACCTTAGATCACTCCTCCACCA
	LYKLSTRYGPLMHVLIGS	AGCACTCTACAAACTCTCCACCCGATATGGGCCCTTGATGCACGTCCTCATCGGT
	QHVIVASSAEMAKQILKT	TCGCAACATGTCATCGTTGCGTCCTCTGCAGAAATGGCTAAGCAAATCCTAAAA
	YEESFCNRPIMIASENLTY	ACCTACGAGGAGTCGTTTTGCAACCGTCCGATCATGATCGCGAGCGAGAATTTG
	GAADYFFIPYGTYWRFLK	ACTTACGGCGCCGCCGATTACTTCTTCATTCCATACGGAACCTACTGGAGGTTCC
	KLCMTELLSGKTLEHFVN	TTAAGAAGCTATGCATGACAGAGCTTCTTAGTGGGAAGACGCTTGAGCACTTTGT
	IREDEIKCFLRNVLEISKT	CAATATCCGCGAGGATGAGATTAAGTGCTTCTTGAGGAATGTTCTGGAGATATCG
	GKGVEMRQELIRHTNNII	AAGACCGGGAAAGGGGTGGAGATGAGGCAGGAGTTGATAAGGCACACAAACA
	SRMTMGKKSNGTNDEV	ACATCATTTCTAGGATGACTATGGGGAAGAAGAGTAATGGGACAAATGATGAG
GiIF	GQVRKLVREIGELLGAFN	GTTGGTCAGGTGAGGAAGTTGGTTAGGGAGATTGGGGAACTTCTTGGTGCTTTTA
S1	LGDIIGFMRPFDLQGFGK	ACTTGGGTGACATTATTGGGTTCATGAGGCCTTTTGATCTGCAAGGGTTTGGGAA
	KNRDAHHNMDVMMEK	GAAGAATAGGGATGCACACCATAATATGGATGTGATGATGGAGAAGGTGCTCA
	VLKEHEEARAKEKGGAE	AGGAGCATGAAGAGGCCAGGGCAAAGGAGAAGGGTGGTGCTGAGAGTGATAG
	SDRKKDLFDILLNLIEAD	GAAGAAGGATCTATTTGACATTCTGTTGAACTTGATTGAAGCTGATGGTGCTGAC
	GADNKLTRESAKAFALD	AATAAACTCACTAGAGAAAGTGCCAAAGCCTTTGCACTGGACATGTTCATTGCT
	MFIAGTNGPASVLEWSL	GGGACAAACGGACCAGCAAGTGTGTTAGAATGGTCACTGGCAGAGCTGATTAG
	AELIRNPQVFKKAREEID	AAATCCACAAGTTTTCAAGAAGGCAAGAGAAGAGATTGACTCTGTTGTTGGAAA
	SVVGKERLVKESDIPNLP	GGAAAGGCTAGTCAAAGAATCAGACATACCAAACCTACCTTACCTCCAAGCAGT
	YLQAVVKETLRMHPPTPI	GGTCAAAGAAACCCTGAGGATGCACCCACCAACCCCAATCTTTGCAAGGGAAG
	FAREAIRGCQVDGYDIPA	CCATAAGGGGTTGCCAAGTTGATGGCTATGACATCCCAGCAAACTCAAAGATCT
	NSKIFINAWAIGRDPKY	TCATCAATGCATGGGCCATTGGAAGGGATCCAAAGTATTGGGACAACCCACAAG
	WDNPQVYSPERFLITDEP	TTTATAGCCCTGAAAGGTTCTTAATCACTGATGAACCTGGAAAGAGTAAAATTG

	GKSKIDVRGQYYQLLPFG<br>SGRRSCPGSSLALLVIQAT<br>LASLVQCFDWVVNDGK<br>NSEIDMSEEGRVTVFLAK<br>PLKCKPVPRFVPFSA*	ATGTTAGGGGGCAATACTACCAATTGTTGCCATTTGGGAGTGGGAGAAGAAGCT<br>GCCCTGGATCCTCACTTGCATTGCTTGTCATCCAAGCAACACTTGCAAGTTTGGT<br>ACAGTGCTTTGATTGGGTTGTTAATGATGGTAAGAATAGTGAGATTGACATGTCA<br>GAGGAAGGAAGGGTAACTGTGTTTTTGGCTAAGCCTCTCAAGTGCAAGCCTGTTC<br>CTCGTTTTGTTCCATTCTCTGCCTGA
GiIF<br>S2	MADFGDYFGLLLIVLFSIL<br>LFRVIFTNNKACLPPSPR<br>ALPVLGHLYLLTNLPHQ<br>AFQKISSRYGPLVYLLFGS<br>KHCVLVSSPEMAKQCLK<br>TNESCFLNRPKRTNVDYI<br>TYGSSDFVMAPYGPYWR<br>FMKRLCMNELLGGRILH<br>QHLPIRAEEIKLFLKGMM<br>KRSDFGERVNVGEELSLL<br>SNNIITRMALRRRCSDVE<br>GEGHQLIELVREMTELGG<br>KFNLGDMLWFVKRLDL<br>QGFGKRLESVRSRYDAIM<br>ERIMKEHEDARMVKKK<br>NKDSCNGDEAVRDLLDV<br>LLDIYADESSEIRLTRENIK<br>AFIMNMFGAGTETSACTI<br>EWALAELINHPDMMER<br>ARQEIDSVVGKSRLVEES<br>DIPNLPYVQSIVKETMRL<br>HPTGPLIVRQSTEDCNVD<br>GYHIPARTSVFVNVWAIG<br>RDPKYWESPLEFKPERFL<br>NEEGQSMLDLKGQNFEL<br>LSFGAGRRSCPGASLALQ<br>IIYTTLAGMVQCFEWKV<br>GEEGNNNGTIDMEEGPG<br>MALPRANPLICTPVARL<br>HPFGTL*	ATGGCTGATTTTGGTGACTATTTTGGGCTACTTCTCATTGTTCTATTCTCAATCCTC<br>TTGTTCCGAGTGATCTTCACCAACAATAAAGCATGTCTTCCACCAAGCCCTCGTG<br>CCTTACCCGTTCTGGGACACCTCTACCTTCTCACAAACCTCCCTCACCAAGCATT<br>CCAAAAAATCTCATCCCGTTATGGCCCTTTGGTCTACCTCTTGTTTGGTTCCAAAC<br>ACTGTGTTCTTGTCTCTTCCCCTGAAATGGCCAAACAATGCCTTAAAACCAATGA<br>GTCCTGTTTCCTAAACAGACCCAAGAGAACCAATGTGGACTACATCACATACGG<br>TTCCTCAGATTTTGTAATGGCACCCTATGGACCCTATTGGAGGTTCATGAAGAGG<br>CTTTGCATGAATGAACTCCTTGGTGGTCGAATACTTCACCAGCACCTTCCCATTA<br>GAGCTGAAGAGATTAAGCTTTTCTTGAAGGGTATGATGAAAAGGTCTGATTTTGG<br>AGAGAGGGTGAATGTTGGGGAGGAACTTTCTTTGCTTTCGAATAACATCATCACA<br>AGGATGGCTTTGAGAAGAAGGTGTAGTGATGTTGAAGGGGAAGGGCACCAATT<br>GATTGAGCTCGTGAGGGAGATGACTGAGCTAGGTGGGAAGTTCAACTTGGGAGA<br>CATGTTGTGGTTTGTTAAGAGGCTTGATTTGCAAGGGTTTGGTAAGAGGTTGGAG<br>AGTGTAAGGAGTAGGTATGATGCTATAATGGAGAGGATCATGAAGGAACATGA<br>AGATGCAAGGATGGTGAAGAAGAAAAATAAGGATAGTTGTAATGGAGATGAAG<br>CAGTGAGGGATTTACTTGATGTTTTACTTGATATCTATGCTGATGAAAGTTCAGA<br>GATTAGATTAACCAGAGAAAATATCAAGGCCTTCATCATGAACATGTTTGGTGCT<br>GGAACTGAGACATCAGCATGTACAATAGAATGGGCTTTGGCTGAGCTAATAAAC<br>CATCCAGACATGATGGAAAGAGCAAGACAAGAGATTGATTCAGTGGTTGGTAA<br>GAGCAGATTGGTGGAGGAATCAGATATTCCCAACCTTCCCTATGTTCAATCCATA<br>GTGAAAGAAACAATGAGGCTTCACCCAACTGGGCCTCTAATAGTGAGGCAATCA<br>ACTGAGGATTGCAATGTTGATGGGTATCACATTCCAGCAAGGACTTCTGTGTTTG<br>TTAATGTGTGGGCCATTGGTAGGGACCCAAAATACTGGGAAAGCCCACTTGAGT<br>TTAAGCCAGAGAGGTTCCTCAATGAGGAGGGACAGAGCATGTTGGATCTGAAGG<br>GGCAGAATTTTGAACTGTTGTCTTTTGGTGCTGGAAGGAGAAGTTGCCCTGGTGC<br>TTCACTAGCCTTGCAGATTATTTATACAACACTGGCTGGTATGGTTCAGTGTTTTG<br>AATGGAAGGTTGGTGAAGAAGGGAATAATAATGGAACCATTGACATGGAGGAG<br>GGTCCTGGAATGGCACTTCCTAGGGCAAATCCCCTGATATGCACCCCTGTGGCTA<br>GGCTCCATCCTTTTGGGACACTCTGA
GiIF<br>S3	MAEIQDFIQLFLIWLLSTI<br>AIRAILTRKQNNKEGHSQ<br>SHPPSPLALPIIGHLHLIS<br>QLPHQSFHNLSTRYGPIM<br>QIFLGSVPCVVASTPEIAK<br>EFLKTNEASFSNRFRSAA<br>VHYLSYGSKGFLFAPYGE<br>YWKFMKKLCVSELLGGR<br>TLDQLSPLRKQETVRFLR	ATGGCTGAAATCCAAGACTTTATCCAACTTTTCCTAATTTGGCTACTCTCCACCAT<br>TGCAATTCGAGCCATACTAACCCGAAAACAGAACAACAAGGAGGGTCATAGCC<br>AAAGCCACCCACCAAGCCCTCTAGCACTACCCATAATTGGACACCTTCACCTTA<br>TTTCTCAATTACCCCACCAAAGCTTCCACAACCTCTCAACTCGCTATGGACCCAT<br>CATGCAAATCTTCCTAGGCTCTGTCCCTTGTGTAGTAGCTTCCACCCCAGAAATC<br>GCCAAGGAGTTCCTCAAAACCAATGAAGCATCCTTCTCCAACCGTTTCAGAAGT<br>GCTGCGGTTCACTATTTATCGTATGGCTCAAAGGGGTTCTTGTTTGCGCCTTATGG<br>AGAGTACTGGAAGTTCATGAAGAAGCTCTGTGTATCTGAGCTTCTCGGTGGAAG<br>AACACTCGATCAGCTAAGTCCACTGAGGAAACAAGAGACTGTGAGGTTTCTGAG

	LMQSKGEAGVAIDVGGE	GCTTATGCAGAGTAAAGGAGAAGCTGGTGTTGCTATTGATGTTGGTGGGGAGCT
	LLTLANSVITRMTMSKTC	ATTGACACTTGCAAATAGTGTCATAACGAGGATGACTATGAGCAAAACGTGTTT
	FENDGDVEDIRKMVKDT	TGAAAATGATGGTGATGTGGAAGATATTAGGAAGATGGTGAAGGACACTGCGG
	AELAGKFNVSDFIWFCQ	AGCTTGCTGGGAAGTTTAATGTGTCTGACTTTATTTGGTTCTGTCAGAATTTGGAT
	NLDLQGMNKRLKEILDR	CTGCAGGGGATGAACAAGAGGCTTAAGGAGATTCTTGATAGGTTTGATACCATG
	FDTMMERVIREHEVERK	ATGGAGAGGGTGATAAGGGAGCATGAAGTGGAAAGGAAGAGAAGGAAGGAAA
	RRKERGEEGANQVMDLL	GGGGTGAAGAAGGAGCTAATCAAGTTATGGACTTGCTTGATATTTTGTTGGAAAT
	DILLEIQGDERTEMKLTR	ACAGGGTGATGAGAGAACTGAGATGAAACTGACCAGAGAGAATGTCAAGGCTT
	ENVKAFILDIFMAGTDTS	TCATCTTGGACATATTTATGGCAGGAACTGACACGTCAGCTATAACGATCGAAT
	AITIEWALAELINYPHVM	GGGCTCTTGCTGAGTTAATCAACTACCCGCATGTGATGGAGAAAGGAAGACAAG
	EKGRQEIDSVIGKNRLIEE	AAATCGATTCAGTAATAGGAAAGAATAGATTAATAGAAGAGTCAGATCTTCCCA
	SDLPNLPYLRAIVKETLRL	ACCTTCCTTACTTGCGAGCTATAGTTAAAGAAACATTAAGGCTTCACCCTGCAGC
	HPAAPLLGRESSESCNVC	ACCACTTCTAGGGAGAGAATCATCTGAAAGTTGCAATGTTTGCGGGTATGAAAT
	GYEIPAKTVVFVNLWSM	TCCAGCGAAGACAGTTGTATTTGTTAATTTGTGGTCAATGGGTAGGGACCCAAAC
	GRDPNMWENPLDFRPER	ATGTGGGAGAACCCACTTGATTTCAGGCCAGAGAGGTTTATAATGGGTGAAGAG
	FIMGEESVQLDVRGQNF	AGTGTTCAGTTAGATGTGAGGGGACAGAATTTCCAACTAATGCCATTTGGGACTG
	QLMPFGTGRRACPGASL	GAAGAAGGGCATGTCCTGGTGCCTCACTTGCACTTCAGATGGTTCCCACTAACCT
	ALQMVPTNLAAMVQCF	TGCTGCTATGGTTCAGTGCTTTGAATGGAAGGTTGTTGATGATGGTGGAGATGGT
	EWKVVDDGGDGNNGK	AATAATGGGAAAGTTAGCATGGAAGAGAAACCAGGCATGACCCTTCCAAGGGC
	VSMEEKPGMTLPRAHPL	TCATCCTTTGACGTGTGTCCCTGTTCCTCGTTTTGATTGCATTCCTTCTTGTGTATA
	TCVPVPRFDCIPSCV*	G
	MADYQSYTLLFILWLVSII	ATGGCTGATTACCAAAGCTACACCCTACTTTTTATCCTATGGCTAGTGTCCATAAT
	VVRTILTRKQNKAHVPPS	CGTGGTTCGAACCATTCTCACCAGAAAACAGAACAAGGCTCATGTACCACCAAG
	PLALPIIGHLHLLAPIPHQ	CCCACTAGCCTTACCCATAATTGGACACCTTCACCTTCTTGCACCAATACCCCAT
	ALHKLSTRYGPIMQLSLG	CAAGCTCTTCACAAGCTATCAACACGCTATGGACCCATAATGCAACTTTCCCTTG
	SVPCLVASTPESAKEFLKT	GTTCCGTCCCTTGCCTCGTGGCTTCAACACCAGAATCCGCCAAAGAGTTCCTTAA
	HETKFSNRPQSSAVHYLT	AACCCACGAGACCAAATTCTCCAACCGTCCTCAAAGCTCTGCTGTTCATTACCTA
	YGSQDFSFAPYGPYWKF	ACATATGGTTCCCAAGACTTCTCCTTTGCCCCATATGGACCTTATTGGAAATTCAT
	MKRICMSELLGGHTLTLL	GAAGAGAATATGCATGTCTGAACTTCTTGGGGGTCACACACTCACACTGCTTCTT
	LPVRKQETTRFLRLLLKK	CCCGTGAGAAAACAAGAGACGACAAGGTTTCTTAGACTCTTGCTAAAGAAAGG
	GNAGETVDVGGELLTLS	GAATGCAGGTGAGACTGTTGATGTTGGAGGGGAACTTCTGACACTGTCGAATAA
	NNIVSRMIMSQTCSEEDD	CATTGTTTCGAGGATGATTATGAGCCAAACTTGCTCAGAAGAAGATGATGGGGA
GiIF S4	GEGEEVVRKLVQDTVLL	AGGGGAAGAAGTGGTGAGGAAGTTGGTGCAAGACACGGTGCTTCTCACGGGGA
	TGKFNVSDFVWFFKNWD	AGTTTAATGTCTCGGACTTTGTTTGGTTCTTTAAGAATTGGGATTTGCAGGGGTTT
	LQGFGKRLREIRERFDTM	GGTAAAAGGCTTAGGGAGATTCGGGAGAGGTTTGATACCATGATGGAGAAGGT
	MEKVIKEHEEERRKRKEV	GATCAAGGAGCATGAAGAGGAGAGGAGGAAGAGAAAAGAAGTTGGTGGAGGA
	GGGDGRVKDLLDILLDIA	GATGGTCGAGTCAAGGATCTACTCGATATATTGTTGGATATAGCTGAAGATGAG
	EDESSDIKLTMENIKAFIL	AGCTCTGACATAAAATTGACAATGGAGAACATAAAGGCCTTCATCTTGGACATA
	DIFMAGTDTSALTTEWA	TTTATGGCAGGAACAGACACTTCAGCGCTAACCACTGAATGGGCTCTGGCAGAG
	LAELINHPNVMERARKEI	TTGATTAACCACCCAAATGTGATGGAGAGAGCAAGAAAAGAGATTGATGATGT
	DDVVGKTRIVEESDLVNL	AGTTGGGAAAACTAGAATAGTAGAAGAATCAGACCTTGTCAACCTTCCTTATCT
	PYLQAIVKETLRIHPTGPL	ACAAGCTATAGTCAAAGAAACACTGAGGATTCACCCCACAGGTCCTTTAATTGT
	IVRESSEKCTIWGYEIPAK	TAGAGAATCATCTGAAAAATGCACCATATGGGGCTATGAGATTCCAGCAAAGAC
	TQLFVNVWSIGRDPNHW	ACAATTGTTTGTTAATGTGTGGTCTATTGGCAGGGACCCCAATCACTGGGAGAAC

	ENPLEFKPERFIGEEGSGK	CCACTTGAGTTCAAACCAGAGAGATTTATTGGTGAGGAGGGAAGTGGGAAGAGT
	SQLDVRGQHFHLIPFGSG	CAGTTAGATGTGAGGGGACAACATTTTCACCTAATTCCATTTGGGAGTGGAAGA
	RRGCPGTSLALQVVQTN	AGAGGGTGCCCTGGAACCTCACTGGCTTTGCAGGTTGTGCAGACAAACCTTGCT
	LAAMIQCFEWKVNNNA	GCTATGATTCAGTGTTTTGAATGGAAGGTTAATAATAATGCAGGCAATAATTATG
	GNNYETVVNMEEKPGLT	AGACTGTAGTTAACATGGAAGAGAAACCTGGGTTAACACTTTCAAGGGCTTATC
	LSRAYPLICVPMPRLSPFP	CGTTAATATGTGTCCCAATGCCTAGGCTTAGTCCTTTTCCTTCAATGTGA
	SM*	
	MADFQDYLQIFLICVLSTI	ATGGCTGATTTTCAAGACTATCTTCAAATCTTCCTCATTTGTGTTCTATCCACCAT
	LVGSILWRKQNKHPHPP	CCTAGTTGGTTCCATACTATGGAGAAAACAAAACAAGCACCCTCATCCACCAGG
	GPLALPIIGHFHLLALLSP	TCCCTTAGCCCTACCCATCATTGGTCACTTCCACCTTTTGGCCCTTCTTTCCCCACT
	LLHSAFHKLSIRYGPIILLF	ACTTCACTCTGCTTTTCACAAACTCTCAATCCGCTATGGCCCCATAATACTCCTTT
	MGSVPCIVVSTAEAAKEF	TCATGGGCTCTGTCCCTTGTATTGTAGTTTCCACTGCAGAAGCTGCCAAAGAGTTT
	LKTHETSFSNRARTVAIET	CTCAAAACTCATGAAACCTCCTTTTCCAACCGTGCAAGAACTGTTGCCATTGAGA
	YSYGLQGIVFTPYGDYWR	CCTATAGCTATGGTCTTCAAGGCATAGTCTTTACACCCTATGGAGACTATTGGAG
	FIKKLCMSELLGQNILNR	GTTTATTAAGAAATTGTGCATGTCTGAACTTCTTGGTCAAAATATATTGAATCGAT
	FLAVRQQETERFIKLVFN	TCCTTGCTGTGAGGCAGCAAGAGACCGAGAGGTTCATCAAACTTGTGTTCAATA
	KGVEGEAVDFGEQFMKL	AAGGGGTGGAAGGTGAGGCTGTGGATTTTGGGGAACAATTCATGAAGCTTGCAA
	ANNIMSRMTISQTSSKND	ATAATATCATGTCAAGAATGACCATTAGTCAAACAAGTTCTAAGAATGATGATG
	DEANEMMKMVADITEIL	AAGCAAACGAGATGATGAAGATGGTGGCAGATATTACAGAGATCTTAGGGGAG
	GEFNISEFIWFLKRFNLQ	TTCAATATATCGGAATTCATTTGGTTCTTGAAGAGGTTCAATCTACAGGGACACA
GiIF	GHNKRLKEVHDKFDTRL	ACAAGAGGCTAAAGGAAGTTCATGACAAGTTTGATACCAGGCTGAACAGAGTG
	NRVIKEHEEERRKRKEM	ATAAAAGAGCATGAAGAGGAAAGAAGGAAGAGAAAGGAAATGGGTGGAAGTG
S5	GGSGTHQSKDILDVLLDI	GAACTCATCAATCTAAGGATATACTTGATGTGTTATTAGATATATATGAAGATAA
	YEDKSSEMKLTKENIKAF	AAGTTCAGAAATGAAATTGACTAAAGAGAACATAAAGGCCTTCATCTTGGACAT
	ILDIFLAGTDTSALALEW	ATTTCTTGCTGGGACTGACACGTCAGCATTGGCCCTAGAATGGGCTATAGCAGA
	AIAELINNPHVMEKARQ	ATTGATCAACAACCCACATGTGATGGAGAAAGCAAGGCAAGAGATAGATGCAG
	EIDAVVGKSRIVEESDVM	TGGTTGGAAAGAGTAGAATAGTGGAAGAATCTGATGTTATGAATCTTCCTTACTT
	NLPYLQAIVKETLRLHPV	ACAAGCCATAGTCAAAGAAACATTAAGGCTTCACCCTGTAGTTCCGATGATTTTC
	VPMIFRESSRRDIVGGYDI	AGGGAGTCATCTAGAAGGGATATAGTTGGTGGTTATGATATTCCAGCCAAGTCT
	PAKSKVFINIWSIGRDPN	AAAGTATTTATCAATATTTGGTCTATTGGTAGGGACCCCAATCACTGGGAGAACC
	HWENPLEFRPERFIGEDN	CACTTGAGTTTAGGCCAGAAAGGTTTATAGGTGAAGATAATGATCAATTGGATG
	DQLDVKGRHYHLIPFGS	TTAAGGGACGACATTATCATTTGATTCCATTTGGGAGTGGAAGAAGAGTGTGTCC
	GRRVCPGISLALQVLHA	TGGAATCTCTTTGGCATTGCAGGTTTTGCATGCGAACCTGGGTGCTGTGATTCAAT
	NLGAVIQCFELKVEGGN	GTTTCGAGTTGAAGGTTGAAGGTGGCAATGACTCGATTGACATGGCAGAGAAGC
	DSIDMAEKLGITMRRVHP	TTGGCATTACTATGCGCAGGGTTCATCCCATTATTTGTGTCCCTCTTCCAAGGCTT
	IICVPLPRLNPFPSM*	AATCCATTTCCTTCTATGTGA
	MESHLMAVSVVLVSALIC	ATGGAATCTCATCTCATGGCGGTGTCTGTAGTGTTGGTTTCAGCACTTATTTGCTA
	YFLFRPYFHRHGKNLPPS	CTTCCTTTTCCGGCCATATTTTCACCGCCACGGAAAAAACCTCCCACCATCTCCTC
	PLFRLPIIGHMHMLGPLL	TGTTTCGGCTTCCAATAATTGGGCACATGCACATGTTGGGTCCCCTTCTCCACCA
GiIF	HQSLHNLSHRYGPLFSLN	GTCCCTTCACAACCTCTCTCACCGTTACGGTCCTCTGTTTTCTCTTAACTTTGGCTC
S6	FGSVLCVVASSPHFAKQL	TGTTCTCTGTGTCGTTGCTTCCTCCCCTCACTTCGCCAAACAACTCCTTCAAACCA
	LQTNELAFSSRIETTAVKR	ACGAACTTGCATTTAGCAGTCGCATTGAAACCACCGCCGTTAAACGCCTCACTTA
	LTYESSLAFAPCGDYWRF	CGAATCCTCATTGGCCTTCGCACCCTGTGGGGATTATTGGAGGTTCATCAAGAAG
	IKKLSMNEFLGSRGMNN	CTTAGCATGAACGAGTTCTTGGGCTCACGTGGCATGAACAACTTCCTGCACGTGC

	FLHVRAHETHRLLRLLSN	GTGCACACGAGACTCATCGGTTGTTACGGCTTTTGTCTAACAGGGCAAAAACGT
	RAKTCEAVNLTEELLRLT	GTGAGGCCGTTAATCTCACCGAGGAACTGCTTAGGTTGACCAATAACGTTATTTC
	NNVISRMMLGEAEEARD	TAGGATGATGTTGGGGGAGGCAGAGGAGGCCAGAGATGTAGTGCGTGGTGTGA
	VVRGVSQIFGEFNVSDFI	GCCAGATATTCGGAGAGTTTAATGTCTCGGATTTCATTTGGTTGTTTAAGAAGAT
	WLFKKMDLQGFGKRIED	GGATTTGCAGGGGTTTGGGAAGAGGATAGAGGATTTGTTTCTGAGGTTCGATAC
	LFLRFDTLVERIVCKRVQ	GTTGGTGGAGAGGATTGTGTGCAAACGGGTGCAGATGAGGAAAGAGAAACGAA
	MRKEKRMGNGGSGKQQ	TGGGAAATGGAGGGAGTGGCAAGCAGCAGGGTACTGATGAGGTCAAAGATTTT
	GTDEVKDFLDLLLDCAE	CTTGATCTTTTGCTTGATTGCGCTGAGGATGAGAACTGCGAGGTGAAGATTCGAA
	DENCEVKIRKTQIKALIM	AGACTCAAATTAAGGCCTTGATTATGGATTTCTTCACTGCTGGGACAGATACCAT
	DFFTAGTDTMATSTEWA	GGCGACTTCAACAGAGTGGGCATTAGTGGAGCTTATCAAGAATCCCTTGTTACTA
	LVELIKNPLLLQKAREEID	CAAAAAGCTCGTGAAGAGATAGACAACGTAGTAGGGAATAATAGACTAGTTGA
	NVVGNNRLVEESDYPNL	GGAATCTGACTATCCCAACCTTCCTTATCTCCAAGCCATTATGAAGGAAACATTC
	PYLQAIMKETFRLHPPVP	CGTTTGCACCCACCGGTTCCTATGGTTACAAGAAGATGTGTGACAGAGTGCAGG
	MVTRRCVTECRIENYVIP	ATTGAGAATTACGTCATCCCAGAGAACACACTACTCTTTGTGAATGTTTGGTCCA
	ENTLLFVNVWSIGRNPN	TTGGAAGAAACCCAAACTATTGGGACAACCCATTGGTGTTTCGCCCCGAACGAT
	YWDNPLVFRPERFLKLEE	TCTTAAAGCTCGAAGAAGGAGATTCTAGTGGAGTCATTGATATAAGGGGGCAGC
	GDSSGVIDIRGQHFQLLP	ATTTTCAGCTTCTGCCATTTGGGTCTGGGAGGAGGATGTGCCCTGGTGTGTCCTTG
	FGSGRRMCPGVSLAMQE	GCCATGCAAGAGGTGCCATCACTTCTTGGTGCTATTATCCAGTGCTTTGATTTCCA
	VPSLLGAIIQCFDFQVVGS	GGTTGTGGGTTCCAAAGGTGAGATTTTGAAGGGTGATGACATAGTCAATATTGAC
	KGEILKGDDIVNIDVDER	GTGGATGAAAGGCCAGGATTGACAGCTCCAAGGGCCCACGAGTTGGTGTGTGTT
	PGLTAPRAHELVCVPVER	CCCGTTGAAAGGACCAGCTGTGGACCCCTTAAAATCCTTGGATGTTGA
	TSCGPLKILGC*	
	MEPQLVAVSVLVSALICY	ATGGAACCTCAACTCGTAGCAGTGTCTGTGTTGGTTTCAGCACTTATTTGCTACTT
	FFFRPYFHRYGKNLPPSPF	CTTTTTCCGGCCATATTTCCACCGTTACGGAAAAAACCTCCCACCGTCTCCTTTTT
	FRLPIIGHMHMLGPLLH	TCCGGCTTCCAATAATTGGCCACATGCACATGTTAGGTCCCCTTCTTCACCAATC
	QSFHNLSHRYGPLFSLNF	CTTCCACAACCTCTCTCACCGTTACGGTCCTCTGTTTTCACTTAACTTTGGCTCTGT
	GSVLCVVASTPHFAKQLL	TCTCTGTGTCGTTGCTTCCACCCCTCATTTTGCCAAACAACTCCTTCAAACCAACG
	QTNELAFNCRIESTAVKK	AACTCGCCTTTAACTGTCGCATTGAATCAACCGCCGTTAAAAAACTCACTTACGA
	LTYESSLAFAPYGDYWRF	GTCTTCCTTGGCCTTCGCACCTTACGGTGATTACTGGAGGTTCATTAAGAAGCTG
	IKKLSMNELLGSRSINNF	AGCATGAACGAGCTTTTGGGCTCTCGTAGCATAAACAACTTCCAACACCTGCGA
	QHLRAQETHQLLRLLSN	GCACAAGAGACTCATCAATTGTTAAGGCTTTTGTCCAACAGGGCAAGAGCGTTT
	RARAFEAVNITEELLKLT	GAGGCCGTGAATATCACCGAGGAGCTTCTTAAGTTGACCAACAACGTTATTTCTA
GiIF	NNVISIMMVGEAEEARD	TAATGATGGTTGGGGAGGCAGAGGAGGCAAGGGATGTGGTGCGTGATGTGACG
S7	VVRDVTEIFGEFNVSDFI	GAGATCTTTGGAGAGTTTAATGTTTCGGATTTTATTTGGTTGTTTAAGAAGATAGA
	WLFKKIDLQGFGKRIEDL	CTTGCAGGGGTTTGGGAAGAGGATTGAGGATCTGTTTCAGAGGTTTGATACGTTG
	FQRFDTLVERIISKREQTR	GTGGAAAGGATTATTAGCAAGCGGGAGCAGACGAGGAAAGACAGAAGGAGGA
	KDRRRSGKKGEQGSDDG	GTGGGAAGAAGGGTGAGCAGGGGAGTGATGATGGGATCAGAGACTTTCTTGAT
	IRDFLDILLDCTEDENSEI	ATCTTGCTTGACTGTACTGAGGATGAGAATTCCGAGATTAAAATCCAAAGGGTTC
	KIQRVHIKALIMDFFTAG	ACATTAAGGCCTTGATTATGGATTTCTTCACTGCAGGGACGGATACCACAGCGAT
	TDTTAISTEWALVELVKK	TTCAACAGAGTGGGCATTAGTGGAGCTCGTCAAGAAACCCTCCTTGCTACAAAA
	PSLLQKVREEIDNVVGKD	AGTTCGTGAAGAGATAGACAATGTCGTAGGAAAAGACAGACTTGTTGAGGAAT
	RLVEESDCPNLPYLQAIL	CTGATTGTCCCAATCTCCCATATCTCCAAGCCATTCTTAAAGAAACATTCCGTTTG
	KETFRLHPPVPMVTRRCV	CACCCACCGGTTCCTATGGTTACAAGAAGATGCGTGGCAGAGTGCAGGGTAGAG
	AECRVENYVIPEDSLLFV	AATTACGTCATCCCAGAAGACTCACTTCTCTTTGTGAATGTTTGGTCCATTGGGA

	NVWSIGRNPNFWDNPLE	GAAACCCAAACTTTTGGGACAACCCATTGGAGTTTCGCCCCGAACGATTCTTAA
	FRPERFLKLEGDSSGAIDV	AACTAGAAGGAGATTCCAGTGGAGCTATTGATGTGAGGGGAAGCCATTTTCAGC
	RGSHFQLLPFGSGRRMCP	TTCTGCCATTTGGGTCTGGAAGGAGGATGTGTCCTGGTGTGTCCTTGGCTATGCA
	GVSLAMQEVPALIGAIIQ	AGAGGTGCCAGCACTAATTGGTGCTATAATCCAGTGCTTTGATTTCCACGTTGTG
	CFDFHVVGPKGEILKGED	GGTCCCAAAGGTGAGATTTTGAAGGGTGAGGATATAGTCATTAATGTGGATGAA
	IVINVDERPGLTAPRAHN	AGGCCAGGATTGACGGCTCCAAGGGCTCATAACCTTGTGTGTGTTCCCGTTGAAA
	LVCVPVERTSGGGPLKIIG	GGACAAGTGGCGGTGGACCCCTCAAAATCATTGGATGTTGA
	C*	
	MISDHQSILLSLSLLFFAFL	ATGATTTCTGATCATCAGTCCATTTTATTATCCCTCAGTCTGTTGTTCTTTGCTTTC
	LRILSLKRNNKPKPHFRE	CTTCTCCGTATCCTCTCGTTGAAGAGAAACAACAAACCAAAGCCCCATTTTCGTG
	PPSPPALPIIGHLHLLKPL	AGCCACCAAGCCCACCAGCACTACCCATAATTGGCCATCTCCATCTCCTCAAAC
	VHQAFCDLSEQHGPLVL	CACTCGTCCATCAAGCCTTCTGCGACCTCTCCGAACAACACGGCCCTCTCGTGTT
	LRLGSVRFVVASTPSLAR	GCTTCGGCTCGGCTCGGTCCGGTTCGTCGTTGCCAGCACCCCATCACTCGCAAGA
	EFLKTHELAYSFRKTSTAI	GAGTTTCTCAAGACACACGAGCTAGCATACTCTTTCCGCAAAACGAGCACGGCC
	NTVIYDNATFAFSPHGDY	ATCAACACGGTCATCTACGACAACGCCACTTTTGCTTTCTCCCCTCATGGGGACT
	WKFIKKLSTTELLGNRTI	ACTGGAAGTTCATCAAGAAGCTCAGCACCACAGAACTCTTGGGCAACCGAACTA
	GQFLPIRAREVHEFIWAL	TTGGACAATTCCTACCCATTCGGGCCCGTGAGGTTCATGAATTCATTTGGGCCTT
	ENKSKAQESVNLTQELL	AGAGAACAAGTCTAAGGCCCAAGAGAGCGTGAACCTCACTCAGGAGCTCCTGA
	KLSNNIISKMMLSIKSSGT	AGCTTTCCAACAACATAATATCGAAGATGATGCTGAGCATCAAGAGCTCCGGGA
	DGQAEQARALVREVTEIF	CAGACGGCCAGGCGGAGCAGGCGAGGGCTTTGGTTCGCGAGGTGACGGAGATC
	GEFNVSDFIGIFKNLDLQ	TTTGGGGAATTCAACGTCTCCGATTTCATAGGAATCTTCAAGAACTTGGACCTGC
	GFKKRAMHIQKRYDALL	AGGGTTTTAAAAAGAGAGCCATGCACATACAAAAGAGGTACGATGCTCTGTTGG
GiIF	EKIISDREESRRKEEGRCEI	AGAAGATCATCTCCGACCGCGAGGAATCGAGAAGGAAGGAAGAGGGACGTTGT
S8	NGGEEKVRDFLDILLDFS	GAGATTAACGGAGGAGAAGAGAAAGTGAGGGATTTTCTAGACATTTTGCTTGAT
	EEKDCEVSLTRNHIKSVIL	TTCTCTGAGGAGAAAGATTGTGAAGTCAGCTTAACTAGAAACCACATCAAATCA
	DYFTAATDTTAISVEWAI	GTCATATTGGATTACTTTACAGCAGCTACGGACACAACAGCCATCTCAGTGGAA
	AELFNNPRVLKKAQEEV	TGGGCAATAGCGGAACTGTTCAACAATCCAAGGGTGCTGAAGAAAGCGCAAGA
	DRVIGNKRLVCEADSPN	GGAGGTAGACAGAGTAATTGGGAACAAAAGACTAGTGTGTGAAGCAGACAGTC
	LPYIHAIIKETMRLHPPIP	CAAACCTTCCTTACATACATGCCATCATAAAAGAGACGATGAGGCTTCACCCAC
	VIMRKGIEDCVVDGYMIP	CGATACCGGTGATCATGAGGAAGGGGATAGAGGACTGCGTGGTTGATGGGTAC
	KGSVVGVNIWAMGRDP	ATGATACCAAAAGGCTCAGTAGTCGGTGTGAACATTTGGGCCATGGAAGGGAC
	KVWESPLEFRPERFLEGE	CCAAAAGTGTGGGAAAGCCCATTGGAGTTCAGGCCAGAGAGGTTTCTAGAAGG
	KGREIDLKGHHFELLPFG	CGAAAAAGGGAGAGAGATAGATCTCAAAGGCCATCACTTTGAGTTGTTGCCGTT
	SGRRGCPGMNLAMRELP	TGGTTCTGGGAGGAGGGGTTGCCCTGGAATGAATTTGGCCATGCGGGAATTGCC
	AIIGALVQCFEWKMLDSE	CGCAATAATTGGAGCATTGGTACAGTGCTTTGAGTGGAAGATGCTTGATTCCGAA
	RTILDRGRKIDMDERPGL	CGTACGATCTTAGACCGTGGTAGAAAAATTGACATGGATGAACGGCCAGGATTG
	TVPRATDLICIPVARLNN	ACTGTTCCTCGGGCCACTGATCTTATTTGTATTCCAGTCGCACGATTGAATAATCC
	PIPFLQV*	CATTCCTTTTCTTCAAGTGTAG
	MLVELAITLLVIALFIHLR	ATGTTGGTGGAACTTGCAATTACTCTGTTGGTGATAGCCCTGTTCATACACCTGC
	PTPSAKSKSLRHLPNPPSP	GTCCCACACCAAGTGCAAAATCAAAGTCCCTTCGCCACCTTCCAAACCCTCCAA
GiIF	KPRLPFVGHLHLLDKPLL	GTCCAAAACCCCGTCTCCCATTCGTGGGTCACCTTCACCTTTTAGACAAACCCCT
S9	HNSLIDLSKRYGPLYSLYF	TCTCCACAACTCCCTCATCGATCTAAGCAAACGCTATGGTCCCCTTTACTCCCTCT
	GSMPTVVVSTPELFKLFL	ACTTCGGTTCCATGCCAACCGTTGTAGTCTCCACCCCTGAACTTTTCAAACTCTTC
	QTHEASSFNTRFQTSAIR	CTCCAAACCCACGAGGCCTCTTCCTTCAACACCAGGTTCCAAACCTCTGCCATTA

	RLTYDNSVAMVPFGPYW	GGCGCCTAACCTACGACAACTCTGTTGCCATGGTTCCCTTTGGTCCTTACTGGAA
	KFIRKLIMNDLLNATTVN	GTTCATTAGGAAGCTTATCATGAACGACCTCCTCAATGCCACAACTGTGAACAA
	KLRPLRSQEIRKVLRVMA	GTTGAGGCCTTTAAGGAGCCAGGAAATCCGAAAGGTCCTTAGGGTGATGGCACT
	LSAESQVPLNVTEELLKW	GAGTGCAGAGTCTCAAGTTCCTCTTAATGTTACCGAGGAGCTTCTCAAGTGGACC
	TNSTISRMMLGEAEEIRDI	AACAGCACCATCTCGAGGATGATGCTTGGGGAAGCAGAGGAAATCAGGGACAT
	ARDVLKIFGEYSLTDFIWP	AGCACGTGACGTGCTTAAGATCTTTGGGGAGTATAGTCTCACCGACTTCATCTGG
	LKKLKVGQYEKRIDDIFN	CCCTTGAAGAAACTCAAGGTTGGGCAATACGAGAAGAGGATTGACGATATATTC
	RFDPVIERVIKKRQEIRKK	AACAGGTTTGACCCCGTCATTGAGAGGGTCATCAAGAAAAGACAGGAGATTAG
	RKERNGEVEEGEQSVVFL	GAAGAAGAGGAAGGAGAGGAATGGTGAGGTCGAGGAGGGTGAACAGAGTGTG
	DTLLDFAEDETMEIKITKE	GTTTTTCTCGACACTTTGCTCGATTTTGCTGAGGATGAGACCATGGAGATCAAAA
	QIKGLVVDFFSAGTDSTA	TCACCAAGGAACAAATCAAGGGTCTTGTTGTGGATTTCTTCTCAGCAGGGACGG
	VATEWALSELINNPRVL	ATTCCACGGCCGTGGCAACAGAATGGGCTCTGTCAGAGCTCATCAACAACCCCA
	QKAREEVDAVVGKDRLV	GGGTGCTTCAAAAGGCACGAGAGGAGGTCGATGCGGTTGTGGGAAAAGACAGA
	DEADVQNLPYIRSIVKET	CTCGTTGACGAGGCAGATGTCCAGAACCTTCCTTACATTAGATCCATCGTGAAGG
	FRMHPPLPVVKRKCVQE	AGACGTTCCGCATGCACCCACCACTACCCGTGGTCAAAAGAAAGTGCGTGCAGG
	CEIDGYAIPEGALILFNV	AGTGTGAGATCGACGGTTATGCGATCCCAGAGGGAGCATTGATCCTTTTCAATGT
	WAVGRDPKYWDRPTEFR	TTGGGCCGTCGGAAGAGACCCAAAATACTGGGACAGGCCCACTGAGTTCCGTCC
	PERFLENVGEGDQAVDL	CGAAAGGTTCTTAGAAAATGTGGGTGAAGGGGATCAAGCCGTTGACCTTAGGGG
	RGQHFQLLPFGSGRRMC	TCAACATTTCCAACTTCTTCCATTTGGGTCTGGAAGGAGGATGTGCCCTGGTGTG
	PGVNLATAGMATLLASV	AATTTGGCCACTGCGGGAATGGCCACACTGCTTGCGTCAGTTATCCAGTGCTTTG
	IQCFDLSVVGPQGKILKG	ATCTCAGCGTGGTGGGCCCACAGGGAAAGATATTGAAGGGCAATGATGCCAAG
	NDAKVSMEESAGLTVPR	GTTAGCATGGAAGAGAGTGCTGGACTCACGGTTCCAAGGGCACATAACCTCGTG
	AHNLVCVPVARSSAVPK	TGTGTCCCGGTTGCAAGATCAAGTGCCGTACCTAAACTCTTTTCCTCGTGA
	LFSS*	
	MLDIQGYLVLFLLWFISTI	ATGCTTGACATCCAAGGCTACCTCGTGCTATTCCTCTTATGGTTCATCTCAACCAT
	LIRSIFKKSQCYKLPPGPPI	TTTGATTAGATCCATCTTCAAAAAATCCCAGTGTTATAAACTACCACCAGGACCC
	SLPLIGHAPYLRSLLHQA	CCAATCTCTTTACCACTCATTGGACACGCTCCTTACCTTAGATCACTCCTCCACCA
	LYKLSTRYGPLMHVLIGS	AGCACTCTACAAACTCTCCACCCGATATGGGCCCTTGATGCACGTCCTCATCGGT
	QHVIVASSAEMAKQILKT	TCGCAACATGTCATCGTTGCGTCCTCTGCAGAAATGGCTAAGCAAATCCTAAAA
	YEESFCNRPIMIASENLTY	ACCTACGAGGAGTCGTTTTGCAACCGTCCGATCATGATCGCGAGCGAGAATTTG
	GAADYFFIPYGTYWRFLK	ACTTACGGCGCCGCCGATTACTTCTTCATTCCATACGGAACCTACTGGAGGTTCC
	KLCMTELLSGKTLEHFVN	TTAAGAAGCTATGCATGACAGAGCTTCTTAGTGGGAAGACGCTTGAGCACTTTGT
	IREDEIKCFLRNVLEISKT	CAATATCCGCGAGGATGAGATTAAGTGCTTCTTGAGGAATGTTCTGGAGATATCG
GuI	GKGVEMRQELIRHTNNII	AAGACCGGGAAAGGGGTGGAGATGAGGCAGGAGTTGATAAGGCACACAAACA
FS1	SRMTMGKKSNGTNDEV	ACATCATTTCTAGGATGACTATGGGGAAGAAGAGTAATGGGACAAATGATGAG
	GQVRKLVREIGELLGAFN	GTTGGTCAGGTGAGGAAGTTGGTTAGGGAGATTGGGGAACTTCTTGGTGCTTTTA
	LGDIIGFMRPFDLQGFGK	ACTTGGGTGACATTATTGGGTTCATGAGGCCTTTTGATCTGCAAGGGTTTGGGAA
	KNRDAHHNMDVMMEK	GAAGAATAGGGATGCACACCATAATATGGATGTGATGATGGAGAAGGTGCTCA
	VLKEHEEARAKEKGGAE	AGGAGCATGAAGAGGCCAGGGCAAAGGAGAAGGGTGGTGCTGAGAGTGATAG
	SDRKKDLFDILLNLIEAD	GAAGAAGGATCTATTTGACATTCTGTTGAACTTGATTGAAGCTGATGGTGCTGAC
	GADNKLTRESAKAFALD	AATAAACTCACTAGAGAAAGTGCCAAAGCCTTTGCACTGGACATGTTCATTGCT
	MFIAGTNGPASVLEWSL	GGGACAAACGGACCAGCAAGTGTGTTAGAATGGTCACTGGCAGAGCTGATTAG
	AELIRNPQVFKKAREEID	AAATCCACAAGTTTTCAAGAAGGCAAGAGAAGAGATTGACTCTGTTGTTGGAAA
	SVVGKERLVKESDIPNLP	GGAAAGGCTAGTCAAAGAATCAGACATACCAAACCTACCTTACCTCCAAGCAGT

	YLQAVVKETLRMHPPTPI	GGTCAAAGAAACCCTGAGGATGCACCCACCAACCCCAATCTTTGCAAGGGAAG
	FAREAIRGCQVDGYDIPA	CCATAAGGGGTTGCCAAGTTGATGGCTATGACATCCCAGCAAACTCAAAGATCT
	NSKIFINAWAIGRDPKY	TCATCAATGCATGGGCCATTGGAAGGGATCCAAAGTATTGGGACAACCCACAAG
	WDNPQVYSPERFLITDEP	TTTATAGCCCTGAAAGGTTCTTAATCACTGATGAACCTGGAAAGAGTAAAATTG
	GKSKIDVRGQYYQLLPFG	ATGTTAGGGGGCAATACTACCAATTGTTGCCATTTGGGAGTGGGAGAAGAAGCT
	SGRRSCPGSSLALLVIQAT	GCCCTGGATCCTCACTTGCATTGCTTGTCATCCAAGCAACACTTGCAAGTTTGGT
	LASLVQCFDWVVNDGK	ACAGTGCTTTGATTGGGTTGTTAATGATGGTAAGAATAGTGAGATTGACATGTCA
	NSEIDMSEEGRVTVFLAK	GAGGAAGGAAGGGTAACTGTGTTTTTGGCTAAGCCTCTCAAGTGCAAGCCTGTTC
	PLKCKPVPRFVPFSA*	CTCGTTTTGTTCCATTCTCTGCCTGA
	MADFGDYFGLLLIVLFSIL	ATGGCTGATTTTGGTGACTATTTTGGGCTACTTCTCATTGTTCTATTCTCAATCCTC
	LFRVIFTNNKACLPPSPR	TTGTTCCGAGTGATCTTCACCAACAATAAAGCATGTCTTCCACCAAGCCCTCGTG
	ALPVLGHLYLLTNLPHQ	CCTTACCCGTTCTGGGACACCTCTACCTTCTCACAAACCTCCCTCACCAAGCATT
	AFQKISSRYGPLVYLLFGS	CCAAAAAATCTCATCCCGTTATGGCCCTTTGGTCTACCTCTTGTTTGGTTCCAAAC
	KHCVLVSSPEMAKQCLK	ACTGTGTTCTTGTCTCTTCCCCTGAAATGGCCAAACAATGCCTTAAAACCAATGA
	TNESCFLNRPKRTNVDYI	GTCCTGTTTCCTAAACAGACCCAAGAGAACCAACGTGGACTACATCACATACGG
	TYGSSDFVMAPYGPYWR	TTCCTCAGATTTTGTAATGGCACCCTATGGACCCTATTGGAGGTTCATGAAGAGG
	FMKRLCMNELLGGRILH	CTTTGCATGAATGAACTCCTTGGTGGTCGAATACTTCACCAGCACCTTCCCATTA
	QHLPIRAEEIKLFLKGMM	GAGCTGAAGAGATTAAGCTTTTCTTGAAGGGTATGATGAAAAGGTCTGATTTTGG
	KRSDFGERVNVGEELSLL	AGAGAGGGTGAATGTTGGGGAGGAACTTTCTTTGCTTTCGAATAACATCATCACA
	SNNIITRMALRRRCSDVE	AGGATGGCTTTGAGAAGAAGGTGTAGTGATGTTGAAGGGGAAGGGCACCAATT
	GEGHQLIELVREMTELGG	GATTGAGCTTGTGAGGGAGATGACTGAGCTAGGTGGGAAGTTCAACTTGGGAGA
	KFNLGDMLWFVKRLDL	CATGTTGTGGTTTGTTAAGAGGCTTGATTTGCAAGGGTTTGGTAAGAGGTTGGAG
	QGFGKRLESVRSRYDAIM	AGTGTTAGGAGTAGGTATGATGCTATAATGGAGAGGATCATGAAGGAACATGA
GuI	ERIMKEHEDARMVKKK	AGATGCAAGGATGGTGAAGAAGAAAAATAAGGATAGTTGTAATGGAGATGAAG
FS2	NKDSCNGDEAVRDLLDV	CAGTGAGGGATTTACTTGATGTTTTACTTGATATCTATGCTGATGAAAGTTCAGA
	LLDIYADESSEIRLTRENIK	GATTAGATTAACCAGAGAAAATATCAAGGCCTTCATCATGAACATGTTTGGTGCT
	AFIMNMFGAGTETSACTI	GGAACTGAGACATCAGCATGTACAATAGAATGGGCTTTGGCTGAGCTAATAAAC
	EWALAELINHPDTMERA	CATCCAGACACGATGGAAAGAGCAAGACAAGAGATTGATTCAGTGGTTGGTAA
	RQEIDSVVGKSRLVEESDI	GAGCAGATTGGTGGAGGAATCAGATATTCCCAACCTTCCCTATGTTCAATCCATA
	PNLPYVQSIVKETMRLHP	GTGAAAGAAACAATGAGGCTTCACCCAACTGGGCCTCTAATAGTGAGGCAATCA
	TGPLIVRQSTEDCNVDGY	ACTGAGGATTGCAATGTTGATGGGTATCACATTCCAGCAAGGACTTCTGTGTTTG
	HIPARTSVFVNVWAIGRD	TTAATGTGTGGGCCATTGGTAGGGACCCAAAATACTGGGAAAGCCCACTTGAGT
	PKYWESPLEFKPERFLNE	TTAAGCCAGAGAGGTTCCTCAATGAGGAGGGACAGAGCATGTTGGATCTGAAGG
	EGQSMLDLKGQNFELLSF	GGCAGAATTTTGAACTGTTGTCTTTTGGTGCTGGAAGGAGAAGTTGCCCTGGTGC
	GAGRRSCPGASLALQIIY	TTCACTAGCCTTGCAGATTATTTATACAACACTGGCTGGTATGGTTCAGTGTTTTG
	TTLAGMVQCFEWKVGEE	AATGGAAGGTTGGTGAAGAAGGGAATAATAATGGAACCATTGACATGGAGGAG
	GNNNGTIDMEEGPGMA	GGTCCTGGAATGGCACTTCCTAGGGCAAATCCCCTGATATGCACCCCTGTGGCTA
	LPRANPLICTPVARLHPF	GGCTCCATCCTTTTGGGACACTCTGA
	GTL*	
	MDSIQEYTPILLILLASTIL	ATGGACAGTATTCAAGAATACACACCAATTTTACTTATTTTGCTAGCCTCCACAA
GuI	LQAIFTRARSKFHLPPGPL	TATTGCTCCAAGCCATATTCACAAGAGCCCGGTCAAAGTTTCACCTCCCACCAGG
FS3	ALPVIGHFHLLLQPPLHR	TCCATTGGCCTTACCAGTTATTGGACACTTCCACTTGCTACTACAACCACCCCTTC
	AIHKISNRYGPLIHLHLGS	ACAGAGCCATTCACAAGATCTCAAACCGTTATGGACCCTTGATTCACCTACACCT
	TPVVFVSSAEIAKEIFRTH	TGGTTCCACTCCAGTGGTGTTTGTTTCCTCAGCTGAGATAGCCAAAGAGATCTTC

EASFCNRPSNVAISYLTY AGAACCCATGAGGCTTCTTTCTGCAACCGACCCTCTAATGTTGCCATTAGCTATC
KASDLGFAPYGTYWKFM TCACTTACAAAGCATCTGATTTGGGTTTCGCTCCCTATGGAACCTACTGGAAGTT
KKLCMSELLNGRMLDQL CATGAAGAAACTCTGCATGTCAGAGCTTCTCAATGGAAGGATGCTGGATCAACT
LPIRQEEINRFVQVIKKKA CCTTCCCATAAGGCAAGAGGAGATAAACAGGTTTGTGCAGGTGATTAAAAAGA
EAHGAVNVTDELLKLTN AAGCTGAAGCACATGGGGCTGTCAATGTTACTGATGAGCTATTGAAGCTAACAA
SVVMRMAISKSCFNRDD ACAGTGTGGTAATGAGAATGGCAATAAGCAAGAGCTGTTTCAACAGGGATGATG
EAHKVTERVRESSMLSG AGGCTCACAAGGTGACAGAGAGGGTGAGAGAGTCTTCCATGTTGAGTGGAATGT
MFNLADYFWFCKKLDLQ TTAACCTTGCCGATTACTTTTGGTTTTGCAAAAAGTTGGATCTTCAGGGAATGGG
GMGKRLKEVHDRLDTM GAAGAGGCTAAAGGAGGTTCATGACAGGCTTGACACCATGATGGAGAGTATCA
MESIIQEHEEARRGESTR TTCAGGAGCATGAAGAGGCAAGAAGAGGTGAATCGACCAGAAATAAGGATGCT
NKDAAKDVLDALLSIYE GCAAAGGACGTGCTTGATGCTCTTTTGAGTATTTATGAAGATCAAAGCTCAGAGG
DQSSEVKITRDNIKAFLVE TCAAAATAACCAGAGACAACATTAAAGCCTTCTTGGTGGAATCCGGGTACGATA
SGYDMFTGGTDTTAVTL TGTTCACAGGTGGGACAGATACAACTGCTGTGACTCTAGAATGGTCATTGGCAG
EWSLAELINHPTVMEKA AACTAATTAACCACCCAACAGTGATGGAGAAAGCAAGGAAGGAAATTGACTCT
RKEIDSVIGKDRTVVESDI GTGATTGGCAAGGACAGAACAGTAGTGGAATCAGACATAGCTGATCTTCCTTAT
ADLPYLQAIVKETLRLHP CTCCAAGCCATAGTGAAGGAGACACTAAGGCTTCACCCTCCATCTCCATTTATAT
PSPFILRASTEDCTVAGFD TGAGAGCATCAACTGAGGACTGCACCGTTGCTGGGTTTGATATCCCATCAAAAA
IPSKTQVFTNVWAIGRDP CTCAGGTCTTCACTAATGTGTGGGCCATTGGAAGGGATCCAAAGCATTGGGATA
KHWDNPLEFRPERFLRRE ACCCTCTTGAGTTTAGGCCAGAAAGGTTTCTCAGAAGGGAGAATCAAGTTGAAT
NQVELRGQHYQLLPFGS TAAGAGGGCAACATTATCAGCTTTTGCCTTTTGGGAGTGGAAGAAGAAGGTGCC
GRRRCPGTSLALNVAHT CTGGAACTTCACTAGCACTAAATGTTGCCCACACCACCCTTGCTGCTATGATTCA
TLAAMIQCFEWKGDQD ATGCTTTGAATGGAAGGGTGATCAAGATGGAGGAGGGGATGGTAGTGTTGACAT
GGGDGSVDMKEGPSFILS GAAAGAGGGTCCTTCTTTCATTCTTTCAAGAGCTCAGCCTCTGATTTGTGTCCCAA
RAQPLICVPKPRFMPFPS AACCACGATTTATGCCATTTCCCTCCATGTAA
M*
MAEIQDFIQLFLIWLLSTI ATGGCTGAAATCCAAGACTTTATCCAACTTTTCCTAATTTGGCTACTCTCCACCAT
AIRAILTRKQNKEGHSQS TGCAATTCGAGCCATACTAACCCGAAAACAGAACAAGGAGGGTCATAGCCAAA
HPPSPLALPIIGHLHLISQ GCCACCCACCAAGCCCTCTAGCACTACCCATAATTGGACACCTTCACCTTATTTC
LPHQSFHNLSTRYGPIMQ TCAATTACCCCACCAAAGCTTCCACAACCTCTCAACTCGCTATGGACCCATCATG
IFLGSVPCVVASTPEIAKE CAAATCTTCCTAGGCTCTGTCCCTTGTGTAGTAGCTTCCACCCCAGAAATCGCCA
FLKTNEASFSNRFRSAAV AGGAGTTCCTCAAAACCAATGAAGCATCCTTCTCCAACCGTTTCAGAAGTGCTG
HYLSYGSKGFLFAPYGEY CGGTTCACTATTTATCGTATGGCTCAAAGGGGTTCTTGTTTGCGCCTTATGGAGAG
WKFMKKLCVSELLGGRT TACTGGAAGTTCATGAAGAAGCTCTGTGTATCTGAGCTTCTCGGTGGAAGAACA
LDQLSPLRKQETVRFLRL CTCGATCAGCTAAGTCCACTGAGGAAACAAGAGACTGTGAGGTTTCTGAGGCTT
GuI MQSKGEAGVAIDVGGEL ATGCAGAGTAAAGGAGAAGCTGGTGTTGCTATTGATGTTGGTGGGGAGCTATTG
FS4 LTLANSVITRMTMSKTCF ACACTTGCAAATAGTGTCATAACGAGGATGACTATGAGCAAAACGTGTTTTGAA
ENDGDVEDIRKMVKDTA AATGATGGTGATGTGGAAGATATTAGGAAGATGGTGAAGGACACTGCGGAGCTT
ELAGKFNVSDFIWFCQN GCTGGGAAGTTTAATGTGTCTGACTTTATTTGGTTCTGTCAGAATTTGGATCTGCA
LDLQGMNKRLKEILDRF GGGGATGAACAAGAGGCTTAAGGAGATTCTTGATAGGTTTGATACCATGATGGA
DTMMERVIREHEVERKR GAGGGTGATAAGGGAGCATGAAGTGGAAAGGAAGAGAAGGAAGGAAAGGGGT
RKERGEEGANQVMDLL GAAGAAGGAGCTAATCAAGTTATGGACTTGCTTGATATTTTGTTGGAAATACAG
DILLEIQGDERTEMKLTR GGTGATGAGAGAACTGAGATGAAACTGACCAGAGAGAATGTCAAGGCTTTCAT
ENVKAFILDIFMAGTDTS CTTGGACATATTTATGGCAGGAACTGACACGTCAGCTATAACGATCGAATGGGC
AITIEWALAELINHPHVM TCTTGCTGAGTTAATCAACCACCCGCATGTGATGGAGAAAGCAAGACAAGAAAT

	EKARQEIDSVIGKNRLIEE	CGATTCAGTAATAGGAAAGAATAGATTAATAGAAGAGTCAGATCTTCCCAACCT
	SDLPNLPYLRAIVKETLRL	TCCTTACTTGCGAGCTATAGTTAAAGAAACATTAAGGCTTCACCCTGCAGCACCA
	HPAAPLLGRESSESCNVC	CTTCTAGGGAGAGAATCATCTGAAAGTTGCAATGTTTGCGGGTATGAAATTCCAG
	GYEIPAKTVVFVNLWSM	CGAAGACAGTTGTATTTGTTAATTTGTGGTCAATGGGTAGGGACCCAAACATGTG
	GRDPNMWENPLDFRPER	GGAGAACCCACTTGATTTCAGGCCAGAGAGGTTTATAATGGGTGAAGAGAGTGT
	FIMGEESVQLDVRGQNF	TCAGTTAGATGTGAGGGGACAGAATTTCCAACTAATGCCATTTGGGACTGGAAG
	QLMPFGTGRRACPGASL	AAGGGCATGTCCTGGTGCCTCACTTGCACTTCAGATGGTTCCCACTAACCTTGCT
	ALQMVPTNLAAMIQCFE	GCTATGATTCAGTGCTTTGAATGGAAGGTTGTTGATGATGGTGGAGATGGTAATA
	WKVVDDGGDGNNGKVS	ATGGGAAAGTTAGCATGGAAGAGAAACCAGGCATGACCCTTCCAAGGGCTCAT
	MEEKPGMTLPRAHPLTC	CCTTTGACGTGTGTCCCTGTGCCTCGTTTTGATTGCATTCCTTCTTGTGTATAG
	VPVPRFDCIPSCV*	
	MADYQSYTLLFILWLVSII	ATGGCTGATTACCAAAGCTACACCCTACTTTTTATCCTATGGCTAGTGTCCATAAT
	VVRTILTRKQNKAHVPPS	CGTGGTTCGAACCATTCTCACCAGAAAACAGAACAAGGCTCATGTACCACCAAG
	PLALPIIGHLHLLAPIPHQ	CCCACTAGCCTTACCCATAATTGGACACCTTCACCTTCTTGCACCAATACCCCAT
	ALHKLSTRYGPIMQLSLG	CAAGCTCTTCACAAGCTATCAACACGCTATGGACCCATAATGCAACTTTCCCTTG
	SVPCLVASTPESAKEFLKT	GTTCCGTCCCTTGCCTCGTGGCTTCAACACCAGAATCCGCCAAAGAGTTCCTTAA
	HETKFSNRPQSSAVHYLT	AACCCACGAGACCAAATTCTCCAACCGTCCTCAAAGCTCTGCTGTTCATTACCTA
	YGSQDFSFAPYGPYWKF	ACATATGGTTCCCAAGACTTCTCCTTTGCCCCATATGGACCTTATTGGAAATTCAT
	MKRICMSELLGGHTLTLL	GAAGAGAATATGCATGTCTGAACTTCTTGGGGGTCACACACTCACACTGCTTCTT
	LPVRKQETTRFLRLLLKK	CCCGTGAGAAAACAAGAGACGACAAGGTTTCTTAGACTCTTGCTAAAGAAAGG
	GNAGETVDVGGELLTLS	GAATGCAGGTGAGACTGTTGATGTTGGAGGGGAACTTCTGACACTGTCGAATAA
	NNIVSRMIMSQTCSEEDD	CATTGTTTCGAGGATGATTATGAGCCAAACTTGCTCAGAAGAAGATGATGGGGA
	GEGEEVVRKLVQDTVLL	AGGGGAAGAAGTGGTGAGGAAGTTGGTGCAAGACACGGTGCTTCTCACGGGGA
	TGKFNVSDFVWFFKNWD	AGTTTAATGTCTCGGACTTTGTTTGGTTCTTTAAGAATTGGGATTTGCAGGGGTTT
	LQGFGKRLREIRERFDTM	GGTAAAAGGCTTAGGGAGATTCGGGAGAGGTTTGATACCATGATGGAGAAGGT
GuI	MEKVIKEHEEERRKRKEV	GATCAAGGAGCATGAAGAGGAGAGGAGGAAGAGAAAAGAAGTTGGTGGAGGA
FS5	GGGDGRVKDLLDILLDIA	GATGGTCGAGTCAAGGATCTACTCGATATATTGTTGGATATAGCTGAAGATGAG
	EDESSDIKLTMENIKAFIL	AGCTCTGACATAAAATTGACAATGGAGAACATAAAGGCCTTCATCTTGGACATA
	DIFMAGTDTSALTTEWA	TTTATGGCAGGAACAGACACTTCAGCGCTAACCACTGAATGGGCTCTGGCAGAG
	LAELINHPNVMERARKEI	TTGATTAACCACCCAAATGTGATGGAGAGAGCAAGAAAAGAGATTGATGATGT
	DDVVGKTRIVEESDLVNL	AGTTGGGAAAACTAGAATAGTAGAAGAATCAGACCTTGTCAACCTTCCTTATCT
	PYLQAIVKETLRIHPTGPL	ACAAGCTATAGTCAAAGAAACACTGAGGATTCACCCCACAGGTCCTTTAATTGT
	IVRESSEKCTIWGYEIPAK	TAGAGAATCATCTGAAAAATGCACCATATGGGGCTATGAGATTCCAGCAAAGAC
	TQLFVNVWSIGRDPNHW	ACAATTGTTTGTTAATGTGTGGTCTATTGGCAGGGACCCCAATCACTGGGAGAAC
	ENPLEFKPERFIGEEGSGK	CCACTTGAGTTCAAACCAGAGAGATTTATTGGTGAGGAGGGAAGTGGGAAGAGT
	SQLDVRGQHFHLIPFGSG	CAGTTAGATGTGAGGGGACAACATTTTCACCTAATTCCATTTGGGAGTGGAAGA
	RRGCPGTSLALQVVQTN	AGAGGGTGCCCTGGAACCTCACTGGCTTTGCAGGTTGTGCAGACAAACCTTGCT
	LAAMIQCFEWKVPAGQS	GCTATGATTCAGTGTTTTGAATGGAAGGTGCCAGCTGGACAGTCCTACATGGAA
	YMENNNTASIQGEGQNV	AATAATAATACAGCAAGTATACAAGGAGAGGGGCAAAACGTGGAAAAGGAATT
	EKELVVANNPEIQGVRR	GGTTGTTGCTAACAACCCGGAAATTCAGGGAGTGAGACGTGGGATGAGGACAC
	GMRTRTCNSMLREFV*	GGACCTGCAATAGCATGCTGAGGGAATTTGTGTGA
GuI	MADFQDYLQIFLICVLSTI	ATGGCTGATTTTCAAGACTATCTTCAAATCTTCCTCATTTGTGTTCTATCCACCAT
FS6	LVGSILWRKQNKHPHPP	CCTAGTTGGTTCCATACTATGGAGAAAACAAAACAAGCACCCTCATCCACCAGG
	GPLALPIIGHFHLLALLSP	TCCCTTAGCCCTACCCATCATTGGCCACTTCCACCTTTTGGCCCTTCTCTCCCCAC

	LLHSAFHKLSIHYGPIML	TACTTCACTCTGCTTTTCACAAACTCTCAATCCACTATGGCCCCATAATGCTCCTT
	LFMGSVPCIVVSTAEAAK	TTCATGGGCTCTGTCCCTTGTATTGTAGTTTCCACCGCAGAAGCTGCCAAAGAGT
	EFLKTHETSFSNRARTVAI	TTCTCAAAACTCATGAAACCTCCTTTTCCAACCGTGCAAGAACTGTTGCCATTGA
	ETYSYGLQGIVFTPYGDY	GACCTATAGCTATGGTCTTCAAGGCATAGTCTTTACACCCTATGGAGACTATTGG
	WRFIKKLCMSELLGQNIL	AGGTTTATTAAGAAATTGTGCATGTCTGAACTTCTTGGTCAAAATATATTGAATC
	NRFLAVRQQETERFIKLV	GATTCCTTGCTGTGAGGCAGCAAGAGACCGAGAGGTTCATCAAACTTGTGTTCA
	FNKGVEGEAVDFGEQFM	ATAAAGGGGTGGAAGGTGAGGCTGTGGATTTTGGGGAACAATTCATGAAGCTTG
	KLANNIMSRMTISQTSSK	CAAATAATATCATGTCAAGAATGACCATTAGTCAAACAAGTTCTAAGAATGATG
	NDDEANEMMKMVADIT	ATGAAGCAAACGAGATGATGAAGATGGTGGCAGATATTACAGAGATCTTAGGG
	EILGVFNISEFIWFLKRFN	GTGTTCAATATATCGGAATTCATTTGGTTCTTGAAGAGGTTCAATCTACAGGGAC
	LQGHNKRLKEVHDKFDT	ACAACAAGAGGCTAAAGGAAGTTCATGACAAGTTTGATACCAGGCTGAACAGA
	RLNRLIKEHEEERRKRKE	CTGATAAAAGAGCATGAAGAGGAAAGAAGGAAGAGAAAGGAAATGGGTGGAA
	MGGSGTHQSKDILDVLL	GTGGAACTCATCAATCTAAGGATATACTTGATGTGTTATTAGATATATATGAAGA
	DIYEDKSSEMKLTKENIK	TAAAAGTTCAGAAATGAAATTGACTAAAGAGAACATAAAGGCCTTCATCTTGGA
	AFILDIFLAGTDTSALTLD	CATATTTCTTGCTGGGACTGACACGTCAGCATTGACCCTAGACTGGGCTATAGCA
	WAIAELINNPHVMEKAR	GAATTGATCAACAACCCACATGTGATGGAGAAAGCAAGGCAAGAGATAGATGC
	QEIDAVVGKSRIVEESDV	AGTGGTTGGAAAGAGTAGAATAGTGGAAGAATCTGATGTTATGAATCTTCCTCA
	MNLPHLQAIVKETLRLH	CTTGCAAGCCATAGTTAAAGAAACATTAAGGCTTCACCCTGTAGTTCCGATGATT
	PVVPMIFRESSRRDIVGGY	TTCAGGGAGTCATCTAGAAGGGATATAGTTGGTGGTTATGATATTCCAGCCAAGT
	DIPAKSKVFINIWAIGRDP	CTAAAGTATTTATCAATATTTGGGCTATTGGTAGGGACCCCAATCACTGGGAGAA
	NHWENPLEFRPERFIGED	CCCACTTGAGTTTAGGCCAGAAAGGTTTATAGGTGAAGATAATGATCAAATGGA
	NDQMDVRGQHYHLIPF	TGTTAGGGGACAACATTATCATTTGATTCCATTTGGGAGTGGAAGAAGAGTGTGT
	GSGRRVCPGTSLALQVL	CCTGGAACCTCTTTGGCATTGCAGGTTTTGCATGTGAACCTGGGTGCTGTGATTCA
	HVNLGAVIQCFELKVEG	ATGTTTCGAGTTGAAGGTTGAAGGTGGCAATGGCACGATTGACATGGCAGAGAA
	GNGTIDMAEKPGMTMR	GCCTGGCATGACTATGCGCAGGGCTCAACCTCTCATCTGTGTCCCTCTTCCAAGG
	RAQPLICVPLPRLNPFPS	CTTAATCCATTTCCTTCTATGTGA
	M*	
	MESQLMAVSVVLVSALIC	ATGGAATCTCAACTCATGGCGGTGTCTGTAGTGTTGGTTTCAGCACTTATTTGCTA
	YFLFRPYFHRHGKNLPPS	CTTCCTTTTCCGGCCATATTTTCACCGCCACGGAAAAAACCTCCCACCATCTCCTC
	PLFRLPIIGHMHMLGPLL	TGTTTCGGCTTCCAATAATTGGGCACATGCACATGTTGGGTCCCCTTCTCCACCA
	HQSLHNLSHRYGPLFSLN	GTCCCTTCACAATCTCTCTCACCGTTACGGTCCTCTGTTTCTCTTAACTTTGGCTC
	FGSVLCVVASSPHFAKQL	TGTTCTCTGTGTCGTTGCTTCCTCCCCTCACTTCGCCAAACAACTCCTTCAAACCA
	LQTNELAFSSRIETTAVKR	ACGAACTTGCATTTAGCAGTCGCATTGAAACCACCGCCGTTAAACGCCTCACTTA
	LTYESSLAFAPCGDYWRF	CGAATCCTCATTGGCCTTCGCACCCTGTGGTGATTATTGGAGGTTCATCAAGAAG
	IKKLSMNEFLGSRGMNN	CTTAGCATGAACGAGTTCTTGGGCTCACGTGGCATGAACAACTTCCTGCACGTGC
GuI FS7	FLHVRAHETHRLLRLLSN	GTGCACACGAGACTCATCGGTTGTTACGGCTTTTGTCCAACAGGGCAAAAACGT
	RAKTCEAVNLTEELLRLT	GTGAGGCCGTTAATCTCACCGAGGAACTGCTTAGGTTGACCAATAACGTTATTTC
	NNVISRMMLGEAEEARD	TAGGATGATGTTGGGGGAGGCAGAGGAGGCCAGAGATGTAGTGCGTGGTGTGA
	VVRGVSQIFGEFNVSDFI	GCCAGATATTCGGAGAGTTTAATGTCTCGGATTTCATTTGGTTGTTTAAGAAGAT
	WLFKKMDLQGFGKRIED	GGATTTGCAGGGGTTTGGGAAGAGGATAGAGGATTTGTTTCTGAGGTTCGATAC
	LFLRFDTLVERIVCKRVQ	GTTGGTGGAGAGGATTGTGTGCAAACGGGTGCAGATGAGGAAAGAGAAACGAA
	MRKEKRMGNGGSGKQQ	TGGGAAATGGAGGGAGTGGCAAGCAGCAGGGTACTGATGAGGTCAAAGATTTT
	GTDEVKDFLDLLLDCAE	CTTGATCTTTTGCTTGATTGCGCTGAGGATGAGAACTGCGAGGTGAAGATTCGAA
	DENCEVKIRKTQIKALIM	AGACTCAAATTAAGGCCTTGATTATGGATTTCTTCACTGCTGGGACAGATACCAT

	DFFTAGTDTMATSTEWA	GGCGACTTCAACAGAGTGGGCATTAGTGGAGCTTATCAAGAATCCCTTGTTACTA
	LVELIKNPLLLQKAREEID	CAAAAAGCTCGTGAAGAGATAGACAACGTAGTAGGGAATAATAGACTAGTTGA
	NVVGNNRLVEESDYPNL	GGAATCTGACTATCCCAACCTTCCTTATCTCCAAGCCATTATGAAGGAAACATTC
	PYLQAIMKETFRLHPPVP	CGTTTGCACCCACCGGTTCCTATGGTTACAAGAAGATGTGTGACAGAGTGCAGG
	MVTRRCVTECRIENYVIP	ATTGAGAATTACGTCATCCCAGAGAACACACTACTCTTTGTGAATGTTTGGTCCA
	ENTLLFVNVWSIGRNPN	TTGGAAGAAACCCAAACTATTGGGACAACCCATTGGTGTTTCGCCCCGAACGAT
	YWDNPLVFRPERFLKLEE	TCTTAAAGCTCGAAGAAGGAGATTCTAGTGGAGTCATTGATATAAGGGGGCAGC
	GDSSGVIDIRGQHFQLLP	ATTTTCAGCTTCTGCCATTTGGGTCTGGGAGGAGGATGTGCCCTGGTGTGTCCTTG
	FGSGRRMCPGVSLAMQE	GCAATGCAAGAGGTGCCAGCACTTCTTGGTGCTATTATCCAGTGCTTTGATTTCC
	VPALLGAIIQCFDFQVVG	AGGTTGTGGGTTCCAAAGGTGAGATTTTGAAGGGTGATGACATAGTCAATATTG
	SKGEILKGDDIVNIDVDE	ACGTGGATGAAAGGCCAGGATTGACAGCTCCAAGGGCCCACGAGTTGGTGTGTG
	RPGLTAPRAHELVCVPVE	TTCCCGTTGAAAGGACCAGCTGTGGACCCCTTAAAATCCTTGGATGTTGA
	RTSCGPLKILGC*	
	MEPQLVAVSVLVSALICY	ATGGAACCTCAACTCGTAGCAGTGTCTGTGTTGGTTTCAGCACTTATTTGCTACTT
	FFFRPYFHRYGKNLPPSPF	CTTTTTCCGGCCATATTTCCACCGTTACGGAAAAAACCTCCCACCGTCTCCTTTTT
	FRLPIIGHMHMLGPLLH	TCCGGCTTCCAATAATTGGCCACATGCACATGTTAGGTCCCCTTCTTCACCAATC
	QSFHNLSHRYGPLFSLNF	CTTCCACAACCTCTCTCACCGTTACGGTCCTCTGTTTTCACTTAACTTTGGCTCTGT
	GSVLCVVASTPHFAKQLL	TCTCTGTGTCGTTGCTTCCACCCCTCATTTTGCCAAACAACTCCTTCAAACCAACG
	QTNELAFNCRIESTAVKK	AACTCGCCTTTAACTGTCGCATTGAATCAACCGCCGTTAAAAAACTCACTTACGA
	LTYESSLAFAPYGDYWRF	GTCTTCCTTGGCCTTCGCACCTTACGGTGATTACTGGAGGTTCATTAAGAAGCTG
	IKKLSMNELLGSRSINNF	AGCATGAACGAGCTTTTGGGCTCTCGTAGCATAAACAACTTCCAACACCTGCGA
	QHLRAQETHQLLRLLSN	GCACAAGAGACTCATCAATTGTTAAGGCTTTTGTCCAACAGGGCAAGAGCGTTT
	RARAFEAVNITEELLKLT	GAGGCCGTGAATATCACGGAGGAGCTTCTTAAGTTGACCAACAACGTTATTTCTA
	NNVISIMMVGEAEEARD	TAATGATGGTTGGGGAGGCAGAGGAGGCAAGGGATGTGGTGCGTGATGTGACG
	VVRDVTEIFGEFNVSDFI	GAGATCTTTGGAGAGTTTAATGTTTCGGATTTTATTTGGTTGTTTAAGAAGATAGA
	WLFKKIDLQGFGKRIEDL	CTTGCAGGGGTTTGGGAAGAGGATTGAGGATCTGTTTCAGAGGTTTGATACGTTG
	FQRFDTLVERIISKREQTR	GTGGAAAGGATTATTAGCAAGCGGGAGCAGACGAGGAAAGACAGAAGGAGGA
GuI	KDRRRSGKKGEQGSDDG	GTGGGAAGAAGGGTGAGCAGGGGAGTGATGATGGGATCAGAGACTTTCTTGAT
FS8	IRDFLDILLDCTEDENSEI	ATCTTGCTTGACTGTACTGAGGATGAGAATTCCGAGATTAAAATCCAAAGGGTTC
	KIQRVHIKALIMDFFTAG	ACATTAAGGCCTTGATTATGGATTTCTTCACTGCAGGGACGGATACCACAGCGAT
	TDTTAISTEWALVELVKK	TTCAACAGAGTGGGCATTAGTGGAGCTCGTCAAGAAACCCTCCTTGCTACAAAA
	PSLLQKVREEIDNVVGKD	AGTCCGTGAAGAGATAGACAATGTCGTAGGAAAAGACAGACTTGTTGAGGAAT
	RLVEESDCPNLPYLQAIL	CTGATTGTCCCAATCTCCCATATCTCCAAGCCATTCTTAAAGAAACATTCCGTTTG
	KETFRLHPPVPMVTRRCV	CACCCACCGGTTCCTATGGTTACAAGAAGATGCGTGGCAGAGTGCAGGGTAGAG
	AECRVENYVIPEDSLLFV	AATTACGTCATCCCAGAAGACTCACTTCTCTTTGTGAATGTTTGGTCCATTGGGA
	NVWSIGRNPNFWDNPLE	GAAACCCAAACTTTTGGGACAACCCATTGGAGTTTCGCCCCGAACGATTCTTAA
	FRPERFLKLEGDSSGAIDV	AACTAGAAGGAGATTCCAGTGGAGCTATTGATGTGAGGGGAAGCCATTTTCAGC
	RGSHFQLLPFGSGRRMCP	TTCTGCCATTTGGGTCTGGAAGGAGGATGTGTCCTGGTGTGTCCTTGGCTATGCA
	GVSLAMQEVPALIGAIIQ	AGAGGTGCCAGCACTAATTGGTGCTATAATCCAGTGCTTTGATTTCCACGTTGTG
	CFDFHVVGPKGEILKGED	GGTCCCAAAGGTGAGATTTTGAAGGGTGAGGATATAGTCATTAATGTGGATGAA
	IVINVDERPGLTAPRAHN	AGGCCAGGATTGACGGCTCCAAGGGCTCATAACCTTGTGTGTGTTCCCGTTGAAA
	LVCVPVERTSGGGPLKTI	GGACAAGTGGCGGTGGACCCCTCAAAACCATTGGATGTTGA
	GC*	
GuI	MISDHQSILLSLSLLFFAFL	ATGATTTCTGATCATCAGTCCATTTTATTATCCCTCAGTCTGTTGTTCTTTGCTTTC

FS9	LHILSLKRNNKPKPHFRE	CTTCTCCATATCCTCTCGTTGAAGAGAAACAACAAACCAAAGCCCCATTTTCGTG
	PPSPPALPIIGHLHLLKPL	AGCCACCAAGCCCACCAGCACTACCCATAATTGGCCATCTCCATCTCCTCAAAC
	VHQAFRDLSEQHGPLVL	CACTCGTCCATCAAGCCTTCCGCGACCTCTCCGAACAACACGGCCCTCTCGTGTT
	LRLARSAYSFRKTNTAIN	GCTTCGGCTGGCTCGGTCCGCATACTCTTTCCGCAAAACGAACACGGCCATCAA
	TVIYDNATFAFSPHGDY	CACGGTCATCTACGACAACGCCACTTTTGCTTTCTCCCCTCACGGGGACTACTGG
	WKFIKKLSTTELLGNRTI	AAGTTCATCAAGAAGCTCAGCACCACAGAACTCTTGGGCAACCGAACTATTGGA
	GQFLSIRAREVHEFIWAL	CAATTCCTATCCATTCGGGCCCGTGAGGTTCATGAATTCATTTGGGCCTTAGAGA
	ENKSKAQESVNLTQELL	ACAAGTCTAAGGCCCAAGAGAGCGTGAACCTCACTCAGGAGCTCCTGAAGCTTT
	KLSNNIISKMMLSIKSSGT	CCAACAACATAATATCGAAGATGATGCTGAGCATCAAGAGCTCCGGGACAGAC
	DGQAEQARALVREVTEIF	GGCCAGGCGGAGCAAGCGAGGGCTTTGGTTCGCGAGGTGACGGAGATCTTCGG
	GEFNVSDFIGIFKNLDLQ	GGAATTCAACGTCTCCGATTTCATAGGAATCTTCAAGAACTTGGACCTGCAGGGT
	GFKKRAMHIQKRYDALL	TTTAAAAAGAGAGCCATGCACATACAAAAGAGGTACGATGCTCTGTTGGAGAA
	EKIISDREESRRKEEGRCEI	GATCATCTCCGACCGCGAGGAATCGAGAAGGAAGGAAGAGGGACGTTGTGAGA
	NGGEEKVRDFLDILLDFS	TTAACGGAGGAGAAGAGAAAGTGAGGGATTTTCTAGACATTTTGCTTGATTTCTC
	EEKDCEVSLTRNHIKSVIL	TGAGGAGAAAGATTGTGAAGTCAGCTTAACTAGAAACCACATCAAATCAGTCAT
	DYFTAATDTTAISVEWAI	ATTGGATTACTTTACAGCAGCTACGGACACAACAGCCATCTCAGTGGAATGGGC
	AELFNNPRVLKKAQEEV	AATAGCGGAACTGTTCAACAATCCAAGGGTGCTGAAGAAAGCGCAAGAGGAGG
	DRVIGNKRLVCEADSPN	TAGACAGAGTAATTGGGAACAAAAGACTAGTGTGTGAAGCAGACAGTCCAAAC
	LPYIHAIIKETMRLHPPIP	CTTCCTTACATACATGCCATCATAAAAGAGACGATGAGGCTTCACCCACCGATA
	VIMRKGIEDCVVDGYMIP	CCGGTGATCATGAGGAAGGGGATAGAGGACTGCGTGGTTGATGGGTACATGATA
	KGSVVGVNIWAMGRDP	CCAAAAGGCTCAGTAGTCGGTGTGAACATTTGGGCCATGGGAAGGGACCCAAA
	KVWESPLEFRPERFLEGE	AGTGTGGGAAAGCCCATTGGAGTTCAGGCCAGAGAGGTTTCTAGAAGGCGAAA
	KGREIDLKGHHFELLPFG	AAGGGAGAGAGATAGATCTCAAAGGCCATCACTTTGAGTTGTTGCCGTTTGGTTC
	SGRRGCPGMNLAMRELP	TGGGAGGAGGGGTTGCCCTGGAATGAATTTGGCCATGCGGGAATTGCCCGCAAT
	AIIGALVQCFEWKMLDSE	AATTGGAGCATTGGTACAGTGCTTTGAGTGGAAGATGCTTGATTCCGAACGTACG
	RTILDRGRKIDMDERPGL	ATCTTAGACCGTGGTAGAAAAATTGACATGGATGAACGGCCAGGATTGACTGTT
	TVPRATDLICIPVARLNN	CCTCGGGCCACTGATCTTATTTGTATTCCAGTCGCACGATTGAATAATCCCATTCC
	PIPFLQV*	TTTTCTTCAAGTGTAG
	MLVELAITLLVIALFIHLR	ATGTTGGTGGAACTTGCAATTACTCTGTTGGTGATAGCCCTGTTCATACACCTGC
	PTPSAKSKSLRHLPNPPSP	GTCCCACACCAAGTGCAAAATCAAAGTCCCTTCGCCACCTTCCAAACCCTCCAA
	KPRLPFVGHLHLLDKPLL	GTCCAAAACCCCGTCTCCCATTCGTGGGTCACCTTCACCTTTTAGACAAACCCCT
	HNSLIDLSKRYGPLYSLYF	TCTCCACAACTCCCTCATCGATCTAAGCAAACGCTATGGTCCCCTTTACTCCCTCT
	GSMPTVVVSTPELFKLFL	ACTTCGGTTCCATGCCAACCGTTGTAGTCTCCACCCCTGAACTTTTCAAACTCTTC
	QTHEASSFNTRFQTSAIR	CTCCAAACCCACGAGGCCTCTTCCTTCAACACCAGGTTCCAAACCTCTGCCATTA
	RLTYDNSVAMVPFGPYW	GGCGCCTAACCTACGACAACTCTGTTGCCATGGTTCCCTTTGGTCCTTACTGGAA
GuI	KFIRKLIMNDLLNATTVN	GTTCATTAGGAAGCTTATCATGAACGACCTCCTCAATGCCACAACTGTGAACAA
FS10	KLRPLRSQEIRKVLRVMA	GTTGAGGCCTTTAAGGAGCCAGGAAATCCGAAAGGTCCTTAGGGTGATGGCACT
	LSAESQVPLNVTEELLKW	GAGTGCAGAGTCTCAAGTTCCTCTTAATGTTACCGAGGAGCTTCTCAAGTGGACC
	TNSTISRMMLGEAEEIRDI	AACAGCACCATCTCGAGGATGATGCTTGGGGAAGCAGAGGAAATCAGGGACAT
	ARDVLKIFGEYSLTDFIWP	AGCACGTGACGTGCTTAAGATCTTTGGGGAGTATAGTCTCACCGACTTCATCTGG
	LKKLKVGQYEKRIDDIFN	CCCTTGAAGAAACTCAAGGTTGGGCAATACGAGAAGAGGATTGACGATATATTC
	RFDPVIERVIKKRQEIRKK	AACAGGTTTGACCCCGTCATTGAGAGGGTCATCAAGAAAAGACAGGAGATTAG
	RKERNGEVEEGEQSVVFL	GAAGAAGAGGAAGGAGAGGAATGGTGAGGTCGAGGAGGGTGAACAGAGTGTG
	DTLLDFAEDETMEIKITKE	GTTTTTCTCGACACTTTGCTCGATTTTGCTGAGGATGAGACCATGGAGATCAAAA

QIKGLVVDFFSAGTDSTA	TCACCAAGGAACAAATCAAGGGTCTTGTTGTGGATTTCTTCTCAGCAGGGACGG
VATEWALSELINNPKVL	ATTCCACGGCCGTGGCAACAGAATGGGCTCTGTCAGAGCTCATCAACAACCCCA
QKAREEVDAVVGKDRLV	AGGTGCTTCAAAAGGCACGAGAGGAGGTCGATGCGGTTGTGGGAAAAGACAGA
DEADVQNLPYIRSIVKET	CTCGTTGACGAGGCAGATGTCCAGAACCTTCCTTACATTAGATCCATCGTGAAGG
FRMHPPLPVVKRKCVQE	AGACGTTCCGCATGCACCCACCACTACCCGTGGTCAAAAGAAAGTGCGTGCAGG
CEIDGYAIPEGALILFNV	AGTGTGAGATCGACGGTTATGCGATCCCAGAGGGAGCATTGATCCTTTTCAATGT
WAVGRDPKYWDRPTEFR	TTGGGCCGTCGGAAGAGACCCAAAATACTGGGACAGGCCCACTGAGTTCCGTCC
PERFLENVGEGDQAVDL	CGAAAGGTTCTTAGAAAATGTGGGTGAAGGGGATCAAGCCGTTGACCTTAGGGG
RGQHFQLLPFGSGRRMC	TCAACATTTCCAACTTCTTCCATTTGGGTCTGGAAGGAGGATGTGCCCTGGTGTG
PGVNLATAGMATLLASV	AATTTGGCCACTGCGGGAATGGCCACACTGCTTGCGTCAGTTATCCAGTGCTTTG
IQCFDLSVVGPQGKILKG	ATCTCAGCGTGGTGGGCCCACAGGGAAAGATATTGAAGGGCAATGATGCCAAG
NDAKVSMEESAGLTVPR	GTTAGCATGGAAGAGAGTGCTGGACTCACGGTTCCAAGGGCACATAACCTCGTG
AHNLVCVPVARSSAVPK	TGTGTCCCGGTTGCAAGATCAAGTGCCGTACCTAAACTCTTTTCCTCGTGA
LFSS*	

Table S2. The basic properties of the *IFS* genes in the three *Glycyrrhiza* species.

Sequence ID	Original ID	Number of Amino Acid	Molecular Weight	Theoretical pI	Instability Index	Aliphatic Index	Grand Average of Hydropathicity	Prediction of subcellular localization
GgIFS1	GglaChr2G00011970.1	516	58857.42	8.1	42.54	95.41	-0.168	Cytoplasm
GgIFS2	GglaChr2G00011980.1	522	59144.34	6.56	40.57	94.08	-0.211	Cytoplasm
GgIFS3	GglaChr2G00020550.1	525	59356.7	6.23	40.77	89.7	-0.2	Cytoplasm
GgIFS4	GglaChr2G00020570.1	535	60610.13	7.14	45.08	89.27	-0.249	Nucleus
GgIFS5	GglaChr2G00024480.1	514	58163.6	8.86	33.71	93.91	-0.182	Nucleus
GgIFS6	GglaChr4G00127940.1	339	38817.24	8.91	34.84	95.19	-0.096	Endosome; Membrane
GgIFS7	GglaChr4G00127950.1	469	54014.98	6.93	41.35	91.62	-0.219	Membrane
GgIFS8	GglaChr4G00127980.1	530	60410.14	8.52	38.64	98.79	-0.209	Cytoplasm
GgIFS9	GglaChr4G00135790.1	523	59217.88	8.95	39.23	99.48	-0.147	Chloroplast; Cytoplasm; Membrane
GiIFS1	GinfChr2G00015530.1	514	58154.59	8.86	34.08	93.91	-0.183	Nucleus
GiIFS2	GinfChr2G00019460.1	517	58747.04	6.45	47.86	90.7	-0.192	Cytoplasm
GiIFS3	GinfChr2G00019500.1	525	59368.67	6.07	40.76	89.5	-0.2	Membrane
GiIFS4	GinfChr2G00027950.1	522	59132.29	6.56	40.49	93.33	-0.221	Cytoplasm
GiIFS5	GinfChr2G00027960.1	516	58947.45	7.02	44.17	98.99	-0.135	Cytoplasm
GiIFS6	GinfChr4G00048990.1	526	59828.5	7.57	37.69	96.12	-0.087	Membrane
GiIFS7	GinfChr4G00049000.1	523	59505.75	6.72	40.27	96.5	-0.115	Chloroplast
GiIFS8	GinfChr4G00049030.1	530	60322.05	8.3	39.14	98.79	-0.188	Cytoplasm
GiIFS9	GinfChr4G00056940.1	523	59231.87	8.95	40.22	99.29	-0.148	Cytoplasm
GuIFS1	Glycyrrhiza_uralensis_Fisch0056130.1	514	58154.59	8.86	34.08	93.91	-0.183	Nucleus
GuIFS2	Glycyrrhiza_uralensis	517	58716.95	6.45	47.62	90.7	-0.197	Cytoplasm

	_Fisch0059890.1							
GuIFS3	Glycyrrhiza_uralensis_Fisch0059900.1	517	58569.61	7.67	41.91	89.23	-0.236	Nucleus
GuIFS4	Glycyrrhiza_uralensis_Fisch0059930.1	524	59256.58	6.12	40.69	90.06	-0.193	Cytoplasm
GuIFS5	Glycyrrhiza_uralensis_Fisch0068340.1	534	60491.63	6.56	40.21	91.78	-0.269	Cytoplasm
GuIFS6	Glycyrrhiza_uralensis_Fisch0068350.1	516	58859.35	7.04	44.59	95.97	-0.151	Cytoplasm
GuIFS7	Glycyrrhiza_uralensis_Fisch0128220.1	526	59803.49	7.56	38.06	96.31	-0.083	Membrane
GuIFS8	Glycyrrhiza_uralensis_Fisch0128230.1	523	59493.7	6.72	40.43	95.76	-0.125	Chloroplast
GuIFS9	Glycyrrhiza_uralensis_Fisch0128260.1	510	58160.46	8.34	40.21	98.47	-0.225	Cytoplasm
GuIFS10	Glycyrrhiza_uralensis_Fisch0136030.1	523	59203.85	8.95	40.2	99.29	-0.147	Cytoplasm

Table S3. Collinear gene pairs of *IFS* gene family members among three *Glycyrrhiza* species

Chromosome	Gene 1	Chromosome	Gene 2
Chr2	GgIFS1	Chr2	GiIFS1
Chr2	GgIFS2	Chr2	GiIFS2
Chr2	GgIFS2	Chr2	GiIFS4
Chr2	GgIFS4	Chr2	GiIFS4
Chr4	GgIFS9	Chr4	GiIFS9
Chr2	GgIFS1	Chr2	GuIFS1
Chr2	GgIFS2	Chr2	GuIFS2
Chr2	GgIFS2	Chr2	GuIFS5
Chr2	GgIFS4	Chr2	GuIFS5
Chr4	GgIFS9	Chr4	GuIFS10
Chr2	GiIFS1	Chr2	GuIFS1
Chr2	GiIFS2	Chr2	GuIFS2
Chr2	GiIFS2	Chr2	GuIFS5
Chr2	GiIFS4	Chr2	GuIFS5
Chr4	GiIFS9	Chr4	GuIFS10

Table S4. The results of yeast library screening

ID	Description
GglaChr1G00078930	RNA binding molecular function
GglaChr1G00081390	kinase activity molecular function
GglaChr1G00097650	UDP molecular_function
GglaChr1G00103410	Neprosin activation peptide
GglaChr2G00006730	RNA binding molecular function

GglaChr2G00016820	TrHb2_AtGlb3-like_O
GglaChr3G00038530	rRNA processing biological process
GglaChr3G00040550	response to auxin biological process
GglaChr3G00044980	ATP binding molecular function
GglaChr3G00049750	Nucleus cellular component
GglaChr5G00164510	regulation of transcription
GglaChr5G00167650	CYTOCHROME P450
GglaChr5G00182350	metal ion binding molecular function
GglaChr6G00230990	ADP binding molecular function
GglaChr6G00242390	defense response biological process
GglaChr6G00245300	hydrolase activity molecular function
GglaChr6G00248230	hydrolase activity molecular function
GglaChr7G00200000	nucleic acid binding molecular function
GglaChr7G00209080	cytosolic ribosome cellular component
GglaChr7G00209940	nucleus cellular component
GglaChr7G00221740	metal ion binding molecular function
GglaChr7G00230290	AUXIN RESPONSE FACTOR 4
GglaChr8G00273590	ATP-DEPENDENT CLP PROTEASE
GglaChr8G00273610	APO PROTEIN 1
GglaChr8G00275250	UDP molecular function
GglaChr8G00285890	Nucleus cellular component

Table S5. Specific primers of the Gg*IFS* genes detected by qRT-PCR

Primer name	Primer sequence（5'-3'）
GgIFS1-sense	GGCCCTTCTTTCCCCACTAC
GgIFS1-antisense	ACAGTTCTTGCACGGTTGGA
GgIFS2-sense	TCCAACCGTCCTCAAAGCTC
GgIFS2-antisense	CGGGAAGAAGCAGTGTGAGT
GgIFS3-sense	AGCTTCCACCCCAGAAATCG
GgIFS3-antisense	GCGCAAACAAGAACCCCTTT
GgIFS4-sense	GGAGGAGGGGATGGTAGTGT
GgIFS4-antisense	CTTTGGCGGTTTCTGGGTTG
GgIFS5-sense	CACTCATTGGACACGCTCCT
GgIFS5-antisense	GACGGTTGCAAAACGACTCC
GgIFS6-sense	CAGGGCAAAAACGTGTGAGG
GgIFS6-antisense	CATCCTCAGCGCAATCAAGC
GgIFS7-sense	ACTCGCCTTTAACTGTCGCA
GgIFS7-antisense	CTGCCTCCCCAACCATCATT
GgIFS8-sense	AAGGGGATAGAGGACTGCGT
GgIFS8-antisense	TAAGATCAGTGGCCCGAGGA
GgIFS9-sense	CACTGAGTTCCGTCCCGAAA
GgIFS9-antisense	ACGAGGTTATGTGCCCTTGG
TUB-sense	GCTCACTTTCTCTGTCTTCCCATC
TUB-antisense	GCATTCATCGGCATTCTCAACAAG

Table S6. The probe sequence in the EMSA

CAGATGTCTGTCTGTCTGTCTGTCTGTCGTAC

Table S7. The DEGs of RNA-seq

ID	log2FoldChange	lfcSE	stat	pvalue	padj
Glycyrrhiza_uralensis_Fisch0112960.1	-24.78885188	3.907040277	-6.344662486	NA	NA
Glycyrrhiza_uralensis_Fisch0102150.1	-22.66312583	3.908085106	-5.7990359	NA	NA
Glycyrrhiza_uralensis_Fisch0232550.1	-22.65085732	3.908098647	-5.795876555	NA	NA
Glycyrrhiza_uralensis_Fisch0112890.1	-22.09176273	3.229103685	-6.841453508	NA	NA
Glycyrrhiza_uralensis_Fisch0215350.1	-22.04263635	3.204892246	-6.877808881	NA	NA
Glycyrrhiza_uralensis_Fisch0286940.1	-22.04048314	3.242711486	-6.796930049	NA	NA
Glycyrrhiza_uralensis_Fisch0113150.1	-21.93431707	3.284534219	-6.678060148	NA	NA
Glycyrrhiza_uralensis_Fisch0271690.1	-21.5712146	3.429121113	-6.29059572	NA	NA
Glycyrrhiza_uralensis_Fisch0284500.1	-7.521534863	2.69319662	-2.792790845	0.005225547	0.180457547
Glycyrrhiza_uralensis_Fisch0152150.1	-7.40121093	2.633586561	-2.810316182	0.004949285	0.174932884
Glycyrrhiza_uralensis_Fisch0178420.1	-7.384971791	2.545536	-2.901146081	0.003718005	0.148900684
Glycyrrhiza_uralensis_Fisch0199760.1	-7.304894984	1.870669652	-3.904962577	9.42E-05	0.0123391
Glycyrrhiza_uralensis_Fisch0198950.1	-7.228908461	3.526284949	-2.050006895	NA	NA
Glycyrrhiza_uralensis_Fisch0186670.1	-7.197444341	1.533654589	-4.693002187	2.69E-06	0.000800029
Glycyrrhiza_uralensis_Fisch0241000.1	-6.852163984	1.856367517	-3.691167789	0.000223227	0.023647227
Glycyrrhiza_uralensis_Fisch0185540.1	-6.819339645	3.722791747	-1.831781123	0.066984037	0.59232394
Glycyrrhiza_uralensis_Fisch0229640.1	-6.768713127	3.749770163	-1.805100801	0.07105891	0.603706813
Glycyrrhiza_uralensis_Fisch0249700.1	-6.708728789	2.039941072	-3.288687542	0.001006557	0.066743325
Glycyrrhiza_uralensis_Fisch0	-6.54976402	1.412881	-4.635748	3.56E-06	0.001019

200420.1		732	251		103
Glycyrrhiza_uralensis_Fisch0034940.1	-6.453210876	3.912698878	-1.649299135	0.099086367	0.679653036
Glycyrrhiza_uralensis_Fisch0217690.1	-6.453210876	3.912698878	-1.649299135	0.099086367	0.679653036
Glycyrrhiza_uralensis_Fisch0272990.1	-6.427971535	3.912803527	-1.642804575	0.100423373	0.681701613
Glycyrrhiza_uralensis_Fisch0062860.1	-6.245858253	2.248548376	-2.777729098	0.005474024	0.184644923
Glycyrrhiza_uralensis_Fisch0143440.1	-6.212433953	3.146857162	-1.974170937	0.048362309	0.524360715
Glycyrrhiza_uralensis_Fisch0258640.1	-6.208564714	2.09400122	-2.96492889	0.003027529	0.134949303
Glycyrrhiza_uralensis_Fisch0100320.1	-6.183889877	3.010583645	-2.05405018	0.039970835	0.486678992
Glycyrrhiza_uralensis_Fisch0049700.1	-6.170622276	2.172981514	-2.839703069	0.004515554	0.165938007
Glycyrrhiza_uralensis_Fisch0081090.1	-6.165519236	3.062377523	-2.013311288	0.044081892	0.505740037
Glycyrrhiza_uralensis_Fisch0189520.1	-6.156745613	3.01699557	-2.040687655	0.041281883	0.493864279
Glycyrrhiza_uralensis_Fisch0230330.1	-6.13129766	3.914180952	-1.566431837	0.117247554	0.706303999
Glycyrrhiza_uralensis_Fisch0036620.1	-6.129251742	3.914191467	-1.565904937	0.117370874	0.706303999
Glycyrrhiza_uralensis_Fisch0212290.1	-6.106100483	3.914311492	-1.559942405	0.118773492	0.710103816
Glycyrrhiza_uralensis_Fisch0267020.1	-6.093997307	2.179503512	-2.796048399	0.005173165	0.179420876
Glycyrrhiza_uralensis_Fisch0150840.1	-6.087424057	3.914409729	-1.555132058	0.119914621	0.711449612
Glycyrrhiza_uralensis_Fisch0273360.1	-6.044347396	3.914641209	-1.544036113	0.122579584	0.714644751
Glycyrrhiza_uralensis_Fisch0027710.1	-5.971128389	3.152023826	-1.894379204	0.058174695	0.562355382
Glycyrrhiza_uralensis_Fisch0026590.1	-5.909230779	3.180221773	-1.858119088	0.063152105	0.577986481
Glycyrrhiza_uralensis_Fisch0233670.1	-5.868276958	3.915662443	-1.498667733	0.133959851	0.730409401
Glycyrrhiza_uralensis_Fisch0154890.1	-5.847272812	3.214739356	-1.818894836	0.068927476	0.597968345
Glycyrrhiza_uralensis_Fisch0139330.1	-5.778243589	2.286687405	-2.526905766	0.011507237	0.266647518
Glycyrrhiza_uralensis_Fisch0	-5.771123223	2.278013	-2.533401	0.011296	0.264575

032420.1		552	62	144	469
Glycyrrhiza_uralensis_Fisch0257690.1	-5.769147155	2.405387353	-2.398427492	0.016465636	0.315815568
Glycyrrhiza_uralensis_Fisch0272480.1	-5.756366631	3.231113649	-1.781542606	0.074823851	0.618340453
Glycyrrhiza_uralensis_Fisch0036860.1	-5.745438019	3.331378508	-1.724642818	0.084591862	0.643325787
Glycyrrhiza_uralensis_Fisch0193720.1	-5.716282455	3.916649781	-1.459482664	0.144432309	0.744212491
Glycyrrhiza_uralensis_Fisch0251790.1	-5.716282455	3.916649781	-1.459482664	0.144432309	0.744212491
Glycyrrhiza_uralensis_Fisch0051840.1	-5.700319635	3.916759623	-1.455366217	0.145567904	0.744859576
Glycyrrhiza_uralensis_Fisch0224030.1	-5.643721937	3.917158993	-1.440769176	0.149649904	0.752312697
Glycyrrhiza_uralensis_Fisch0154120.1	-5.575206243	3.383891003	-1.647572643	0.099440398	0.680028712
Glycyrrhiza_uralensis_Fisch0022600.1	-5.57029647	2.565283186	-2.171415811	0.029899755	0.425598337
Glycyrrhiza_uralensis_Fisch0233140.1	-5.552861819	3.432293775	-1.617828246	0.105699617	0.692097679
Glycyrrhiza_uralensis_Fisch0018710.1	-5.544486891	2.358023523	-2.351328066	NA	NA
Glycyrrhiza_uralensis_Fisch0258800.1	-5.48269896	2.527874769	-2.168896588	0.030090536	0.426204537
Glycyrrhiza_uralensis_Fisch0188550.1	-5.473134464	3.405933468	-1.606941097	0.10806729	0.69307452
Glycyrrhiza_uralensis_Fisch0032010.1	-5.452254895	3.49180307	-1.561443983	0.118419031	0.708895317
Glycyrrhiza_uralensis_Fisch0035920.1	-5.4279127	3.504016394	-1.549054596	0.121368596	0.713577584
Glycyrrhiza_uralensis_Fisch0140300.1	-5.426157554	3.534386825	-1.535247222	0.124723113	0.719234626
Glycyrrhiza_uralensis_Fisch0228350.1	-5.409599342	3.448625156	-1.568624915	0.116735363	0.706303999
Glycyrrhiza_uralensis_Fisch0042000.1	-5.406194369	3.453584876	-1.565386276	0.117492364	0.706303999
Glycyrrhiza_uralensis_Fisch0048510.1	-5.39578062	3.919104982	-1.376788998	0.168577493	0.776340257
Glycyrrhiza_uralensis_Fisch0062360.1	-5.367281286	2.540298743	-2.112854364	0.034613235	0.45379288
Glycyrrhiza_uralensis_Fisch0070320.1	-5.365646748	2.577855736	-2.081437946	0.037393838	0.472725678
Glycyrrhiza_uralensis_Fisch0	-5.353734877	3.919469	-1.365933	0.171959	0.779233

273370.1		328	607	803	384
Glycyrrhiza_uralensis_Fisch0026050.1	-5.353734877	3.919469328	-1.365933607	0.171959803	0.779233384
Glycyrrhiza_uralensis_Fisch0189900.1	-5.349047461	3.550374613	-1.506614947	0.131909384	0.727923
Glycyrrhiza_uralensis_Fisch0155110.1	-5.298786603	3.532156186	-1.50015637	0.133573902	0.73004628
Glycyrrhiza_uralensis_Fisch0169900.1	-5.287613509	2.548330633	-2.074932287	0.037992811	0.476791948
Glycyrrhiza_uralensis_Fisch0022340.1	-5.283350174	3.920103438	-1.347757848	0.177736279	0.788665611
Glycyrrhiza_uralensis_Fisch0242800.1	-5.268473776	3.548605188	-1.48466045	0.137633793	0.735697404
Glycyrrhiza_uralensis_Fisch0112900.1	-5.233621291	3.58308066	-1.460648472	0.144111937	0.744212491
Glycyrrhiza_uralensis_Fisch0225180.1	-5.218347298	2.610739121	-1.998800744	0.045629917	0.511080553
Glycyrrhiza_uralensis_Fisch0223060.1	-5.216526553	2.611162093	-1.997779673	0.045740552	0.511843369
Glycyrrhiza_uralensis_Fisch0106470.1	-5.209354841	3.920804172	-1.328644485	0.183965288	0.792181538
Glycyrrhiza_uralensis_Fisch0029950.1	-5.209354841	3.920804172	-1.328644485	0.183965288	0.792181538
Glycyrrhiza_uralensis_Fisch0277630.1	-5.204680211	3.60849451	-1.442341175	0.14920615	0.751337244
Glycyrrhiza_uralensis_Fisch0156730.1	-5.184344769	3.921049231	-1.322183034	0.186107209	0.795073375
Glycyrrhiza_uralensis_Fisch0163480.1	-5.169647688	3.921195226	-1.318385694	0.187374567	0.797211647
Glycyrrhiza_uralensis_Fisch0264840.1	-5.151304126	2.778342766	-1.854092371	0.063725946	NA
Glycyrrhiza_uralensis_Fisch0241590.1	-5.141849536	3.639833253	-1.41266074	0.1577555	NA
Glycyrrhiza_uralensis_Fisch0131020.1	-5.125876056	3.693630675	-1.387760853	0.165209879	NA
Glycyrrhiza_uralensis_Fisch0148280.1	-5.106369718	3.921841007	-1.302033843	0.192904817	NA
Glycyrrhiza_uralensis_Fisch0156230.1	-5.075965148	3.712870428	-1.367126929	0.171585524	NA
Glycyrrhiza_uralensis_Fisch0109840.1	-5.057991297	3.743183114	-1.351254038	0.17661407	NA
Glycyrrhiza_uralensis_Fisch0275670.1	-5.029898775	3.758840055	-1.33815185	0.180846941	NA
Glycyrrhiza_uralensis_Fisch0	-4.999658345	3.922996	-1.274448	0.202504	NA

230000.1		037	992	339	
Glycyrrhiza_uralensis_Fisch0029410.1	-4.999658345	3.922996037	-1.274448992	0.202504339	NA
Glycyrrhiza_uralensis_Fisch0111810.1	-4.999658345	3.922996037	-1.274448992	0.202504339	NA
Glycyrrhiza_uralensis_Fisch0038940.1	-4.97627814	3.772263965	-1.319175483	0.187110453	NA
Glycyrrhiza_uralensis_Fisch0273350.1	-4.975881156	3.772522566	-1.318979826	0.187175857	NA
Glycyrrhiza_uralensis_Fisch0092570.1	-4.961446287	3.92343077	-1.264568328	0.206026113	NA
Glycyrrhiza_uralensis_Fisch0154130.1	-4.955269223	3.81330168	-1.299469499	0.193782854	NA
Glycyrrhiza_uralensis_Fisch0213190.1	-4.950618446	3.782608888	-1.30878412	0.190607487	NA
Glycyrrhiza_uralensis_Fisch0216710.1	-4.949063414	3.78166	-1.308701315	0.190635546	NA
Glycyrrhiza_uralensis_Fisch0115370.1	-4.949063414	3.78166	-1.308701315	0.190635546	NA
Glycyrrhiza_uralensis_Fisch0030090.1	-4.942813826	3.8212998	-1.293490196	0.195841573	NA
Glycyrrhiza_uralensis_Fisch0053620.1	-4.896413048	3.859720152	-1.268592762	0.204586347	NA
Glycyrrhiza_uralensis_Fisch0151860.1	-4.878616329	3.871492376	-1.260138431	0.207619429	NA
Glycyrrhiza_uralensis_Fisch0031620.1	-4.876056015	3.898108372	-1.25087749	0.210979178	NA
Glycyrrhiza_uralensis_Fisch0029730.1	-4.868344606	3.924539232	-1.240488199	0.214794876	NA
Glycyrrhiza_uralensis_Fisch0239620.1	-4.851801465	3.873666421	-1.252508848	0.210384506	NA
Glycyrrhiza_uralensis_Fisch0108650.1	-4.851801465	3.873666421	-1.252508848	0.210384506	NA
Glycyrrhiza_uralensis_Fisch0210060.1	-4.806930377	3.925310389	-1.224598796	0.220726422	NA
Glycyrrhiza_uralensis_Fisch0201660.1	-4.796139854	3.925449289	-1.221806601	0.221780772	NA
Glycyrrhiza_uralensis_Fisch0115430.1	-4.796139854	3.925449289	-1.221806601	0.221780772	NA
Glycyrrhiza_uralensis_Fisch0054430.1	-4.796139854	3.925449289	-1.221806601	0.221780772	NA
Glycyrrhiza_uralensis_Fisch0209280.1	-4.785269596	3.921433723	-1.220285726	0.222356579	NA
Glycyrrhiza_uralensis_Fisch0	-4.77980609	3.925661	-1.217579	0.223383	NA

055610.1		52	78	702	
Glycyrrhiza_uralensis_Fisch0154860.1	-4.77426622	2.212115494	-2.158235513	0.030909526	0.430680825
Glycyrrhiza_uralensis_Fisch0248740.1	-4.768817591	3.92580565	-1.214736035	0.224466784	NA
Glycyrrhiza_uralensis_Fisch0238860.1	-4.767776686	2.081027992	-2.291068022	0.02195948	0.363774727
Glycyrrhiza_uralensis_Fisch0053450.1	-4.743947116	3.926135924	-1.208299256	0.226932175	NA
Glycyrrhiza_uralensis_Fisch0080230.1	-4.699964552	3.926734053	-1.196914405	0.231339919	NA
Glycyrrhiza_uralensis_Fisch0219530.1	-4.699964552	3.926734053	-1.196914405	0.231339919	NA
Glycyrrhiza_uralensis_Fisch0112460.1	-4.679593826	3.927017287	-1.19164075	0.233402139	NA
Glycyrrhiza_uralensis_Fisch0192210.1	-4.6651765	2.991676691	-1.559385248	0.118905226	NA
Glycyrrhiza_uralensis_Fisch0230970.1	-4.661912286	3.927266376	-1.187062918	0.235202804	NA
Glycyrrhiza_uralensis_Fisch0155970.1	-4.650502837	3.927428727	-1.18410878	0.236370005	NA
Glycyrrhiza_uralensis_Fisch0049830.1	-4.648592875	3.92745603	-1.183614238	0.236565802	NA
Glycyrrhiza_uralensis_Fisch0243450.1	-4.646680483	3.927483403	-1.183119063	0.236761964	NA
Glycyrrhiza_uralensis_Fisch0112480.1	-4.637228219	1.739332216	-2.666096894	0.007673757	0.221471627
Glycyrrhiza_uralensis_Fisch0113130.1	-4.584427322	3.928394482	-1.166997699	0.243211299	NA
Glycyrrhiza_uralensis_Fisch0271980.1	-4.571872466	3.928583009	-1.163745925	0.244526992	NA
Glycyrrhiza_uralensis_Fisch0182380.1	-4.571872466	3.928583009	-1.163745925	0.244526992	NA
Glycyrrhiza_uralensis_Fisch0235760.1	-4.56379339	3.928705192	-1.16165331	0.245376318	NA
Glycyrrhiza_uralensis_Fisch0144560.1	-4.551091127	3.029533789	-1.502241416	0.133034772	NA
Glycyrrhiza_uralensis_Fisch0248640.1	-4.54644615	3.928969851	-1.157159846	0.247207059	NA
Glycyrrhiza_uralensis_Fisch0011160.1	-4.54644615	3.928969851	-1.157159846	0.247207059	NA
Glycyrrhiza_uralensis_Fisch0011170.1	-4.54644615	3.928969851	-1.157159846	0.247207059	NA
Glycyrrhiza_uralensis_Fisch0	-4.54644615	3.928969	-1.157159	0.247207	NA

160180.1		851	846	059	
Glycyrrhiza_uralensis_Fisch0175160.1	-4.54644615	3.928969851	-1.157159846	0.247207059	NA
Glycyrrhiza_uralensis_Fisch0116220.1	-4.54644615	3.928969851	-1.157159846	0.247207059	NA
Glycyrrhiza_uralensis_Fisch0131410.1	-4.54287444	3.929024737	-1.156234624	0.247585201	NA
Glycyrrhiza_uralensis_Fisch0062490.1	-4.54287444	3.929024737	-1.156234624	0.247585201	NA
Glycyrrhiza_uralensis_Fisch0155940.1	-4.538230068	3.929096309	-1.155031517	0.24807752	NA
Glycyrrhiza_uralensis_Fisch0012990.1	-4.532038682	3.929192077	-1.153427624	0.248734909	NA
Glycyrrhiza_uralensis_Fisch0036640.1	-4.529969231	3.929224179	-1.152891519	0.248954914	NA
Glycyrrhiza_uralensis_Fisch0235170.1	-4.521663263	3.929353484	-1.150739754	0.249839316	NA
Glycyrrhiza_uralensis_Fisch0117850.1	-4.521663263	3.929353484	-1.150739754	0.249839316	NA
Glycyrrhiza_uralensis_Fisch0259880.1	-4.458826242	3.930356068	-1.1344586	0.25660223	NA
Glycyrrhiza_uralensis_Fisch0152710.1	-4.44200038	3.930631979	-1.130098265	0.258434821	NA
Glycyrrhiza_uralensis_Fisch0276140.1	-4.44200038	3.930631979	-1.130098265	0.258434821	NA
Glycyrrhiza_uralensis_Fisch0075090.1	-4.441716582	3.930636661	-1.130024717	0.258465809	NA
Glycyrrhiza_uralensis_Fisch0141020.1	-4.413537117	3.931106066	-1.122721454	0.261555813	NA
Glycyrrhiza_uralensis_Fisch0029690.1	-4.409738152	3.931170047	-1.121736811	0.26197436	NA
Glycyrrhiza_uralensis_Fisch0037830.1	-4.396200604	3.931399412	-1.118227924	0.263469661	NA
Glycyrrhiza_uralensis_Fisch0135690.1	-4.338333726	1.523678936	-2.847275514	0.004409518	0.163426587
Glycyrrhiza_uralensis_Fisch0065140.1	-4.306230667	3.932979321	-1.094902952	0.273559156	NA
Glycyrrhiza_uralensis_Fisch0249940.1	-4.306230667	3.932979321	-1.094902952	0.273559156	NA
Glycyrrhiza_uralensis_Fisch0231650.1	-4.302766427	3.933042133	-1.094004662	0.273952934	NA
Glycyrrhiza_uralensis_Fisch0111050.1	-4.294877622	3.93318573	-1.091959017	0.274851115	NA
Glycyrrhiza_uralensis_Fisch0	-4.283439853	3.933395	-1.088992	0.276156	NA

179360.1		318	971	985	
Glycyrrhiza_uralensis_Fisch0 184840.1	-4.283439853	3.933395 318	-1.088992 971	0.276156 985	NA
Glycyrrhiza_uralensis_Fisch0 167570.1	-4.283439853	3.933395 318	-1.088992 971	0.276156 985	NA
Glycyrrhiza_uralensis_Fisch0 155300.1	-4.283439853	3.933395 318	-1.088992 971	0.276156 985	NA
Glycyrrhiza_uralensis_Fisch0 135110.1	-4.283439853	3.933395 318	-1.088992 971	0.276156 985	NA
Glycyrrhiza_uralensis_Fisch0 106110.1	-4.283439853	3.933395 318	-1.088992 971	0.276156 985	NA
Glycyrrhiza_uralensis_Fisch0 265190.1	-4.283439853	3.933395 318	-1.088992 971	0.276156 985	NA
Glycyrrhiza_uralensis_Fisch0 175690.1	-4.283439853	3.933395 318	-1.088992 971	0.276156 985	NA
Glycyrrhiza_uralensis_Fisch0 053660.1	-4.276083036	3.933531	-1.087085 124	0.276999 191	NA
Glycyrrhiza_uralensis_Fisch0 139800.1	-4.227635722	2.452685 082	-1.723676 534	0.084766 253	0.643723 846
Glycyrrhiza_uralensis_Fisch0 222220.1	-4.161508352	2.991354 419	-1.391178 633	0.164171 265	0.770057 013
Glycyrrhiza_uralensis_Fisch0 029570.1	-4.156675259	3.935832 097	-1.056110 92	0.290917 542	NA
Glycyrrhiza_uralensis_Fisch0 272790.1	-4.148657678	3.935993 483	-1.054030 627	0.291868 895	NA
Glycyrrhiza_uralensis_Fisch0 235550.1	-4.139907548	3.936170 633	-1.051760 184	0.292909 591	NA
Glycyrrhiza_uralensis_Fisch0 041450.1	-4.131454848	3.936342 778	-1.049566 839	0.293917 31	NA
Glycyrrhiza_uralensis_Fisch0 180050.1	-4.131454848	3.936342 778	-1.049566 839	0.293917 31	NA
Glycyrrhiza_uralensis_Fisch0 233720.1	-4.131454848	3.936342 778	-1.049566 839	0.293917 31	NA
Glycyrrhiza_uralensis_Fisch0 138930.1	-4.127907155	3.936415 327	-1.048646 246	0.294340 963	NA
Glycyrrhiza_uralensis_Fisch0 035120.1	-4.115111153	3.936678 479	-1.045325 691	0.295872 468	NA
Glycyrrhiza_uralensis_Fisch0 032760.1	-4.106873856	3.936849 11	-1.043188 027	0.296861 218	NA
Glycyrrhiza_uralensis_Fisch0 250010.1	-4.022753879	1.530387 975	-2.628584 35	0.008574 109	0.231916 014
Glycyrrhiza_uralensis_Fisch0 111110.1	-3.999079396	3.939173 435	-1.015207 749	0.310006 807	NA
Glycyrrhiza_uralensis_Fisch0	-3.999079396	3.939173	-1.015207	0.310006	NA

113110.1		435	749	807	
Glycyrrhiza_uralensis_Fisch0 272590.1	-3.999079396	3.939173 435	-1.015207 749	0.310006 807	NA
Glycyrrhiza_uralensis_Fisch0 036510.1	-3.999079396	3.939173 435	-1.015207 749	0.310006 807	NA
Glycyrrhiza_uralensis_Fisch0 027820.1	-3.999079396	3.939173 435	-1.015207 749	0.310006 807	NA
Glycyrrhiza_uralensis_Fisch0 105850.1	-3.999079396	3.939173 435	-1.015207 749	0.310006 807	NA
Glycyrrhiza_uralensis_Fisch0 015380.1	-3.999079396	3.939173 435	-1.015207 749	0.310006 807	NA
Glycyrrhiza_uralensis_Fisch0 108310.1	-3.999079396	3.939173 435	-1.015207 749	0.310006 807	NA
Glycyrrhiza_uralensis_Fisch0 117150.1	-3.985112515	3.939487 4	-1.011581 485	0.311738 202	NA
Glycyrrhiza_uralensis_Fisch0 232510.1	-3.985112515	3.939487 4	-1.011581 485	0.311738 202	NA
Glycyrrhiza_uralensis_Fisch0 031110.1	-3.984255162	0.750704 814	-5.307352 62	1.11E-07	5.82E-05
Glycyrrhiza_uralensis_Fisch0 013720.1	-3.980428842	3.939593 363	-1.010365 405	0.312320 256	NA
Glycyrrhiza_uralensis_Fisch0 228550.1	-3.980428842	3.939593 363	-1.010365 405	0.312320 256	NA
Glycyrrhiza_uralensis_Fisch0 129580.1	-3.980428842	3.939593 363	-1.010365 405	0.312320 256	NA
Glycyrrhiza_uralensis_Fisch0 184690.1	-3.980428842	3.939593 363	-1.010365 405	0.312320 256	NA
Glycyrrhiza_uralensis_Fisch0 122130.1	-3.980428842	3.939593 363	-1.010365 405	0.312320 256	NA
Glycyrrhiza_uralensis_Fisch0 030930.1	-3.979021031	3.594899 604	-1.106851 781	0.268358 016	NA
Glycyrrhiza_uralensis_Fisch0 072370.1	-3.976114982	3.939691 261	-1.009245 324	0.312856 995	NA
Glycyrrhiza_uralensis_Fisch0 100720.1	-3.976114982	3.939691 261	-1.009245 324	0.312856 995	NA
Glycyrrhiza_uralensis_Fisch0 081590.1	-3.968384085	3.939867 432	-1.007237 973	0.313820 429	NA
Glycyrrhiza_uralensis_Fisch0 166950.1	-3.961551892	3.940023 905	-1.005463 923	0.314673 513	NA
Glycyrrhiza_uralensis_Fisch0 077890.1	-3.961551892	3.940023 905	-1.005463 923	0.314673 513	NA
Glycyrrhiza_uralensis_Fisch0 229650.1	-3.961551892	3.940023 905	-1.005463 923	0.314673 513	NA
Glycyrrhiza_uralensis_Fisch0	-3.961551892	3.940023	-1.005463	0.314673	NA

182530.1		905	923	513	
Glycyrrhiza_uralensis_Fisch0071380.1	-3.961551892	3.940023905	-1.005463923	0.314673513	NA
Glycyrrhiza_uralensis_Fisch0255270.1	-3.961551892	3.940023905	-1.005463923	0.314673513	NA
Glycyrrhiza_uralensis_Fisch0111040.1	-3.961551892	3.940023905	-1.005463923	0.314673513	NA
Glycyrrhiza_uralensis_Fisch0274910.1	-3.961551892	3.940023905	-1.005463923	0.314673513	NA
Glycyrrhiza_uralensis_Fisch0108670.1	-3.961551892	3.940023905	-1.005463923	0.314673513	NA
Glycyrrhiza_uralensis_Fisch0255510.1	-3.961551892	3.940023905	-1.005463923	0.314673513	NA
Glycyrrhiza_uralensis_Fisch0252190.1	-3.961551892	3.940023905	-1.005463923	0.314673513	NA
Glycyrrhiza_uralensis_Fisch0032800.1	-3.961551892	3.940023905	-1.005463923	0.314673513	NA
Glycyrrhiza_uralensis_Fisch0032650.1	-3.961551892	3.940023905	-1.005463923	0.314673513	NA
Glycyrrhiza_uralensis_Fisch0065510.1	-3.961551892	3.940023905	-1.005463923	0.314673513	NA
Glycyrrhiza_uralensis_Fisch0242380.1	-3.961551892	3.940023905	-1.005463923	0.314673513	NA
Glycyrrhiza_uralensis_Fisch0026060.1	-3.961551892	3.940023905	-1.005463923	0.314673513	NA
Glycyrrhiza_uralensis_Fisch0276400.1	-3.961551892	3.940023905	-1.005463923	0.314673513	NA
Glycyrrhiza_uralensis_Fisch0092340.1	-3.961551892	3.940023905	-1.005463923	0.314673513	NA
Glycyrrhiza_uralensis_Fisch0177470.1	-3.952416541	3.940234277	-1.003091761	0.315816589	NA
Glycyrrhiza_uralensis_Fisch0220620.1	-3.949359454	3.940304972	-1.002297914	0.31619973	NA
Glycyrrhiza_uralensis_Fisch0272420.1	-3.949359454	3.940304972	-1.002297914	0.31619973	NA
Glycyrrhiza_uralensis_Fisch0209700.1	-3.949359454	3.940304972	-1.002297914	0.31619973	NA
Glycyrrhiza_uralensis_Fisch0167480.1	-3.949359454	3.940304972	-1.002297914	0.31619973	NA
Glycyrrhiza_uralensis_Fisch0158380.1	-3.949359454	3.940304972	-1.002297914	0.31619973	NA
Glycyrrhiza_uralensis_Fisch0015450.1	-3.949359454	3.940304972	-1.002297914	0.31619973	NA
Glycyrrhiza_uralensis_Fisch0	-3.949359454	3.940304	-1.002297	0.316199	NA

048600.1		972	914	73	
Glycyrrhiza_uralensis_Fisch0011680.1	-3.942681369	2.55012703	-1.546072538	0.12208705	0.714260574
Glycyrrhiza_uralensis_Fisch0222570.1	-3.937070667	3.940590654	-0.999106736	0.317742988	NA
Glycyrrhiza_uralensis_Fisch0208030.1	-3.937070667	3.940590654	-0.999106736	0.317742988	NA
Glycyrrhiza_uralensis_Fisch0050180.1	-3.937070667	3.940590654	-0.999106736	0.317742988	NA
Glycyrrhiza_uralensis_Fisch0059790.1	-3.937070667	3.940590654	-0.999106736	0.317742988	NA
Glycyrrhiza_uralensis_Fisch0237070.1	-3.937070667	3.940590654	-0.999106736	0.317742988	NA
Glycyrrhiza_uralensis_Fisch0238170.1	-3.937070667	3.940590654	-0.999106736	0.317742988	NA
Glycyrrhiza_uralensis_Fisch0174300.1	-3.937070667	3.940590654	-0.999106736	0.317742988	NA
Glycyrrhiza_uralensis_Fisch0074240.1	-3.937070667	3.940590654	-0.999106736	0.317742988	NA
Glycyrrhiza_uralensis_Fisch0271840.1	-3.937070667	3.940590654	-0.999106736	0.317742988	NA
Glycyrrhiza_uralensis_Fisch0275690.1	-3.937070667	3.940590654	-0.999106736	0.317742988	NA
Glycyrrhiza_uralensis_Fisch0019520.1	-3.812066261	1.966041742	-1.938954895	0.052506831	0.538262101
Glycyrrhiza_uralensis_Fisch0230740.1	-3.806270702	3.943785669	-0.965131227	0.334479088	NA
Glycyrrhiza_uralensis_Fisch0082500.1	-3.806270702	3.943785669	-0.965131227	0.334479088	NA
Glycyrrhiza_uralensis_Fisch0189700.1	-3.806270702	3.943785669	-0.965131227	0.334479088	NA
Glycyrrhiza_uralensis_Fisch0051810.1	-3.806270702	3.943785669	-0.965131227	0.334479088	NA
Glycyrrhiza_uralensis_Fisch0112070.1	-3.806270702	3.943785669	-0.965131227	0.334479088	NA
Glycyrrhiza_uralensis_Fisch0170500.1	-3.790377864	3.944193801	-0.961001932	0.336551196	NA
Glycyrrhiza_uralensis_Fisch0200810.1	-3.778167254	3.674601119	-1.02818432	0.303863133	NA
Glycyrrhiza_uralensis_Fisch0115690.1	-3.772830698	3.67814231	-1.025743536	0.305012487	NA
Glycyrrhiza_uralensis_Fisch0179760.1	-3.768934346	3.944751582	-0.955430087	0.339360245	NA
Glycyrrhiza_uralensis_Fisch0	-3.768934346	3.944751	-0.955430	0.339360	NA

240720.1		582	087	245	
Glycyrrhiza_uralensis_Fisch0031380.1	-3.758542999	3.945024846	-0.952729868	0.340726956	NA
Glycyrrhiza_uralensis_Fisch0074910.1	-3.755063885	3.945116774	-0.951825789	0.341185341	NA
Glycyrrhiza_uralensis_Fisch0163750.1	-3.747418385	1.871308735	-2.002565539	0.045223942	0.509005012
Glycyrrhiza_uralensis_Fisch0133540.1	-3.745976	2.04129885	-1.835094357	NA	NA
Glycyrrhiza_uralensis_Fisch0228280.1	-3.744580093	3.945395115	-0.949101417	0.342569034	NA
Glycyrrhiza_uralensis_Fisch0189530.1	-3.744580093	3.945395115	-0.949101417	0.342569034	NA
Glycyrrhiza_uralensis_Fisch0157310.1	-3.744580093	3.945395115	-0.949101417	0.342569034	NA
Glycyrrhiza_uralensis_Fisch0132900.1	-3.744580093	3.945395115	-0.949101417	0.342569034	NA
Glycyrrhiza_uralensis_Fisch0048710.1	-3.744580093	3.945395115	-0.949101417	0.342569034	NA
Glycyrrhiza_uralensis_Fisch0009060.1	-3.744580093	3.945395115	-0.949101417	0.342569034	NA
Glycyrrhiza_uralensis_Fisch0154620.1	-3.683878483	3.301105652	-1.115952918	0.264442289	0.85183092
Glycyrrhiza_uralensis_Fisch0230370.1	-3.623581309	2.008474602	-1.804145945	0.071208431	0.603706813
Glycyrrhiza_uralensis_Fisch0221300.1	-3.583662615	3.949928469	-0.907272788	0.36426256	NA
Glycyrrhiza_uralensis_Fisch0232400.1	-3.583662615	3.949928469	-0.907272788	0.36426256	NA
Glycyrrhiza_uralensis_Fisch0150400.1	-3.583302389	1.760495774	-2.035393917	0.041811253	0.49475144
Glycyrrhiza_uralensis_Fisch0039680.1	-3.570915664	2.049351612	-1.742461197	0.081427776	0.636559454
Glycyrrhiza_uralensis_Fisch0132020.1	-3.546577528	3.951046088	-0.897630007	0.369382836	NA
Glycyrrhiza_uralensis_Fisch0018640.1	-3.546577528	3.951046088	-0.897630007	0.369382836	NA
Glycyrrhiza_uralensis_Fisch0242470.1	-3.546577528	3.951046088	-0.897630007	0.369382836	NA
Glycyrrhiza_uralensis_Fisch0112090.1	-3.546577528	3.951046088	-0.897630007	0.369382836	NA
Glycyrrhiza_uralensis_Fisch0240230.1	-3.546577528	3.951046088	-0.897630007	0.369382836	NA
Glycyrrhiza_uralensis_Fisch0	-3.546577528	3.951046	-0.897630	0.369382	NA

176190.1		088	007	836	
Glycyrrhiza_uralensis_Fisch0193910.1	-3.546577528	3.951046088	-0.897630007	0.369382836	NA
Glycyrrhiza_uralensis_Fisch0054450.1	-3.546577528	3.951046088	-0.897630007	0.369382836	NA
Glycyrrhiza_uralensis_Fisch0233340.1	-3.546577528	3.951046088	-0.897630007	0.369382836	NA
Glycyrrhiza_uralensis_Fisch0180380.1	-3.546577528	3.951046088	-0.897630007	0.369382836	NA
Glycyrrhiza_uralensis_Fisch0276860.1	-3.546577528	3.951046088	-0.897630007	0.369382836	NA
Glycyrrhiza_uralensis_Fisch0034130.1	-3.546577528	3.951046088	-0.897630007	0.369382836	NA
Glycyrrhiza_uralensis_Fisch0245560.1	-3.546577528	3.951046088	-0.897630007	0.369382836	NA
Glycyrrhiza_uralensis_Fisch0188730.1	-3.546577528	3.951046088	-0.897630007	0.369382836	NA
Glycyrrhiza_uralensis_Fisch0193020.1	-3.546577528	3.951046088	-0.897630007	0.369382836	NA
Glycyrrhiza_uralensis_Fisch0214880.1	-3.543076866	3.951153061	-0.89671972	0.369868495	NA
Glycyrrhiza_uralensis_Fisch0053650.1	-3.532767116	3.9514696	-0.894038794	0.371301135	NA
Glycyrrhiza_uralensis_Fisch0026760.1	-3.523069257	1.778985094	-1.980381549	0.047660672	0.521890596
Glycyrrhiza_uralensis_Fisch0247670.1	-3.522389996	3.951790475	-0.891340272	0.37274665	NA
Glycyrrhiza_uralensis_Fisch0028350.1	-3.522389996	3.951790475	-0.891340272	0.37274665	NA
Glycyrrhiza_uralensis_Fisch0090240.1	-3.522389996	3.951790475	-0.891340272	0.37274665	NA
Glycyrrhiza_uralensis_Fisch0116260.1	-3.522389996	3.951790475	-0.891340272	0.37274665	NA
Glycyrrhiza_uralensis_Fisch0248470.1	-3.522389996	3.951790475	-0.891340272	0.37274665	NA
Glycyrrhiza_uralensis_Fisch0231930.1	-3.522389996	3.951790475	-0.891340272	0.37274665	NA
Glycyrrhiza_uralensis_Fisch0264430.1	-3.522389996	3.951790475	-0.891340272	0.37274665	NA
Glycyrrhiza_uralensis_Fisch0045420.1	-3.522389996	3.951790475	-0.891340272	0.37274665	NA
Glycyrrhiza_uralensis_Fisch0111590.1	-3.522389996	3.951790475	-0.891340272	0.37274665	NA
Glycyrrhiza_uralensis_Fisch0	-3.522389996	3.951790	-0.891340	0.372746	NA

065980.1		475	272	65	
Glycyrrhiza_uralensis_Fisch0155590.1	-3.521113348	1.388463504	-2.535978322	0.011213368	0.264170564
Glycyrrhiza_uralensis_Fisch0091100.1	-3.489319528	3.492062361	-0.999214552	0.317690768	0.884229508
Glycyrrhiza_uralensis_Fisch0101450.1	-3.450376172	3.346700161	-1.030978578	0.302550866	NA
Glycyrrhiza_uralensis_Fisch0286890.1	-3.448161347	3.33859509	-1.032818073	0.301689044	NA
Glycyrrhiza_uralensis_Fisch0114390.1	-3.447370531	1.857720261	-1.855699484	0.063496404	0.578923656
Glycyrrhiza_uralensis_Fisch0232460.1	-3.432949815	2.271079651	-1.511593754	0.130637242	0.725528013
Glycyrrhiza_uralensis_Fisch0083990.1	-3.363614076	3.031554458	-1.109534439	0.267199696	0.854585363
Glycyrrhiza_uralensis_Fisch0129340.1	-3.321658039	3.933395191	-0.844476051	0.39840344	NA
Glycyrrhiza_uralensis_Fisch0170950.1	-3.320328259	3.958515346	-0.838781202	0.401592101	NA
Glycyrrhiza_uralensis_Fisch0089070.1	-3.320328259	3.958515346	-0.838781202	0.401592101	NA
Glycyrrhiza_uralensis_Fisch0239960.1	-3.320328259	3.958515346	-0.838781202	0.401592101	NA
Glycyrrhiza_uralensis_Fisch0001470.1	-3.320328259	3.958515346	-0.838781202	0.401592101	NA
Glycyrrhiza_uralensis_Fisch0180400.1	-3.320328259	3.958515346	-0.838781202	0.401592101	NA
Glycyrrhiza_uralensis_Fisch0117990.1	-3.320328259	3.958515346	-0.838781202	0.401592101	NA
Glycyrrhiza_uralensis_Fisch0121610.1	-3.320328259	3.958515346	-0.838781202	0.401592101	NA
Glycyrrhiza_uralensis_Fisch0154030.1	-3.320328259	3.958515346	-0.838781202	0.401592101	NA
Glycyrrhiza_uralensis_Fisch0113710.1	-3.305733838	3.959038037	-0.834984106	0.403726657	NA
Glycyrrhiza_uralensis_Fisch0032900.1	-3.296322468	3.959377878	-0.832535456	0.405106773	NA
Glycyrrhiza_uralensis_Fisch0032870.1	-3.29366562	3.959474212	-0.83184419	0.405496897	NA
Glycyrrhiza_uralensis_Fisch0177510.1	-3.283591063	3.959841099	-0.829222936	0.406978269	NA
Glycyrrhiza_uralensis_Fisch0181490.1	-3.283591063	3.959841099	-0.829222936	0.406978269	NA
Glycyrrhiza_uralensis_Fisch0	-3.283591063	3.959841	-0.829222	0.406978	NA

044990.1		099	936	269	
Glycyrrhiza_uralensis_Fisch0203960.1	-3.283591063	3.959841099	-0.829222936	0.406978269	NA
Glycyrrhiza_uralensis_Fisch0000350.1	-3.283591063	3.959841099	-0.829222936	0.406978269	NA
Glycyrrhiza_uralensis_Fisch0167260.1	-3.283591063	3.959841099	-0.829222936	0.406978269	NA
Glycyrrhiza_uralensis_Fisch0279310.1	-3.283591063	3.959841099	-0.829222936	0.406978269	NA
Glycyrrhiza_uralensis_Fisch0246220.1	-3.283591063	3.959841099	-0.829222936	0.406978269	NA
Glycyrrhiza_uralensis_Fisch0221970.1	-3.283591063	3.959841099	-0.829222936	0.406978269	NA
Glycyrrhiza_uralensis_Fisch0273090.1	-3.278827869	3.960015443	-0.827983607	0.407679784	NA
Glycyrrhiza_uralensis_Fisch0231050.1	-3.274050441	3.96019088	-0.826740564	0.408384125	NA
Glycyrrhiza_uralensis_Fisch0278410.1	-3.259634308	3.960723756	-0.822989562	0.410513929	NA
Glycyrrhiza_uralensis_Fisch0228250.1	-3.259634308	3.960723756	-0.822989562	0.410513929	NA
Glycyrrhiza_uralensis_Fisch0281010.1	-3.259634308	3.960723756	-0.822989562	0.410513929	NA
Glycyrrhiza_uralensis_Fisch0285290.1	-3.259634308	3.960723756	-0.822989562	0.410513929	NA
Glycyrrhiza_uralensis_Fisch0229330.1	-3.259634308	3.960723756	-0.822989562	0.410513929	NA
Glycyrrhiza_uralensis_Fisch0223750.1	-3.259634308	3.960723756	-0.822989562	0.410513929	NA
Glycyrrhiza_uralensis_Fisch0029290.1	-3.259634308	3.960723756	-0.822989562	0.410513929	NA
Glycyrrhiza_uralensis_Fisch0138200.1	-3.223972317	1.173328915	-2.747714025	0.006001233	0.194366451
Glycyrrhiza_uralensis_Fisch0199870.1	-3.219839972	2.491825169	-1.292161269	0.196301302	0.80474948
Glycyrrhiza_uralensis_Fisch0183240.1	-3.180425346	3.664388391	-0.867928016	0.385433741	0.90705809
Glycyrrhiza_uralensis_Fisch0028060.1	-3.160366538	3.492218049	-0.904974001	0.365479161	0.900049459
Glycyrrhiza_uralensis_Fisch0136120.1	-3.149627389	1.327384386	-2.372807322	0.01765347	0.325724612
Glycyrrhiza_uralensis_Fisch0045250.1	-3.133108022	2.743455997	-1.142029624	0.253441706	NA
Glycyrrhiza_uralensis_Fisch0	-3.101484332	2.665609	-1.163518	0.244619	0.839393

234290.1		205	015	393	558
Glycyrrhiza_uralensis_Fisch0060330.1	-3.080213846	1.494506933	-2.061023457	0.039300802	0.481900311
Glycyrrhiza_uralensis_Fisch0259540.1	-3.079688048	0.968439743	-3.180051284	0.00147249	0.086657291
Glycyrrhiza_uralensis_Fisch0118500.1	-3.077664335	1.948862749	-1.579210407	0.114287804	0.702225065
Glycyrrhiza_uralensis_Fisch0285940.1	-3.07597084	2.099836537	-1.464862043	NA	NA
Glycyrrhiza_uralensis_Fisch0251960.1	-3.060946999	0.852836764	-3.589135846	0.000331776	0.031317046
Glycyrrhiza_uralensis_Fisch0134540.1	-3.045306661	2.544872094	-1.196644291	0.231445229	0.828030507
Glycyrrhiza_uralensis_Fisch0140710.1	-3.018537674	3.596776696	-0.839234106	0.401337952	NA
Glycyrrhiza_uralensis_Fisch0031420.1	-3.016010593	1.807195148	-1.668890378	0.095139107	0.669003302
Glycyrrhiza_uralensis_Fisch0286820.1	-2.9979603	3.971366989	-0.754893795	0.450312709	NA
Glycyrrhiza_uralensis_Fisch0275790.1	-2.9979603	3.971366989	-0.754893795	0.450312709	NA
Glycyrrhiza_uralensis_Fisch0112370.1	-2.9979603	3.971366989	-0.754893795	0.450312709	NA
Glycyrrhiza_uralensis_Fisch0035940.1	-2.9979603	3.971366989	-0.754893795	0.450312709	NA
Glycyrrhiza_uralensis_Fisch0098600.1	-2.9979603	3.971366989	-0.754893795	0.450312709	NA
Glycyrrhiza_uralensis_Fisch0060750.1	-2.9979603	3.971366989	-0.754893795	0.450312709	NA
Glycyrrhiza_uralensis_Fisch0051910.1	-2.9979603	3.971366989	-0.754893795	0.450312709	NA
Glycyrrhiza_uralensis_Fisch0220540.1	-2.9979603	3.971366989	-0.754893795	0.450312709	NA
Glycyrrhiza_uralensis_Fisch0183810.1	-2.9979603	3.971366989	-0.754893795	0.450312709	NA
Glycyrrhiza_uralensis_Fisch0250700.1	-2.9979603	3.971366989	-0.754893795	0.450312709	NA
Glycyrrhiza_uralensis_Fisch0203920.1	-2.9979603	3.971366989	-0.754893795	0.450312709	NA
Glycyrrhiza_uralensis_Fisch0270190.1	-2.9979603	3.971366989	-0.754893795	0.450312709	NA
Glycyrrhiza_uralensis_Fisch0053610.1	-2.9979603	3.971366989	-0.754893795	0.450312709	NA
Glycyrrhiza_uralensis_Fisch0	-2.9979603	3.971366	-0.754893	0.450312	NA

267370.1		989	795	709	
Glycyrrhiza_uralensis_Fisch0218970.1	-2.9979603	3.971366989	-0.754893795	0.450312709	NA
Glycyrrhiza_uralensis_Fisch0166140.1	-2.9979603	3.971366989	-0.754893795	0.450312709	NA
Glycyrrhiza_uralensis_Fisch0137600.1	-2.9979603	3.971366989	-0.754893795	0.450312709	NA
Glycyrrhiza_uralensis_Fisch0170200.1	-2.9979603	3.971366989	-0.754893795	0.450312709	NA
Glycyrrhiza_uralensis_Fisch0182800.1	-2.9979603	3.971366989	-0.754893795	0.450312709	NA
Glycyrrhiza_uralensis_Fisch0024880.1	-2.9979603	3.971366989	-0.754893795	0.450312709	NA
Glycyrrhiza_uralensis_Fisch0152850.1	-2.9979603	3.971366989	-0.754893795	0.450312709	NA
Glycyrrhiza_uralensis_Fisch0242040.1	-2.9979603	3.971366989	-0.754893795	0.450312709	NA
Glycyrrhiza_uralensis_Fisch0132030.1	-2.9979603	3.971366989	-0.754893795	0.450312709	NA
Glycyrrhiza_uralensis_Fisch0086370.1	-2.9979603	3.971366989	-0.754893795	0.450312709	NA
Glycyrrhiza_uralensis_Fisch0250680.1	-2.9979603	3.971366989	-0.754893795	0.450312709	NA
Glycyrrhiza_uralensis_Fisch0149260.1	-2.9979603	3.971366989	-0.754893795	0.450312709	NA
Glycyrrhiza_uralensis_Fisch0266650.1	-2.9979603	3.971366989	-0.754893795	0.450312709	NA
Glycyrrhiza_uralensis_Fisch0180610.1	-2.9979603	3.971366989	-0.754893795	0.450312709	NA
Glycyrrhiza_uralensis_Fisch0027060.1	-2.9979603	3.971366989	-0.754893795	0.450312709	NA
Glycyrrhiza_uralensis_Fisch0151820.1	-2.9979603	3.971366989	-0.754893795	0.450312709	NA
Glycyrrhiza_uralensis_Fisch0278920.1	-2.9979603	3.971366989	-0.754893795	0.450312709	NA
Glycyrrhiza_uralensis_Fisch0086960.1	-2.9979603	3.971366989	-0.754893795	0.450312709	NA
Glycyrrhiza_uralensis_Fisch0153180.1	-2.9979603	3.971366989	-0.754893795	0.450312709	NA
Glycyrrhiza_uralensis_Fisch0127060.1	-2.9979603	3.971366989	-0.754893795	0.450312709	NA
Glycyrrhiza_uralensis_Fisch0171360.1	-2.9979603	3.971366989	-0.754893795	0.450312709	NA
Glycyrrhiza_uralensis_Fisch0	-2.9979603	3.971366	-0.754893	0.450312	NA

054050.1		989	795	709	
Glycyrrhiza_uralensis_Fisch0163940.1	-2.9979603	3.971366989	-0.754893795	0.450312709	NA
Glycyrrhiza_uralensis_Fisch0251560.1	-2.9979603	3.971366989	-0.754893795	0.450312709	NA
Glycyrrhiza_uralensis_Fisch0179380.1	-2.9979603	3.971366989	-0.754893795	0.450312709	NA
Glycyrrhiza_uralensis_Fisch0107370.1	-2.9979603	3.971366989	-0.754893795	0.450312709	NA
Glycyrrhiza_uralensis_Fisch0156260.1	-2.990746205	3.622314651	-0.825645062	0.409005465	NA
Glycyrrhiza_uralensis_Fisch0139470.1	-2.979943922	3.972172104	-0.750205138	0.453131165	NA
Glycyrrhiza_uralensis_Fisch0091960.1	-2.977408125	3.121357126	-0.953882559	0.340143093	NA
Glycyrrhiza_uralensis_Fisch0051490.1	-2.976190504	2.734109869	-1.088540932	0.276356377	0.859692133
Glycyrrhiza_uralensis_Fisch0280570.1	-2.970862832	3.972581684	-0.747841849	0.454555556	NA
Glycyrrhiza_uralensis_Fisch0010780.1	-2.961731735	3.972996083	-0.745465556	0.455990326	NA
Glycyrrhiza_uralensis_Fisch0101310.1	-2.961731735	3.972996083	-0.745465556	0.455990326	NA
Glycyrrhiza_uralensis_Fisch0071200.1	-2.961731735	3.972996083	-0.745465556	0.455990326	NA
Glycyrrhiza_uralensis_Fisch0225900.1	-2.961731735	3.972996083	-0.745465556	0.455990326	NA
Glycyrrhiza_uralensis_Fisch0251520.1	-2.961731735	3.972996083	-0.745465556	0.455990326	NA
Glycyrrhiza_uralensis_Fisch0258670.1	-2.961731735	3.972996083	-0.745465556	0.455990326	NA
Glycyrrhiza_uralensis_Fisch0004690.1	-2.961731735	3.972996083	-0.745465556	0.455990326	NA
Glycyrrhiza_uralensis_Fisch0068810.1	-2.961731735	3.972996083	-0.745465556	0.455990326	NA
Glycyrrhiza_uralensis_Fisch0010000.1	-2.961731735	3.972996083	-0.745465556	0.455990326	NA
Glycyrrhiza_uralensis_Fisch0249190.1	-2.961731735	3.972996083	-0.745465556	0.455990326	NA
Glycyrrhiza_uralensis_Fisch0060350.1	-2.961731735	3.972996083	-0.745465556	0.455990326	NA
Glycyrrhiza_uralensis_Fisch0154580.1	-2.961731735	3.972996083	-0.745465556	0.455990326	NA
Glycyrrhiza_uralensis_Fisch0	-2.961731735	3.972996	-0.745465	0.455990	NA

152540.1		083	556	326	
Glycyrrhiza_uralensis_Fisch0243310.1	-2.961731735	3.972996083	-0.745465556	0.455990326	NA
Glycyrrhiza_uralensis_Fisch0250760.1	-2.961731735	3.972996083	-0.745465556	0.455990326	NA
Glycyrrhiza_uralensis_Fisch0004260.1	-2.961731735	3.972996083	-0.745465556	0.455990326	NA
Glycyrrhiza_uralensis_Fisch0186710.1	-2.961731735	3.972996083	-0.745465556	0.455990326	NA
Glycyrrhiza_uralensis_Fisch0108600.1	-2.961731735	3.972996083	-0.745465556	0.455990326	NA
Glycyrrhiza_uralensis_Fisch0030940.1	-2.961731735	3.972996083	-0.745465556	0.455990326	NA
Glycyrrhiza_uralensis_Fisch0134680.1	-2.961731735	3.972996083	-0.745465556	0.455990326	NA
Glycyrrhiza_uralensis_Fisch0190860.1	-2.961731735	3.972996083	-0.745465556	0.455990326	NA
Glycyrrhiza_uralensis_Fisch0063810.1	-2.961731735	3.972996083	-0.745465556	0.455990326	NA
Glycyrrhiza_uralensis_Fisch0155810.1	-2.961731735	3.972996083	-0.745465556	0.455990326	NA
Glycyrrhiza_uralensis_Fisch0070470.1	-2.961731735	3.972996083	-0.745465556	0.455990326	NA
Glycyrrhiza_uralensis_Fisch0029980.1	-2.961731735	3.972996083	-0.745465556	0.455990326	NA
Glycyrrhiza_uralensis_Fisch0150570.1	-2.961731735	3.972996083	-0.745465556	0.455990326	NA
Glycyrrhiza_uralensis_Fisch0238510.1	-2.961731735	3.972996083	-0.745465556	0.455990326	NA
Glycyrrhiza_uralensis_Fisch0138220.1	-2.961731735	3.972996083	-0.745465556	0.455990326	NA
Glycyrrhiza_uralensis_Fisch0241320.1	-2.961731735	3.972996083	-0.745465556	0.455990326	NA
Glycyrrhiza_uralensis_Fisch0058530.1	-2.961731735	3.972996083	-0.745465556	0.455990326	NA
Glycyrrhiza_uralensis_Fisch0277830.1	-2.961731735	3.972996083	-0.745465556	0.455990326	NA
Glycyrrhiza_uralensis_Fisch0067030.1	-2.961731735	3.972996083	-0.745465556	0.455990326	NA
Glycyrrhiza_uralensis_Fisch0236080.1	-2.961731735	3.972996083	-0.745465556	0.455990326	NA
Glycyrrhiza_uralensis_Fisch0244860.1	-2.961731735	3.972996083	-0.745465556	0.455990326	NA
Glycyrrhiza_uralensis_Fisch0	-2.961731735	3.972996	-0.745465	0.455990	NA

275020.1		083	556	326	
Glycyrrhiza_uralensis_Fisch0061170.1	-2.961731735	3.972996083	-0.745465556	0.455990326	NA
Glycyrrhiza_uralensis_Fisch0197270.1	-2.961731735	3.972996083	-0.745465556	0.455990326	NA
Glycyrrhiza_uralensis_Fisch0263490.1	-2.961731735	3.972996083	-0.745465556	0.455990326	NA
Glycyrrhiza_uralensis_Fisch0244030.1	-2.961731735	3.972996083	-0.745465556	0.455990326	NA
Glycyrrhiza_uralensis_Fisch0190720.1	-2.950328885	1.157274274	-2.549377403	0.010791545	0.259492986
Glycyrrhiza_uralensis_Fisch0210650.1	-2.949963374	3.973533989	-0.742402955	0.45784323	NA
Glycyrrhiza_uralensis_Fisch0005330.1	-2.945258733	1.961088275	-1.501849137	0.133136075	0.729217799
Glycyrrhiza_uralensis_Fisch0019750.1	-2.93864391	3.873644383	-0.758625114	0.448076845	NA
Glycyrrhiza_uralensis_Fisch0009470.1	-2.938111829	3.974080076	-0.739318728	0.459713479	NA
Glycyrrhiza_uralensis_Fisch0082170.1	-2.938111829	3.974080076	-0.739318728	0.459713479	NA
Glycyrrhiza_uralensis_Fisch0071070.1	-2.938111829	3.974080076	-0.739318728	0.459713479	NA
Glycyrrhiza_uralensis_Fisch0155660.1	-2.938111829	3.974080076	-0.739318728	0.459713479	NA
Glycyrrhiza_uralensis_Fisch0230900.1	-2.938111829	3.974080076	-0.739318728	0.459713479	NA
Glycyrrhiza_uralensis_Fisch0113480.1	-2.938111829	3.974080076	-0.739318728	0.459713479	NA
Glycyrrhiza_uralensis_Fisch0237450.1	-2.938111829	3.974080076	-0.739318728	0.459713479	NA
Glycyrrhiza_uralensis_Fisch0140560.1	-2.938111829	3.974080076	-0.739318728	0.459713479	NA
Glycyrrhiza_uralensis_Fisch0127200.1	-2.938111829	3.974080076	-0.739318728	0.459713479	NA
Glycyrrhiza_uralensis_Fisch0232580.1	-2.938111829	3.974080076	-0.739318728	0.459713479	NA
Glycyrrhiza_uralensis_Fisch0181550.1	-2.938111829	3.974080076	-0.739318728	0.459713479	NA
Glycyrrhiza_uralensis_Fisch0112360.1	-2.938111829	3.974080076	-0.739318728	0.459713479	NA
Glycyrrhiza_uralensis_Fisch0203340.1	-2.938111829	3.974080076	-0.739318728	0.459713479	NA
Glycyrrhiza_uralensis_Fisch0	-2.938111829	3.974080	-0.739318	0.459713	NA

084570.1		076	728	479	
Glycyrrhiza_uralensis_Fisch0206980.1	-2.938111829	3.974080076	-0.739318728	0.459713479	NA
Glycyrrhiza_uralensis_Fisch0169260.1	-2.938111829	3.974080076	-0.739318728	0.459713479	NA
Glycyrrhiza_uralensis_Fisch0231970.1	-2.938111829	3.974080076	-0.739318728	0.459713479	NA
Glycyrrhiza_uralensis_Fisch0112020.1	-2.938111829	3.974080076	-0.739318728	0.459713479	NA
Glycyrrhiza_uralensis_Fisch0256150.1	-2.938111829	3.974080076	-0.739318728	0.459713479	NA
Glycyrrhiza_uralensis_Fisch0021530.1	-2.938111829	3.974080076	-0.739318728	0.459713479	NA
Glycyrrhiza_uralensis_Fisch0204880.1	-2.938111829	3.974080076	-0.739318728	0.459713479	NA
Glycyrrhiza_uralensis_Fisch0052750.1	-2.938111829	3.974080076	-0.739318728	0.459713479	NA
Glycyrrhiza_uralensis_Fisch0050690.1	-2.938111829	3.974080076	-0.739318728	0.459713479	NA
Glycyrrhiza_uralensis_Fisch0060140.1	-2.938111829	3.974080076	-0.739318728	0.459713479	NA
Glycyrrhiza_uralensis_Fisch0046520.1	-2.938111829	3.974080076	-0.739318728	0.459713479	NA
Glycyrrhiza_uralensis_Fisch0082150.1	-2.938111829	3.974080076	-0.739318728	0.459713479	NA
Glycyrrhiza_uralensis_Fisch0006460.1	-2.938111829	3.974080076	-0.739318728	0.459713479	NA
Glycyrrhiza_uralensis_Fisch0154920.1	-2.938111829	3.974080076	-0.739318728	0.459713479	NA
Glycyrrhiza_uralensis_Fisch0180000.1	-2.938111829	3.974080076	-0.739318728	0.459713479	NA
Glycyrrhiza_uralensis_Fisch0054900.1	-2.938111829	3.974080076	-0.739318728	0.459713479	NA
Glycyrrhiza_uralensis_Fisch0048240.1	-2.938111829	3.974080076	-0.739318728	0.459713479	NA
Glycyrrhiza_uralensis_Fisch0013160.1	-2.938111829	3.974080076	-0.739318728	0.459713479	NA
Glycyrrhiza_uralensis_Fisch0018330.1	-2.938111829	3.974080076	-0.739318728	0.459713479	NA
Glycyrrhiza_uralensis_Fisch0039530.1	-2.938111829	3.974080076	-0.739318728	0.459713479	NA
Glycyrrhiza_uralensis_Fisch0162170.1	-2.927994101	1.357175273	-2.157417807	0.030973125	0.430680825
Glycyrrhiza_uralensis_Fisch0	-2.919683527	1.173484	-2.488045	0.012844	0.280301

178440.1		715	638	725	476
Glycyrrhiza_uralensis_Fisch0009380.1	-2.915538584	1.276783595	-2.283502542	0.022400783	0.36555041
Glycyrrhiza_uralensis_Fisch0183180.1	-2.909506629	2.812384933	-1.034533572	0.300886791	0.872869614
Glycyrrhiza_uralensis_Fisch0057530.1	-2.908331415	1.424383852	-2.041817176	0.04116967	0.492766759
Glycyrrhiza_uralensis_Fisch0203610.1	-2.889398509	2.451870375	-1.178446682	0.238618577	0.834895529
Glycyrrhiza_uralensis_Fisch0140370.1	-2.880912378	1.251260618	-2.302407936	0.021312178	0.357517972
Glycyrrhiza_uralensis_Fisch0152200.1	-2.864322389	1.655905001	-1.729762509	0.083672715	0.641860957
Glycyrrhiza_uralensis_Fisch0101930.1	-2.8631497	1.449940647	-1.97466683	0.04830597	0.524360715
Glycyrrhiza_uralensis_Fisch0109760.1	-2.856322007	2.653543457	-1.076418025	0.281740345	0.86117142
Glycyrrhiza_uralensis_Fisch0271950.1	-2.843752357	2.087473026	-1.362294181	0.173105063	0.780561797
Glycyrrhiza_uralensis_Fisch0174210.1	-2.831588652	1.335350744	-2.120483074	0.033965327	0.451283183
Glycyrrhiza_uralensis_Fisch0251940.1	-2.831276346	1.905171125	-1.486100807	0.137252459	0.734882382
Glycyrrhiza_uralensis_Fisch0208410.1	-2.823363576	3.835004399	-0.736208693	0.461603703	0.924721482
Glycyrrhiza_uralensis_Fisch0170290.1	-2.817884642	3.752395137	-0.750956266	0.452678976	NA
Glycyrrhiza_uralensis_Fisch0097980.1	-2.817736532	2.830393042	-0.995528356	0.31947936	0.885321694
Glycyrrhiza_uralensis_Fisch0197190.1	-2.809883133	2.504396453	-1.12198016	0.261870876	0.850983121
Glycyrrhiza_uralensis_Fisch0101640.1	-2.795565662	1.913398949	-1.461046931	0.144002562	0.744212491
Glycyrrhiza_uralensis_Fisch0033470.1	-2.772974714	0.809337517	-3.426227817	0.000612027	0.048458834
Glycyrrhiza_uralensis_Fisch0037630.1	-2.734986535	2.805799642	-0.97476188	0.32967839	0.887225647
Glycyrrhiza_uralensis_Fisch0017880.1	-2.724339395	2.781946924	-0.97929237	0.32743554	0.887225647
Glycyrrhiza_uralensis_Fisch0110190.1	-2.717882136	2.273335768	-1.195548046	0.231872976	0.828314416
Glycyrrhiza_uralensis_Fisch0060820.1	-2.70869462	2.063680844	-1.312555005	0.18933294	0.797474632
Glycyrrhiza_uralensis_Fisch0	-2.691824509	0.803809	-3.348832	0.000811	0.058308

270980.1		832	523	528	917
Glycyrrhiza_uralensis_Fisch0110340.1	-2.676799893	1.736577127	-1.541422981	0.123213874	0.715913454
Glycyrrhiza_uralensis_Fisch0029130.1	-2.667635965	2.007935358	-1.328546735	0.183997555	0.792181538
Glycyrrhiza_uralensis_Fisch0258520.1	-2.66649569	1.158859055	-2.300966351	0.021393533	0.357777081
Glycyrrhiza_uralensis_Fisch0095160.1	-2.662864298	2.582111393	-1.031273982	0.302412356	0.87347594
Glycyrrhiza_uralensis_Fisch0237520.1	-2.662025382	1.246834849	-2.135026451	0.03275885	0.442980624
Glycyrrhiza_uralensis_Fisch0032510.1	-2.642623762	1.042900079	-2.53391846	0.011279497	0.264575469
Glycyrrhiza_uralensis_Fisch0286340.1	-2.613430639	1.579594485	-1.654494659	0.098027048	0.676853424
Glycyrrhiza_uralensis_Fisch0052840.1	-2.609571596	3.872342351	-0.673900022	0.500374878	NA
Glycyrrhiza_uralensis_Fisch0108390.1	-2.608941258	0.906965897	-2.87655938	0.004020365	0.155176783
Glycyrrhiza_uralensis_Fisch0245530.1	-2.600710008	2.775496237	-0.937025233	0.348745579	0.896157369
Glycyrrhiza_uralensis_Fisch0027550.1	-2.598736983	3.073493046	-0.845532085	0.397813826	0.910230427
Glycyrrhiza_uralensis_Fisch0235040.1	-2.583543455	2.575171583	-1.003250996	0.315739774	0.882780387
Glycyrrhiza_uralensis_Fisch0051520.1	-2.582212331	3.99271022	-0.646731716	0.517805593	NA
Glycyrrhiza_uralensis_Fisch0257370.1	-2.582212331	3.99271022	-0.646731716	0.517805593	NA
Glycyrrhiza_uralensis_Fisch0092470.1	-2.582212331	3.99271022	-0.646731716	0.517805593	NA
Glycyrrhiza_uralensis_Fisch0093570.1	-2.582212331	3.99271022	-0.646731716	0.517805593	NA
Glycyrrhiza_uralensis_Fisch0057360.1	-2.582212331	3.99271022	-0.646731716	0.517805593	NA
Glycyrrhiza_uralensis_Fisch0181560.1	-2.582212331	3.99271022	-0.646731716	0.517805593	NA
Glycyrrhiza_uralensis_Fisch0234690.1	-2.582212331	3.99271022	-0.646731716	0.517805593	NA
Glycyrrhiza_uralensis_Fisch0277780.1	-2.582212331	3.99271022	-0.646731716	0.517805593	NA
Glycyrrhiza_uralensis_Fisch0032830.1	-2.582212331	3.99271022	-0.646731716	0.517805593	NA
Glycyrrhiza_uralensis_Fisch0	-2.582212331	3.992710	-0.646731	0.517805	NA

280190.1		22	716	593	
Glycyrrhiza_uralensis_Fisch0 004650.1	-2.582212331	3.992710 22	-0.646731 716	0.517805 593	NA
Glycyrrhiza_uralensis_Fisch0 106530.1	-2.582212331	3.992710 22	-0.646731 716	0.517805 593	NA
Glycyrrhiza_uralensis_Fisch0 273810.1	-2.582212331	3.992710 22	-0.646731 716	0.517805 593	NA
Glycyrrhiza_uralensis_Fisch0 238400.1	-2.582212331	3.992710 22	-0.646731 716	0.517805 593	NA
Glycyrrhiza_uralensis_Fisch0 231680.1	-2.582212331	3.992710 22	-0.646731 716	0.517805 593	NA
Glycyrrhiza_uralensis_Fisch0 177070.1	-2.582212331	3.992710 22	-0.646731 716	0.517805 593	NA
Glycyrrhiza_uralensis_Fisch0 148000.1	-2.582212331	3.992710 22	-0.646731 716	0.517805 593	NA
Glycyrrhiza_uralensis_Fisch0 135050.1	-2.582212331	3.992710 22	-0.646731 716	0.517805 593	NA
Glycyrrhiza_uralensis_Fisch0 188110.1	-2.582212331	3.992710 22	-0.646731 716	0.517805 593	NA
Glycyrrhiza_uralensis_Fisch0 240490.1	-2.582212331	3.992710 22	-0.646731 716	0.517805 593	NA
Glycyrrhiza_uralensis_Fisch0 285650.1	-2.582212331	3.992710 22	-0.646731 716	0.517805 593	NA
Glycyrrhiza_uralensis_Fisch0 180030.1	-2.582212331	3.992710 22	-0.646731 716	0.517805 593	NA
Glycyrrhiza_uralensis_Fisch0 071100.1	-2.573353487	1.956423 488	-1.315335 613	0.188397 134	0.797474 632
Glycyrrhiza_uralensis_Fisch0 120960.1	-2.563750865	2.239151 234	-1.144965 479	0.252223 458	0.845741 676
Glycyrrhiza_uralensis_Fisch0 286310.1	-2.558684769	3.994107 832	-0.640614 845	0.521772 952	NA
Glycyrrhiza_uralensis_Fisch0 022160.1	-2.550860979	2.819540 792	-0.904707 953	0.365620 127	0.900212 403
Glycyrrhiza_uralensis_Fisch0 030620.1	-2.54680225	3.994822 212	-0.637525 806	0.523782 406	NA
Glycyrrhiza_uralensis_Fisch0 206400.1	-2.54680225	3.994822 212	-0.637525 806	0.523782 406	NA
Glycyrrhiza_uralensis_Fisch0 226030.1	-2.54680225	3.994822 212	-0.637525 806	0.523782 406	NA
Glycyrrhiza_uralensis_Fisch0 238060.1	-2.54680225	3.994822 212	-0.637525 806	0.523782 406	NA
Glycyrrhiza_uralensis_Fisch0 050200.1	-2.54680225	3.994822 212	-0.637525 806	0.523782 406	NA
Glycyrrhiza_uralensis_Fisch0	-2.54680225	3.994822	-0.637525	0.523782	NA

013310.1		212	806	406	
Glycyrrhiza_uralensis_Fisch0033320.1	-2.54680225	3.994822212	-0.637525806	0.523782406	NA
Glycyrrhiza_uralensis_Fisch0114420.1	-2.54680225	3.994822212	-0.637525806	0.523782406	NA
Glycyrrhiza_uralensis_Fisch0186320.1	-2.54680225	3.994822212	-0.637525806	0.523782406	NA
Glycyrrhiza_uralensis_Fisch0244340.1	-2.54680225	3.994822212	-0.637525806	0.523782406	NA
Glycyrrhiza_uralensis_Fisch0039070.1	-2.54680225	3.994822212	-0.637525806	0.523782406	NA
Glycyrrhiza_uralensis_Fisch0068220.1	-2.54680225	3.994822212	-0.637525806	0.523782406	NA
Glycyrrhiza_uralensis_Fisch0245470.1	-2.54680225	3.994822212	-0.637525806	0.523782406	NA
Glycyrrhiza_uralensis_Fisch0252300.1	-2.54680225	3.994822212	-0.637525806	0.523782406	NA
Glycyrrhiza_uralensis_Fisch0109850.1	-2.54680225	3.994822212	-0.637525806	0.523782406	NA
Glycyrrhiza_uralensis_Fisch0278840.1	-2.54680225	3.994822212	-0.637525806	0.523782406	NA
Glycyrrhiza_uralensis_Fisch0273550.1	-2.54680225	3.994822212	-0.637525806	0.523782406	NA
Glycyrrhiza_uralensis_Fisch0012190.1	-2.54680225	3.994822212	-0.637525806	0.523782406	NA
Glycyrrhiza_uralensis_Fisch0255920.1	-2.54680225	3.994822212	-0.637525806	0.523782406	NA
Glycyrrhiza_uralensis_Fisch0129090.1	-2.54680225	3.994822212	-0.637525806	0.523782406	NA
Glycyrrhiza_uralensis_Fisch0048140.1	-2.54680225	3.994822212	-0.637525806	0.523782406	NA
Glycyrrhiza_uralensis_Fisch0052710.1	-2.54680225	3.994822212	-0.637525806	0.523782406	NA
Glycyrrhiza_uralensis_Fisch0179400.1	-2.54590656	2.068714951	-1.230670547	0.218446108	0.817601901
Glycyrrhiza_uralensis_Fisch0255120.1	-2.531449873	3.995753763	-0.633535003	0.52638433	NA
Glycyrrhiza_uralensis_Fisch0053400.1	-2.523723369	3.996226272	-0.631526645	0.527696233	NA
Glycyrrhiza_uralensis_Fisch0150290.1	-2.523723369	3.996226272	-0.631526645	0.527696233	NA
Glycyrrhiza_uralensis_Fisch0215770.1	-2.523723369	3.996226272	-0.631526645	0.527696233	NA
Glycyrrhiza_uralensis_Fisch0	-2.523723369	3.996226	-0.631526	0.527696	NA

279590.1		272	645	233	
Glycyrrhiza_uralensis_Fisch0 138690.1	-2.523723369	3.996226 272	-0.631526 645	0.527696 233	NA
Glycyrrhiza_uralensis_Fisch0 238350.1	-2.523723369	3.996226 272	-0.631526 645	0.527696 233	NA
Glycyrrhiza_uralensis_Fisch0 240520.1	-2.523723369	3.996226 272	-0.631526 645	0.527696 233	NA
Glycyrrhiza_uralensis_Fisch0 173450.1	-2.523723369	3.996226 272	-0.631526 645	0.527696 233	NA
Glycyrrhiza_uralensis_Fisch0 030310.1	-2.523723369	3.996226 272	-0.631526 645	0.527696 233	NA
Glycyrrhiza_uralensis_Fisch0 167930.1	-2.523723369	3.996226 272	-0.631526 645	0.527696 233	NA
Glycyrrhiza_uralensis_Fisch0 228760.1	-2.523723369	3.996226 272	-0.631526 645	0.527696 233	NA
Glycyrrhiza_uralensis_Fisch0 250280.1	-2.523723369	3.996226 272	-0.631526 645	0.527696 233	NA
Glycyrrhiza_uralensis_Fisch0 005170.1	-2.523723369	3.996226 272	-0.631526 645	0.527696 233	NA
Glycyrrhiza_uralensis_Fisch0 282920.1	-2.523723369	3.996226 272	-0.631526 645	0.527696 233	NA
Glycyrrhiza_uralensis_Fisch0 109490.1	-2.523723369	3.996226 272	-0.631526 645	0.527696 233	NA
Glycyrrhiza_uralensis_Fisch0 124610.1	-2.523723369	3.996226 272	-0.631526 645	0.527696 233	NA
Glycyrrhiza_uralensis_Fisch0 073260.1	-2.523723369	3.996226 272	-0.631526 645	0.527696 233	NA
Glycyrrhiza_uralensis_Fisch0 049570.1	-2.523723369	3.996226 272	-0.631526 645	0.527696 233	NA
Glycyrrhiza_uralensis_Fisch0 201740.1	-2.523723369	3.996226 272	-0.631526 645	0.527696 233	NA
Glycyrrhiza_uralensis_Fisch0 055070.1	-2.523723369	3.996226 272	-0.631526 645	0.527696 233	NA
Glycyrrhiza_uralensis_Fisch0 281330.1	-2.523723369	3.996226 272	-0.631526 645	0.527696 233	NA
Glycyrrhiza_uralensis_Fisch0 095230.1	-2.523723369	3.996226 272	-0.631526 645	0.527696 233	NA
Glycyrrhiza_uralensis_Fisch0 049720.1	-2.523723369	3.996226 272	-0.631526 645	0.527696 233	NA
Glycyrrhiza_uralensis_Fisch0 239920.1	-2.523723369	3.996226 272	-0.631526 645	0.527696 233	NA
Glycyrrhiza_uralensis_Fisch0 197290.1	-2.523723369	3.996226 272	-0.631526 645	0.527696 233	NA
Glycyrrhiza_uralensis_Fisch0	-2.49354771	2.925554	-0.852333	0.394029	0.910003

060000.1		782	282	139	682
Glycyrrhiza_uralensis_Fisch0214550.1	-2.487859103	0.397175356	-6.263880834	3.76E-10	5.02E-07
Glycyrrhiza_uralensis_Fisch0175110.1	-2.480696748	3.890045458	-0.637703794	0.523666515	NA
Glycyrrhiza_uralensis_Fisch0277170.1	-2.479415518	3.029484545	-0.818428178	NA	NA
Glycyrrhiza_uralensis_Fisch0063760.1	-2.475939966	2.941406632	-0.841753717	0.399925824	0.910230427
Glycyrrhiza_uralensis_Fisch0152460.1	-2.467990339	3.068921795	-0.804188084	0.421288359	NA
Glycyrrhiza_uralensis_Fisch0254180.1	-2.455765353	1.531967565	-1.603013934	0.108931578	0.695942517
Glycyrrhiza_uralensis_Fisch0266350.1	-2.452473486	1.88887422	-1.2983784	0.194157338	0.801353197
Glycyrrhiza_uralensis_Fisch0223490.1	-2.450407484	3.730978258	-0.656773456	0.511326594	0.940518961
Glycyrrhiza_uralensis_Fisch0128600.1	-2.445387757	1.405220299	-1.740216647	0.081820984	0.637381217
Glycyrrhiza_uralensis_Fisch0234970.1	-2.436257505	3.093680593	-0.78749484	0.430992248	NA
Glycyrrhiza_uralensis_Fisch0051920.1	-2.421807032	1.692249259	-1.431117206	0.152396627	0.756015418
Glycyrrhiza_uralensis_Fisch0276020.1	-2.420938927	3.839611471	-0.630516641	0.528356619	NA
Glycyrrhiza_uralensis_Fisch0159510.1	-2.419052517	3.809059511	-0.635078688	0.525377096	0.944922987
Glycyrrhiza_uralensis_Fisch0243800.1	-2.418938385	2.125733667	-1.13793107	0.255149266	0.847381568
Glycyrrhiza_uralensis_Fisch0086050.1	-2.418039743	1.896923194	-1.274716736	0.202409521	0.807557959
Glycyrrhiza_uralensis_Fisch0209180.1	-2.415017253	2.962018971	-0.815328084	0.414884537	0.912286761
Glycyrrhiza_uralensis_Fisch0166540.1	-2.406755349	2.139776293	-1.124769611	0.260686675	0.850426487
Glycyrrhiza_uralensis_Fisch0113080.1	-2.402587087	2.477509472	-0.969758991	0.332166638	0.888277533
Glycyrrhiza_uralensis_Fisch0188460.1	-2.388711799	2.806437111	-0.851154579	0.39468349	0.910080524
Glycyrrhiza_uralensis_Fisch0212540.1	-2.383263813	2.923185986	-0.815296674	0.414902512	0.912286761
Glycyrrhiza_uralensis_Fisch0235590.1	-2.382513997	1.87134192	-1.273158032	0.20296197	0.808104045
Glycyrrhiza_uralensis_Fisch0	-2.377316655	2.964558	-0.801912	0.422603	0.913852

098060.1		172	635	497	659
Glycyrrhiza_uralensis_Fisch0048250.1	-2.371346284	0.869615377	-2.72689093	0.006393417	0.199597333
Glycyrrhiza_uralensis_Fisch0159560.1	-2.365871186	2.898761889	-0.8161661	0.414405143	0.912286761
Glycyrrhiza_uralensis_Fisch0111620.1	-2.357244047	1.170511919	-2.013857364	NA	NA
Glycyrrhiza_uralensis_Fisch0024220.1	-2.35079223	3.451795193	-0.681034679	0.495849548	NA
Glycyrrhiza_uralensis_Fisch0228140.1	-2.347533919	1.356430786	-1.73066989	0.083510659	0.641469928
Glycyrrhiza_uralensis_Fisch0101330.1	-2.339469528	1.044054944	-2.240753268	0.025042062	0.386983097
Glycyrrhiza_uralensis_Fisch0084880.1	-2.337268395	2.082270377	-1.122461531	0.261666255	0.850912419
Glycyrrhiza_uralensis_Fisch0238870.1	-2.336020315	1.482900847	-1.575304458	0.115186185	0.704403324
Glycyrrhiza_uralensis_Fisch0007640.1	-2.329290861	3.1111669	-0.748687209	0.454045755	NA
Glycyrrhiza_uralensis_Fisch0231130.1	-2.327894519	3.897230346	-0.597320228	0.550293602	NA
Glycyrrhiza_uralensis_Fisch0091410.1	-2.320507981	0.356117742	-6.516125724	7.21E-11	1.16E-07
Glycyrrhiza_uralensis_Fisch0089140.1	-2.319552814	1.527357218	-1.518670804	0.128845379	0.72182017
Glycyrrhiza_uralensis_Fisch0161270.1	-2.318761144	0.982259289	-2.360640587	0.018243402	0.331111164
Glycyrrhiza_uralensis_Fisch0039930.1	-2.306949824	2.011841699	-1.146685559	0.251511601	0.845377633
Glycyrrhiza_uralensis_Fisch0270990.1	-2.301923747	0.732537613	-3.142396659	0.001675709	0.093150819
Glycyrrhiza_uralensis_Fisch0030540.1	-2.301161575	1.583577604	-1.453141021	0.146184604	0.745903116
Glycyrrhiza_uralensis_Fisch0056400.1	-2.297852695	3.960723493	-0.580159837	0.561806835	NA
Glycyrrhiza_uralensis_Fisch0056450.1	-2.297383846	0.884498327	-2.597386308	0.009393621	0.237504686
Glycyrrhiza_uralensis_Fisch0022940.1	-2.292434854	3.600162482	-0.63675872	0.524282018	0.944922987
Glycyrrhiza_uralensis_Fisch0115930.1	-2.284366356	3.73456028	-0.611682818	NA	NA
Glycyrrhiza_uralensis_Fisch0219950.1	-2.283158435	2.660032	-0.858319913	0.390715837	0.908672653
Glycyrrhiza_uralensis_Fisch0	-2.280251334	1.935582	-1.178069	0.238768	0.834895

167560.1		937	557	877	529
Glycyrrhiza_uralensis_Fisch0161460.1	-2.27685564	3.725398009	-0.611171111	NA	NA
Glycyrrhiza_uralensis_Fisch0150930.1	-2.274654449	2.466630253	-0.922170822	0.35643948	0.897807124
Glycyrrhiza_uralensis_Fisch0222640.1	-2.267433081	0.630681025	-3.595213734	0.000324125	0.030836749
Glycyrrhiza_uralensis_Fisch0094670.1	-2.264989021	2.655792052	-0.852848784	0.393743167	0.910003682
Glycyrrhiza_uralensis_Fisch0188370.1	-2.264123647	1.19324409	-1.897452219	0.057768279	0.561357482
Glycyrrhiza_uralensis_Fisch0030280.1	-2.261299149	1.332036623	-1.697625358	0.089578494	0.653414296
Glycyrrhiza_uralensis_Fisch0150630.1	-2.258680381	1.711868192	-1.319424236	NA	NA
Glycyrrhiza_uralensis_Fisch0218760.1	-2.257877298	2.996974246	-0.753385619	0.451218222	0.922710496
Glycyrrhiza_uralensis_Fisch0231270.1	-2.256255119	2.651098781	-0.851064146	0.394733721	0.910080524
Glycyrrhiza_uralensis_Fisch0015390.1	-2.251947002	2.559112296	-0.879971936	NA	NA
Glycyrrhiza_uralensis_Fisch0180640.1	-2.238699479	1.349579715	-1.658812336	0.097153614	0.67528983
Glycyrrhiza_uralensis_Fisch0105920.1	-2.235028434	2.049878016	-1.090322652	0.27557104	0.859418884
Glycyrrhiza_uralensis_Fisch0157500.1	-2.234731203	2.558146952	-0.87357421	NA	NA
Glycyrrhiza_uralensis_Fisch0027620.1	-2.234349376	0.770503002	-2.899858105	0.003733316	0.149023087
Glycyrrhiza_uralensis_Fisch0118830.1	-2.233418119	1.538633382	-1.45155964	0.146624088	0.745903116
Glycyrrhiza_uralensis_Fisch0173210.1	-2.23075563	1.335508129	-1.670342233	0.094851672	0.667957794
Glycyrrhiza_uralensis_Fisch0188520.1	-2.228029576	1.870857974	-1.190913264	0.233687637	0.830237848
Glycyrrhiza_uralensis_Fisch0241840.1	-2.224272568	3.813443292	-0.583271442	0.559710584	0.950482887
Glycyrrhiza_uralensis_Fisch0137500.1	-2.216075391	1.388367482	-1.596173506	0.110450061	0.698118389
Glycyrrhiza_uralensis_Fisch0176580.1	-2.203073857	1.421647628	-1.549662388	0.12122257	0.713403979
Glycyrrhiza_uralensis_Fisch0202690.1	-2.195519074	3.90008947	-0.562940694	0.573475269	NA
Glycyrrhiza_uralensis_Fisch0	-2.190039892	3.023682	-0.724295	0.468884	0.928510

092760.1		704	538	315	528
Glycyrrhiza_uralensis_Fisch0 052900.1	-2.18780906	3.039433 411	-0.719808 189	0.471643 102	0.928837 698
Glycyrrhiza_uralensis_Fisch0 003790.1	-2.186241819	1.310872 901	-1.667775 584	0.095360 284	0.669385 257
Glycyrrhiza_uralensis_Fisch0 012360.1	-2.180252751	1.143988 606	-1.905834 323	0.056671 704	0.555863 049
Glycyrrhiza_uralensis_Fisch0 239740.1	-2.176536632	3.730692 081	-0.583413 636	0.559614 88	0.950482 887
Glycyrrhiza_uralensis_Fisch0 254570.1	-2.174529989	1.373639 222	-1.583043 024	0.113411 658	0.701200 196
Glycyrrhiza_uralensis_Fisch0 217800.1	-2.171759657	1.675997 537	-1.295801 222	0.195043 977	0.802102 943
Glycyrrhiza_uralensis_Fisch0 065780.1	-2.169446983	1.245327 09	-1.742070 015	0.081496 194	0.636887 461
Glycyrrhiza_uralensis_Fisch0 181470.1	-2.16874829	2.673037 379	-0.811342 298	0.417169 126	0.913278 077
Glycyrrhiza_uralensis_Fisch0 064310.1	-2.166334611	1.582119 825	-1.369260 771	0.170917 776	0.779044 888
Glycyrrhiza_uralensis_Fisch0 111370.1	-2.165435109	0.904597 216	-2.393811 379	0.016674 321	0.317487 481
Glycyrrhiza_uralensis_Fisch0 246640.1	-2.162614187	2.289070 284	-0.944756 568	0.344783 17	0.894679 699
Glycyrrhiza_uralensis_Fisch0 177330.1	-2.161780556	3.814168 571	-0.566776 354	0.570866 138	0.952518 696
Glycyrrhiza_uralensis_Fisch0 220880.1	-2.158188056	0.971317 346	-2.221918 578	0.026288 809	0.396768 725
Glycyrrhiza_uralensis_Fisch0 124090.1	-2.158157796	2.309014 891	-0.934666 037	0.349960 435	0.897065 603
Glycyrrhiza_uralensis_Fisch0 011230.1	-2.154790748	2.114386 916	-1.019109 006	0.308151 219	0.877596 169
Glycyrrhiza_uralensis_Fisch0 276850.1	-2.150527941	3.579960 01	-0.600712 839	0.548031 266	NA
Glycyrrhiza_uralensis_Fisch0 006370.1	-2.1483803	2.779572	-0.772917 665	0.439571 11	NA
Glycyrrhiza_uralensis_Fisch0 217790.1	-2.13927595	1.880883 084	-1.137378 484	0.255380 098	0.847381 568
Glycyrrhiza_uralensis_Fisch0 277900.1	-2.138412844	0.867331 361	-2.465508 502	0.013681 893	0.288157 19
Glycyrrhiza_uralensis_Fisch0 040060.1	-2.136323452	3.281113 593	-0.651097 072	0.514983 827	0.941847 938
Glycyrrhiza_uralensis_Fisch0 245200.1	-2.133510322	3.901520 922	-0.546840 672	0.584488 198	NA
Glycyrrhiza_uralensis_Fisch0	-2.133441041	2.985157	-0.714682	0.474804	0.929133

150720.1		135	995	948	34
Glycyrrhiza_uralensis_Fisch0164830.1	-2.13271058	2.312410569	-0.922288892	0.356377907	0.897807124
Glycyrrhiza_uralensis_Fisch0199550.1	-2.132343982	2.268140068	-0.94012888	0.347151457	0.896081753
Glycyrrhiza_uralensis_Fisch0059160.1	-2.130994535	3.046141426	-0.699571765	0.48419478	0.93291241
Glycyrrhiza_uralensis_Fisch0020260.1	-2.130346281	2.2978314	-0.927111659	0.353868563	0.897364869
Glycyrrhiza_uralensis_Fisch0037560.1	-2.12996575	1.660410342	-1.282794798	0.199563977	0.806142846
Glycyrrhiza_uralensis_Fisch0058190.1	-2.128295256	3.012411207	-0.706508876	0.479871735	0.93190416
Glycyrrhiza_uralensis_Fisch0117620.1	-2.127793584	2.101408576	-1.012555867	0.311272349	0.880015095
Glycyrrhiza_uralensis_Fisch0284820.1	-2.125675024	1.545868375	-1.375068576	0.169110186	0.776990799
Glycyrrhiza_uralensis_Fisch0251890.1	-2.124000015	1.482411031	-1.432801005	0.151914714	0.754869358
Glycyrrhiza_uralensis_Fisch0211820.1	-2.118757682	0.9484678	-2.233874131	0.025491356	0.390813339
Glycyrrhiza_uralensis_Fisch0205200.1	-2.117657813	1.397655346	-1.515150226	0.129734365	0.724285196
Glycyrrhiza_uralensis_Fisch0283620.1	-2.107713083	0.583563423	-3.611797793	0.000304082	0.029394558
Glycyrrhiza_uralensis_Fisch0176760.1	-2.100931031	0.774534874	-2.712506695	0.006677643	0.205013868
Glycyrrhiza_uralensis_Fisch0066890.1	-2.0986858	1.816762565	-1.15517891	0.248017169	0.843032358
Glycyrrhiza_uralensis_Fisch0053690.1	-2.095743829	2.235621648	-0.937432249	0.348536259	0.896081753
Glycyrrhiza_uralensis_Fisch0121250.1	-2.083588077	3.059200941	-0.681088989	0.495815184	0.937582768
Glycyrrhiza_uralensis_Fisch0217660.1	-2.079595012	0.972419394	-2.138578298	0.032469837	0.440911127
Glycyrrhiza_uralensis_Fisch0266920.1	-2.076586547	3.076417492	-0.675001541	0.499674786	0.938792103
Glycyrrhiza_uralensis_Fisch0133970.1	-2.073467231	1.856377706	-1.11694254	0.264018894	0.85183092
Glycyrrhiza_uralensis_Fisch0283440.1	-2.07283465	3.844769743	-0.539131024	0.58979645	NA
Glycyrrhiza_uralensis_Fisch0147010.1	-2.069964348	0.848897588	-2.438414688	0.01475184	0.300454876
Glycyrrhiza_uralensis_Fisch0	-2.067635346	0.672782	-3.073259	0.002117	0.107734

102950.1		448	94	34	233
Glycyrrhiza_uralensis_Fisch0156010.1	-2.066731804	2.294511399	-0.900728497	0.367732693	0.900974301
Glycyrrhiza_uralensis_Fisch0000140.1	-2.064358927	0.778171583	-2.652832579	0.007981945	0.225415683
Glycyrrhiza_uralensis_Fisch0275440.1	-2.061056934	2.778209694	-0.741865144	0.458169046	NA
Glycyrrhiza_uralensis_Fisch0277720.1	-2.060529592	2.654877114	-0.776129931	0.437672273	0.918289828
Glycyrrhiza_uralensis_Fisch0123130.1	-2.060416302	1.934163204	-1.065275308	NA	NA
Glycyrrhiza_uralensis_Fisch0161360.1	-2.059919904	3.080773483	-0.668637248	0.503726906	0.939462779
Glycyrrhiza_uralensis_Fisch0084560.1	-2.043773054	3.815626445	-0.535632375	0.592212649	NA
Glycyrrhiza_uralensis_Fisch0242330.1	-2.04076846	3.533390997	-0.577566553	0.563556794	NA
Glycyrrhiza_uralensis_Fisch0187960.1	-2.04041616	2.296924912	-0.888325147	0.374365877	0.90447444
Glycyrrhiza_uralensis_Fisch0071140.1	-2.039494367	0.492451192	-4.141515746	3.45E-05	0.005877175
Glycyrrhiza_uralensis_Fisch0218520.1	-2.037569777	3.277004115	-0.621778217	0.534087711	NA
Glycyrrhiza_uralensis_Fisch0285950.1	-2.03626615	3.180544364	-0.640225671	0.522025896	0.944252825
Glycyrrhiza_uralensis_Fisch0101670.1	-2.034337887	0.513892256	-3.958685624	7.54E-05	0.010425264
Glycyrrhiza_uralensis_Fisch0219360.1	-2.026410799	0.689585004	-2.938594642	0.00329704	0.138665403
Glycyrrhiza_uralensis_Fisch0005080.1	-2.023536973	3.106970982	-0.651289306	0.514859751	0.941847938
Glycyrrhiza_uralensis_Fisch0124180.1	-2.021191872	1.533712732	-1.317842533	0.187556366	0.797211647
Glycyrrhiza_uralensis_Fisch0236850.1	-2.01832511	0.733989144	-2.7498024	0.005963121	0.193439795
Glycyrrhiza_uralensis_Fisch0087810.1	-2.016932799	1.888071625	-1.068250151	0.285407688	0.862094057
Glycyrrhiza_uralensis_Fisch0111720.1	-2.016778373	1.084097734	-1.860328925	0.062839004	0.57702658
Glycyrrhiza_uralensis_Fisch0121930.1	-2.011644586	1.393391741	-1.443703538	0.148822386	0.750830863
Glycyrrhiza_uralensis_Fisch0183950.1	-2.01156175	0.832121365	-2.417389861	0.015632264	0.31188596
Glycyrrhiza_uralensis_Fisch0	-2.009095389	1.097833	-1.830054	0.067241	0.592655

185960.1		616	536	784	068
Glycyrrhiza_uralensis_Fisch0145350.1	-2.008755166	3.109858265	-0.64593142	0.51832377	0.943012745
Glycyrrhiza_uralensis_Fisch0203620.1	-1.996762695	1.217475353	-1.640084697	0.100987558	0.682417327
Glycyrrhiza_uralensis_Fisch0024190.1	-1.995916308	4.035111845	-0.494637171	0.620856259	NA
Glycyrrhiza_uralensis_Fisch0240080.1	-1.995916308	4.035111845	-0.494637171	0.620856259	NA
Glycyrrhiza_uralensis_Fisch0073120.1	-1.995916308	4.035111845	-0.494637171	0.620856259	NA
Glycyrrhiza_uralensis_Fisch0126700.1	-1.995916308	4.035111845	-0.494637171	0.620856259	NA
Glycyrrhiza_uralensis_Fisch0112920.1	-1.995916308	4.035111845	-0.494637171	0.620856259	NA
Glycyrrhiza_uralensis_Fisch0113350.1	-1.995916308	4.035111845	-0.494637171	0.620856259	NA
Glycyrrhiza_uralensis_Fisch0102580.1	-1.995916308	4.035111845	-0.494637171	0.620856259	NA
Glycyrrhiza_uralensis_Fisch0113930.1	-1.995916308	4.035111845	-0.494637171	0.620856259	NA
Glycyrrhiza_uralensis_Fisch0144730.1	-1.995916308	4.035111845	-0.494637171	0.620856259	NA
Glycyrrhiza_uralensis_Fisch0134950.1	-1.995916308	4.035111845	-0.494637171	0.620856259	NA
Glycyrrhiza_uralensis_Fisch0178650.1	-1.995916308	4.035111845	-0.494637171	0.620856259	NA
Glycyrrhiza_uralensis_Fisch0144140.1	-1.995916308	4.035111845	-0.494637171	0.620856259	NA
Glycyrrhiza_uralensis_Fisch0243070.1	-1.995916308	4.035111845	-0.494637171	0.620856259	NA
Glycyrrhiza_uralensis_Fisch0276030.1	-1.995916308	4.035111845	-0.494637171	0.620856259	NA
Glycyrrhiza_uralensis_Fisch0241540.1	-1.995916308	4.035111845	-0.494637171	0.620856259	NA
Glycyrrhiza_uralensis_Fisch0278980.1	-1.995916308	4.035111845	-0.494637171	0.620856259	NA
Glycyrrhiza_uralensis_Fisch0248440.1	-1.995102931	0.628716872	-3.173293132	0.001507203	0.088419538
Glycyrrhiza_uralensis_Fisch0015910.1	-1.993221986	2.009958045	-0.991673429	0.321356857	0.886369102
Glycyrrhiza_uralensis_Fisch0156860.1	-1.99189853	1.402048994	-1.420705366	0.155402432	0.75874765
Glycyrrhiza_uralensis_Fisch0	-1.989473153	1.266819	-1.570446	0.116311	0.705996

280790.1		914	699	225	975
Glycyrrhiza_uralensis_Fisch0013110.1	-1.985428413	0.94884963	-2.092458436	NA	NA
Glycyrrhiza_uralensis_Fisch0047050.1	-1.981652723	0.549433423	-3.606720376	0.000310092	0.029855611
Glycyrrhiza_uralensis_Fisch0157710.1	-1.978382125	2.051917182	-0.964162756	0.334964334	0.889039773
Glycyrrhiza_uralensis_Fisch0258420.1	-1.97388472	1.463467311	-1.348772675	0.177409994	0.788451579
Glycyrrhiza_uralensis_Fisch0020620.1	-1.973762795	1.960827669	-1.006596768	0.314128588	0.882272787
Glycyrrhiza_uralensis_Fisch0237130.1	-1.969006704	2.308956339	-0.852769137	0.393787342	0.910003682
Glycyrrhiza_uralensis_Fisch0166280.1	-1.962036527	4.038111857	-0.485879687	0.627052471	NA
Glycyrrhiza_uralensis_Fisch0084120.1	-1.962036527	4.038111857	-0.485879687	0.627052471	NA
Glycyrrhiza_uralensis_Fisch0087850.1	-1.962036527	4.038111857	-0.485879687	0.627052471	NA
Glycyrrhiza_uralensis_Fisch0059920.1	-1.962036527	4.038111857	-0.485879687	0.627052471	NA
Glycyrrhiza_uralensis_Fisch0120750.1	-1.962036527	4.038111857	-0.485879687	0.627052471	NA
Glycyrrhiza_uralensis_Fisch0002830.1	-1.962036527	4.038111857	-0.485879687	0.627052471	NA
Glycyrrhiza_uralensis_Fisch0053150.1	-1.962036527	4.038111857	-0.485879687	0.627052471	NA
Glycyrrhiza_uralensis_Fisch0098580.1	-1.962036527	4.038111857	-0.485879687	0.627052471	NA
Glycyrrhiza_uralensis_Fisch0115140.1	-1.962036527	4.038111857	-0.485879687	0.627052471	NA
Glycyrrhiza_uralensis_Fisch0006350.1	-1.962036527	4.038111857	-0.485879687	0.627052471	NA
Glycyrrhiza_uralensis_Fisch0159740.1	-1.962036527	4.038111857	-0.485879687	0.627052471	NA
Glycyrrhiza_uralensis_Fisch0286280.1	-1.962036527	4.038111857	-0.485879687	0.627052471	NA
Glycyrrhiza_uralensis_Fisch0147530.1	-1.962036527	4.038111857	-0.485879687	0.627052471	NA
Glycyrrhiza_uralensis_Fisch0194360.1	-1.962036527	4.038111857	-0.485879687	0.627052471	NA
Glycyrrhiza_uralensis_Fisch0007050.1	-1.962036527	4.038111857	-0.485879687	0.627052471	NA
Glycyrrhiza_uralensis_Fisch0	-1.962036527	4.038111	-0.485879	0.627052	NA

154700.1		857	687	471	
Glycyrrhiza_uralensis_Fisch0197560.1	-1.962036527	4.038111857	-0.485879687	0.627052471	NA
Glycyrrhiza_uralensis_Fisch0152020.1	-1.962036527	4.038111857	-0.485879687	0.627052471	NA
Glycyrrhiza_uralensis_Fisch0178770.1	-1.962036527	4.038111857	-0.485879687	0.627052471	NA
Glycyrrhiza_uralensis_Fisch0242000.1	-1.962036527	4.038111857	-0.485879687	0.627052471	NA
Glycyrrhiza_uralensis_Fisch0286520.1	-1.962036527	4.038111857	-0.485879687	0.627052471	NA
Glycyrrhiza_uralensis_Fisch0178180.1	-1.962036527	4.038111857	-0.485879687	0.627052471	NA
Glycyrrhiza_uralensis_Fisch0274430.1	-1.962036527	4.038111857	-0.485879687	0.627052471	NA
Glycyrrhiza_uralensis_Fisch0083160.1	-1.958423331	0.69363088	-2.823437346	0.004751171	0.170943755
Glycyrrhiza_uralensis_Fisch0244390.1	-1.957992624	2.842335013	-0.688867644	0.490906563	0.936011543
Glycyrrhiza_uralensis_Fisch0167750.1	-1.953455286	1.109714261	-1.760322774	0.078353095	0.627606986
Glycyrrhiza_uralensis_Fisch0284650.1	-1.95264801	1.454302572	-1.342669708	0.179378948	0.789067991
Glycyrrhiza_uralensis_Fisch0230090.1	-1.952114159	2.406056317	-0.811333528	0.417174161	0.913278077
Glycyrrhiza_uralensis_Fisch0093230.1	-1.951885383	0.646503594	-3.019140807	0.002534927	0.118477067
Glycyrrhiza_uralensis_Fisch0034680.1	-1.949658852	2.885439177	-0.675688771	0.499238267	0.938792103
Glycyrrhiza_uralensis_Fisch0151960.1	-1.941887279	2.92534684	-0.663814373	0.506809125	0.940226457
Glycyrrhiza_uralensis_Fisch0022530.1	-1.939966873	4.0401031	-0.480177566	0.631101138	NA
Glycyrrhiza_uralensis_Fisch0234100.1	-1.939966873	4.0401031	-0.480177566	0.631101138	NA
Glycyrrhiza_uralensis_Fisch0171610.1	-1.939966873	4.0401031	-0.480177566	0.631101138	NA
Glycyrrhiza_uralensis_Fisch0211200.1	-1.939966873	4.0401031	-0.480177566	0.631101138	NA
Glycyrrhiza_uralensis_Fisch0090190.1	-1.939966873	4.0401031	-0.480177566	0.631101138	NA
Glycyrrhiza_uralensis_Fisch0237320.1	-1.939966873	4.0401031	-0.480177566	0.631101138	NA
Glycyrrhiza_uralensis_Fisch0	-1.939966873	4.040103	-0.480177	0.631101	NA

051800.1		1	566	138	
Glycyrrhiza_uralensis_Fisch0 104780.1	-1.939966873	4.040103 1	-0.480177 566	0.631101 138	NA
Glycyrrhiza_uralensis_Fisch0 181400.1	-1.939966873	4.040103 1	-0.480177 566	0.631101 138	NA
Glycyrrhiza_uralensis_Fisch0 272320.1	-1.939966873	4.040103 1	-0.480177 566	0.631101 138	NA
Glycyrrhiza_uralensis_Fisch0 170680.1	-1.939966873	4.040103 1	-0.480177 566	0.631101 138	NA
Glycyrrhiza_uralensis_Fisch0 236730.1	-1.939966873	4.040103 1	-0.480177 566	0.631101 138	NA
Glycyrrhiza_uralensis_Fisch0 025530.1	-1.939966873	4.040103 1	-0.480177 566	0.631101 138	NA
Glycyrrhiza_uralensis_Fisch0 052770.1	-1.939966873	4.040103 1	-0.480177 566	0.631101 138	NA
Glycyrrhiza_uralensis_Fisch0 035070.1	-1.939966873	4.040103 1	-0.480177 566	0.631101 138	NA
Glycyrrhiza_uralensis_Fisch0 270360.1	-1.938563369	2.057841 409	-0.942037 302	0.346173 542	0.895282 815
Glycyrrhiza_uralensis_Fisch0 196990.1	-1.932665514	1.067595 826	-1.810296 994	0.070249 743	0.601523 553
Glycyrrhiza_uralensis_Fisch0 154420.1	-1.931994299	2.690451 64	-0.718092 929	0.472699 99	NA
Glycyrrhiza_uralensis_Fisch0 280870.1	-1.931626575	3.110448 422	-0.621012 251	0.534591 563	0.946078 885
Glycyrrhiza_uralensis_Fisch0 263840.1	-1.931593653	2.862979 182	-0.674679 601	0.499879 347	0.938792 103
Glycyrrhiza_uralensis_Fisch0 203270.1	-1.924694475	2.921423 486	-0.658820 772	0.510010 867	0.940226 457
Glycyrrhiza_uralensis_Fisch0 164470.1	-1.92195175	1.016769 427	-1.890253 287	0.058724 093	0.564267 034
Glycyrrhiza_uralensis_Fisch0 123750.1	-1.920988097	3.541840 882	-0.542369 96	0.587563 67	0.955972 525
Glycyrrhiza_uralensis_Fisch0 270320.1	-1.91318087	0.855723 353	-2.235746 942	0.025368 352	0.389175 423
Glycyrrhiza_uralensis_Fisch0 036100.1	-1.904922867	3.758563 761	-0.506822 017	0.612279 706	0.961668 681
Glycyrrhiza_uralensis_Fisch0 086930.1	-1.904678892	3.815028 246	-0.499256 826	0.617598 466	NA
Glycyrrhiza_uralensis_Fisch0 233610.1	-1.903507572	2.785008 964	-0.683483 463	0.494301 396	0.937222 783
Glycyrrhiza_uralensis_Fisch0 063260.1	-1.901674284	1.220422 82	-1.558209 378	0.119183 623	0.710739 792
Glycyrrhiza_uralensis_Fisch0	-1.901086157	1.170213	-1.624563	0.104255	0.690798

136140.1		305	786	539	139
Glycyrrhiza_uralensis_Fisch0088160.1	-1.899036149	1.043737421	-1.81945776	0.068841622	0.597968345
Glycyrrhiza_uralensis_Fisch0068380.1	-1.897937306	0.726973309	-2.610738638	0.009034691	0.236526869
Glycyrrhiza_uralensis_Fisch0102230.1	-1.896256304	0.782051841	-2.424719441	0.015320221	0.308052495
Glycyrrhiza_uralensis_Fisch0280090.1	-1.896112454	1.130032773	-1.677926959	0.093361359	0.663747356
Glycyrrhiza_uralensis_Fisch0027930.1	-1.895573446	2.877085621	-0.658851941	0.50999085	0.940226457
Glycyrrhiza_uralensis_Fisch0142430.1	-1.893696622	1.359754397	-1.39267549	NA	NA
Glycyrrhiza_uralensis_Fisch0104380.1	-1.890780884	0.965353894	-1.958640139	0.050154945	0.530417195
Glycyrrhiza_uralensis_Fisch0074660.1	-1.881607086	3.015463097	-0.62398611	0.532636707	0.94591302
Glycyrrhiza_uralensis_Fisch0186910.1	-1.879005145	1.140271596	-1.647857538	0.099381909	0.680028712
Glycyrrhiza_uralensis_Fisch0206360.1	-1.873809713	3.792885534	-0.494032761	0.621283043	0.962594079
Glycyrrhiza_uralensis_Fisch0153630.1	-1.872861679	1.314380327	-1.424900876	NA	NA
Glycyrrhiza_uralensis_Fisch0074840.1	-1.869730547	0.520036759	-3.595381512	0.000323917	0.030836749
Glycyrrhiza_uralensis_Fisch0145050.1	-1.8675455	1.435050709	-1.301379449	0.193128605	0.80004393
Glycyrrhiza_uralensis_Fisch0249110.1	-1.853183493	1.452426279	-1.275922585	0.201982887	0.806791915
Glycyrrhiza_uralensis_Fisch0231460.1	-1.852926099	0.812374217	-2.280877531	0.022555695	0.366834848
Glycyrrhiza_uralensis_Fisch0145140.1	-1.852787568	1.488874651	-1.244421461	0.213344489	0.814082192
Glycyrrhiza_uralensis_Fisch0265990.1	-1.851395587	2.836481249	-0.652708558	0.513944184	0.941686425
Glycyrrhiza_uralensis_Fisch0210330.1	-1.850995071	0.568053108	-3.258489471	0.00112007	0.072085809
Glycyrrhiza_uralensis_Fisch0072740.1	-1.849650946	2.308068539	-0.801384757	0.422908937	0.913935905
Glycyrrhiza_uralensis_Fisch0182210.1	-1.848489139	0.608499455	-3.037782737	0.002383257	0.115257626
Glycyrrhiza_uralensis_Fisch0059140.1	-1.847435752	1.768802966	-1.044455367	0.296274759	0.869761143
Glycyrrhiza_uralensis_Fisch0	-1.847071691	3.138352	-0.588548	0.556164	NA

163770.1		595	174	408	
Glycyrrhiza_uralensis_Fisch0273290.1	-1.846571579	2.046017254	-0.902520042	0.36678068	0.900974301
Glycyrrhiza_uralensis_Fisch0083370.1	-1.844457257	1.005191097	-1.834931946	0.066515774	0.590787701
Glycyrrhiza_uralensis_Fisch0084350.1	-1.843160012	1.242328682	-1.483633147	0.137906271	0.735920766
Glycyrrhiza_uralensis_Fisch0176890.1	-1.842492716	1.722902883	-1.069411825	0.284884141	0.862094057
Glycyrrhiza_uralensis_Fisch0006160.1	-1.838233768	0.914897599	-2.009223514	0.044513435	0.50820677
Glycyrrhiza_uralensis_Fisch0276570.1	-1.832250104	2.250736317	-0.814066974	0.415606584	0.91279862
Glycyrrhiza_uralensis_Fisch0286740.1	-1.828264354	2.455121034	-0.744673818	0.456468931	0.92402224
Glycyrrhiza_uralensis_Fisch0246230.1	-1.825785511	2.880763516	-0.63378528	0.526220961	0.944927971
Glycyrrhiza_uralensis_Fisch0167650.1	-1.824200626	2.79650768	-0.652313827	0.514198742	0.941720577
Glycyrrhiza_uralensis_Fisch0095290.1	-1.820404871	1.228913624	-1.481312304	0.138523371	0.736689692
Glycyrrhiza_uralensis_Fisch0241750.1	-1.812652088	3.144192017	-0.576508075	0.564271813	NA
Glycyrrhiza_uralensis_Fisch0280980.1	-1.812425352	0.695875972	-2.604523544	0.00920021	0.236747677
Glycyrrhiza_uralensis_Fisch0173190.1	-1.812268354	0.72414571	-2.502629414	0.012327455	0.274652222
Glycyrrhiza_uralensis_Fisch0092150.1	-1.811451355	1.093974849	-1.655843694	0.097753477	0.676623527
Glycyrrhiza_uralensis_Fisch0041300.1	-1.810044291	1.156269433	-1.5654174	0.117485071	0.706303999
Glycyrrhiza_uralensis_Fisch0004950.1	-1.806682499	1.096565233	-1.64758324	0.099438222	0.680028712
Glycyrrhiza_uralensis_Fisch0134460.1	-1.805068025	2.607857479	-0.692165135	0.488833631	0.935873483
Glycyrrhiza_uralensis_Fisch0275820.1	-1.799576384	1.745891318	-1.030749374	0.302658364	0.87348346
Glycyrrhiza_uralensis_Fisch0213100.1	-1.795240681	1.649980209	-1.088037706	0.276578462	0.859692133
Glycyrrhiza_uralensis_Fisch0131630.1	-1.789733361	2.729597969	-0.655676543	0.512032265	0.940947113
Glycyrrhiza_uralensis_Fisch0151810.1	-1.788590269	1.664422538	-1.074601087	0.282553365	0.86117142
Glycyrrhiza_uralensis_Fisch0	-1.787266202	0.915142	-1.952993	0.050820	0.532773

049550.1		082	132	409	19
Glycyrrhiza_uralensis_Fisch0286570.1	-1.783761241	2.510290923	-0.710579489	0.477344857	NA
Glycyrrhiza_uralensis_Fisch0238100.1	-1.783654153	2.922320842	-0.610355347	0.541626441	0.947234196
Glycyrrhiza_uralensis_Fisch0253700.1	-1.782596698	2.686147426	-0.663625787	0.506929849	0.940226457
Glycyrrhiza_uralensis_Fisch0281060.1	-1.780008374	1.004306736	-1.772375222	0.076332292	0.622773127
Glycyrrhiza_uralensis_Fisch0211250.1	-1.779763946	1.333891481	-1.334264423	0.182117213	0.791863958
Glycyrrhiza_uralensis_Fisch0056770.1	-1.777450017	0.671591368	-2.646624275	0.008129963	0.22728014
Glycyrrhiza_uralensis_Fisch0170570.1	-1.771863133	2.596819671	-0.682320437	0.495036353	NA
Glycyrrhiza_uralensis_Fisch0042630.1	-1.771530918	1.601899237	-1.105894102	0.268772358	0.855350341
Glycyrrhiza_uralensis_Fisch0155070.1	-1.771141454	2.553082752	-0.693726615	0.487853672	0.935346173
Glycyrrhiza_uralensis_Fisch0286910.1	-1.7702975	1.822644376	-0.971279709	0.33140901	0.887837307
Glycyrrhiza_uralensis_Fisch0271590.1	-1.766933414	1.0003611	-1.766295604	0.077346269	0.624530254
Glycyrrhiza_uralensis_Fisch0003970.1	-1.763196021	1.791729891	-0.984074681	0.325078795	0.887225647
Glycyrrhiza_uralensis_Fisch0201270.1	-1.757800803	0.709162162	-2.478700778	0.013186187	0.283565766
Glycyrrhiza_uralensis_Fisch0112010.1	-1.757598313	3.193113054	-0.550434101	0.582021665	NA
Glycyrrhiza_uralensis_Fisch0279350.1	-1.752451003	0.555680776	-3.153700972	0.001612142	0.091735824
Glycyrrhiza_uralensis_Fisch0219810.1	-1.751885088	0.996792058	-1.75752312	0.078828685	0.629619411
Glycyrrhiza_uralensis_Fisch0048350.1	-1.751366934	1.170791373	-1.495883019	0.134684142	0.7309299
Glycyrrhiza_uralensis_Fisch0072250.1	-1.750532516	1.215358895	-1.440342044	0.149770652	0.752762495
Glycyrrhiza_uralensis_Fisch0153580.1	-1.749932929	1.853567414	-0.944089175	0.345124081	0.894851545
Glycyrrhiza_uralensis_Fisch0251420.1	-1.748803812	1.536955909	-1.137836031	0.255188956	0.847381568
Glycyrrhiza_uralensis_Fisch0233590.1	-1.748663401	0.902902681	-1.936713046	0.05278043	0.540376416
Glycyrrhiza_uralensis_Fisch0	-1.745714478	2.606377	-0.669785	0.502994	0.939412

197510.1		421	758	373	59
Glycyrrhiza_uralensis_Fisch0061950.1	-1.745337162	2.445323771	-0.713744815	0.475384989	NA
Glycyrrhiza_uralensis_Fisch0070190.1	-1.744650854	1.51511727	-1.15149559	0.249528409	0.844321666
Glycyrrhiza_uralensis_Fisch0114660.1	-1.739082189	0.946621422	-1.837146455	0.066188278	0.589381612
Glycyrrhiza_uralensis_Fisch0252450.1	-1.738057728	1.188740981	-1.462099613	0.143713915	0.744212491
Glycyrrhiza_uralensis_Fisch0065840.1	-1.732239679	0.488241495	-3.547915729	0.000388292	0.034487819
Glycyrrhiza_uralensis_Fisch0250520.1	-1.731034348	1.493249717	-1.159239696	0.246358495	0.842096076
Glycyrrhiza_uralensis_Fisch0118280.1	-1.730526559	0.977545615	-1.770277041	0.076681001	0.623502421
Glycyrrhiza_uralensis_Fisch0242220.1	-1.73039475	1.126129559	-1.536585854	0.124394759	0.718559387
Glycyrrhiza_uralensis_Fisch0051480.1	-1.722382723	3.181919829	-0.541302992	0.588298759	NA
Glycyrrhiza_uralensis_Fisch0195030.1	-1.721745138	1.637543276	-1.051419626	0.293065906	0.868196475
Glycyrrhiza_uralensis_Fisch0061620.1	-1.721468402	1.302658577	-1.321503909	0.1863334	0.795093909
Glycyrrhiza_uralensis_Fisch0116140.1	-1.720609455	0.907895211	-1.895163049	0.058070804	0.562219369
Glycyrrhiza_uralensis_Fisch0286140.1	-1.719974601	1.301328859	-1.321706338	0.186265957	0.795093909
Glycyrrhiza_uralensis_Fisch0285260.1	-1.71882967	3.912832799	-0.439280122	0.660458576	NA
Glycyrrhiza_uralensis_Fisch0161110.1	-1.718075493	2.258035986	-0.760871617	0.446733753	0.921519204
Glycyrrhiza_uralensis_Fisch0286580.1	-1.717093951	2.137691535	-0.803246831	0.421832083	0.913755795
Glycyrrhiza_uralensis_Fisch0217700.1	-1.715369509	0.655004763	-2.618865703	0.008822267	0.233353815
Glycyrrhiza_uralensis_Fisch0048320.1	-1.711442783	1.121578995	-1.525922643	0.127029131	0.72182017
Glycyrrhiza_uralensis_Fisch0241860.1	-1.710733801	0.678919357	-2.519789402	0.011742507	0.268397024
Glycyrrhiza_uralensis_Fisch0198170.1	-1.710708353	2.766299991	-0.61841028	0.536304923	0.946841579
Glycyrrhiza_uralensis_Fisch0249430.1	-1.710537916	0.800726213	-2.136233194	0.032660411	0.442207404
Glycyrrhiza_uralensis_Fisch0	-1.707271371	1.511355	-1.129628	0.258632	0.848380

138380.1		995	874	639	675
Glycyrrhiza_uralensis_Fisch0086450.1	-1.706776834	3.848408592	-0.443501981	0.657402687	NA
Glycyrrhiza_uralensis_Fisch0154880.1	-1.706468041	0.58077492	-2.93826056	0.003300595	0.138665403
Glycyrrhiza_uralensis_Fisch0075100.1	-1.70432471	1.870249789	-0.911281862	0.362146881	0.899040488
Glycyrrhiza_uralensis_Fisch0056970.1	-1.703227031	2.869207916	-0.593622728	0.552764476	0.94916655
Glycyrrhiza_uralensis_Fisch0240180.1	-1.698590316	0.788370412	-2.154558681	0.031196385	0.432295334
Glycyrrhiza_uralensis_Fisch0212530.1	-1.698517698	3.19933151	-0.530897687	0.595489681	NA
Glycyrrhiza_uralensis_Fisch0253140.1	-1.696046557	0.941010258	-1.802367767	0.071487563	0.60422435
Glycyrrhiza_uralensis_Fisch0217670.1	-1.694822432	0.943882781	-1.795585709	0.07256043	0.607066235
Glycyrrhiza_uralensis_Fisch0082130.1	-1.694489132	1.067869555	-1.586794122	0.112559281	0.700471813
Glycyrrhiza_uralensis_Fisch0057550.1	-1.694345729	1.389549114	-1.219349293	0.222711647	0.820427829
Glycyrrhiza_uralensis_Fisch0177110.1	-1.693873385	3.779919399	-0.448124208	0.654063558	NA
Glycyrrhiza_uralensis_Fisch0247370.1	-1.693041935	1.514599679	-1.117814799	0.2636461	0.851448621
Glycyrrhiza_uralensis_Fisch0127210.1	-1.692039068	1.528084037	-1.107294512	0.268166616	0.85529651
Glycyrrhiza_uralensis_Fisch0274420.1	-1.689672111	0.558436599	-3.025718788	0.002480429	0.117415276
Glycyrrhiza_uralensis_Fisch0047060.1	-1.689557681	0.535032545	-3.157859638	0.001589321	0.090920841
Glycyrrhiza_uralensis_Fisch0237150.1	-1.686014497	0.781519669	-2.157353889	0.030978101	0.430680825
Glycyrrhiza_uralensis_Fisch0245220.1	-1.685647323	0.672799333	-2.505423593	0.012230482	0.273663217
Glycyrrhiza_uralensis_Fisch0277600.1	-1.683309552	0.572900792	-2.93822172	0.003301009	0.138665403
Glycyrrhiza_uralensis_Fisch0010050.1	-1.682394916	3.818339476	-0.440609047	0.659496052	NA
Glycyrrhiza_uralensis_Fisch0106270.1	-1.681335139	2.105877666	-0.798401145	0.424637739	0.915482048
Glycyrrhiza_uralensis_Fisch0174780.1	-1.680574031	0.830267384	-2.024135916	0.042956169	0.500462241
Glycyrrhiza_uralensis_Fisch0	-1.67783306	1.038324	-1.615904	0.106115	0.692097

255290.1		616	154	037	679
Glycyrrhiza_uralensis_Fisch00095580.1	-1.675877705	0.995843448	-1.682872653	NA	NA
Glycyrrhiza_uralensis_Fisch00156650.1	-1.673740087	0.82154673	-2.037303572	0.04161963	0.49475144
Glycyrrhiza_uralensis_Fisch00002860.1	-1.671314031	2.558827619	-0.653156164	0.513655607	NA
Glycyrrhiza_uralensis_Fisch00215440.1	-1.670587669	0.917041085	-1.821715183	0.068498213	0.597373909
Glycyrrhiza_uralensis_Fisch00031180.1	-1.670522485	0.720049255	-2.320011405	0.02034026	0.347651918
Glycyrrhiza_uralensis_Fisch00286080.1	-1.669205078	2.277994722	-0.732751952	0.463709731	NA
Glycyrrhiza_uralensis_Fisch00073010.1	-1.66781605	2.896664702	-0.575771179	0.564769857	0.951495097
Glycyrrhiza_uralensis_Fisch00032910.1	-1.666974337	1.618092369	-1.030209628	0.302911611	0.873493229
Glycyrrhiza_uralensis_Fisch00087780.1	-1.662087101	2.153910096	-0.771660388	0.440315597	0.919303851
Glycyrrhiza_uralensis_Fisch00211560.1	-1.661663077	0.60580897	-2.742882923	0.00609024	0.194366451
Glycyrrhiza_uralensis_Fisch00056960.1	-1.660716062	0.885769991	-1.87488409	0.060808671	0.571804506
Glycyrrhiza_uralensis_Fisch00221420.1	-1.656087263	0.613206687	-2.700699938	0.006919373	0.20798179
Glycyrrhiza_uralensis_Fisch00084440.1	-1.655609883	0.862394488	-1.919782544	0.054885373	0.549455166
Glycyrrhiza_uralensis_Fisch00083520.1	-1.655496137	0.739730964	-2.237970584	0.025222973	0.388430564
Glycyrrhiza_uralensis_Fisch00219320.1	-1.653857532	2.047815346	-0.807620441	0.419309116	0.913601698
Glycyrrhiza_uralensis_Fisch00244350.1	-1.653076974	1.325914769	-1.246744521	0.212491189	0.813575823
Glycyrrhiza_uralensis_Fisch00235100.1	-1.651290897	1.462921146	-1.128762751	0.25899793	0.848439484
Glycyrrhiza_uralensis_Fisch00154850.1	-1.646585439	2.992990597	-0.550147214	0.582218405	0.954219017
Glycyrrhiza_uralensis_Fisch00275190.1	-1.64643313	2.077905927	-0.792352102	0.428155399	0.916516843
Glycyrrhiza_uralensis_Fisch00232920.1	-1.645716783	2.560874719	-0.642638537	0.520458671	NA
Glycyrrhiza_uralensis_Fisch00279750.1	-1.645690371	1.551670192	-1.060592888	0.288874956	0.865554822
Glycyrrhiza_uralensis_Fisch0	-1.64498125	1.965999	-0.836714	0.402752	0.910612

209090.1		654	923	829	334
Glycyrrhiza_uralensis_Fisch0239630.1	-1.64127949	2.424727152	-0.676892445	0.498474198	0.938351305
Glycyrrhiza_uralensis_Fisch0260650.1	-1.639391952	3.191160049	-0.513729154	0.607441366	NA
Glycyrrhiza_uralensis_Fisch0041470.1	-1.638212186	0.839649841	-1.951065916	0.051049205	0.533776876
Glycyrrhiza_uralensis_Fisch0095220.1	-1.636380418	0.765541927	-2.137545132	0.032553679	0.441446228
Glycyrrhiza_uralensis_Fisch0009370.1	-1.635427087	0.814018452	-2.009078643	0.044528794	0.50820677
Glycyrrhiza_uralensis_Fisch0049170.1	-1.634692629	0.938973345	-1.740936137	0.081694774	0.637335735
Glycyrrhiza_uralensis_Fisch0028020.1	-1.634166046	3.070059231	-0.532291374	0.594524206	0.95798827
Glycyrrhiza_uralensis_Fisch0078790.1	-1.63234673	3.391537502	-0.481299921	0.630303354	NA
Glycyrrhiza_uralensis_Fisch0070750.1	-1.631475349	0.780902528	-2.089217656	0.036688135	0.467282603
Glycyrrhiza_uralensis_Fisch0122610.1	-1.63147412	2.398751791	-0.680134613	0.49641923	0.937582768
Glycyrrhiza_uralensis_Fisch0156150.1	-1.630155707	1.166627299	-1.397323472	0.162316321	0.767574428
Glycyrrhiza_uralensis_Fisch0006600.1	-1.629198825	0.920300556	-1.77028995	0.076678851	0.623502421
Glycyrrhiza_uralensis_Fisch0178330.1	-1.623841886	3.46756341	-0.468294792	0.639573792	0.965035372
Glycyrrhiza_uralensis_Fisch0049300.1	-1.622261177	1.435078175	-1.130434011	0.25829339	0.848148207
Glycyrrhiza_uralensis_Fisch0192340.1	-1.62189053	0.718568783	-2.257112427	0.024001051	0.378423129
Glycyrrhiza_uralensis_Fisch0154790.1	-1.621688148	1.475874916	-1.098797826	0.271856261	0.857255693
Glycyrrhiza_uralensis_Fisch0028690.1	-1.621262169	3.821935865	-0.424199208	0.671420538	NA
Glycyrrhiza_uralensis_Fisch0079880.1	-1.620430984	3.992709787	-0.405847424	0.68485471	NA
Glycyrrhiza_uralensis_Fisch0261070.1	-1.620229105	1.239456296	-1.307209548	0.191141554	0.798375977
Glycyrrhiza_uralensis_Fisch0134140.1	-1.616992714	0.503530041	-3.211313291	0.001321298	0.07931083
Glycyrrhiza_uralensis_Fisch0083180.1	-1.613609871	3.842693542	-0.419916356	0.674546559	NA
Glycyrrhiza_uralensis_Fisch0	-1.609548282	1.686172	-0.954557	0.339801	0.893467

068480.1		335	401	568	693
Glycyrrhiza_uralensis_Fisch0 235670.1	-1.609202499	2.793620 772	-0.576027 539	0.564596 567	0.951431 185
Glycyrrhiza_uralensis_Fisch0 202830.1	-1.609202379	1.440949 557	-1.116765 24	0.264094 715	0.851830 92
Glycyrrhiza_uralensis_Fisch0 112990.1	-1.608706612	3.993403 461	-0.402840 992	0.687065 203	NA
Glycyrrhiza_uralensis_Fisch0 170410.1	-1.607197528	1.034586 099	-1.553469 092	0.120311 108	0.711449 612
Glycyrrhiza_uralensis_Fisch0 065700.1	-1.605609888	1.168158 276	-1.374479 744	0.169292 795	0.777203 427
Glycyrrhiza_uralensis_Fisch0 004080.1	-1.602946288	2.952256 475	-0.542956 312	0.587159 883	0.955932 608
Glycyrrhiza_uralensis_Fisch0 031900.1	-1.600111373	0.744901 142	-2.148085 541	0.031706 961	0.436355 942
Glycyrrhiza_uralensis_Fisch0 049080.1	-1.59865256	1.408916 894	-1.134667 748	0.256514 555	0.847400 43
Glycyrrhiza_uralensis_Fisch0 024070.1	-1.59641831	3.240482 723	-0.492648 302	0.622261 113	NA
Glycyrrhiza_uralensis_Fisch0 048290.1	-1.595173073	1.016299 175	-1.569590 051	0.116510 515	0.706222 132
Glycyrrhiza_uralensis_Fisch0 257630.1	-1.595118892	1.408879 238	-1.132189 934	0.257554 591	0.847534 416
Glycyrrhiza_uralensis_Fisch0 275500.1	-1.591998722	0.700344 177	-2.273166 216	0.023016 165	0.369202 969
Glycyrrhiza_uralensis_Fisch0 095500.1	-1.590346402	2.193776 223	-0.724935 563	0.468491 562	0.927993 447
Glycyrrhiza_uralensis_Fisch0 049640.1	-1.588627274	2.133097 659	-0.744751 309	0.456422 075	0.924022 24
Glycyrrhiza_uralensis_Fisch0 032780.1	-1.585890244	3.796261 691	-0.417750 506	0.676129 541	0.968658 892
Glycyrrhiza_uralensis_Fisch0 185570.1	-1.585020903	3.994821 769	-0.396768 866	0.691537 911	NA
Glycyrrhiza_uralensis_Fisch0 036470.1	-1.583854018	3.515041 697	-0.450593 238	0.652282 741	NA
Glycyrrhiza_uralensis_Fisch0 252820.1	-1.58350961	0.660656 07	-2.396874 384	0.016535 591	0.316813 618
Glycyrrhiza_uralensis_Fisch0 071950.1	-1.58117812	0.910605 033	-1.736403 89	0.082492 445	0.639269 575
Glycyrrhiza_uralensis_Fisch0 278070.1	-1.578315372	0.749615 083	-2.105501 088	0.035247 713	0.456503 854
Glycyrrhiza_uralensis_Fisch0 275460.1	-1.578254309	3.790457 062	-0.416375 725	0.677135 088	0.968777 158
Glycyrrhiza_uralensis_Fisch0	-1.576653165	1.508276	-1.045334	0.295868	0.869761

258510.1		72	152	559	143
Glycyrrhiza_uralensis_Fisch0030460.1	-1.576523055	1.449025911	-1.087988174	0.276600328	0.859692133
Glycyrrhiza_uralensis_Fisch0157390.1	-1.57473176	0.898672236	-1.752287094	0.079724454	0.632826576
Glycyrrhiza_uralensis_Fisch0079290.1	-1.574110229	0.842204166	-1.869036384	0.061617753	0.573609826
Glycyrrhiza_uralensis_Fisch0041150.1	-1.573878344	3.265638041	-0.481951253	0.629840576	NA
Glycyrrhiza_uralensis_Fisch0048660.1	-1.572913819	0.687862582	-2.286668676	0.022215169	0.365057829
Glycyrrhiza_uralensis_Fisch0070880.1	-1.570971785	0.890840733	-1.763470986	0.077821087	0.626707386
Glycyrrhiza_uralensis_Fisch0234240.1	-1.570168329	1.639967984	-0.957438404	0.338346022	0.892394122
Glycyrrhiza_uralensis_Fisch0000040.1	-1.568326008	2.845705283	-0.551120321	0.581551196	0.954187955
Glycyrrhiza_uralensis_Fisch0255460.1	-1.568058579	2.382322367	-0.658205875	0.51040585	0.940226457
Glycyrrhiza_uralensis_Fisch0017050.1	-1.567465698	0.545042809	-2.875857957	0.00402931	0.155176783
Glycyrrhiza_uralensis_Fisch0114030.1	-1.566906691	1.412198371	-1.109551408	0.26719238	0.854585363
Glycyrrhiza_uralensis_Fisch0080020.1	-1.566375468	2.759425245	-0.567645552	0.57027567	0.952518696
Glycyrrhiza_uralensis_Fisch0018270.1	-1.564462886	2.104530916	-0.743378429	0.4572526	0.924102621
Glycyrrhiza_uralensis_Fisch0165920.1	-1.562958269	0.568038703	-2.751499608	0.005932309	0.192699972
Glycyrrhiza_uralensis_Fisch0190910.1	-1.562080105	2.149127167	-0.726843962	NA	NA
Glycyrrhiza_uralensis_Fisch0208170.1	-1.56018702	0.492744615	-3.166319776	0.00154381	0.089540967
Glycyrrhiza_uralensis_Fisch0239930.1	-1.559720891	2.24637949	-0.694326537	0.487477453	NA
Glycyrrhiza_uralensis_Fisch0223890.1	-1.556400053	2.155170785	-0.722170171	0.47018986	0.928837698
Glycyrrhiza_uralensis_Fisch0249790.1	-1.555811136	1.043067906	-1.491572242	0.13581132	0.731522758
Glycyrrhiza_uralensis_Fisch0121240.1	-1.551849211	1.450935112	-1.069551077	0.284821427	0.862094057
Glycyrrhiza_uralensis_Fisch0078970.1	-1.551501581	2.19234479	-0.7076905	0.479137478	0.931830837
Glycyrrhiza_uralensis_Fisch0	-1.550955354	0.772963	-2.006504	0.044802	0.508436

176720.1		637	938	402	505
Glycyrrhiza_uralensis_Fisch0 105240.1	-1.550634057	1.513959 048	-1.024224 571	0.305729 215	0.876161 605
Glycyrrhiza_uralensis_Fisch0 190740.1	-1.550263662	0.575106 993	-2.695609 129	0.007026 006	0.209390 819
Glycyrrhiza_uralensis_Fisch0 138340.1	-1.540410617	0.776117 368	-1.984765 036	0.047170 619	0.519037 46
Glycyrrhiza_uralensis_Fisch0 240940.1	-1.540311989	0.467031 788	-3.298088 115	0.000973 456	0.065267 636
Glycyrrhiza_uralensis_Fisch0 209560.1	-1.539153028	0.555736 58	-2.769573 004	0.005612 982	0.186866 509
Glycyrrhiza_uralensis_Fisch0 225810.1	-1.537667784	1.889866 033	-0.813638 51	0.415852 069	0.912798 62
Glycyrrhiza_uralensis_Fisch0 280900.1	-1.537017369	0.606291 437	-2.535113 108	0.011241 102	0.264482 278
Glycyrrhiza_uralensis_Fisch0 130760.1	-1.53676449	0.564337 617	-2.723129 638	0.006466 668	0.200325 214
Glycyrrhiza_uralensis_Fisch0 180860.1	-1.536443657	3.607336 828	-0.425921 873	0.670164 776	0.966501 962
Glycyrrhiza_uralensis_Fisch0 181600.1	-1.536411631	0.841496 657	-1.825808 359	0.067879 134	0.594613 222
Glycyrrhiza_uralensis_Fisch0 047320.1	-1.534444903	0.800579 894	-1.916666 8	0.055280 276	0.550743 474
Glycyrrhiza_uralensis_Fisch0 095720.1	-1.530307793	1.899998 817	-0.805425 656	0.420574 092	0.913746 696
Glycyrrhiza_uralensis_Fisch0 235580.1	-1.529136595	1.000022 674	-1.529101 924	0.126239 178	0.720896 091
Glycyrrhiza_uralensis_Fisch0 029580.1	-1.528883564	2.389893 867	-0.639728 645	0.522349 029	0.944252 825
Glycyrrhiza_uralensis_Fisch0 142850.1	-1.527685824	0.984200 444	-1.552210 053	0.120611 971	0.711449 612
Glycyrrhiza_uralensis_Fisch0 081580.1	-1.52572846	1.556515 432	-0.980220 581	0.326977 248	0.887225 647
Glycyrrhiza_uralensis_Fisch0 232070.1	-1.521572203	3.823711 998	-0.397930 651	0.690681 306	NA
Glycyrrhiza_uralensis_Fisch0 174370.1	-1.516767983	3.919624 606	-0.386967 665	0.698780 146	NA
Glycyrrhiza_uralensis_Fisch0 254730.1	-1.51626385	0.423764 059	-3.578085 064	0.000346 121	0.032291 194
Glycyrrhiza_uralensis_Fisch0 208400.1	-1.51346643	0.480493 233	-3.149818 405	0.001633 72	0.092152 854
Glycyrrhiza_uralensis_Fisch0 134520.1	-1.513388663	1.600893 154	-0.945340 205	0.344485 219	0.894405 752
Glycyrrhiza_uralensis_Fisch0	-1.510595337	0.830248	-1.819448	0.068842	0.597968

160370.1		856	862	978	345
Glycyrrhiza_uralensis_Fisch0016960.1	-1.509862961	1.05666573	-1.428893659	0.1530348	0.757607737
Glycyrrhiza_uralensis_Fisch0012070.1	-1.509351961	1.101118874	-1.370743883	0.17045481	0.778850891
Glycyrrhiza_uralensis_Fisch0283340.1	-1.507082142	0.599036404	-2.515843996	0.011874773	0.269392827
Glycyrrhiza_uralensis_Fisch0174350.1	-1.504847507	0.595629569	-2.526482204	0.011521123	0.266647518
Glycyrrhiza_uralensis_Fisch0046880.1	-1.504635182	0.344501661	-4.367570182	1.26E-05	0.002584674
Glycyrrhiza_uralensis_Fisch0071960.1	-1.504416846	0.733317463	-2.051521913	0.040216148	0.487167931
Glycyrrhiza_uralensis_Fisch0147280.1	-1.504388171	1.190260761	-1.263914782	0.206260615	0.81096352
Glycyrrhiza_uralensis_Fisch0124670.1	-1.501291011	1.99979178	-0.750723663	0.452818979	0.922710496
Glycyrrhiza_uralensis_Fisch0080270.1	-1.500893172	0.764750828	-1.962591104	0.049693705	0.52913375
Glycyrrhiza_uralensis_Fisch0230150.1	-1.499599747	1.625012175	-0.922823699	0.356099088	0.897807124
Glycyrrhiza_uralensis_Fisch0107110.1	-1.495289387	0.6186899	-2.416864065	0.015654862	0.31188596
Glycyrrhiza_uralensis_Fisch0027180.1	-1.48973275	2.091890124	-0.712146749	0.476373908	0.930301083
Glycyrrhiza_uralensis_Fisch0052620.1	-1.48875847	1.109629781	-1.341671335	0.179702585	0.789067991
Glycyrrhiza_uralensis_Fisch0231590.1	-1.488708951	2.654013491	-0.560927424	0.574847014	0.952669211
Glycyrrhiza_uralensis_Fisch0011670.1	-1.487515131	1.13195376	-1.314112982	0.188808186	0.797474632
Glycyrrhiza_uralensis_Fisch0076360.1	-1.485168628	2.9667222	-0.500609268	0.616646139	0.96223075
Glycyrrhiza_uralensis_Fisch0186920.1	-1.484752054	2.428754034	-0.611322527	0.54098608	0.947110698
Glycyrrhiza_uralensis_Fisch0257240.1	-1.483773753	3.824417952	-0.387973745	0.698035464	NA
Glycyrrhiza_uralensis_Fisch0240290.1	-1.482359112	1.021146363	-1.451661746	0.146595681	0.745903116
Glycyrrhiza_uralensis_Fisch0128880.1	-1.481668929	0.685839997	-2.160371129	0.030743951	0.429546783
Glycyrrhiza_uralensis_Fisch0141030.1	-1.477700013	2.683020342	-0.550759899	0.581798277	NA
Glycyrrhiza_uralensis_Fisch0	-1.477559652	0.658122	-2.245111	0.024760	0.385758

171420.1		999	711	962	159
Glycyrrhiza_uralensis_Fisch0237630.1	-1.477434878	1.558780074	-0.947814834	0.343223727	0.894315373
Glycyrrhiza_uralensis_Fisch0272410.1	-1.476152709	0.711194046	-2.075597675	0.037931177	0.476515356
Glycyrrhiza_uralensis_Fisch0090670.1	-1.474569476	2.848768435	-0.517616475	0.604725884	0.959659299
Glycyrrhiza_uralensis_Fisch0060870.1	-1.474455791	2.88768598	-0.510601153	0.609630368	0.96164146
Glycyrrhiza_uralensis_Fisch0166740.1	-1.474389722	1.467963741	-1.00437748	0.315196701	0.882780387
Glycyrrhiza_uralensis_Fisch0178120.1	-1.471898011	3.054720023	-0.481843835	0.629916887	0.963563011
Glycyrrhiza_uralensis_Fisch0263770.1	-1.471487885	1.066853758	-1.379277969	0.167809066	0.775569168
Glycyrrhiza_uralensis_Fisch0180620.1	-1.470177032	3.297583331	-0.445834687	0.655716664	NA
Glycyrrhiza_uralensis_Fisch0163910.1	-1.469988101	0.543704552	-2.703652372	0.0068582	0.207712166
Glycyrrhiza_uralensis_Fisch0168990.1	-1.469974414	1.236573035	-1.188748559	0.234538626	0.830738508
Glycyrrhiza_uralensis_Fisch0286470.1	-1.466820359	1.390070819	-1.055212683	0.291328065	0.867015168
Glycyrrhiza_uralensis_Fisch0286170.1	-1.466776886	1.783035646	-0.822629031	0.410718984	0.911783198
Glycyrrhiza_uralensis_Fisch0168920.1	-1.461421577	0.807827759	-1.80907571	0.070439242	0.601523553
Glycyrrhiza_uralensis_Fisch0141390.1	-1.459500205	0.392235999	-3.720974641	0.000198455	0.021811967
Glycyrrhiza_uralensis_Fisch0179100.1	-1.457324623	0.969405942	-1.503317196	0.132757268	0.728560747
Glycyrrhiza_uralensis_Fisch0174090.1	-1.455609445	1.509040721	-0.964592555	0.33474893	0.888988497
Glycyrrhiza_uralensis_Fisch0009460.1	-1.453915314	2.739554586	-0.530712299	0.595618162	NA
Glycyrrhiza_uralensis_Fisch0154400.1	-1.453354494	1.495003591	-0.972141139	0.330980338	0.887837307
Glycyrrhiza_uralensis_Fisch0155390.1	-1.45277164	3.128134414	-0.464421105	0.642346072	0.965035372
Glycyrrhiza_uralensis_Fisch0266690.1	-1.451411732	1.85673656	-0.781700411	0.43439066	0.917057463
Glycyrrhiza_uralensis_Fisch0078230.1	-1.451077998	1.367719374	-1.060947169	0.288713911	0.865554822
Glycyrrhiza_uralensis_Fisch0	-1.449408797	1.506321	-0.962217	0.335940	0.890101

183900.1		94	145	538	398
Glycyrrhiza_uralensis_Fisch0011070.1	-1.448676388	0.670544223	-2.16044869	0.030737952	0.429546783
Glycyrrhiza_uralensis_Fisch0111780.1	-1.448152093	2.657651483	-0.544899172	0.585822866	0.955565775
Glycyrrhiza_uralensis_Fisch0053530.1	-1.447507708	1.493413482	-0.969261176	0.332414895	0.888277533
Glycyrrhiza_uralensis_Fisch0071210.1	-1.445814724	0.58204633	-2.484019999	0.012990851	0.282083407
Glycyrrhiza_uralensis_Fisch0188430.1	-1.445629523	1.44631846	-0.999523662	0.317541083	0.883943851
Glycyrrhiza_uralensis_Fisch0112170.1	-1.444745607	1.176542542	-1.227958663	0.219462486	0.817830773
Glycyrrhiza_uralensis_Fisch0027330.1	-1.444323749	1.32330375	-1.091452925	0.275073635	0.859092046
Glycyrrhiza_uralensis_Fisch0258410.1	-1.441805643	1.102494353	-1.307766918	0.190952378	0.798375977
Glycyrrhiza_uralensis_Fisch0184450.1	-1.441289372	0.75306818	-1.913889619	0.055634261	0.551985435
Glycyrrhiza_uralensis_Fisch0112220.1	-1.44126233	0.768739929	-1.874837349	0.060815103	0.571804506
Glycyrrhiza_uralensis_Fisch0177740.1	-1.438911565	1.794754758	-0.801731578	0.422708245	0.913852659
Glycyrrhiza_uralensis_Fisch0157100.1	-1.435662836	0.765486969	-1.875489581	0.060725402	0.571804506
Glycyrrhiza_uralensis_Fisch0086150.1	-1.434210984	1.816166476	-0.789691365	0.429708035	NA
Glycyrrhiza_uralensis_Fisch0281420.1	-1.434165846	1.33447124	-1.074707197	0.282505841	0.86117142
Glycyrrhiza_uralensis_Fisch0178190.1	-1.432819609	0.770332184	-1.860002267	0.062885205	0.57702658
Glycyrrhiza_uralensis_Fisch0268510.1	-1.430797079	1.7655979	-0.810375386	0.41772446	0.913280981
Glycyrrhiza_uralensis_Fisch0142170.1	-1.429642632	1.602960098	-0.891876619	0.372459068	0.90343083
Glycyrrhiza_uralensis_Fisch0221740.1	-1.428215731	1.397138875	-1.022243212	0.306665803	0.876615969
Glycyrrhiza_uralensis_Fisch0204760.1	-1.428129958	1.10797839	-1.288951094	0.197415089	0.806142846
Glycyrrhiza_uralensis_Fisch0132290.1	-1.424346874	0.386812478	-3.682267129	0.000231169	0.024268264
Glycyrrhiza_uralensis_Fisch0034870.1	-1.421267214	0.6726148	-2.113047787	0.034596678	0.45379288
Glycyrrhiza_uralensis_Fisch0	-1.421037397	1.241648	-1.144476	0.252426	0.845754

252510.1		835	085	25	433
Glycyrrhiza_uralensis_Fisch0 101300.1	-1.420648506	1.169815 442	-1.214421 057	0.224586 978	0.821475 428
Glycyrrhiza_uralensis_Fisch0 035990.1	-1.420113867	2.261749 8	-0.627882 831	0.530080 699	0.945670 257
Glycyrrhiza_uralensis_Fisch0 255630.1	-1.419007058	1.109665 856	-1.278769 686	0.200978 172	0.806142 846
Glycyrrhiza_uralensis_Fisch0 164480.1	-1.417825255	0.737413 999	-1.922699 13	0.054517 846	0.548718 966
Glycyrrhiza_uralensis_Fisch0 017030.1	-1.415809236	1.895664 675	-0.746866 92	0.455143 896	0.923744 623
Glycyrrhiza_uralensis_Fisch0 095250.1	-1.414047153	0.343978 361	-4.110860 782	3.94E-05	0.006373 68
Glycyrrhiza_uralensis_Fisch0 112470.1	-1.413223711	1.597264 057	-0.884777 757	0.376276 508	0.904514 347
Glycyrrhiza_uralensis_Fisch0 002090.1	-1.413167434	1.498789 602	-0.942872 457	0.345746 145	0.894875 771
Glycyrrhiza_uralensis_Fisch0 115790.1	-1.412375227	0.784109 996	-1.801246 298	0.071664 068	0.605247 058
Glycyrrhiza_uralensis_Fisch0 031650.1	-1.412304491	2.726473 756	-0.517996 73	0.604460 551	0.959570 366
Glycyrrhiza_uralensis_Fisch0 223540.1	-1.411592594	0.875250 225	-1.612787 467	0.106790 69	0.692097 679
Glycyrrhiza_uralensis_Fisch0 285390.1	-1.409026087	0.498505 491	-2.826500 632	0.004705 965	0.170453 588
Glycyrrhiza_uralensis_Fisch0 004660.1	-1.408808924	0.808870 252	-1.741699 512	0.081561 038	0.636980 592
Glycyrrhiza_uralensis_Fisch0 194050.1	-1.405781101	0.944751 222	-1.487990 773	0.136753 327	0.733728 967
Glycyrrhiza_uralensis_Fisch0 166320.1	-1.40530851	2.865562 343	-0.490412 82	0.623841 806	0.963420 425
Glycyrrhiza_uralensis_Fisch0 118410.1	-1.405253603	0.428432 958	-3.279984 831	0.001038 127	0.068272 429
Glycyrrhiza_uralensis_Fisch0 005750.1	-1.405007701	0.780711 065	-1.799651 324	0.071915 712	0.606096 351
Glycyrrhiza_uralensis_Fisch0 209870.1	-1.403470573	1.909067 24	-0.735160 367	0.462241 833	0.924944 794
Glycyrrhiza_uralensis_Fisch0 136830.1	-1.4033019	2.874707 374	-0.488154 695	0.625440 272	0.963502 294
Glycyrrhiza_uralensis_Fisch0 260850.1	-1.403143881	0.730930 534	-1.919667 896	0.054899 862	0.549455 166
Glycyrrhiza_uralensis_Fisch0 252890.1	-1.401899722	1.097250 87	-1.277647 401	0.201373 779	0.806142 846
Glycyrrhiza_uralensis_Fisch0	-1.401470573	2.091326	-0.670134	0.502771	0.939412

044650.1		134	873	816	59
Glycyrrhiza_uralensis_Fisch0034900.1	-1.401092756	1.342014498	-1.044022071	0.296475178	0.869761143
Glycyrrhiza_uralensis_Fisch0224630.1	-1.400794295	3.271829299	-0.428137952	0.668550687	NA
Glycyrrhiza_uralensis_Fisch0272340.1	-1.40010997	0.658843657	-2.125101996	0.033578103	0.44905883
Glycyrrhiza_uralensis_Fisch0232130.1	-1.398994349	0.636021716	-2.199601545	0.027835177	0.409522135
Glycyrrhiza_uralensis_Fisch0091550.1	-1.397954534	0.663034082	-2.108420323	0.034994648	0.455062765
Glycyrrhiza_uralensis_Fisch0140120.1	-1.397386773	0.926402422	-1.508401468	0.131451807	0.727359417
Glycyrrhiza_uralensis_Fisch0188510.1	-1.396699766	1.255715948	-1.112273654	0.266020508	0.854047617
Glycyrrhiza_uralensis_Fisch0106200.1	-1.395491913	0.461243045	-3.025502344	0.002482205	0.117415276
Glycyrrhiza_uralensis_Fisch0014280.1	-1.395362355	3.232169436	-0.43171077	0.665951639	NA
Glycyrrhiza_uralensis_Fisch0067140.1	-1.394652541	0.849091376	-1.642523503	0.100481559	0.681701613
Glycyrrhiza_uralensis_Fisch0011910.1	-1.393398182	1.351058945	-1.031337816	0.302382431	0.87347594
Glycyrrhiza_uralensis_Fisch0282580.1	-1.38947869	1.082604257	-1.283459475	0.199331149	0.806142846
Glycyrrhiza_uralensis_Fisch0257530.1	-1.389440419	1.103397458	-1.259238372	0.207944246	0.811901001
Glycyrrhiza_uralensis_Fisch0277010.1	-1.389080633	0.813148487	-1.708274264	0.087585464	0.649470772
Glycyrrhiza_uralensis_Fisch0153550.1	-1.388557832	0.625797523	-2.218861184	0.02649617	0.39889536
Glycyrrhiza_uralensis_Fisch0095600.1	-1.386744213	0.997396629	-1.390363846	0.164418419	0.770229223
Glycyrrhiza_uralensis_Fisch0194560.1	-1.385414723	1.697861398	-0.815976336	0.41451367	0.912286761
Glycyrrhiza_uralensis_Fisch0138020.1	-1.384418008	3.578750616	-0.386843945	0.698871741	NA
Glycyrrhiza_uralensis_Fisch0045200.1	-1.381676425	0.413740136	-3.339478833	0.000839357	0.059227469
Glycyrrhiza_uralensis_Fisch0133270.1	-1.379950296	0.587639855	-2.348292556	0.018859701	0.335268105
Glycyrrhiza_uralensis_Fisch0256850.1	-1.379892194	2.285849279	-0.603667183	0.546064937	0.948067715
Glycyrrhiza_uralensis_Fisch0	-1.37942977	0.405413	-3.402523	0.000667	0.051180

272620.1		697	846	665	584
Glycyrrhiza_uralensis_Fisch0195330.1	-1.378077912	0.582934424	-2.364035911	0.01807706	0.329632454
Glycyrrhiza_uralensis_Fisch0285660.1	-1.376033146	3.908593232	-0.3520533	0.72479829	NA
Glycyrrhiza_uralensis_Fisch0088760.1	-1.374910284	1.111901754	-1.236539361	0.216258144	0.816005312
Glycyrrhiza_uralensis_Fisch0182070.1	-1.373723805	0.645240072	-2.129011923	0.033253274	0.446904693
Glycyrrhiza_uralensis_Fisch0153800.1	-1.369340867	2.382800482	-0.574677098	0.565509699	NA
Glycyrrhiza_uralensis_Fisch0257560.1	-1.369211447	3.107621515	-0.440597879	0.659504139	0.96632848
Glycyrrhiza_uralensis_Fisch0189890.1	-1.36861305	0.601780415	-2.274273169	0.022949567	0.369126803
Glycyrrhiza_uralensis_Fisch0034110.1	-1.368508066	2.600320904	-0.5262843	0.59869069	0.95843753
Glycyrrhiza_uralensis_Fisch0223510.1	-1.368473959	0.984185648	-1.390463233	0.164388256	0.770229223
Glycyrrhiza_uralensis_Fisch0152640.1	-1.36845556	1.532811653	-0.892774763	0.371977804	0.90343083
Glycyrrhiza_uralensis_Fisch0233680.1	-1.366755719	1.215357457	-1.124570974	0.260770879	0.850426487
Glycyrrhiza_uralensis_Fisch0072890.1	-1.366633387	2.826495306	-0.483508104	0.628735007	0.963563011
Glycyrrhiza_uralensis_Fisch0118070.1	-1.365541062	2.77460461	-0.492156993	0.622608365	0.962923206
Glycyrrhiza_uralensis_Fisch0212260.1	-1.364931373	0.363601051	-3.753925816	0.000174086	0.019891479
Glycyrrhiza_uralensis_Fisch0037720.1	-1.363745206	1.018759128	-1.338633607	0.18068998	0.789903344
Glycyrrhiza_uralensis_Fisch0148560.1	-1.363157519	2.753335258	-0.495093184	0.620534345	0.962594079
Glycyrrhiza_uralensis_Fisch0188420.1	-1.362813814	0.599829388	-2.272002408	0.023086365	0.369719763
Glycyrrhiza_uralensis_Fisch0028990.1	-1.360914433	2.230475115	-0.610145535	0.541765405	0.947234196
Glycyrrhiza_uralensis_Fisch0233360.1	-1.360227936	1.363507897	-0.997594469	0.318476044	0.885080409
Glycyrrhiza_uralensis_Fisch0065180.1	-1.359979824	3.571271078	-0.380811144	0.70334339	NA
Glycyrrhiza_uralensis_Fisch0243700.1	-1.359122033	0.794226294	-1.711252879	0.087034441	0.648142654
Glycyrrhiza_uralensis_Fisch0	-1.358646008	2.838418	-0.478663	0.632178	0.963563

175020.1		631	011	384	011
Glycyrrhiza_uralensis_Fisch0196640.1	-1.357851716	0.851290911	-1.595050175	0.110701016	0.698157566
Glycyrrhiza_uralensis_Fisch0249390.1	-1.357595271	1.126420881	-1.205229141	0.228114856	0.825365409
Glycyrrhiza_uralensis_Fisch0266260.1	-1.352723476	0.73785511	-1.833318571	0.066755209	0.591625745
Glycyrrhiza_uralensis_Fisch0212820.1	-1.351611479	2.1898285	-0.617222526	0.537087959	0.947110698
Glycyrrhiza_uralensis_Fisch0251690.1	-1.350312923	0.720520186	-1.874080627	0.060919313	0.572113877
Glycyrrhiza_uralensis_Fisch0119910.1	-1.349358119	2.926214599	-0.461127533	0.644707109	0.965587841
Glycyrrhiza_uralensis_Fisch0005490.1	-1.348867015	3.81808669	-0.353283496	0.723875917	NA
Glycyrrhiza_uralensis_Fisch0045800.1	-1.348867015	3.81808669	-0.353283496	0.723875917	NA
Glycyrrhiza_uralensis_Fisch0027070.1	-1.347823272	0.788947893	-1.708380597	0.087565745	0.649470772
Glycyrrhiza_uralensis_Fisch0285570.1	-1.347703226	0.407623054	-3.306248781	0.000945541	0.064473572
Glycyrrhiza_uralensis_Fisch0096920.1	-1.347621644	1.446510411	-0.931636325	0.351524504	0.897130588
Glycyrrhiza_uralensis_Fisch0186450.1	-1.344999772	1.269159811	-1.059756037	0.289255602	0.865860259
Glycyrrhiza_uralensis_Fisch0206510.1	-1.344435372	2.525113788	-0.532425659	0.594431218	NA
Glycyrrhiza_uralensis_Fisch0018310.1	-1.342761901	0.329327371	-4.077286072	4.56E-05	0.007030356
Glycyrrhiza_uralensis_Fisch0049850.1	-1.340988654	0.423418559	-3.167052141	0.001539927	0.089540967
Glycyrrhiza_uralensis_Fisch0074520.1	-1.340697147	0.798595328	-1.678819172	0.093187291	0.663026335
Glycyrrhiza_uralensis_Fisch0239580.1	-1.34006921	1.227548474	-1.091662967	0.274981268	0.858915018
Glycyrrhiza_uralensis_Fisch0191270.1	-1.339884329	1.375739003	-0.973937881	0.330087384	0.887225647
Glycyrrhiza_uralensis_Fisch0092490.1	-1.337251382	0.585125139	-2.285410921	0.022288744	0.365057829
Glycyrrhiza_uralensis_Fisch0227910.1	-1.336699043	0.962069281	-1.389399983	0.164711155	0.770273462
Glycyrrhiza_uralensis_Fisch0087360.1	-1.335852491	1.6912412	-0.789865154	0.429606524	0.916516843
Glycyrrhiza_uralensis_Fisch0	-1.33479963	0.465165	-2.869517	0.004110	0.157315

089020.1		184	484	986	475
Glycyrrhiza_uralensis_Fisch0154780.1	-1.334550168	1.501772402	-0.888650082	0.374191166	0.90447444
Glycyrrhiza_uralensis_Fisch0287000.1	-1.330692885	0.979170866	-1.358999671	0.174146693	0.783367469
Glycyrrhiza_uralensis_Fisch0005540.1	-1.329648227	0.645275344	-2.060590474	0.039342126	0.481901786
Glycyrrhiza_uralensis_Fisch0001190.1	-1.329429047	0.990864927	-1.341685442	0.179698009	0.789067991
Glycyrrhiza_uralensis_Fisch0172520.1	-1.329177536	1.190890168	-1.116121009	0.26437034	0.85183092
Glycyrrhiza_uralensis_Fisch0077940.1	-1.328181703	1.507513995	-0.881041043	0.378295606	0.904607774
Glycyrrhiza_uralensis_Fisch0123060.1	-1.32722338	0.35908921	-3.696082595	0.000218952	0.023422973
Glycyrrhiza_uralensis_Fisch0273560.1	-1.324378878	0.437769774	-3.025286247	0.002483979	0.117415276
Glycyrrhiza_uralensis_Fisch0050490.1	-1.324205681	3.818558063	-0.346781602	0.7287554	NA
Glycyrrhiza_uralensis_Fisch0284470.1	-1.323364419	1.631129591	-0.811317768	0.417183209	0.913278077
Glycyrrhiza_uralensis_Fisch0040180.1	-1.321685717	0.40698891	-3.247473541	0.001164345	0.073946653
Glycyrrhiza_uralensis_Fisch0220740.1	-1.32160888	0.837424011	-1.578183648	0.114523426	0.702850296
Glycyrrhiza_uralensis_Fisch0275000.1	-1.321072828	0.899992334	-1.4678712	0.142139212	0.742901537
Glycyrrhiza_uralensis_Fisch0013630.1	-1.319971739	2.185960979	-0.603840486	0.5459497	0.948067715
Glycyrrhiza_uralensis_Fisch0178870.1	-1.319604177	0.89539518	-1.473767346	0.140544243	0.740564784
Glycyrrhiza_uralensis_Fisch0168550.1	-1.316002095	0.667304416	-1.97211657	0.048596301	0.525390669
Glycyrrhiza_uralensis_Fisch0078500.1	-1.315905039	1.363797881	-0.964882742	0.334603546	0.888988497
Glycyrrhiza_uralensis_Fisch0263170.1	-1.314736238	0.554880825	-2.369402903	0.017816833	0.32694177
Glycyrrhiza_uralensis_Fisch0084780.1	-1.314289557	0.62219381	-2.112347531	0.034656652	0.45379288
Glycyrrhiza_uralensis_Fisch0037160.1	-1.313945878	1.381599907	-0.951032112	0.341588075	0.894105199
Glycyrrhiza_uralensis_Fisch0159760.1	-1.312650736	1.120375692	-1.17161658	0.241351029	0.837396698
Glycyrrhiza_uralensis_Fisch0	-1.312031042	2.019163	-0.649789	NA	NA

283190.1		392	436		
Glycyrrhiza_uralensis_Fisch0227200.1	-1.311355043	0.731444475	-1.792829241	0.073000232	0.609263382
Glycyrrhiza_uralensis_Fisch0234210.1	-1.311286192	1.060372288	-1.236628122	0.216225175	0.816005312
Glycyrrhiza_uralensis_Fisch0155480.1	-1.310890809	0.723291294	-1.812396775	0.069924911	0.600931266
Glycyrrhiza_uralensis_Fisch0225680.1	-1.309984174	2.148582924	-0.609696819	0.542062662	0.94732072
Glycyrrhiza_uralensis_Fisch0061750.1	-1.308914603	0.667089952	-1.962126096	0.049747805	0.52913375
Glycyrrhiza_uralensis_Fisch0018570.1	-1.308828038	0.504603163	-2.59377692	0.009492806	0.238758449
Glycyrrhiza_uralensis_Fisch0287120.1	-1.30712514	3.731378567	-0.350306225	NA	NA
Glycyrrhiza_uralensis_Fisch0168660.1	-1.306134198	0.588894868	-2.217941211	0.026558841	0.399196096
Glycyrrhiza_uralensis_Fisch0185860.1	-1.302722466	2.993130604	-0.435237428	0.663390098	0.96632848
Glycyrrhiza_uralensis_Fisch0159920.1	-1.302478094	1.731879485	-0.752060467	0.452014697	0.922710496
Glycyrrhiza_uralensis_Fisch0242070.1	-1.301298065	0.741780672	-1.754289527	0.079380909	0.631844738
Glycyrrhiza_uralensis_Fisch0155130.1	-1.301225846	0.921768136	-1.411662863	0.158049253	0.761519387
Glycyrrhiza_uralensis_Fisch0168590.1	-1.299641018	0.879318169	-1.478009967	0.13940511	0.737745126
Glycyrrhiza_uralensis_Fisch0007610.1	-1.299320394	0.541742767	-2.398408384	0.016466495	0.315815568
Glycyrrhiza_uralensis_Fisch0148370.1	-1.298436887	0.882513627	-1.471293867	0.141211661	0.741661163
Glycyrrhiza_uralensis_Fisch0017630.1	-1.296714054	1.733095514	-0.748206918	0.454335359	0.923623358
Glycyrrhiza_uralensis_Fisch0060520.1	-1.295151203	0.550968681	-2.350680259	0.018739129	0.334620502
Glycyrrhiza_uralensis_Fisch0173470.1	-1.293216522	1.005115903	-1.286634226	0.198221808	0.806142846
Glycyrrhiza_uralensis_Fisch0086460.1	-1.292174693	0.383314535	-3.371055815	0.000748807	0.055287671
Glycyrrhiza_uralensis_Fisch0009850.1	-1.290358732	0.500430344	-2.578498183	0.009923082	0.245729
Glycyrrhiza_uralensis_Fisch0278040.1	-1.290134201	0.844412565	-1.527848181	0.126550237	0.72105359
Glycyrrhiza_uralensis_Fisch0	-1.287672765	0.526907	-2.443830	0.014532	0.298966

252150.1		541	589	249	86
Glycyrrhiza_uralensis_Fisch0160830.1	-1.287417767	2.914943698	-0.441661281	0.658734332	0.96632848
Glycyrrhiza_uralensis_Fisch0163300.1	-1.28719899	0.71418423	-1.802334658	0.071492769	0.60422435
Glycyrrhiza_uralensis_Fisch0173250.1	-1.286385944	0.417335729	-3.082376739	0.002053548	0.105784917
Glycyrrhiza_uralensis_Fisch0107000.1	-1.284584009	1.101848865	-1.16584411	0.243677479	0.839393558
Glycyrrhiza_uralensis_Fisch0220750.1	-1.283064013	1.449071641	-0.885438633	0.375920104	0.90447444
Glycyrrhiza_uralensis_Fisch0225800.1	-1.282350319	0.488896704	-2.622947359	0.008717274	0.232899664
Glycyrrhiza_uralensis_Fisch0180280.1	-1.280688206	0.655138387	-1.954836156	0.050602412	0.531413638
Glycyrrhiza_uralensis_Fisch0237400.1	-1.280649348	1.005452379	-1.273704628	0.202768116	0.807975295
Glycyrrhiza_uralensis_Fisch0136840.1	-1.280494495	3.748928176	-0.341562824	0.732679918	0.976483982
Glycyrrhiza_uralensis_Fisch0153200.1	-1.280136547	2.538321836	-0.50432397	0.614033736	0.962115213
Glycyrrhiza_uralensis_Fisch0213350.1	-1.27953038	1.114650411	-1.14792079	0.251001265	0.845365739
Glycyrrhiza_uralensis_Fisch0009340.1	-1.278769335	1.502745539	-0.850955336	0.394794164	0.910082902
Glycyrrhiza_uralensis_Fisch0285550.1	-1.278145278	0.500719481	-2.552617434	0.010691686	0.258123252
Glycyrrhiza_uralensis_Fisch0053180.1	-1.277368255	1.383015135	-0.923611191	0.355688785	0.897807124
Glycyrrhiza_uralensis_Fisch0121620.1	-1.276574435	0.74097369	-1.722833688	0.084918605	0.643781241
Glycyrrhiza_uralensis_Fisch0215970.1	-1.276300379	0.630430176	-2.024491255	0.042919631	0.500278699
Glycyrrhiza_uralensis_Fisch0207000.1	-1.275778582	0.552987054	-2.307067719	0.021051045	0.355079648
Glycyrrhiza_uralensis_Fisch0091460.1	-1.275351337	0.996951034	-1.279251732	0.200808424	0.806142846
Glycyrrhiza_uralensis_Fisch0106810.1	-1.274348527	2.802669218	-0.454691021	0.649331542	0.965935919
Glycyrrhiza_uralensis_Fisch0267800.1	-1.273849591	0.647849963	-1.966272539	0.049267137	0.528259591
Glycyrrhiza_uralensis_Fisch0085440.1	-1.273554416	0.523470041	-2.432907932	0.014978109	0.303670311
Glycyrrhiza_uralensis_Fisch0	-1.272800989	0.850720	-1.496145	0.134615	0.730929

096250.1		282	108	845	9
Glycyrrhiza_uralensis_Fisch0263820.1	-1.272655407	1.554079778	-0.818912533	0.412836319	0.911978305
Glycyrrhiza_uralensis_Fisch0220260.1	-1.2678874	3.561868097	-0.355961357	0.721869505	0.975652703
Glycyrrhiza_uralensis_Fisch0221160.1	-1.26784039	1.458623824	-0.869203127	0.384736037	0.906957344
Glycyrrhiza_uralensis_Fisch0136270.1	-1.267273032	1.177068285	-1.076635101	0.281643316	0.86117142
Glycyrrhiza_uralensis_Fisch0208820.1	-1.267030142	1.258560041	-1.006729994	0.314064544	0.882272787
Glycyrrhiza_uralensis_Fisch0277100.1	-1.265935174	0.631096163	-2.00593071	0.044863641	0.508891538
Glycyrrhiza_uralensis_Fisch0178510.1	-1.265894751	1.856373295	-0.681918208	0.495290671	0.937582768
Glycyrrhiza_uralensis_Fisch0103880.1	-1.265762973	0.864068287	-1.464887663	0.142951569	0.743059364
Glycyrrhiza_uralensis_Fisch0188970.1	-1.26542239	1.416839027	-0.893130671	0.3717872	0.90343083
Glycyrrhiza_uralensis_Fisch0259650.1	-1.264213588	0.410349474	-3.080821758	0.002064302	0.105944019
Glycyrrhiza_uralensis_Fisch0147770.1	-1.264114444	3.800919595	-0.332581212	0.739450428	NA
Glycyrrhiza_uralensis_Fisch0157480.1	-1.263129601	3.147152778	-0.401356302	NA	NA
Glycyrrhiza_uralensis_Fisch0251330.1	-1.261991124	1.095628314	-1.151842379	0.24938585	0.844321666
Glycyrrhiza_uralensis_Fisch0009730.1	-1.261329521	0.302249878	-4.173134924	3.00E-05	0.005240229
Glycyrrhiza_uralensis_Fisch0019190.1	-1.260563298	0.456930331	-2.758764766	0.005802028	0.190785276
Glycyrrhiza_uralensis_Fisch0004980.1	-1.260390107	0.743079232	-1.696171893	0.089853332	0.654987793
Glycyrrhiza_uralensis_Fisch0176030.1	-1.259738233	1.698735718	-0.741573995	0.458345484	0.924102621
Glycyrrhiza_uralensis_Fisch0275230.1	-1.258328244	2.04974514	-0.613894976	0.53928473	0.947110698
Glycyrrhiza_uralensis_Fisch0155740.1	-1.257948081	1.611116565	-0.780792717	0.434924418	0.917168025
Glycyrrhiza_uralensis_Fisch0173490.1	-1.257926281	2.119826641	-0.593409978	0.552906813	0.94916655
Glycyrrhiza_uralensis_Fisch0173350.1	-1.257409304	2.123333326	-0.592186487	0.553725717	0.94916655
Glycyrrhiza_uralensis_Fisch0	-1.25588577	0.734336	-1.710231	0.087223	0.648381

198850.1		56	845	009	048
Glycyrrhiza_uralensis_Fisch0 053280.1	-1.255173834	0.474897 046	-2.643044 095	0.008216 433	0.228635 32
Glycyrrhiza_uralensis_Fisch0 283370.1	-1.254994646	0.820708 835	-1.529159 42	0.126224 928	0.720896 091
Glycyrrhiza_uralensis_Fisch0 065590.1	-1.254905254	0.691293 561	-1.815300 077	0.069477 807	0.600477 853
Glycyrrhiza_uralensis_Fisch0 180130.1	-1.254650041	3.569070 781	-0.351534 088	0.725187 704	NA
Glycyrrhiza_uralensis_Fisch0 227700.1	-1.254650041	3.569070 781	-0.351534 088	0.725187 704	NA
Glycyrrhiza_uralensis_Fisch0 271800.1	-1.254522722	0.409365 324	-3.064555 423	0.002179 938	0.109772 181
Glycyrrhiza_uralensis_Fisch0 107740.1	-1.251576231	0.747003 932	-1.675461 369	0.093843 744	0.664820 202
Glycyrrhiza_uralensis_Fisch0 130550.1	-1.250060055	2.783276 947	-0.449132 472	0.653336 097	0.965950 771
Glycyrrhiza_uralensis_Fisch0 257300.1	-1.249928542	0.627672 437	-1.991370 764	0.046440 141	0.515700 201
Glycyrrhiza_uralensis_Fisch0 028470.1	-1.249052618	1.430381 551	-0.873230 374	0.382537 522	0.906356 743
Glycyrrhiza_uralensis_Fisch0 059390.1	-1.248542058	3.214778 191	-0.388375 802	0.697737 949	NA
Glycyrrhiza_uralensis_Fisch0 262890.1	-1.248219567	0.801892 806	-1.556591 551	0.119567 491	0.711149 478
Glycyrrhiza_uralensis_Fisch0 060070.1	-1.247920787	2.001134 124	-0.623606 77	0.532885 863	0.945913 02
Glycyrrhiza_uralensis_Fisch0 262910.1	-1.246850828	2.121880 858	-0.587615 852	0.556790 169	0.949492 406
Glycyrrhiza_uralensis_Fisch0 172710.1	-1.246595211	0.957203 707	-1.302330 112	0.192803 562	0.800043 93
Glycyrrhiza_uralensis_Fisch0 091150.1	-1.246282759	0.681702 641	-1.828191 186	0.067520 863	0.593148 606
Glycyrrhiza_uralensis_Fisch0 057420.1	-1.243696844	0.536841 174	-2.316694 217	0.020520 395	0.349676 938
Glycyrrhiza_uralensis_Fisch0 033110.1	-1.242681918	2.744754 475	-0.452747 934	0.650730 259	0.965935 919
Glycyrrhiza_uralensis_Fisch0 257880.1	-1.242270822	2.778126 047	-0.447161 432	0.654758 506	0.965983 804
Glycyrrhiza_uralensis_Fisch0 133470.1	-1.241481945	0.703038 613	-1.765880 169	0.077415 955	0.624673 83
Glycyrrhiza_uralensis_Fisch0 211090.1	-1.241263381	0.620198 763	-2.001396 093	0.045349 721	0.509126 769
Glycyrrhiza_uralensis_Fisch0	-1.239306136	1.974013	-0.627810	0.530128	0.945670

252200.1		064	504	084	257
Glycyrrhiza_uralensis_Fisch0 040170.1	-1.239270614	0.319762 997	-3.875591 067	0.000106 366	0.013691 092
Glycyrrhiza_uralensis_Fisch0 100890.1	-1.235949541	0.687606 408	-1.797466 585	0.072261 578	0.606540 739
Glycyrrhiza_uralensis_Fisch0 120800.1	-1.235535122	0.439061 596	-2.814035 965	0.004892 375	0.173686 544
Glycyrrhiza_uralensis_Fisch0 244830.1	-1.235459103	0.408928 174	-3.021212 969	0.002517 642	0.118358 684
Glycyrrhiza_uralensis_Fisch0 195770.1	-1.232584717	1.590410 026	-0.775010 656	0.438333 363	0.918289 88
Glycyrrhiza_uralensis_Fisch0 264700.1	-1.231929075	0.645148 571	-1.909527 713	0.056194 05	0.554099 427
Glycyrrhiza_uralensis_Fisch0 275220.1	-1.231543277	0.778770 237	-1.581394 895	0.113787 774	0.701472 458
Glycyrrhiza_uralensis_Fisch0 008560.1	-1.231179396	0.965710 014	-1.274895 547	0.202346 216	0.807468 172
Glycyrrhiza_uralensis_Fisch0 085230.1	-1.230709128	0.525836 003	-2.340480 912	0.019258 923	0.340002 899
Glycyrrhiza_uralensis_Fisch0 287110.1	-1.230578329	0.604363 639	-2.036155 472	0.041734 746	0.494751 44
Glycyrrhiza_uralensis_Fisch0 124390.1	-1.228657958	3.021892 305	-0.406585 62	0.684312 36	0.969304 92
Glycyrrhiza_uralensis_Fisch0 049410.1	-1.227238152	0.389692 61	-3.149246 667	0.001636 92	0.092152 854
Glycyrrhiza_uralensis_Fisch0 220530.1	-1.226979995	0.690332 838	-1.777374 518	0.075506 64	0.620554 067
Glycyrrhiza_uralensis_Fisch0 131610.1	-1.225708794	0.466371 109	-2.628183 374	0.008584 223	0.231916 014
Glycyrrhiza_uralensis_Fisch0 156630.1	-1.225663454	1.922681 896	-0.637475 943	0.523814 875	0.944922 987
Glycyrrhiza_uralensis_Fisch0 225580.1	-1.225319108	1.688054 899	-0.725876 338	0.467914 584	0.927735 093
Glycyrrhiza_uralensis_Fisch0 086840.1	-1.224992134	0.647547 463	-1.891741 076	0.058525 488	0.563437 26
Glycyrrhiza_uralensis_Fisch0 137400.1	-1.223485711	0.678913 735	-1.802122 491	0.071526 136	0.604294 17
Glycyrrhiza_uralensis_Fisch0 279600.1	-1.222984671	1.273893 665	-0.960036 7	0.337036 745	0.890991 152
Glycyrrhiza_uralensis_Fisch0 218380.1	-1.222814056	0.535303 168	-2.284339 283	0.022351 599	0.365243 031
Glycyrrhiza_uralensis_Fisch0 212670.1	-1.220149356	0.467994 058	-2.607189 843	0.009128 873	0.236747 677
Glycyrrhiza_uralensis_Fisch0	-1.220010902	0.661704	-1.843738	0.065221	0.585118

066610.1		792	956	193	938
Glycyrrhiza_uralensis_Fisch0286800.1	-1.219666122	1.567043854	-0.778322904	0.436378677	0.918112168
Glycyrrhiza_uralensis_Fisch0185100.1	-1.2194375	2.108947403	-0.578220916	0.563114979	0.951408356
Glycyrrhiza_uralensis_Fisch0076450.1	-1.219274629	0.578653052	-2.107090986	0.035109693	0.456066013
Glycyrrhiza_uralensis_Fisch0187820.1	-1.219001604	0.463838305	-2.628074463	0.008586972	0.231916014
Glycyrrhiza_uralensis_Fisch0063230.1	-1.218123723	0.654425159	-1.861364446	0.062692728	0.576463894
Glycyrrhiza_uralensis_Fisch0275180.1	-1.218039842	1.962401062	-0.620688536	0.534804574	0.946315753
Glycyrrhiza_uralensis_Fisch0066970.1	-1.216438733	0.952195468	-1.277509476	0.201422437	0.806142846
Glycyrrhiza_uralensis_Fisch0016970.1	-1.215613728	1.052842	-1.154602237	0.24825335	0.84309081
Glycyrrhiza_uralensis_Fisch0225240.1	-1.215539286	3.364096431	-0.361327123	0.71785492	NA
Glycyrrhiza_uralensis_Fisch0118380.1	-1.214922209	0.808612914	-1.502476881	0.132973994	0.729217799
Glycyrrhiza_uralensis_Fisch0163690.1	-1.214705591	0.603999698	-2.011102979	0.044314581	0.506963858
Glycyrrhiza_uralensis_Fisch0149210.1	-1.214115647	0.472853027	-2.567638521	0.010239387	0.250037179
Glycyrrhiza_uralensis_Fisch0036750.1	-1.213975605	2.572516338	-0.471902	0.636996741	0.964430214
Glycyrrhiza_uralensis_Fisch0100090.1	-1.213920813	0.955876298	-1.269955972	0.204100314	0.808522144
Glycyrrhiza_uralensis_Fisch0070830.1	-1.213691785	1.198443865	-1.012723099	0.311192441	0.880015095
Glycyrrhiza_uralensis_Fisch0036920.1	-1.2132989	0.858432901	-1.413388162	0.157541624	0.761519387
Glycyrrhiza_uralensis_Fisch0062820.1	-1.212076368	0.522406541	-2.320178393	0.020331229	0.347651918
Glycyrrhiza_uralensis_Fisch0287070.1	-1.211305516	0.566350013	-2.138793127	0.032452426	0.440911127
Glycyrrhiza_uralensis_Fisch0234280.1	-1.210850267	1.56633648	-0.773046074	0.439495113	0.918764919
Glycyrrhiza_uralensis_Fisch0188120.1	-1.210133397	0.591684326	-2.045234839	0.040831711	0.491323029
Glycyrrhiza_uralensis_Fisch0191480.1	-1.208457211	0.941987044	-1.282880927	0.199533796	0.806142846
Glycyrrhiza_uralensis_Fisch0	-1.207567377	0.992327	-1.216903	0.223640	0.821475

180180.1		869	621	888	428
Glycyrrhiza_uralensis_Fisch0 191120.1	-1.207412194	0.786878 688	-1.534432 451	0.124923 299	0.719783 949
Glycyrrhiza_uralensis_Fisch0 206580.1	-1.207411237	0.847822 957	-1.424131 332	0.154408 458	0.758447 927
Glycyrrhiza_uralensis_Fisch0 248730.1	-1.206439259	0.564824 986	-2.135952 355	0.032683 298	0.442207 404
Glycyrrhiza_uralensis_Fisch0 104150.1	-1.205533839	0.481258 86	-2.504959 259	0.012246 55	0.273699 587
Glycyrrhiza_uralensis_Fisch0 109830.1	-1.20511028	0.490113 881	-2.458837 278	0.013938 778	0.289729 175
Glycyrrhiza_uralensis_Fisch0 040050.1	-1.204277744	1.047767 029	-1.149375 491	0.250401 182	0.845320 419
Glycyrrhiza_uralensis_Fisch0 193410.1	-1.20209024	1.150333 734	-1.044992 6	0.296026 39	0.869761 143
Glycyrrhiza_uralensis_Fisch0 244200.1	-1.200878433	0.818152 635	-1.467792 661	0.142160 552	0.742901 537
Glycyrrhiza_uralensis_Fisch0 007630.1	-1.196055316	0.478360 124	-2.500324 039	0.012407 976	0.276025 851
Glycyrrhiza_uralensis_Fisch0 084480.1	-1.195197718	0.462376 666	-2.584900 592	0.009740 708	0.243214 558
Glycyrrhiza_uralensis_Fisch0 124400.1	-1.194400102	3.932603 479	-0.303717 399	0.761343 201	NA
Glycyrrhiza_uralensis_Fisch0 028340.1	-1.194400102	3.932603 479	-0.303717 399	0.761343 201	NA
Glycyrrhiza_uralensis_Fisch0 247730.1	-1.193021302	0.569910 979	-2.093346 763	0.036318 211	0.464664 696
Glycyrrhiza_uralensis_Fisch0 031680.1	-1.192232683	0.709549 725	-1.680266 57	0.092905 462	0.662056 741
Glycyrrhiza_uralensis_Fisch0 190390.1	-1.191835546	0.480592 273	-2.479930 729	0.013140 791	0.282914 881
Glycyrrhiza_uralensis_Fisch0 023940.1	-1.190746679	0.721163 822	-1.651145 889	0.098708 789	0.679611 142
Glycyrrhiza_uralensis_Fisch0 115700.1	-1.190043604	2.731142 215	-0.435731 101	0.663031 838	0.966328 48
Glycyrrhiza_uralensis_Fisch0 101550.1	-1.189919004	0.816442 362	-1.457443 979	0.144993 865	0.744212 491
Glycyrrhiza_uralensis_Fisch0 243280.1	-1.187913626	3.402941 205	-0.349084 382	0.727025 962	NA
Glycyrrhiza_uralensis_Fisch0 189550.1	-1.187749309	1.691281 839	-0.702277 575	0.482506 076	0.932545 92
Glycyrrhiza_uralensis_Fisch0 132800.1	-1.187488728	2.206881 434	-0.538084 516	0.590518 702	0.956703 673
Glycyrrhiza_uralensis_Fisch0	-1.187477142	0.436293	-2.721737	0.006493	0.200912

285000.1		777	515	97	415
Glycyrrhiza_uralensis_Fisch0266800.1	-1.187090725	2.615072765	-0.453941757	0.649870748	0.965935919
Glycyrrhiza_uralensis_Fisch0185290.1	-1.185557813	0.705127933	-1.681337184	0.092697439	0.661782054
Glycyrrhiza_uralensis_Fisch0015020.1	-1.185158269	0.547341257	-2.165300447	0.030364684	0.427929048
Glycyrrhiza_uralensis_Fisch0075070.1	-1.185008901	0.709375534	-1.670495872	0.094821295	0.667939298
Glycyrrhiza_uralensis_Fisch0053250.1	-1.184906543	1.156061079	-1.024951504	0.305386071	0.876015102
Glycyrrhiza_uralensis_Fisch0020700.1	-1.183453904	0.680471669	-1.739167048	0.082005384	0.637960598
Glycyrrhiza_uralensis_Fisch0039780.1	-1.182141341	0.412393473	-2.866537468	0.00414989	0.157800727
Glycyrrhiza_uralensis_Fisch0129450.1	-1.180495862	0.399555539	-2.954522583	0.003131532	0.135346245
Glycyrrhiza_uralensis_Fisch0278270.1	-1.179877496	1.729674105	-0.682138614	0.495151306	0.937582768
Glycyrrhiza_uralensis_Fisch0008140.1	-1.179867972	0.660571881	-1.786131089	0.074078036	0.614635752
Glycyrrhiza_uralensis_Fisch0121060.1	-1.177448454	0.674232921	-1.74635266	0.080749691	0.635999849
Glycyrrhiza_uralensis_Fisch0268420.1	-1.17669153	0.876396805	-1.342646988	0.179386308	0.789067991
Glycyrrhiza_uralensis_Fisch0279420.1	-1.176462055	1.528418572	-0.769725046	0.44146301	0.919801681
Glycyrrhiza_uralensis_Fisch0046530.1	-1.176368917	0.947696979	-1.241292252	0.214497806	0.814589934
Glycyrrhiza_uralensis_Fisch0267600.1	-1.175444844	0.902358072	-1.302636814	0.192698783	0.80004393
Glycyrrhiza_uralensis_Fisch0005420.1	-1.175001931	0.893687527	-1.314779379	0.18858406	0.797474632
Glycyrrhiza_uralensis_Fisch0103080.1	-1.174700674	2.129904754	-0.551527326	0.58127224	0.95408193
Glycyrrhiza_uralensis_Fisch0116020.1	-1.17438253	0.823196324	-1.426612942	0.153691492	0.758333908
Glycyrrhiza_uralensis_Fisch0078940.1	-1.174055573	0.499720169	-2.349426028	0.01880238	0.334719555
Glycyrrhiza_uralensis_Fisch0204250.1	-1.173792729	0.647138555	-1.813819807	0.069705472	0.600786806
Glycyrrhiza_uralensis_Fisch0192980.1	-1.171956783	0.515300037	-2.27431923	0.0229468	0.369126803
Glycyrrhiza_uralensis_Fisch0	-1.171485544	1.313814	-0.891667	0.372571	0.903430

013530.1		891	123	38	83
Glycyrrhiza_uralensis_Fisch0 070810.1	-1.171450706	2.120196 344	-0.552519 916	0.580592 193	0.953719 654
Glycyrrhiza_uralensis_Fisch0 061720.1	-1.170463652	0.495685 403	-2.361303 452	0.018210 822	0.331111 164
Glycyrrhiza_uralensis_Fisch0 164270.1	-1.169256234	0.581795 204	-2.009738 525	0.044458 87	0.507890 369
Glycyrrhiza_uralensis_Fisch0 284280.1	-1.168393255	1.703925 952	-0.685706 591	0.492898 15	0.936981 399
Glycyrrhiza_uralensis_Fisch0 257020.1	-1.167775459	0.909550 927	-1.283903 324	0.199175 784	0.806142 846
Glycyrrhiza_uralensis_Fisch0 273680.1	-1.167656766	0.439923 706	-2.654225 605	0.007949 066	0.225098 847
Glycyrrhiza_uralensis_Fisch0 117740.1	-1.167655514	0.931421 098	-1.253627 941	0.209977 27	0.811966 832
Glycyrrhiza_uralensis_Fisch0 286210.1	-1.167302554	3.933830 146	-0.296734 356	0.766669 326	NA
Glycyrrhiza_uralensis_Fisch0 179040.1	-1.166987647	0.636256 995	-1.834145 096	0.066632 459	0.591169 659
Glycyrrhiza_uralensis_Fisch0 195170.1	-1.165030121	0.418330 127	-2.784953 903	0.005353 533	0.182779 5
Glycyrrhiza_uralensis_Fisch0 127030.1	-1.164748926	0.850073 054	-1.370175 094	0.170632 251	0.778850 891
Glycyrrhiza_uralensis_Fisch0 233990.1	-1.16464845	1.073851 655	-1.084552 456	0.278119 922	0.859950 143
Glycyrrhiza_uralensis_Fisch0 092050.1	-1.164501147	0.690432 559	-1.686625 481	0.091675 389	0.659284 916
Glycyrrhiza_uralensis_Fisch0 141650.1	-1.164087794	1.265224 305	-0.920064 363	0.357539 126	0.897807 124
Glycyrrhiza_uralensis_Fisch0 020850.1	-1.163764834	2.875785 717	-0.404677 173	0.685714 823	0.969389 236
Glycyrrhiza_uralensis_Fisch0 233630.1	-1.163625796	0.622222 038	-1.870113 438	0.061468 067	0.572797 664
Glycyrrhiza_uralensis_Fisch0 252220.1	-1.162942386	0.704701 17	-1.650263 169	0.098889 122	0.679653 036
Glycyrrhiza_uralensis_Fisch0 092300.1	-1.162782356	0.666171 899	-1.745468 937	0.080903 275	0.636553 36
Glycyrrhiza_uralensis_Fisch0 095090.1	-1.162660106	0.430611 755	-2.700019 43	0.006933 543	0.208047 509
Glycyrrhiza_uralensis_Fisch0 077820.1	-1.16188566	3.831235 133	-0.303266 602	0.761686 696	NA
Glycyrrhiza_uralensis_Fisch0 199940.1	-1.16186477	0.452745 364	-2.566265 416	0.010280 013	0.250699 009
Glycyrrhiza_uralensis_Fisch0	-1.161398233	1.051249	-1.104778	0.269255	0.855452

093430.1		544	822	439	31
Glycyrrhiza_uralensis_Fisch0 015900.1	-1.160585684	3.865918 086	-0.300209 59	0.764017 29	NA
Glycyrrhiza_uralensis_Fisch0 151260.1	-1.160167003	1.725436 243	-0.672390 538	0.501335 105	0.938792 103
Glycyrrhiza_uralensis_Fisch0 136470.1	-1.159810843	3.828444 262	-0.302945 73	0.761931 22	NA
Glycyrrhiza_uralensis_Fisch0 048460.1	-1.159810843	3.828444 262	-0.302945 73	0.761931 22	NA
Glycyrrhiza_uralensis_Fisch0 111500.1	-1.159056968	1.652770 207	-0.701281 378	0.483127 433	0.932636 918
Glycyrrhiza_uralensis_Fisch0 252760.1	-1.158507006	2.061666 412	-0.561927 477	0.574165 432	0.952518 696
Glycyrrhiza_uralensis_Fisch0 131220.1	-1.158171524	3.934248 639	-0.294381 883	0.768466 109	NA
Glycyrrhiza_uralensis_Fisch0 132260.1	-1.157926818	1.532482 653	-0.755588 858	0.449895 738	0.922710 496
Glycyrrhiza_uralensis_Fisch0 237200.1	-1.157829129	1.322290 059	-0.875624 165	0.381234 384	0.906067 955
Glycyrrhiza_uralensis_Fisch0 251730.1	-1.157418896	3.929123 949	-0.294574 289	0.768319 105	NA
Glycyrrhiza_uralensis_Fisch0 114590.1	-1.157418896	3.929123 949	-0.294574 289	0.768319 105	NA
Glycyrrhiza_uralensis_Fisch0 240880.1	-1.156775694	0.596449 477	-1.939436 179	0.052448 249	0.538262 101
Glycyrrhiza_uralensis_Fisch0 130920.1	-1.156683378	1.062004 485	-1.089151 124	0.276087 248	0.859487 559
Glycyrrhiza_uralensis_Fisch0 124150.1	-1.155970093	0.530036 431	-2.180925 736	0.029188 91	0.420955 976
Glycyrrhiza_uralensis_Fisch0 125130.1	-1.155692062	1.466318 014	-0.788159 22	0.430603 579	0.916951 181
Glycyrrhiza_uralensis_Fisch0 114760.1	-1.1545924	0.435496 898	-2.651206 942	0.008020 468	0.225746 701
Glycyrrhiza_uralensis_Fisch0 132690.1	-1.153986208	0.588279 643	-1.961628 66	0.049805 733	0.529504 363
Glycyrrhiza_uralensis_Fisch0 083100.1	-1.153675758	0.670866 835	-1.719679 223	0.085490 767	0.643781 241
Glycyrrhiza_uralensis_Fisch0 255340.1	-1.15346611	0.629923 065	-1.831122 203	0.067082 305	0.592340 664
Glycyrrhiza_uralensis_Fisch0 267390.1	-1.152956025	1.717462 024	-0.671313 839	0.502020 621	0.938792 103
Glycyrrhiza_uralensis_Fisch0 181680.1	-1.152722498	0.495989 59	-2.324086 072	0.020120 881	0.347491 768
Glycyrrhiza_uralensis_Fisch0	-1.152622215	0.821002	-1.403919	0.160342	0.764868

113920.1		865	845	719	42
Glycyrrhiza_uralensis_Fisch0<br>157810.1	-1.152124974	3.707527<br>831	-0.310752<br>886	0.755988<br>489	NA
Glycyrrhiza_uralensis_Fisch0<br>202730.1	-1.152096399	0.619516<br>508	-1.859670<br>218	0.062932<br>198	0.577026<br>58
Glycyrrhiza_uralensis_Fisch0<br>234660.1	-1.152035134	1.556248<br>132	-0.740264<br>428	0.459139<br>561	0.924102<br>621
Glycyrrhiza_uralensis_Fisch0<br>229700.1	-1.148487619	0.401675<br>547	-2.859242<br>061	0.004246<br>546	0.159994<br>804
Glycyrrhiza_uralensis_Fisch0<br>043500.1	-1.148089008	0.412708<br>622	-2.781839<br>163	0.005405<br>182	0.183738<br>664
Glycyrrhiza_uralensis_Fisch0<br>116530.1	-1.148016302	0.632960<br>063	-1.813726<br>281	0.069719<br>877	0.600786<br>806
Glycyrrhiza_uralensis_Fisch0<br>250480.1	-1.145738916	0.415691<br>071	-2.756226<br>906	0.005847<br>242	0.191516<br>571
Glycyrrhiza_uralensis_Fisch0<br>260180.1	-1.145695419	0.487295<br>376	-2.351131<br>315	0.018716<br>427	0.334620<br>502
Glycyrrhiza_uralensis_Fisch0<br>207090.1	-1.145628265	0.378231<br>923	-3.028904<br>209	0.002454<br>425	0.116986<br>155
Glycyrrhiza_uralensis_Fisch0<br>037590.1	-1.145505123	0.777064<br>749	-1.474143<br>724	0.140442<br>898	0.740482<br>679
Glycyrrhiza_uralensis_Fisch0<br>083680.1	-1.145322632	3.432596<br>446	-0.333660<br>729	0.738635<br>587	NA
Glycyrrhiza_uralensis_Fisch0<br>195210.1	-1.144757104	1.171252<br>637	-0.977378<br>464	0.328381<br>821	0.887225<br>647
Glycyrrhiza_uralensis_Fisch0<br>196980.1	-1.143823348	0.875984<br>281	-1.305757<br>846	0.191634<br>92	0.798798<br>603
Glycyrrhiza_uralensis_Fisch0<br>179480.1	-1.14379641	2.964709<br>311	-0.385803<br>899	0.699641<br>908	0.972279<br>672
Glycyrrhiza_uralensis_Fisch0<br>111340.1	-1.143414568	0.705031<br>06	-1.621793<br>183	0.104847<br>639	0.692097<br>679
Glycyrrhiza_uralensis_Fisch0<br>127100.1	-1.141778704	0.306947<br>461	-3.719785<br>462	0.000199<br>392	0.021815<br>306
Glycyrrhiza_uralensis_Fisch0<br>109870.1	-1.141729221	0.981355<br>417	-1.163420<br>714	0.244658<br>849	0.839393<br>558
Glycyrrhiza_uralensis_Fisch0<br>035560.1	-1.140556026	0.842932<br>519	-1.353081<br>059	0.176029<br>736	0.785494<br>318
Glycyrrhiza_uralensis_Fisch0<br>141050.1	-1.13940251	3.929937<br>725	-0.289928<br>897	0.771870<br>634	NA
Glycyrrhiza_uralensis_Fisch0<br>172160.1	-1.138816411	0.335937<br>837	-3.389961<br>726	0.000699<br>024	0.052415<br>899
Glycyrrhiza_uralensis_Fisch0<br>268450.1	-1.138519054	0.420922<br>265	-2.704820<br>223	0.006834<br>136	0.207437<br>156
Glycyrrhiza_uralensis_Fisch0	-1.138470974	3.796587	-0.299866	0.764278	NA

139900.1		249	933	658	
Glycyrrhiza_uralensis_Fisch0 057560.1	-1.137873552	0.612403 414	-1.858045 738	0.063162 519	0.577986 481
Glycyrrhiza_uralensis_Fisch0 095400.1	-1.137840805	0.494317 703	-2.301841 1	0.021344 135	0.357517 972
Glycyrrhiza_uralensis_Fisch0 216430.1	-1.137176944	0.925863 283	-1.228234 195	0.219359 066	0.817830 773
Glycyrrhiza_uralensis_Fisch0 183330.1	-1.13710862	0.573672 329	-1.982156 994	0.047461 672	0.521406 87
Glycyrrhiza_uralensis_Fisch0 058830.1	-1.137054317	0.390608 897	-2.910979 049	0.003602 982	0.147489 406
Glycyrrhiza_uralensis_Fisch0 269820.1	-1.136331047	0.666953 229	-1.703764 218	0.088425 145	0.651283 123
Glycyrrhiza_uralensis_Fisch0 252000.1	-1.135566432	0.665472 485	-1.706406 286	0.087932 461	0.650152 769
Glycyrrhiza_uralensis_Fisch0 178200.1	-1.13523072	0.502651 295	-2.258485 617	0.023915 4	0.377982 265
Glycyrrhiza_uralensis_Fisch0 032120.1	-1.13487306	0.850647 292	-1.334128 811	0.182161 645	0.791863 958
Glycyrrhiza_uralensis_Fisch0 109790.1	-1.133434351	2.710838 312	-0.418112 119	0.675865 145	NA
Glycyrrhiza_uralensis_Fisch0 043180.1	-1.132708909	0.809844 176	-1.398675 132	0.161910 425	0.767546 855
Glycyrrhiza_uralensis_Fisch0 234050.1	-1.132684495	0.445941 075	-2.539986 913	0.011085 662	0.262360 4
Glycyrrhiza_uralensis_Fisch0 275240.1	-1.131801954	2.100151 339	-0.538914 474	0.589945 87	0.956703 673
Glycyrrhiza_uralensis_Fisch0 265530.1	-1.131568264	0.440127 948	-2.570998 434	0.010140 579	0.248811 137
Glycyrrhiza_uralensis_Fisch0 128450.1	-1.131423057	0.362970 897	-3.117117 831	0.001826 286	0.098783 584
Glycyrrhiza_uralensis_Fisch0 118940.1	-1.131112876	0.996803 756	-1.134739 781	0.256484 364	0.847400 43
Glycyrrhiza_uralensis_Fisch0 275750.1	-1.130902025	0.584541 671	-1.934681 618	0.053029 378	0.542233 27
Glycyrrhiza_uralensis_Fisch0 165960.1	-1.130816371	0.453602 163	-2.492969 529	0.012667 972	0.278463 999
Glycyrrhiza_uralensis_Fisch0 185670.1	-1.130604049	0.468923 545	-2.411062 661	0.015906 115	0.313410 208
Glycyrrhiza_uralensis_Fisch0 100560.1	-1.129479891	1.196695 327	-0.943832 458	0.345255 271	0.894851 545
Glycyrrhiza_uralensis_Fisch0 050050.1	-1.127425617	0.962307 137	-1.171586 049	0.241363 292	0.837396 698
Glycyrrhiza_uralensis_Fisch0	-1.126858468	3.077799	-0.366124	0.714271	0.974505

188670.1		035	771	974	628
Glycyrrhiza_uralensis_Fisch0095320.1	-1.125664242	1.356972407	-0.829540996	0.406798349	0.910802928
Glycyrrhiza_uralensis_Fisch0270070.1	-1.124548147	0.447909135	-2.510661334	0.012050524	0.271842662
Glycyrrhiza_uralensis_Fisch0014040.1	-1.124108399	0.374376668	-3.002613402	0.002676722	0.122955544
Glycyrrhiza_uralensis_Fisch0020990.1	-1.124105962	0.470146311	-2.390970501	0.016803903	0.318731241
Glycyrrhiza_uralensis_Fisch0193920.1	-1.122780915	3.268745547	-0.343489849	0.731229976	NA
Glycyrrhiza_uralensis_Fisch0283680.1	-1.122724793	0.666232432	-1.685184838	0.091952915	0.66060154
Glycyrrhiza_uralensis_Fisch0003300.1	-1.122392062	1.00205778	-1.120087169	0.262676618	0.851289776
Glycyrrhiza_uralensis_Fisch0137330.1	-1.121431585	1.484733134	-0.755308519	0.450063888	0.922710496
Glycyrrhiza_uralensis_Fisch0211800.1	-1.121190318	3.930770564	-0.285234231	0.775464697	NA
Glycyrrhiza_uralensis_Fisch0065050.1	-1.120509769	1.042867433	-1.074450821	0.282620675	0.86117142
Glycyrrhiza_uralensis_Fisch0112290.1	-1.119404178	0.625881273	-1.788524798	0.073691378	0.613222014
Glycyrrhiza_uralensis_Fisch0249850.1	-1.119258471	0.239380954	-4.675637112	2.93E-06	0.000860187
Glycyrrhiza_uralensis_Fisch0186880.1	-1.119171793	0.92646646	-1.208000334	0.227047134	0.824173095
Glycyrrhiza_uralensis_Fisch0126140.1	-1.119160836	2.260240094	-0.495151307	0.62049332	0.962594079
Glycyrrhiza_uralensis_Fisch0133240.1	-1.118983562	0.692741799	-1.615296731	0.106246451	0.692097679
Glycyrrhiza_uralensis_Fisch0265260.1	-1.118453673	1.094826732	-1.02158053	0.306979477	0.876615969
Glycyrrhiza_uralensis_Fisch0267100.1	-1.117899447	1.208519676	-0.925015512	0.354957839	0.897677342
Glycyrrhiza_uralensis_Fisch0186610.1	-1.117765209	0.514148038	-2.174014341	0.029704058	0.424694705
Glycyrrhiza_uralensis_Fisch0244270.1	-1.117374328	0.797793724	-1.400580495	0.161339557	0.766801031
Glycyrrhiza_uralensis_Fisch0059530.1	-1.117096223	2.755023789	-0.405476071	0.685127604	0.969332592
Glycyrrhiza_uralensis_Fisch0280780.1	-1.116992448	0.396867349	-2.814523422	0.004884962	0.173686544
Glycyrrhiza_uralensis_Fisch0	-1.116687448	0.424088	-2.633149	0.008459	0.230867

146920.1		136	462	711	625
Glycyrrhiza_uralensis_Fisch0282180.1	-1.116526589	1.397185125	-0.799125735	0.424217509	0.915195846
Glycyrrhiza_uralensis_Fisch0056100.1	-1.116404666	3.013363377	-0.37048458	0.711021464	0.974179385
Glycyrrhiza_uralensis_Fisch0006030.1	-1.115629038	0.515233807	-2.165286949	0.030365717	0.427929048
Glycyrrhiza_uralensis_Fisch0043520.1	-1.115101031	0.392277594	-2.84263248	0.004474263	0.164772503
Glycyrrhiza_uralensis_Fisch0012910.1	-1.115099072	0.516216618	-2.160137882	0.030761997	0.429546783
Glycyrrhiza_uralensis_Fisch0022620.1	-1.11466052	1.081196378	-1.030951031	0.302563784	0.87348346
Glycyrrhiza_uralensis_Fisch0021720.1	-1.113681493	0.889750839	-1.251677936	0.210687243	0.812109091
Glycyrrhiza_uralensis_Fisch0190150.1	-1.113516479	0.512586012	-2.1723505	0.029829236	0.425218763
Glycyrrhiza_uralensis_Fisch0013870.1	-1.113274031	0.638003858	-1.744933072	0.08099652	0.63655336
Glycyrrhiza_uralensis_Fisch0016300.1	-1.112695112	0.393294039	-2.829168514	0.004666912	0.169686659
Glycyrrhiza_uralensis_Fisch0021370.1	-1.112179099	2.207574302	-0.503801434	0.61440092	0.962115213
Glycyrrhiza_uralensis_Fisch0178450.1	-1.111356633	0.375251802	-2.961629038	0.003060162	0.135232055
Glycyrrhiza_uralensis_Fisch0258480.1	-1.110370362	0.538085803	-2.063556323	0.039059802	0.481530651
Glycyrrhiza_uralensis_Fisch0221020.1	-1.110213054	0.509474778	-2.179132516	0.029321825	0.422325846
Glycyrrhiza_uralensis_Fisch0068160.1	-1.109837078	0.599069002	-1.852603081	0.063939271	0.580899115
Glycyrrhiza_uralensis_Fisch0120610.1	-1.10942195	3.931314252	-0.282201289	0.77778917	NA
Glycyrrhiza_uralensis_Fisch0112210.1	-1.107104595	0.589845884	-1.876938749	0.060526489	0.571546723
Glycyrrhiza_uralensis_Fisch0005710.1	-1.106457224	0.815867936	-1.356171967	0.175044459	0.784165295
Glycyrrhiza_uralensis_Fisch0212310.1	-1.105223211	0.587544897	-1.881087243	0.059960052	0.568651873
Glycyrrhiza_uralensis_Fisch0276010.1	-1.104979011	1.363660411	-0.810303652	0.417765677	0.913280981
Glycyrrhiza_uralensis_Fisch0093640.1	-1.104352156	0.402637067	-2.742798032	0.006091814	0.194366451
Glycyrrhiza_uralensis_Fisch0	-1.103627772	2.027484	-0.544333	0.586212	0.955776

045310.1		913	408	062	219
Glycyrrhiza_uralensis_Fisch0 069190.1	-1.102836909	1.105592 009	-0.997508 032	0.318517 977	0.885080 409
Glycyrrhiza_uralensis_Fisch0 261790.1	-1.10275999	0.504332 084	-2.186575 125	0.028773 555	0.417677 746
Glycyrrhiza_uralensis_Fisch0 172030.1	-1.102304048	0.332531 694	-3.314884 164	0.000916 811	0.063412 766
Glycyrrhiza_uralensis_Fisch0 175580.1	-1.101567478	0.862400 333	-1.277327 287	0.201486 724	0.806142 846
Glycyrrhiza_uralensis_Fisch0 170270.1	-1.098621395	0.385734 954	-2.848125 072	0.004397 764	0.163426 587
Glycyrrhiza_uralensis_Fisch0 104740.1	-1.097950895	2.034146 447	-0.539760 005	0.589362 553	0.956703 673
Glycyrrhiza_uralensis_Fisch0 065810.1	-1.097802059	3.829902 462	-0.286639 691	0.774388 22	NA
Glycyrrhiza_uralensis_Fisch0 277640.1	-1.097711846	3.864207 971	-0.284071 627	0.776355 49	NA
Glycyrrhiza_uralensis_Fisch0 025720.1	-1.097570403	3.931866 207	-0.279147 444	0.780131 674	NA
Glycyrrhiza_uralensis_Fisch0 282850.1	-1.097235886	0.561647 635	-1.953601 899	0.050748 316	0.532279 959
Glycyrrhiza_uralensis_Fisch0 069280.1	-1.096930334	1.125690 613	-0.974450 992	0.329832 661	0.887225 647
Glycyrrhiza_uralensis_Fisch0 146120.1	-1.096484114	2.194438 657	-0.499664 965	0.617311 006	0.962288 147
Glycyrrhiza_uralensis_Fisch0 221730.1	-1.096453341	0.832457 095	-1.317128 952	0.187795 404	0.797474 632
Glycyrrhiza_uralensis_Fisch0 116510.1	-1.096144401	0.650599 971	-1.684820 858	0.092023 139	0.660601 54
Glycyrrhiza_uralensis_Fisch0 054020.1	-1.096073094	0.571168 242	-1.919002 168	0.054984 059	0.549803 889
Glycyrrhiza_uralensis_Fisch0 027650.1	-1.095098145	1.260303 803	-0.868916 004	0.384893 075	0.906957 344
Glycyrrhiza_uralensis_Fisch0 073790.1	-1.094662971	0.371765 07	-2.944501 947	0.003234 749	0.137365 504
Glycyrrhiza_uralensis_Fisch0 183530.1	-1.094660892	0.572727 913	-1.911310 532	0.055964 686	0.553093 989
Glycyrrhiza_uralensis_Fisch0 089430.1	-1.093086366	0.461203 77	-2.370072 485	0.017784 598	0.326855 015
Glycyrrhiza_uralensis_Fisch0 250400.1	-1.092828313	0.842490 525	-1.297140 181	0.194582 958	0.801825 957
Glycyrrhiza_uralensis_Fisch0 276640.1	-1.092633524	1.442303 113	-0.757561 648	0.448713 447	0.922628 42
Glycyrrhiza_uralensis_Fisch0	-1.092577338	0.454678	-2.402964	0.016262	0.315198

245490.1		97	311	775	656
Glycyrrhiza_uralensis_Fisch0160000.1	-1.091470995	0.589485934	-1.851564104	0.064088442	0.581677522
Glycyrrhiza_uralensis_Fisch0083730.1	-1.091125417	0.345256012	-3.160337197	0.001575866	0.090744272
Glycyrrhiza_uralensis_Fisch0244900.1	-1.090945693	1.289823493	-0.845810066	0.397658709	0.910230427
Glycyrrhiza_uralensis_Fisch0166020.1	-1.090466968	0.827362269	-1.318004227	0.187502233	0.797211647
Glycyrrhiza_uralensis_Fisch0162740.1	-1.090246518	0.853469062	-1.277429455	0.201450671	0.806142846
Glycyrrhiza_uralensis_Fisch0137520.1	-1.090038379	1.035805519	-1.05235815	0.292635262	0.867668238
Glycyrrhiza_uralensis_Fisch0192100.1	-1.089729524	1.174698707	-0.927667254	0.353580199	0.897364869
Glycyrrhiza_uralensis_Fisch0256520.1	-1.089726107	0.569953055	-1.911957656	0.055881625	0.553093989
Glycyrrhiza_uralensis_Fisch0079280.1	-1.089321317	0.534425598	-2.038303033	0.041519637	0.49475144
Glycyrrhiza_uralensis_Fisch0053270.1	-1.088991914	0.836562512	-1.301746012	0.193003225	0.80004393
Glycyrrhiza_uralensis_Fisch0214060.1	-1.088860698	1.058658529	-1.028528717	0.30370119	0.874100786
Glycyrrhiza_uralensis_Fisch0184220.1	-1.088764504	1.691824387	-0.643544633	0.519870763	0.943762067
Glycyrrhiza_uralensis_Fisch0144200.1	-1.088449192	1.231837189	-0.883598256	0.376913121	0.904607774
Glycyrrhiza_uralensis_Fisch0184230.1	-1.088137601	0.553511519	-1.96588068	0.049312395	0.528259591
Glycyrrhiza_uralensis_Fisch0075680.1	-1.087121489	0.473886172	-2.294056155	0.021787273	0.361668738
Glycyrrhiza_uralensis_Fisch0062330.1	-1.087080201	0.508313906	-2.138600163	0.032468064	0.440911127
Glycyrrhiza_uralensis_Fisch0057070.1	-1.086486348	0.520434815	-2.087651163	0.036829313	0.468047291
Glycyrrhiza_uralensis_Fisch0075610.1	-1.086434149	0.541343561	-2.006921719	0.044757999	0.508412002
Glycyrrhiza_uralensis_Fisch0235350.1	-1.085117342	0.641757998	-1.690851297	0.090865207	0.657386695
Glycyrrhiza_uralensis_Fisch0254590.1	-1.084364348	0.41490964	-2.613495187	0.008962135	0.235243816
Glycyrrhiza_uralensis_Fisch0039740.1	-1.083823932	0.420090733	-2.579975816	0.009880723	0.24518455
Glycyrrhiza_uralensis_Fisch0	-1.083754149	0.374351	-2.895014	0.003791	0.150097

069790.1		916	301	413	535
Glycyrrhiza_uralensis_Fisch0124220.1	-1.083679498	0.70197374	-1.543760737	0.122646306	0.714644751
Glycyrrhiza_uralensis_Fisch0093380.1	-1.083229374	1.437990596	-0.753293782	0.451273395	0.922710496
Glycyrrhiza_uralensis_Fisch0131900.1	-1.082823526	1.064664982	-1.017055641	0.309126961	0.877922929
Glycyrrhiza_uralensis_Fisch0256910.1	-1.082556145	0.984110674	-1.10003496	0.27131689	0.856901135
Glycyrrhiza_uralensis_Fisch0103090.1	-1.082330826	0.938427366	-1.153345336	0.248768669	0.843479625
Glycyrrhiza_uralensis_Fisch0271150.1	-1.080972097	0.520221377	-2.077907878	0.037717845	0.474697791
Glycyrrhiza_uralensis_Fisch0069490.1	-1.080837494	0.793287043	-1.362479703	0.173046545	0.780447701
Glycyrrhiza_uralensis_Fisch0230460.1	-1.080497089	3.133072277	-0.34486823	0.730193436	0.97648339
Glycyrrhiza_uralensis_Fisch0062600.1	-1.080104126	0.760192029	-1.420830639	0.155366001	0.75874765
Glycyrrhiza_uralensis_Fisch0061670.1	-1.078960775	0.417025213	-2.587279477	0.009673709	0.242512749
Glycyrrhiza_uralensis_Fisch0027230.1	-1.078916407	0.441170233	-2.445578431	0.014461999	0.297776147
Glycyrrhiza_uralensis_Fisch0246800.1	-1.078626616	0.786352862	-1.371682698	0.170162237	0.778850891
Glycyrrhiza_uralensis_Fisch0111010.1	-1.078393273	0.804573339	-1.340329365	0.180138289	0.789067991
Glycyrrhiza_uralensis_Fisch0201920.1	-1.077580265	0.814002606	-1.323804442	0.185568001	0.793924951
Glycyrrhiza_uralensis_Fisch0256020.1	-1.076396911	0.64802684	-1.661037545	0.096705907	0.673652444
Glycyrrhiza_uralensis_Fisch0138300.1	-1.076021746	0.659727286	-1.631009917	0.102888227	0.688261177
Glycyrrhiza_uralensis_Fisch0229410.1	-1.075411162	1.671174959	-0.643506029	0.519895804	0.943762067
Glycyrrhiza_uralensis_Fisch0112240.1	-1.075303371	0.669201211	-1.606846124	0.108088127	0.69307452
Glycyrrhiza_uralensis_Fisch0124530.1	-1.074417281	0.297011297	-3.61742901	0.000297544	0.02911334
Glycyrrhiza_uralensis_Fisch0270760.1	-1.074319689	0.506356119	-2.121668226	0.033865609	0.451283183
Glycyrrhiza_uralensis_Fisch0054140.1	-1.074029756	0.35672609	-3.010796761	0.002605632	0.120379195
Glycyrrhiza_uralensis_Fisch0	-1.073933685	0.558583	-1.922602	0.054529	0.548718

110720.1		214	859	945	966
Glycyrrhiza_uralensis_Fisch0218850.1	-1.073879247	0.382686778	-2.806157176	0.005013623	0.176538318
Glycyrrhiza_uralensis_Fisch0036270.1	-1.07205074	0.816895533	-1.312347414	0.189402941	0.797474632
Glycyrrhiza_uralensis_Fisch0193160.1	-1.070900096	0.491851651	-2.177282711	0.02945948	0.422462527
Glycyrrhiza_uralensis_Fisch0068310.1	-1.070521831	0.390338916	-2.742544459	0.00609652	0.194366451
Glycyrrhiza_uralensis_Fisch0203640.1	-1.070239833	0.337981929	-3.166559337	0.001542539	0.089540967
Glycyrrhiza_uralensis_Fisch0270690.1	-1.069898323	0.4387273	-2.438640868	0.014742611	0.300454876
Glycyrrhiza_uralensis_Fisch0181030.1	-1.069768391	2.8071261	-0.381090251	0.703136281	0.973348236
Glycyrrhiza_uralensis_Fisch0178130.1	-1.069105992	1.239932123	-0.86222945	0.388561267	0.908584712
Glycyrrhiza_uralensis_Fisch0011300.1	-1.068620404	0.517911901	-2.063324672	0.039081791	0.481530651
Glycyrrhiza_uralensis_Fisch0200050.1	-1.068564349	0.441993303	-2.417603033	0.01562311	0.31188596
Glycyrrhiza_uralensis_Fisch0049290.1	-1.067857055	0.951506119	-1.122280807	0.261743064	0.850912419
Glycyrrhiza_uralensis_Fisch0248520.1	-1.065955605	0.855727368	-1.24567198	0.212884845	0.813960263
Glycyrrhiza_uralensis_Fisch0148030.1	-1.06585431	0.396573548	-2.687658611	0.007195492	0.211730431
Glycyrrhiza_uralensis_Fisch0202410.1	-1.0658339	1.194535568	-0.892257986	0.372254668	0.90343083
Glycyrrhiza_uralensis_Fisch0189610.1	-1.065684177	0.259579002	-4.105432913	4.04E-05	0.00639056
Glycyrrhiza_uralensis_Fisch0201940.1	-1.065449031	0.466652519	-2.283174283	0.022420104	0.365617826
Glycyrrhiza_uralensis_Fisch0206960.1	-1.065346076	1.777376459	-0.599392476	0.548911193	0.948764521
Glycyrrhiza_uralensis_Fisch0009520.1	-1.06508984	0.482109845	-2.20922649	0.027158891	0.404026274
Glycyrrhiza_uralensis_Fisch0107480.1	-1.064992975	0.665573964	-1.600112134	0.10957371	0.69750205
Glycyrrhiza_uralensis_Fisch0273600.1	-1.064886527	0.647757883	-1.64395765	0.100184951	0.681008687
Glycyrrhiza_uralensis_Fisch0118270.1	-1.064569135	0.654364336	-1.626875237	0.103763598	0.690599974
Glycyrrhiza_uralensis_Fisch0	-1.064435229	2.190381	-0.485958	0.626996	0.963502

081300.1		058	927	287	294
Glycyrrhiza_uralensis_Fisch0176380.1	-1.064302877	0.906479126	-1.174106327	0.24035244	0.836144417
Glycyrrhiza_uralensis_Fisch0172690.1	-1.064212479	1.325034814	-0.803158126	0.421883345	0.913755795
Glycyrrhiza_uralensis_Fisch0177400.1	-1.062921287	1.179608556	-0.901079668	0.367545962	0.900974301
Glycyrrhiza_uralensis_Fisch0269630.1	-1.062528453	1.55246129	-0.684415424	0.493712879	0.937222783
Glycyrrhiza_uralensis_Fisch0138180.1	-1.061909085	0.451354844	-2.352714496	0.018636937	0.334620502
Glycyrrhiza_uralensis_Fisch0222850.1	-1.061877601	0.692520893	-1.533350997	0.125189396	0.720011864
Glycyrrhiza_uralensis_Fisch0010200.1	-1.061862083	0.292042051	-3.635990362	0.000276915	0.027888437
Glycyrrhiza_uralensis_Fisch0251860.1	-1.061723692	0.400950909	-2.648014181	0.008096613	0.226611014
Glycyrrhiza_uralensis_Fisch0235710.1	-1.061382911	2.355872263	-0.450526511	0.652330843	0.965935919
Glycyrrhiza_uralensis_Fisch0071820.1	-1.060384084	0.707375004	-1.499040931	0.133863014	0.730409401
Glycyrrhiza_uralensis_Fisch0255390.1	-1.060109962	1.723372859	-0.61513674	0.538464421	0.947110698
Glycyrrhiza_uralensis_Fisch0248210.1	-1.059310402	0.502839564	-2.106656833	0.035147337	0.456090139
Glycyrrhiza_uralensis_Fisch0191630.1	-1.058804792	1.918677234	-0.551841015	0.581057284	0.953962814
Glycyrrhiza_uralensis_Fisch0283120.1	-1.058773995	2.732942843	-0.387411686	0.698451454	0.972014924
Glycyrrhiza_uralensis_Fisch0258880.1	-1.058215224	0.956256957	-1.106622248	0.268457284	0.855350341
Glycyrrhiza_uralensis_Fisch0165720.1	-1.057871807	1.131618254	-0.934830985	0.349875408	0.897065603
Glycyrrhiza_uralensis_Fisch0256120.1	-1.055709832	0.92682982	-1.139054667	0.254680354	0.847381568
Glycyrrhiza_uralensis_Fisch0205030.1	-1.055232692	0.649537665	-1.624590458	0.104249852	0.690798139
Glycyrrhiza_uralensis_Fisch0161390.1	-1.054806977	0.420502705	-2.508442783	0.012126461	0.272788696
Glycyrrhiza_uralensis_Fisch0264130.1	-1.054671626	0.54337869	-1.940951392	0.052264174	0.537606264
Glycyrrhiza_uralensis_Fisch0069920.1	-1.054650712	0.497384203	-2.120394466	0.033972793	0.451283183
Glycyrrhiza_uralensis_Fisch0	-1.053039349	1.251174	-0.841640	0.399989	0.910230

098570.1		566	629	14	427
Glycyrrhiza_uralensis_Fisch0133600.1	-1.052350433	0.404339071	-2.602643446	0.009250811	0.237026417
Glycyrrhiza_uralensis_Fisch0125000.1	-1.050712293	0.55724674	-1.885542287	0.059356661	0.566660084
Glycyrrhiza_uralensis_Fisch0172230.1	-1.049626372	0.790194512	-1.328313923	0.184074422	0.792181538
Glycyrrhiza_uralensis_Fisch0185000.1	-1.049252452	0.573058315	-1.830969771	0.067105055	0.592340664
Glycyrrhiza_uralensis_Fisch0266030.1	-1.048596318	0.455874856	-2.300184588	0.021437765	0.357777081
Glycyrrhiza_uralensis_Fisch0128080.1	-1.048457382	2.083264429	-0.503276189	0.614770105	0.962115213
Glycyrrhiza_uralensis_Fisch0053800.1	-1.04839068	3.81251093	-0.274986931	0.783326279	NA
Glycyrrhiza_uralensis_Fisch0263220.1	-1.048152077	0.630195075	-1.663218452	0.096268715	0.672428317
Glycyrrhiza_uralensis_Fisch0163370.1	-1.047600915	0.519170569	-2.017835711	0.043608374	0.503672539
Glycyrrhiza_uralensis_Fisch0213840.1	-1.047114295	0.460174876	-2.275470371	0.022877728	0.369126803
Glycyrrhiza_uralensis_Fisch0062890.1	-1.046721652	1.106351655	-0.946102125	0.3440965	0.894405752
Glycyrrhiza_uralensis_Fisch0262700.1	-1.046207201	1.095171105	-0.955291092	0.339430511	0.893353482
Glycyrrhiza_uralensis_Fisch0239530.1	-1.04296979	1.597544823	-0.65285792	0.513847879	0.941686425
Glycyrrhiza_uralensis_Fisch0240270.1	-1.042837941	0.520977889	-2.001693284	0.045317729	0.509005012
Glycyrrhiza_uralensis_Fisch0155420.1	-1.042667357	0.506090394	-2.060239374	0.039375662	0.481901786
Glycyrrhiza_uralensis_Fisch0048680.1	-1.041902542	0.633851532	-1.643764335	0.100224891	0.681071667
Glycyrrhiza_uralensis_Fisch0287040.1	-1.041316733	0.518956567	-2.006558542	0.044796689	0.508436505
Glycyrrhiza_uralensis_Fisch0177790.1	-1.040901044	0.243355602	-4.277284094	1.89E-05	0.003544366
Glycyrrhiza_uralensis_Fisch0051410.1	-1.040168685	0.423573212	-2.45569988	0.014061053	0.292018595
Glycyrrhiza_uralensis_Fisch0158300.1	-1.039939285	0.603773119	-1.722400771	0.084996944	0.643781241
Glycyrrhiza_uralensis_Fisch0143340.1	-1.039549155	0.64490027	-1.611953357	0.10697209	0.692097679
Glycyrrhiza_uralensis_Fisch0	-1.039471603	0.839276	-1.238533	0.215518	0.815264

050320.1		101	544	292	072
Glycyrrhiza_uralensis_Fisch0004810.1	-1.039120812	0.682807486	-1.521835705	0.128050253	0.72182017
Glycyrrhiza_uralensis_Fisch0195880.1	-1.038358251	1.044214783	-0.994391449	0.320032329	0.885830056
Glycyrrhiza_uralensis_Fisch0228480.1	-1.037393183	0.609729071	-1.70140023	0.088867858	0.652861515
Glycyrrhiza_uralensis_Fisch0285250.1	-1.037346567	1.470827086	-0.705281115	0.48063531	0.93190416
Glycyrrhiza_uralensis_Fisch0005660.1	-1.035882015	0.512736335	-2.020301554	0.043352118	0.502010217
Glycyrrhiza_uralensis_Fisch0187100.1	-1.035264831	2.111489874	-0.490300637	0.623921176	0.963420425
Glycyrrhiza_uralensis_Fisch0219880.1	-1.034721556	0.338090948	-3.060482872	0.002209804	0.110244597
Glycyrrhiza_uralensis_Fisch0216610.1	-1.034135298	4.035111173	-0.256284215	0.797731385	NA
Glycyrrhiza_uralensis_Fisch0008550.1	-1.033256449	0.509281367	-2.028851861	0.042473381	0.497970913
Glycyrrhiza_uralensis_Fisch0287030.1	-1.033128396	0.576649934	-1.791604116	0.073196403	0.610477279
Glycyrrhiza_uralensis_Fisch0159930.1	-1.032567224	0.442138756	-2.3353918	0.019522962	0.341686247
Glycyrrhiza_uralensis_Fisch0011700.1	-1.031585116	0.370101544	-2.787302921	0.005314877	0.182364357
Glycyrrhiza_uralensis_Fisch0239700.1	-1.031469262	1.980981767	-0.520685894	0.602585603	0.959570366
Glycyrrhiza_uralensis_Fisch0193270.1	-1.031205954	0.630121451	-1.636519359	0.101730938	0.684953846
Glycyrrhiza_uralensis_Fisch0148120.1	-1.030655992	2.980830246	-0.345761384	0.729522048	0.976482241
Glycyrrhiza_uralensis_Fisch0141480.1	-1.030197971	0.860721362	-1.196900666	0.231345274	0.828030507
Glycyrrhiza_uralensis_Fisch0229630.1	-1.0301554	2.741784787	-0.375724384	0.707121808	0.973746056
Glycyrrhiza_uralensis_Fisch0057770.1	-1.029265829	0.708619967	-1.452493404	0.146364462	0.745903116
Glycyrrhiza_uralensis_Fisch0017750.1	-1.028861425	0.864304611	-1.190392151	0.233892296	0.830362666
Glycyrrhiza_uralensis_Fisch0012700.1	-1.028732444	3.85775391	-0.266666166	0.789726207	NA
Glycyrrhiza_uralensis_Fisch0259810.1	-1.027981637	0.443877023	-2.315915408	0.020562888	0.349676938
Glycyrrhiza_uralensis_Fisch0	-1.026978928	3.824915	-0.268497	0.788316	NA

164340.1		142	18	646	
Glycyrrhiza_uralensis_Fisch0 004430.1	-1.026978928	3.824915 142	-0.268497 18	0.788316 646	NA
Glycyrrhiza_uralensis_Fisch0 016950.1	-1.026707163	1.481214 713	-0.693152 15	0.488214 073	0.935465 113
Glycyrrhiza_uralensis_Fisch0 140160.1	-1.02660439	0.540360 337	-1.899851 488	0.057452 612	0.559419 242
Glycyrrhiza_uralensis_Fisch0 104360.1	-1.025597874	0.390291 028	-2.627777 226	0.008594 478	0.231916 014
Glycyrrhiza_uralensis_Fisch0 165400.1	-1.025527449	1.497149 51	-0.684986 664	0.493352 336	0.937222 783
Glycyrrhiza_uralensis_Fisch0 268010.1	-1.024405081	0.707179 361	-1.448578 871	0.147455 225	0.747682 172
Glycyrrhiza_uralensis_Fisch0 265670.1	-1.024063263	0.404188 083	-2.533630 522	0.011288 769	0.264575 469
Glycyrrhiza_uralensis_Fisch0 169310.1	-1.022967064	0.355945 298	-2.873944 588	0.004053 801	0.155621 986
Glycyrrhiza_uralensis_Fisch0 059240.1	-1.022728629	0.405973 304	-2.519201 677	0.011762 126	0.268397 024
Glycyrrhiza_uralensis_Fisch0 009400.1	-1.021536889	0.420278 362	-2.430619 753	0.015073 024	0.304880 411
Glycyrrhiza_uralensis_Fisch0 064240.1	-1.021349391	0.529387 149	-1.929305 221	0.053692 982	0.546162 726
Glycyrrhiza_uralensis_Fisch0 076440.1	-1.021248422	1.036379 003	-0.985400 533	0.324427 368	0.887225 647
Glycyrrhiza_uralensis_Fisch0 024760.1	-1.01813874	0.326489 504	-3.118442 489	0.001818 096	0.098562 107
Glycyrrhiza_uralensis_Fisch0 239410.1	-1.018044449	0.651072 034	-1.563643 338	0.117901 351	0.707206 89
Glycyrrhiza_uralensis_Fisch0 282990.1	-1.017022097	0.377308 215	-2.695467 678	0.007028 99	0.209390 819
Glycyrrhiza_uralensis_Fisch0 031060.1	-1.0167318	1.009271 159	-1.007392 107	0.313746 383	0.882272 787
Glycyrrhiza_uralensis_Fisch0 130990.1	-1.016686234	0.627267 214	-1.620818 387	0.105056 594	0.692097 679
Glycyrrhiza_uralensis_Fisch0 144070.1	-1.016285467	3.428443 434	-0.296427 661	0.766903 504	NA
Glycyrrhiza_uralensis_Fisch0 194750.1	-1.016193802	3.183756 716	-0.319180 733	0.749589 466	0.977913 831
Glycyrrhiza_uralensis_Fisch0 004440.1	-1.016037897	0.522441 96	-1.944786 167	0.051800 72	0.535990 973
Glycyrrhiza_uralensis_Fisch0 011210.1	-1.015753223	0.733925 369	-1.384000 698	0.166358 241	0.772572 421
Glycyrrhiza_uralensis_Fisch0	-1.014713207	3.016805	-0.336353	0.736604	0.976483

263050.1		317	559	266	982
Glycyrrhiza_uralensis_Fisch0278280.1	-1.014530508	2.1611856	-0.469432384	0.638760605	0.965035372
Glycyrrhiza_uralensis_Fisch0127010.1	-1.014100258	0.380505946	-2.665136426	0.00769571	0.221839197
Glycyrrhiza_uralensis_Fisch0019990.1	-1.01321189	3.437296163	-0.294770029	0.768169563	NA
Glycyrrhiza_uralensis_Fisch0119270.1	-1.012927352	0.324242109	-3.12398459	0.001784198	0.097257005
Glycyrrhiza_uralensis_Fisch0032430.1	-1.012761785	0.87632619	-1.155690424	0.247807807	0.843032358
Glycyrrhiza_uralensis_Fisch0253810.1	-1.012755025	0.538015097	-1.882391462	0.059782884	0.568408325
Glycyrrhiza_uralensis_Fisch0099230.1	-1.01256836	0.326347781	-3.102727888	0.001917459	0.101435665
Glycyrrhiza_uralensis_Fisch0282370.1	-1.012273957	0.465950652	-2.172491769	0.02981859	0.425218763
Glycyrrhiza_uralensis_Fisch0130460.1	-1.012096932	0.48079628	-2.105043185	0.035287549	0.456503854
Glycyrrhiza_uralensis_Fisch0103130.1	-1.01208728	0.578613716	-1.74915881	0.080263572	0.634047973
Glycyrrhiza_uralensis_Fisch0031030.1	-1.011974839	0.689445672	-1.467809401	0.142156003	0.742901537
Glycyrrhiza_uralensis_Fisch0114320.1	-1.011608565	0.246094609	-4.110649033	3.95E-05	0.00637368
Glycyrrhiza_uralensis_Fisch0139670.1	-1.011224586	0.846901301	-1.194028848	0.232466684	0.828686349
Glycyrrhiza_uralensis_Fisch0141340.1	-1.010683728	0.311598209	-3.243547939	0.001180509	0.074384456
Glycyrrhiza_uralensis_Fisch0171050.1	-1.010199507	0.941949244	-1.072456413	0.283515082	0.86117142
Glycyrrhiza_uralensis_Fisch0214220.1	-1.009193156	1.553020291	-0.649826124	0.515804542	0.942226596
Glycyrrhiza_uralensis_Fisch0085720.1	-1.00854392	0.422424081	-2.387515211	0.016962701	0.319633242
Glycyrrhiza_uralensis_Fisch0163950.1	-1.0084112	3.923828779	-0.256996739	0.797181291	NA
Glycyrrhiza_uralensis_Fisch0109010.1	-1.008103811	0.706381963	-1.427136965	0.153540419	0.758333908
Glycyrrhiza_uralensis_Fisch0270740.1	-1.008084946	0.972107026	-1.037010246	0.299731082	0.871530218
Glycyrrhiza_uralensis_Fisch0068870.1	-1.006604439	3.83510362	-0.262471249	0.792958148	NA
Glycyrrhiza_uralensis_Fisch0	-1.006486536	0.558992	-1.800537	0.071775	0.605702

192330.1		389	102	871	951
Glycyrrhiza_uralensis_Fisch0217590.1	-1.006132611	1.032466905	-0.974493813	0.32981141	0.887225647
Glycyrrhiza_uralensis_Fisch0184770.1	-1.005290526	0.445120145	-2.25847007	0.023916368	0.377982265
Glycyrrhiza_uralensis_Fisch0156270.1	-1.005161882	0.411859143	-2.440547698	0.014665009	0.300454876
Glycyrrhiza_uralensis_Fisch0203090.1	-1.004913656	0.687707076	-1.461252459	0.143946171	0.744212491
Glycyrrhiza_uralensis_Fisch0073960.1	-1.004723331	0.487082327	-2.062738217	0.039137507	0.481530651
Glycyrrhiza_uralensis_Fisch0240220.1	-1.004544895	3.858516299	-0.260344862	0.79459777	NA
Glycyrrhiza_uralensis_Fisch0037370.1	-1.003346891	0.684670111	-1.465445729	0.142799349	0.743059364
Glycyrrhiza_uralensis_Fisch0026160.1	-1.003275582	0.520535134	-1.927392631	0.053930717	0.546343588
Glycyrrhiza_uralensis_Fisch0022290.1	-1.002722241	0.544968386	-1.839964056	0.065773515	0.587067905
Glycyrrhiza_uralensis_Fisch0264580.1	-1.002555688	0.379550354	-2.641429994	0.008255687	0.22867017
Glycyrrhiza_uralensis_Fisch0249290.1	-1.002427094	0.781182468	-1.283217603	0.199415851	0.806142846
Glycyrrhiza_uralensis_Fisch0119430.1	-1.001839006	0.497755863	-2.012711612	0.044144977	0.505740037
Glycyrrhiza_uralensis_Fisch0078360.1	-1.001038091	0.542248665	-1.846086779	0.064879606	0.584231994
Glycyrrhiza_uralensis_Fisch0184720.1	-1.000911453	1.948579711	-0.513662052	0.607488288	0.960732446
Glycyrrhiza_uralensis_Fisch0253900.1	-1.000895405	0.519712536	-1.925863505	0.054121419	0.547354019
Glycyrrhiza_uralensis_Fisch0118050.1	-1.000255514	4.038111169	-0.247703808	0.804363585	NA
Glycyrrhiza_uralensis_Fisch0087030.1	-1.000255514	4.038111169	-0.247703808	0.804363585	NA
Glycyrrhiza_uralensis_Fisch0252160.1	-1.00008649	0.623180652	-1.604809916	0.108535643	0.694431932
Glycyrrhiza_uralensis_Fisch0274980.1	23.16020503	3.907067395	5.927772084	NA	NA
Glycyrrhiza_uralensis_Fisch0010160.1	23.14501513	3.907235291	5.923629731	NA	NA
Glycyrrhiza_uralensis_Fisch0172050.1	23.13805655	3.907046159	5.922135449	NA	NA
Glycyrrhiza_uralensis_Fisch0	22.3558954	3.907598	5.7211339	NA	NA

187980.1		634	99		
Glycyrrhiza_uralensis_Fisch0034050.1	22.22898591	3.907678276	5.688540443	NA	NA
Glycyrrhiza_uralensis_Fisch0273880.1	21.36771031	3.200804747	6.675730635	NA	NA
Glycyrrhiza_uralensis_Fisch0110430.1	20.64969395	3.469489617	5.951795862	NA	NA
Glycyrrhiza_uralensis_Fisch0012540.1	20.61371455	3.483359558	5.917768237	NA	NA
Glycyrrhiza_uralensis_Fisch0052570.1	20.59389281	3.485923417	5.907729558	NA	NA
Glycyrrhiza_uralensis_Fisch0154470.1	20.58220691	3.497637881	5.884602011	NA	NA
Glycyrrhiza_uralensis_Fisch0118910.1	10.26920944	3.907122427	2.628330602	0.008580508	0.231916014
Glycyrrhiza_uralensis_Fisch0201400.1	9.845579434	3.907246366	2.519825604	0.011741299	0.268397024
Glycyrrhiza_uralensis_Fisch0281170.1	9.731665832	3.907286383	2.49064565	0.012751122	0.279271618
Glycyrrhiza_uralensis_Fisch0177150.1	9.621388698	3.907328252	2.462395805	0.013801227	0.288614706
Glycyrrhiza_uralensis_Fisch0287260.1	9.536422386	3.907362766	2.440628874	0.014661714	0.300454876
Glycyrrhiza_uralensis_Fisch0151580.1	9.41268003	3.907416809	2.408926534	0.015999518	0.313428369
Glycyrrhiza_uralensis_Fisch0120720.1	9.386499831	1.76011087	5.332902597	9.67E-08	5.41E-05
Glycyrrhiza_uralensis_Fisch0228930.1	9.120240695	1.523572866	5.986087634	2.15E-09	2.59E-06
Glycyrrhiza_uralensis_Fisch0051310.1	8.59409871	3.907918884	2.199149717	0.027867278	0.409522135
Glycyrrhiza_uralensis_Fisch0020660.1	8.587904859	1.669184264	5.144971135	2.68E-07	0.000121514
Glycyrrhiza_uralensis_Fisch0221260.1	8.586766643	3.907924792	2.197270188	0.028001155	0.41071774
Glycyrrhiza_uralensis_Fisch0278050.1	8.475677291	3.90801807	2.168791735	0.030098499	0.426204537
Glycyrrhiza_uralensis_Fisch0205590.1	8.472976526	1.781315309	4.756584352	1.97E-06	0.000629079
Glycyrrhiza_uralensis_Fisch0117550.1	8.46906645	3.908023851	2.16709692	0.030227465	0.426980679
Glycyrrhiza_uralensis_Fisch0010180.1	8.439155529	3.908050338	2.159428564	0.030816934	0.430007883
Glycyrrhiza_uralensis_Fisch0	8.436773227	1.668138	5.0575967	4.25E-07	0.000179

134970.1		771	49		289
Glycyrrhiza_uralensis_Fisch0057750.1	8.406211064	3.908080155	2.150982255	NA	NA
Glycyrrhiza_uralensis_Fisch0152870.1	8.358410606	2.330886827	3.585935837	0.000335872	0.031579797
Glycyrrhiza_uralensis_Fisch0244220.1	8.326011568	2.333562827	3.56793975	0.000359799	0.033181483
Glycyrrhiza_uralensis_Fisch0086870.1	8.262854536	1.654284024	4.994822182	5.89E-07	0.000244394
Glycyrrhiza_uralensis_Fisch0232410.1	8.209496098	2.052621567	3.999517608	NA	NA
Glycyrrhiza_uralensis_Fisch0187990.1	8.153168675	1.843591147	4.422438614	9.76E-06	0.002155106
Glycyrrhiza_uralensis_Fisch0283060.1	7.973897962	2.475778764	3.220763534	0.001278496	0.077710596
Glycyrrhiza_uralensis_Fisch0172060.1	7.963648572	2.477414921	3.214499317	0.001306722	0.078829085
Glycyrrhiza_uralensis_Fisch0243980.1	7.782294958	2.63150062	2.957360108	0.003102855	0.135300221
Glycyrrhiza_uralensis_Fisch0245840.1	7.752519248	1.901176443	4.077748427	4.55E-05	0.007030356
Glycyrrhiza_uralensis_Fisch0110730.1	7.735522156	1.607435628	4.81233713	1.49E-06	0.000535918
Glycyrrhiza_uralensis_Fisch0119050.1	7.646824299	2.449374026	3.121950432	0.001796572	0.097615089
Glycyrrhiza_uralensis_Fisch0113240.1	7.617957947	1.792890457	4.248981258	2.15E-05	0.00391584
Glycyrrhiza_uralensis_Fisch0010150.1	7.529981433	3.750593697	2.007677195	NA	NA
Glycyrrhiza_uralensis_Fisch0125620.1	7.504384781	2.586364049	2.901519136	0.003713581	0.148900684
Glycyrrhiza_uralensis_Fisch0027410.1	7.489774579	1.659538267	4.513167746	6.39E-06	0.00158481
Glycyrrhiza_uralensis_Fisch0273870.1	7.341040557	1.974478187	3.71796488	0.000200834	0.021873659
Glycyrrhiza_uralensis_Fisch0219420.1	7.230933723	2.734005338	2.644813316	0.0081736	0.22815254
Glycyrrhiza_uralensis_Fisch0117200.1	7.196813368	1.080316687	6.661762662	2.71E-11	5.01E-08
Glycyrrhiza_uralensis_Fisch0138910.1	7.187980452	2.680584568	2.681497364	0.007329349	0.214881163
Glycyrrhiza_uralensis_Fisch0098150.1	7.180426659	1.737218026	4.133290441	3.58E-05	0.006015961
Glycyrrhiza_uralensis_Fisch0	7.135243468	3.535290	2.0182907	NA	NA

138810.1		166	58		
Glycyrrhiza_uralensis_Fisch0090790.1	7.102534748	2.000804579	3.54983931	0.000385466	0.034487819
Glycyrrhiza_uralensis_Fisch0088150.1	7.088652084	2.01193096	3.52330782	0.000426196	0.03734985
Glycyrrhiza_uralensis_Fisch0136360.1	7.016577598	1.996759633	3.513982095	0.000441443	0.037948295
Glycyrrhiza_uralensis_Fisch0281480.1	6.936219622	2.648520422	2.618903582	0.008821288	0.233353815
Glycyrrhiza_uralensis_Fisch0113580.1	6.93386687	2.677035092	2.590129241	0.009593991	0.240800159
Glycyrrhiza_uralensis_Fisch0052980.1	6.853782032	3.668510174	1.868273961	0.061723894	0.574070376
Glycyrrhiza_uralensis_Fisch0112420.1	6.831410193	3.679610555	1.85655794	0.063374073	0.578685101
Glycyrrhiza_uralensis_Fisch0023700.1	6.827294884	2.704202777	2.52469783	0.011579781	0.267234253
Glycyrrhiza_uralensis_Fisch0167540.1	6.785598224	3.702672268	1.832621883	0.066858822	0.591869009
Glycyrrhiza_uralensis_Fisch0051550.1	6.762562828	2.724963755	2.481707442	0.013075458	0.282914881
Glycyrrhiza_uralensis_Fisch0281290.1	6.626561956	2.865237301	2.312744551	0.020736688	0.351007089
Glycyrrhiza_uralensis_Fisch0038450.1	6.59488764	2.895475976	2.277652342	0.022747299	0.368209478
Glycyrrhiza_uralensis_Fisch0019590.1	6.5898382	2.871253592	2.295108387	0.021726914	0.361164923
Glycyrrhiza_uralensis_Fisch0232320.1	6.589208883	1.872680827	3.518596863	0.000433835	0.037698261
Glycyrrhiza_uralensis_Fisch0115190.1	6.581923966	1.955688841	3.365527189	0.000763975	0.056235129
Glycyrrhiza_uralensis_Fisch0194490.1	6.510574753	1.653321432	3.93787598	8.22E-05	0.010992777
Glycyrrhiza_uralensis_Fisch0275650.1	6.503774448	3.854791347	1.687192344	0.091566373	0.659091686
Glycyrrhiza_uralensis_Fisch0118520.1	6.48450855	3.859958581	1.679942521	0.0929685	0.662056741
Glycyrrhiza_uralensis_Fisch0135830.1	6.437339358	3.04094637	2.116886842	0.034269453	0.451733694
Glycyrrhiza_uralensis_Fisch0006550.1	6.430438656	2.874744895	2.236872798	0.025294655	0.388938567
Glycyrrhiza_uralensis_Fisch0271400.1	6.411755976	2.853192102	2.24722197	0.024625845	0.384150411
Glycyrrhiza_uralensis_Fisch0	6.402048613	3.908165	1.6381212	0.101396	0.683644

029870.1		195	9	395	598
Glycyrrhiza_uralensis_Fisch0159050.1	6.387621235	2.875353398	2.221508229	0.026316558	0.396768725
Glycyrrhiza_uralensis_Fisch0034990.1	6.362501131	3.058274259	2.080422026	0.037486841	0.473155876
Glycyrrhiza_uralensis_Fisch0182050.1	6.348623713	3.119647091	2.03504548	0.041846297	0.49475144
Glycyrrhiza_uralensis_Fisch0076630.1	6.330567038	2.00668119	3.154744794	0.001606386	0.091624881
Glycyrrhiza_uralensis_Fisch0266680.1	6.330038766	2.9008578	2.18212653	0.029100196	0.42018099
Glycyrrhiza_uralensis_Fisch0285790.1	6.312062143	1.660553624	3.80117935	0.000144009	0.017245259
Glycyrrhiza_uralensis_Fisch0237590.1	6.301957535	3.912435608	1.61075048	0.107234118	0.692127618
Glycyrrhiza_uralensis_Fisch0178730.1	6.282580586	3.136837116	2.002839278	0.045194542	0.509005012
Glycyrrhiza_uralensis_Fisch0131540.1	6.228927022	2.290009922	2.720043683	0.006527329	0.201426687
Glycyrrhiza_uralensis_Fisch0095150.1	6.223584062	3.912752257	1.590589859	0.111701908	0.698786349
Glycyrrhiza_uralensis_Fisch0199310.1	6.172539725	2.130916115	2.896660118	0.003771581	0.149805215
Glycyrrhiza_uralensis_Fisch0172180.1	6.160303962	2.195677768	2.805650288	0.005021516	0.176538318
Glycyrrhiza_uralensis_Fisch0111600.1	6.156805658	3.010514598	2.045100748	0.040844927	0.491323029
Glycyrrhiza_uralensis_Fisch0149850.1	6.151584205	2.345614791	2.62258928	0.00872644	0.232899664
Glycyrrhiza_uralensis_Fisch0092400.1	6.149494335	0.437793808	14.04655392	8.09E-45	1.95E-40
Glycyrrhiza_uralensis_Fisch0038320.1	6.137320429	1.612049144	3.80715467	0.000140575	0.017003197
Glycyrrhiza_uralensis_Fisch0057830.1	6.059800264	3.026490308	2.002253319	0.045257494	0.509005012
Glycyrrhiza_uralensis_Fisch0051320.1	6.040647853	3.913561753	1.543516682	0.122705463	0.714644751
Glycyrrhiza_uralensis_Fisch0165120.1	5.99919749	3.052150104	1.965564368	0.049348953	0.528259591
Glycyrrhiza_uralensis_Fisch0273520.1	5.926182354	2.152983914	2.752543721	0.005913425	0.192699972
Glycyrrhiza_uralensis_Fisch0010140.1	5.913132161	2.121219538	2.7876097	NA	NA
Glycyrrhiza_uralensis_Fisch0	5.865804067	0.561250	10.451310	1.45E-25	1.74E-21

268930.1		578	52		
Glycyrrhiza_uralensis_Fisch0004070.1	5.833253195	3.16622359	1.842337735	0.065425763	0.585862398
Glycyrrhiza_uralensis_Fisch0009970.1	5.833150668	3.147641598	1.853181338	0.063856372	0.580665986
Glycyrrhiza_uralensis_Fisch0040220.1	5.813628819	3.26295588	1.78170623	0.07479715	0.618340453
Glycyrrhiza_uralensis_Fisch0025350.1	5.785794478	3.172588454	1.823682637	0.068200065	0.596069561
Glycyrrhiza_uralensis_Fisch0238420.1	5.774093504	3.914940738	1.474886567	0.140243042	0.740111824
Glycyrrhiza_uralensis_Fisch0171100.1	5.753099046	3.207487278	1.793646723	0.072869574	0.608383852
Glycyrrhiza_uralensis_Fisch0012550.1	5.723328896	3.793834605	1.508586824	NA	NA
Glycyrrhiza_uralensis_Fisch0153170.1	5.701088256	3.222978468	1.768888099	0.076912551	0.623502421
Glycyrrhiza_uralensis_Fisch0243740.1	5.701088256	3.222978468	1.768888099	0.076912551	0.623502421
Glycyrrhiza_uralensis_Fisch0132170.1	5.689006938	3.915437218	1.452968499	0.1462325	0.745903116
Glycyrrhiza_uralensis_Fisch0021450.1	5.683656445	1.670274905	3.40282694	0.000666925	0.051180584
Glycyrrhiza_uralensis_Fisch0163900.1	5.683205731	2.488427619	2.283854145	0.022380104	0.365460727
Glycyrrhiza_uralensis_Fisch0052110.1	5.666908457	2.284309692	2.480796924	0.013108903	0.282914881
Glycyrrhiza_uralensis_Fisch0149680.1	5.634524306	2.363326633	2.384149625	0.017118641	0.320432823
Glycyrrhiza_uralensis_Fisch0023020.1	5.616503809	3.262035878	1.721778674	0.085109619	0.643781241
Glycyrrhiza_uralensis_Fisch0271390.1	5.607467075	3.280113064	1.709534691	0.087351951	0.648738497
Glycyrrhiza_uralensis_Fisch0059050.1	5.586476463	2.534074117	2.204543436	0.027486149	0.406882903
Glycyrrhiza_uralensis_Fisch0175180.1	5.585855839	3.916079629	1.426389749	0.153755871	0.758333908
Glycyrrhiza_uralensis_Fisch0108730.1	5.585855839	3.916079629	1.426389749	0.153755871	0.758333908
Glycyrrhiza_uralensis_Fisch0194920.1	5.577806318	3.318182825	1.680982216	0.092766369	0.661792087
Glycyrrhiza_uralensis_Fisch0156050.1	5.570615326	3.285545529	1.695491746	0.089982175	0.655331602
Glycyrrhiza_uralensis_Fisch0	5.569373601	2.005146	2.7775399	0.005477	0.184644

171640.1		199	14	211	923
Glycyrrhiza_uralensis_Fisch0213020.1	5.564059459	2.059449476	2.701721758	0.006898146	0.20798179
Glycyrrhiza_uralensis_Fisch0145270.1	5.550610528	1.049137193	5.290643171	1.22E-07	6.11E-05
Glycyrrhiza_uralensis_Fisch0056160.1	5.550176518	3.41548865	1.625002185	0.104162093	0.690798139
Glycyrrhiza_uralensis_Fisch0122660.1	5.54734623	1.482414546	3.742101861	0.000182488	0.020525581
Glycyrrhiza_uralensis_Fisch0009980.1	5.537091733	3.916399651	1.413821935	0.157414191	0.761519387
Glycyrrhiza_uralensis_Fisch0106010.1	5.535581136	2.384650206	2.321338837	0.020268564	0.347651918
Glycyrrhiza_uralensis_Fisch0076130.1	5.5348175	2.403418147	2.302894112	0.021284802	0.357517972
Glycyrrhiza_uralensis_Fisch0060760.1	5.523763795	3.320688528	1.663439298	0.096224532	0.672404034
Glycyrrhiza_uralensis_Fisch0117750.1	5.505829177	3.355314514	1.640927893	0.100812384	0.681807835
Glycyrrhiza_uralensis_Fisch0249540.1	5.501257147	3.410798753	1.612894089	0.10676752	0.692097679
Glycyrrhiza_uralensis_Fisch0122230.1	5.495567096	1.823841721	3.013182028	0.002585238	0.120053174
Glycyrrhiza_uralensis_Fisch0159790.1	5.474757303	3.91682476	1.397753956	0.162186965	0.767574428
Glycyrrhiza_uralensis_Fisch0007290.1	5.437627353	1.549297141	3.509738197	NA	NA
Glycyrrhiza_uralensis_Fisch0276540.1	5.435857568	3.404346253	1.596740509	0.110323562	0.697894385
Glycyrrhiza_uralensis_Fisch0053030.1	5.41582612	3.917243861	1.38256037	0.166799708	0.773428812
Glycyrrhiza_uralensis_Fisch0193600.1	5.407671441	3.390836641	1.594789727	0.110759265	0.698264932
Glycyrrhiza_uralensis_Fisch0027430.1	5.357097907	3.420349057	1.566243041	0.11729173	0.706303999
Glycyrrhiza_uralensis_Fisch0036220.1	5.354384368	3.917699382	1.366716495	0.171714186	0.779233384
Glycyrrhiza_uralensis_Fisch0224500.1	5.352071716	3.417846828	1.565919126	0.117367552	0.706303999
Glycyrrhiza_uralensis_Fisch0219350.1	5.332794059	1.570388223	3.395844404	0.000684172	0.051759221
Glycyrrhiza_uralensis_Fisch0279050.1	5.304461073	3.48687563	1.521264776	0.128193406	0.72182017
Glycyrrhiza_uralensis_Fisch0	5.300994116	3.467340	1.5288357	0.126305	0.720930

074150.1		431	81	16	802
Glycyrrhiza_uralensis_Fisch0 228170.1	5.299922884	3.468725 516	1.5279164 81	0.126533 276	0.721053 59
Glycyrrhiza_uralensis_Fisch0 092550.1	5.25335142	3.918491 79	1.3406564 83	0.180032 01	0.789067 991
Glycyrrhiza_uralensis_Fisch0 016860.1	5.223046339	3.918740 464	1.3328380 35	0.182584 961	0.791863 958
Glycyrrhiza_uralensis_Fisch0 189660.1	5.196229972	2.671042 843	1.9453937 2	0.051727 61	0.535990 973
Glycyrrhiza_uralensis_Fisch0 059040.1	5.194749493	3.563004 246	1.4579689 32	0.144849 107	0.744212 491
Glycyrrhiza_uralensis_Fisch0 247360.1	5.181771076	3.599067 304	1.4397538 69	0.149937 047	0.753284 223
Glycyrrhiza_uralensis_Fisch0 220820.1	5.17800076	3.565374 322	1.4523021 4	0.146417 613	0.745903 116
Glycyrrhiza_uralensis_Fisch0 106020.1	5.170419465	3.541632 685	1.4598971 5	0.144318 343	0.744212 491
Glycyrrhiza_uralensis_Fisch0 089670.1	5.164697789	3.547911 864	1.4557007 02	0.145475 376	0.744673 28
Glycyrrhiza_uralensis_Fisch0 218200.1	5.155076643	3.613599 637	1.4265765 89	0.153701 976	0.758333 908
Glycyrrhiza_uralensis_Fisch0 007150.1	5.148681023	2.599922 994	1.9803205 84	0.047667 517	0.521890 596
Glycyrrhiza_uralensis_Fisch0 156090.1	5.147176502	3.919386 352	1.3132608 12	0.189095 079	0.797474 632
Glycyrrhiza_uralensis_Fisch0 176110.1	5.141905647	3.626452 515	1.4178885 91	0.156223 294	0.760213 791
Glycyrrhiza_uralensis_Fisch0 092520.1	5.123676051	2.642316 432	1.9390849 59	0.052490 994	0.538262 101
Glycyrrhiza_uralensis_Fisch0 110310.1	5.099478794	2.654073 334	1.9213782 56	0.054684 038	0.549121 731
Glycyrrhiza_uralensis_Fisch0 010170.1	5.078542826	3.920000 531	1.2955464 64	0.195131 784	0.802189 929
Glycyrrhiza_uralensis_Fisch0 217220.1	5.072493081	3.671339 632	1.3816463 72	0.167080 308	0.773849 318
Glycyrrhiza_uralensis_Fisch0 002020.1	5.057360674	2.107675 218	2.3994971 47	0.016417 608	0.315380 541
Glycyrrhiza_uralensis_Fisch0 004850.1	5.056435848	2.636338 429	1.9179767 64	0.055113 958	NA
Glycyrrhiza_uralensis_Fisch0 069140.1	5.051761024	3.920248 202	1.2886329 55	0.197525 721	NA
Glycyrrhiza_uralensis_Fisch0 129720.1	5.047337568	3.659628 363	1.3791940 24	0.167834 94	NA
Glycyrrhiza_uralensis_Fisch0	5.045070415	1.348680	3.7407467	NA	NA

143130.1		025	45		
Glycyrrhiza_uralensis_Fisch0049620.1	5.034845687	3.666880836	1.373059532	0.16973384	NA
Glycyrrhiza_uralensis_Fisch0123490.1	5.029061026	2.853816282	1.762223118	0.078031608	NA
Glycyrrhiza_uralensis_Fisch0230470.1	5.026835832	3.64765134	1.378102062	0.168171779	NA
Glycyrrhiza_uralensis_Fisch0183450.1	5.018377421	3.70268949	1.355333045	0.175311471	NA
Glycyrrhiza_uralensis_Fisch0062730.1	5.006888524	3.732108391	1.341571037	0.179735122	0.789067991
Glycyrrhiza_uralensis_Fisch0149360.1	5.000474408	3.920735469	1.275391938	0.202170551	NA
Glycyrrhiza_uralensis_Fisch0073190.1	4.998139214	1.771640653	2.821192438	0.004784549	0.171375148
Glycyrrhiza_uralensis_Fisch0160840.1	4.978583344	3.738996739	1.331529202	0.183014943	NA
Glycyrrhiza_uralensis_Fisch0017130.1	4.974769241	3.717386907	1.338243601	0.18081704	NA
Glycyrrhiza_uralensis_Fisch0024370.1	4.970334974	1.932061399	2.572555394	0.01009508	0.248454575
Glycyrrhiza_uralensis_Fisch0155350.1	4.917938268	3.921556787	1.254078045	0.20981364	NA
Glycyrrhiza_uralensis_Fisch0257650.1	4.899794513	3.921743698	1.249391824	0.211521797	NA
Glycyrrhiza_uralensis_Fisch0208520.1	4.894805777	3.79735916	1.289002586	0.197397188	NA
Glycyrrhiza_uralensis_Fisch0009000.1	4.88898318	3.783592124	1.292153863	0.196303866	NA
Glycyrrhiza_uralensis_Fisch0273930.1	4.886861592	1.709598463	2.858485017	0.004256692	0.159994804
Glycyrrhiza_uralensis_Fisch0279330.1	4.865494816	3.775970356	1.288541582	0.197557504	NA
Glycyrrhiza_uralensis_Fisch0188060.1	4.829546293	3.907060219	1.236107463	0.216418621	NA
Glycyrrhiza_uralensis_Fisch0152300.1	4.82745692	2.770033956	1.742742868	0.08137854	NA
Glycyrrhiza_uralensis_Fisch0105140.1	4.826321189	1.509837213	3.196583809	0.001390654	0.083059669
Glycyrrhiza_uralensis_Fisch0127460.1	4.802742701	3.826942902	1.254981541	0.209485461	NA
Glycyrrhiza_uralensis_Fisch0176750.1	4.796035981	3.867881206	1.239964654	0.214988468	NA
Glycyrrhiza_uralensis_Fisch0	4.787950252	3.848251	1.2441885	0.213430	NA

237920.1		342	49	177	
Glycyrrhiza_uralensis_Fisch0231660.1	4.768377884	3.858981252	1.235657178	0.216586021	NA
Glycyrrhiza_uralensis_Fisch0038100.1	4.764359035	2.819808522	1.689603743	0.091103789	NA
Glycyrrhiza_uralensis_Fisch0242520.1	4.760916245	3.856082117	1.234651156	0.216960361	NA
Glycyrrhiza_uralensis_Fisch0215040.1	4.760916245	3.856082117	1.234651156	0.216960361	NA
Glycyrrhiza_uralensis_Fisch0094270.1	4.752985136	1.300467869	3.654827043	0.000257355	0.026248071
Glycyrrhiza_uralensis_Fisch0269830.1	4.737189231	3.923527575	1.207380129	0.227285784	NA
Glycyrrhiza_uralensis_Fisch0195800.1	4.72494284	3.91518986	1.206823426	0.227500152	NA
Glycyrrhiza_uralensis_Fisch0207370.1	4.719565426	3.918877155	1.204315736	0.228467567	NA
Glycyrrhiza_uralensis_Fisch0286440.1	4.716972377	3.904090534	1.208212857	0.226965398	NA
Glycyrrhiza_uralensis_Fisch0157890.1	4.71176782	3.891175395	1.210885489	0.225939295	NA
Glycyrrhiza_uralensis_Fisch0052670.1	4.703912853	3.902452385	1.205373542	0.228059131	NA
Glycyrrhiza_uralensis_Fisch0275600.1	4.688073817	3.924106969	1.194685531	0.232209918	NA
Glycyrrhiza_uralensis_Fisch0152860.1	4.675564155	1.500994022	3.114978532	0.001839583	0.099279736
Glycyrrhiza_uralensis_Fisch0276150.1	4.642791279	2.925044944	1.58725468	0.112454975	NA
Glycyrrhiza_uralensis_Fisch0176740.1	4.637546269	3.92472387	1.18162358	0.237355093	NA
Glycyrrhiza_uralensis_Fisch0123200.1	4.636832274	3.924732743	1.181438986	0.237428378	NA
Glycyrrhiza_uralensis_Fisch0193450.1	4.605808491	2.586217106	1.780905586	0.074927876	0.618589034
Glycyrrhiza_uralensis_Fisch0100770.1	4.596363229	1.070981967	4.291727939	1.77E-05	0.003360098
Glycyrrhiza_uralensis_Fisch0263450.1	4.59540952	3.92525503	1.170728904	0.241707763	NA
Glycyrrhiza_uralensis_Fisch0029120.1	4.583592546	3.01290449	1.521320228	0.128179496	NA
Glycyrrhiza_uralensis_Fisch0089460.1	4.583056303	2.233206376	2.052231425	0.040147177	0.486806106
Glycyrrhiza_uralensis_Fisch0	4.569394317	2.947919	1.5500403	0.121131	NA

177080.1		648	21	839	
Glycyrrhiza_uralensis_Fisch0 272210.1	4.554323002	2.316380 639	1.9661375 71	0.049282 721	0.528259 591
Glycyrrhiza_uralensis_Fisch0 133550.1	4.553729735	3.010005 881	1.5128640 66	0.130314 191	NA
Glycyrrhiza_uralensis_Fisch0 100080.1	4.542187884	2.970048 721	1.5293311 02	0.126182 383	NA
Glycyrrhiza_uralensis_Fisch0 051050.1	4.537537905	3.926010 164	1.1557631 58	0.247778 046	NA
Glycyrrhiza_uralensis_Fisch0 084130.1	4.537327127	3.926012 97	1.1557086 44	0.247800 351	NA
Glycyrrhiza_uralensis_Fisch0 114900.1	4.519584945	3.926250 614	1.1511198 31	0.249682 94	NA
Glycyrrhiza_uralensis_Fisch0 248670.1	4.516713989	3.926289 343	1.1503772 63	0.249988 521	NA
Glycyrrhiza_uralensis_Fisch0 276310.1	4.504638533	3.926453 08	1.1472538 81	0.251276 709	NA
Glycyrrhiza_uralensis_Fisch0 209080.1	4.479465454	1.380267 185	3.2453611 18	0.001173 018	0.074301 409
Glycyrrhiza_uralensis_Fisch0 211270.1	4.477453519	3.926826 724	1.1402218 21	0.254193 899	NA
Glycyrrhiza_uralensis_Fisch0 167530.1	4.472121066	2.688192 853	1.6636161 58	0.096189 161	0.672404 034
Glycyrrhiza_uralensis_Fisch0 219330.1	4.458828362	3.160686 251	1.4107152 71	0.158328 586	NA
Glycyrrhiza_uralensis_Fisch0 086530.1	4.444878921	3.927283 769	1.1317946 91	0.257720 76	NA
Glycyrrhiza_uralensis_Fisch0 235990.1	4.444878921	3.927283 769	1.1317946 91	0.257720 76	NA
Glycyrrhiza_uralensis_Fisch0 191750.1	4.443383234	3.043450 905	1.4599819	0.144295 049	NA
Glycyrrhiza_uralensis_Fisch0 034020.1	4.433825608	3.927441 202	1.1289349 43	0.258925 279	NA
Glycyrrhiza_uralensis_Fisch0 112580.1	4.430446991	3.927489 564	1.1280607 93	0.259294 246	NA
Glycyrrhiza_uralensis_Fisch0 240770.1	4.420934654	3.927626 331	1.1255996 07	0.260335 035	NA
Glycyrrhiza_uralensis_Fisch0 111860.1	4.414886673	3.927713 756	1.1240347 31	0.260998 292	NA
Glycyrrhiza_uralensis_Fisch0 113860.1	4.414886673	3.927713 756	1.1240347 31	0.260998 292	NA
Glycyrrhiza_uralensis_Fisch0 135180.1	4.414886673	3.927713 756	1.1240347 31	0.260998 292	NA
Glycyrrhiza_uralensis_Fisch0	4.414886673	3.927713	1.1240347	0.260998	NA

218160.1		756	31	292	
Glycyrrhiza_uralensis_Fisch0 131190.1	4.414886673	3.927713 756	1.1240347 31	0.260998 292	NA
Glycyrrhiza_uralensis_Fisch0 154740.1	4.388787754	3.928095 228	1.1172814 05	0.263874 024	NA
Glycyrrhiza_uralensis_Fisch0 157150.1	4.388787754	3.928095 228	1.1172814 05	0.263874 024	NA
Glycyrrhiza_uralensis_Fisch0 163530.1	4.382320399	3.928190 824	1.1156078 19	0.264590 044	NA
Glycyrrhiza_uralensis_Fisch0 042460.1	4.369186224	3.928386 279	1.1122089 1	0.266048 338	NA
Glycyrrhiza_uralensis_Fisch0 106440.1	4.369186224	3.928386 279	1.1122089 1	0.266048 338	NA
Glycyrrhiza_uralensis_Fisch0 028080.1	4.368170631	0.516492 51	8.4573746	2.73E-17	1.32E-13
Glycyrrhiza_uralensis_Fisch0 037200.1	4.357541958	3.928561 049	1.1091954 29	0.267345 884	NA
Glycyrrhiza_uralensis_Fisch0 052520.1	4.349038256	3.928689 571	1.1069946 29	0.268296 25	NA
Glycyrrhiza_uralensis_Fisch0 037230.1	4.340602482	3.928817 813	1.1048113 42	0.269241 345	NA
Glycyrrhiza_uralensis_Fisch0 267150.1	4.322063022	3.929102 286	1.1000128 55	0.271326 521	NA
Glycyrrhiza_uralensis_Fisch0 128440.1	4.322063022	3.929102 286	1.1000128 55	0.271326 521	NA
Glycyrrhiza_uralensis_Fisch0 223350.1	4.322063022	3.929102 286	1.1000128 55	0.271326 521	NA
Glycyrrhiza_uralensis_Fisch0 029020.1	4.315010378	3.929211 461	1.0981873 64	0.272122 683	NA
Glycyrrhiza_uralensis_Fisch0 153040.1	4.315010378	3.929211 461	1.0981873 64	0.272122 683	NA
Glycyrrhiza_uralensis_Fisch0 154450.1	4.311840155	1.414734 615	3.0478084 8	0.002305 168	0.112752 905
Glycyrrhiza_uralensis_Fisch0 118490.1	4.304721866	3.929371 682	1.0955242 25	0.273287 038	NA
Glycyrrhiza_uralensis_Fisch0 231400.1	4.304721866	3.929371 682	1.0955242 25	0.273287 038	NA
Glycyrrhiza_uralensis_Fisch0 201790.1	4.301276913	3.929425 583	1.0946324 91	0.273677 675	NA
Glycyrrhiza_uralensis_Fisch0 273710.1	4.28918669	3.929615 771	1.0915028 19	0.275051 692	NA
Glycyrrhiza_uralensis_Fisch0 185070.1	4.28918669	3.929615 771	1.0915028 19	0.275051 692	NA
Glycyrrhiza_uralensis_Fisch0	4.28918669	3.929615	1.0915028	0.275051	NA

111060.1		771	19	692	
Glycyrrhiza_uralensis_Fisch0201690.1	4.287417952	3.929643727	1.091044952	0.275253103	NA
Glycyrrhiza_uralensis_Fisch0029440.1	4.273431351	3.929866002	1.087424189	0.276849386	NA
Glycyrrhiza_uralensis_Fisch0234640.1	4.273431351	3.929866002	1.087424189	0.276849386	NA
Glycyrrhiza_uralensis_Fisch0216750.1	4.259020883	0.823547714	5.171553279	2.32E-07	0.000109569
Glycyrrhiza_uralensis_Fisch0245350.1	4.25631317	2.473566132	1.720719376	0.085301759	0.643781241
Glycyrrhiza_uralensis_Fisch0106030.1	4.253696625	3.930183292	1.082315075	0.27911255	NA
Glycyrrhiza_uralensis_Fisch0024390.1	4.2510838	1.188662457	3.576359106	0.000348413	0.032379537
Glycyrrhiza_uralensis_Fisch0108440.1	4.233534768	3.24184344	1.305903523	0.191585369	NA
Glycyrrhiza_uralensis_Fisch0206550.1	4.232202886	1.874258829	2.258067467	0.023941454	0.378130438
Glycyrrhiza_uralensis_Fisch0031370.1	4.230009367	3.31582239	1.275704447	0.202060016	NA
Glycyrrhiza_uralensis_Fisch0111200.1	4.226646163	3.930625272	1.0753114	0.282235334	NA
Glycyrrhiza_uralensis_Fisch0077420.1	4.217359376	3.930778919	1.072906786	0.283312941	NA
Glycyrrhiza_uralensis_Fisch0140550.1	4.206411738	3.217854739	1.307209952	0.191141417	NA
Glycyrrhiza_uralensis_Fisch0034520.1	4.199140632	3.221409574	1.303510322	0.192400595	NA
Glycyrrhiza_uralensis_Fisch0118650.1	4.189527001	3.931245327	1.065699709	0.286559409	NA
Glycyrrhiza_uralensis_Fisch0232160.1	4.187849864	3.931273719	1.065265398	0.286755846	NA
Glycyrrhiza_uralensis_Fisch0123960.1	4.186083322	1.557011663	2.688536908	0.00717659	0.211432712
Glycyrrhiza_uralensis_Fisch0121540.1	4.176729425	2.612384832	1.598818587	0.109860918	0.697621363
Glycyrrhiza_uralensis_Fisch0176400.1	4.167039203	3.931628758	1.059876061	0.289200988	NA
Glycyrrhiza_uralensis_Fisch0273890.1	4.151480296	3.931897544	1.055846509	0.291038346	NA
Glycyrrhiza_uralensis_Fisch0117560.1	4.151480296	3.931897544	1.055846509	0.291038346	NA
Glycyrrhiza_uralensis_Fisch0	4.148042256	3.931957	1.0549560	0.291445	NA

190360.1		327	72	416	
Glycyrrhiza_uralensis_Fisch0 053760.1	4.148042256	3.931957 327	1.0549560 72	0.291445 416	NA
Glycyrrhiza_uralensis_Fisch0 280600.1	4.132110717	3.932236 211	1.0508297 3	0.293336 799	NA
Glycyrrhiza_uralensis_Fisch0 162660.1	4.126941537	1.393485 15	2.9615970 69	0.003060 48	0.135232 055
Glycyrrhiza_uralensis_Fisch0 244070.1	4.112490948	3.932583 889	1.0457478	0.295677 488	NA
Glycyrrhiza_uralensis_Fisch0 002870.1	4.112490948	3.932583 889	1.0457478	0.295677 488	NA
Glycyrrhiza_uralensis_Fisch0 049960.1	4.096714396	3.932866 888	1.0416610 87	0.297568 836	NA
Glycyrrhiza_uralensis_Fisch0 046360.1	4.092617158	3.932940 889	1.0405997 12	0.298061 365	NA
Glycyrrhiza_uralensis_Fisch0 022520.1	4.07656388	3.332885 24	1.2231335 87	0.221279 244	NA
Glycyrrhiza_uralensis_Fisch0 152610.1	4.072482414	3.933307 592	1.0353836 61	0.300489 773	NA
Glycyrrhiza_uralensis_Fisch0 149890.1	4.061344719	1.691685 162	2.4007686 6	0.016360 676	0.315198 656
Glycyrrhiza_uralensis_Fisch0 105880.1	4.057111069	0.662155 833	6.1271242 56	8.95E-10	1.13E-06
Glycyrrhiza_uralensis_Fisch0 253760.1	4.055948195	0.848849 067	4.7781735 89	1.77E-06	0.000608 265
Glycyrrhiza_uralensis_Fisch0 226560.1	4.052080157	3.933684 389	1.0300979 33	0.302964 036	NA
Glycyrrhiza_uralensis_Fisch0 231730.1	4.052080157	3.933684 389	1.0300979 33	0.302964 036	NA
Glycyrrhiza_uralensis_Fisch0 247350.1	4.052080157	3.933684 389	1.0300979 33	0.302964 036	NA
Glycyrrhiza_uralensis_Fisch0 158420.1	4.052080157	3.933684 389	1.0300979 33	0.302964 036	NA
Glycyrrhiza_uralensis_Fisch0 131580.1	4.052080157	3.933684 389	1.0300979 33	0.302964 036	NA
Glycyrrhiza_uralensis_Fisch0 121600.1	4.052080157	3.933684 389	1.0300979 33	0.302964 036	NA
Glycyrrhiza_uralensis_Fisch0 179830.1	4.052080157	3.933684 389	1.0300979 33	0.302964 036	NA
Glycyrrhiza_uralensis_Fisch0 258810.1	4.035665199	3.933991 412	1.0258449 44	0.304964 678	NA
Glycyrrhiza_uralensis_Fisch0 284150.1	4.010708149	3.934464 902	1.0193783 01	0.308023 403	NA
Glycyrrhiza_uralensis_Fisch0	4.00187775	1.611348	2.4835585	0.013007	0.282083

284760.1		284	15	696	407
Glycyrrhiza_uralensis_Fisch0124660.1	3.999230828	3.934685397	1.016404216	0.309436939	NA
Glycyrrhiza_uralensis_Fisch0132160.1	3.999230828	3.934685397	1.016404216	0.309436939	NA
Glycyrrhiza_uralensis_Fisch0070050.1	3.997809157	1.916167899	2.086356399	0.036946351	0.469219488
Glycyrrhiza_uralensis_Fisch0024380.1	3.979449694	0.998888362	3.983878323	6.78E-05	0.009599625
Glycyrrhiza_uralensis_Fisch0070030.1	3.977756082	3.935102664	1.010839214	0.312093391	NA
Glycyrrhiza_uralensis_Fisch0250980.1	3.969134664	3.935271925	1.00860493	0.313164142	NA
Glycyrrhiza_uralensis_Fisch0075730.1	3.966904892	3.935315865	1.008027063	0.31344147	NA
Glycyrrhiza_uralensis_Fisch0272950.1	3.955976899	3.93553219	1.005194903	0.314803009	NA
Glycyrrhiza_uralensis_Fisch0117410.1	3.944970904	3.93575171	1.002342423	0.31617824	NA
Glycyrrhiza_uralensis_Fisch0251530.1	3.922855593	3.936197867	0.996610365	0.318953673	NA
Glycyrrhiza_uralensis_Fisch0266580.1	3.91516004	1.575312784	2.48532233	0.012943418	0.28143457
Glycyrrhiza_uralensis_Fisch0155240.1	3.910406222	1.703300895	2.29578123	0.021688393	0.360773756
Glycyrrhiza_uralensis_Fisch0236320.1	3.900145512	3.936663142	0.99072371	0.32182051	NA
Glycyrrhiza_uralensis_Fisch0030840.1	3.900145512	3.936663142	0.99072371	0.32182051	NA
Glycyrrhiza_uralensis_Fisch0235140.1	3.900145512	3.936663142	0.99072371	0.32182051	NA
Glycyrrhiza_uralensis_Fisch0198470.1	3.90004936	1.188168673	3.282403794	0.001029261	0.067874829
Glycyrrhiza_uralensis_Fisch0212970.1	3.886522653	3.936945749	0.987192331	0.323548361	NA
Glycyrrhiza_uralensis_Fisch0243990.1	3.879216104	1.907866626	2.03327426	0.042024822	0.496099003
Glycyrrhiza_uralensis_Fisch0025390.1	3.877373359	3.937137044	0.984820522	0.324712238	NA
Glycyrrhiza_uralensis_Fisch0206130.1	3.828999248	3.938168707	0.972279131	0.330911703	NA
Glycyrrhiza_uralensis_Fisch0119940.1	3.828999248	3.938168707	0.972279131	0.330911703	NA
Glycyrrhiza_uralensis_Fisch0	3.828999248	3.938168	0.9722791	0.330911	NA

035190.1		707	31	703	
Glycyrrhiza_uralensis_Fisch0034010.1	3.828999248	3.938168707	0.972279131	0.330911703	NA
Glycyrrhiza_uralensis_Fisch0032080.1	3.828999248	3.938168707	0.972279131	0.330911703	NA
Glycyrrhiza_uralensis_Fisch0010480.1	3.828999248	3.938168707	0.972279131	0.330911703	NA
Glycyrrhiza_uralensis_Fisch0077210.1	3.828999248	3.938168707	0.972279131	0.330911703	NA
Glycyrrhiza_uralensis_Fisch0060400.1	3.828999248	3.938168707	0.972279131	0.330911703	NA
Glycyrrhiza_uralensis_Fisch0277280.1	3.828999248	3.938168707	0.972279131	0.330911703	NA
Glycyrrhiza_uralensis_Fisch0031990.1	3.828999248	3.938168707	0.972279131	0.330911703	NA
Glycyrrhiza_uralensis_Fisch0114450.1	3.828999248	3.938168707	0.972279131	0.330911703	NA
Glycyrrhiza_uralensis_Fisch0254450.1	3.828999248	3.938168707	0.972279131	0.330911703	NA
Glycyrrhiza_uralensis_Fisch0042650.1	3.828999248	3.938168707	0.972279131	0.330911703	NA
Glycyrrhiza_uralensis_Fisch0231830.1	3.828999248	3.938168707	0.972279131	0.330911703	NA
Glycyrrhiza_uralensis_Fisch0107670.1	3.828999248	3.938168707	0.972279131	0.330911703	NA
Glycyrrhiza_uralensis_Fisch0244310.1	3.820021594	1.290662566	2.959736879	0.003079019	0.135277149
Glycyrrhiza_uralensis_Fisch0222240.1	3.81408754	1.751657835	2.177415854	0.029449554	0.422462527
Glycyrrhiza_uralensis_Fisch0136540.1	3.805489963	1.707860757	2.228220273	0.02586583	0.393546475
Glycyrrhiza_uralensis_Fisch0241200.1	3.78760909	3.665808882	1.033226011	0.301498143	NA
Glycyrrhiza_uralensis_Fisch0136130.1	3.780434968	3.939239523	0.959686494	0.337213023	NA
Glycyrrhiza_uralensis_Fisch0147400.1	3.77648131	1.170375601	3.22672594	0.001252153	0.077082685
Glycyrrhiza_uralensis_Fisch0002940.1	3.76068898	3.939685237	0.954565848	0.339797295	NA
Glycyrrhiza_uralensis_Fisch0097790.1	3.76068898	3.939685237	0.954565848	0.339797295	NA
Glycyrrhiza_uralensis_Fisch0079860.1	3.76068898	3.939685237	0.954565848	0.339797295	NA
Glycyrrhiza_uralensis_Fisch0	3.760109805	2.046512	1.8373255	0.066161	0.589381

028420.1		572	35	853	612
Glycyrrhiza_uralensis_Fisch0 084000.1	3.744057129	0.582982 383	6.4222474 61	1.34E-10	2.02E-07
Glycyrrhiza_uralensis_Fisch0 189690.1	3.73593432	0.942294 871	3.9647189 38	NA	NA
Glycyrrhiza_uralensis_Fisch0 284230.1	3.73075363	2.061906 762	1.8093706 75	0.070393 436	0.601523 553
Glycyrrhiza_uralensis_Fisch0 276690.1	3.730305177	3.940382 997	0.9466859 39	0.343798 838	NA
Glycyrrhiza_uralensis_Fisch0 137640.1	3.730305177	3.940382 997	0.9466859 39	0.343798 838	NA
Glycyrrhiza_uralensis_Fisch0 013730.1	3.730305177	3.940382 997	0.9466859 39	0.343798 838	NA
Glycyrrhiza_uralensis_Fisch0 283910.1	3.730305177	3.940382 997	0.9466859 39	0.343798 838	NA
Glycyrrhiza_uralensis_Fisch0 141370.1	3.730305177	3.940382 997	0.9466859 39	0.343798 838	NA
Glycyrrhiza_uralensis_Fisch0 187160.1	3.730305177	3.940382 997	0.9466859 39	0.343798 838	NA
Glycyrrhiza_uralensis_Fisch0 110320.1	3.730305177	3.940382 997	0.9466859 39	0.343798 838	NA
Glycyrrhiza_uralensis_Fisch0 149880.1	3.730305177	3.940382 997	0.9466859 39	0.343798 838	NA
Glycyrrhiza_uralensis_Fisch0 148520.1	3.730305177	3.940382 997	0.9466859 39	0.343798 838	NA
Glycyrrhiza_uralensis_Fisch0 016470.1	3.730305177	3.940382 997	0.9466859 39	0.343798 838	NA
Glycyrrhiza_uralensis_Fisch0 016690.1	3.720140042	3.940619 709	0.9440494 94	0.345144 357	NA
Glycyrrhiza_uralensis_Fisch0 040520.1	3.715032441	3.940739 274	0.9427247 48	0.345821 711	NA
Glycyrrhiza_uralensis_Fisch0 046490.1	3.715032441	3.940739 274	0.9427247 48	0.345821 711	NA
Glycyrrhiza_uralensis_Fisch0 052380.1	3.709907981	3.940859 656	0.9413956 1	0.346502 162	NA
Glycyrrhiza_uralensis_Fisch0 217930.1	3.709907981	3.940859 656	0.9413956 1	0.346502 162	NA
Glycyrrhiza_uralensis_Fisch0 185610.1	3.709907981	3.940859 656	0.9413956 1	0.346502 162	NA
Glycyrrhiza_uralensis_Fisch0 041280.1	3.709907981	3.940859 656	0.9413956 1	0.346502 162	NA
Glycyrrhiza_uralensis_Fisch0 231000.1	3.709907981	3.940859 656	0.9413956 1	0.346502 162	NA
Glycyrrhiza_uralensis_Fisch0	3.709907981	3.940859	0.9413956	0.346502	NA

022190.1		656	1	162	
Glycyrrhiza_uralensis_Fisch0 174150.1	3.709907981	3.940859 656	0.9413956 1	0.346502 162	NA
Glycyrrhiza_uralensis_Fisch0 113450.1	3.701579802	0.690588 113	5.3600398 45	8.32E-08	5.01E-05
Glycyrrhiza_uralensis_Fisch0 111610.1	3.69131112	1.411755 039	2.6146966 15	0.008930 674	0.235195 537
Glycyrrhiza_uralensis_Fisch0 059600.1	3.689239645	3.941349 513	0.9360346 33	0.349255 357	NA
Glycyrrhiza_uralensis_Fisch0 286320.1	3.689239645	3.941349 513	0.9360346 33	0.349255 357	NA
Glycyrrhiza_uralensis_Fisch0 276300.1	3.689239645	3.941349 513	0.9360346 33	0.349255 357	NA
Glycyrrhiza_uralensis_Fisch0 046420.1	3.689239645	3.941349 513	0.9360346 33	0.349255 357	NA
Glycyrrhiza_uralensis_Fisch0 032240.1	3.689239645	3.941349 513	0.9360346 33	0.349255 357	NA
Glycyrrhiza_uralensis_Fisch0 002410.1	3.689239645	3.941349 513	0.9360346 33	0.349255 357	NA
Glycyrrhiza_uralensis_Fisch0 179770.1	3.689239645	3.941349 513	0.9360346 33	0.349255 357	NA
Glycyrrhiza_uralensis_Fisch0 101110.1	3.689239645	3.941349 513	0.9360346 33	0.349255 357	NA
Glycyrrhiza_uralensis_Fisch0 051750.1	3.689239645	3.941349 513	0.9360346 33	0.349255 357	NA
Glycyrrhiza_uralensis_Fisch0 167440.1	3.689239645	3.941349 513	0.9360346 33	0.349255 357	NA
Glycyrrhiza_uralensis_Fisch0 032850.1	3.689239645	3.941349 513	0.9360346 33	0.349255 357	NA
Glycyrrhiza_uralensis_Fisch0 084520.1	3.678863295	0.768232 346	4.7887378 25	1.68E-06	0.000592 039
Glycyrrhiza_uralensis_Fisch0 205460.1	3.645185925	1.227705 947	2.9691034 2	0.002986 701	0.133873 153
Glycyrrhiza_uralensis_Fisch0 051210.1	3.635963139	3.942644 756	0.9222142 4	0.356416 837	NA
Glycyrrhiza_uralensis_Fisch0 038220.1	3.635963139	3.942644 756	0.9222142 4	0.356416 837	NA
Glycyrrhiza_uralensis_Fisch0 003540.1	3.635963139	3.942644 756	0.9222142 4	0.356416 837	NA
Glycyrrhiza_uralensis_Fisch0 188050.1	3.635963139	3.942644 756	0.9222142 4	0.356416 837	NA
Glycyrrhiza_uralensis_Fisch0 150060.1	3.635963139	3.942644 756	0.9222142 4	0.356416 837	NA
Glycyrrhiza_uralensis_Fisch0	3.635963139	3.942644	0.9222142	0.356416	NA

248500.1		756	4	837	
Glycyrrhiza_uralensis_Fisch0<br>101290.1	3.635963139	3.942644<br>756	0.9222142<br>4	0.356416<br>837	NA
Glycyrrhiza_uralensis_Fisch0<br>049100.1	3.614024413	3.418721<br>364	1.0571275<br>13	0.290453<br>396	0.866905<br>372
Glycyrrhiza_uralensis_Fisch0<br>098650.1	3.602959141	0.620306<br>609	5.8083520<br>16	6.31E-09	5.42E-06
Glycyrrhiza_uralensis_Fisch0<br>024290.1	3.596222611	2.843077<br>731	1.2649047<br>79	0.205905<br>464	0.810963<br>52
Glycyrrhiza_uralensis_Fisch0<br>090850.1	3.594628124	3.943682<br>843	0.9114901<br>65	0.362037<br>166	NA
Glycyrrhiza_uralensis_Fisch0<br>135770.1	3.589659881	2.149700<br>816	1.6698416<br>15	0.094950<br>704	0.668264<br>168
Glycyrrhiza_uralensis_Fisch0<br>143420.1	3.58060422	3.944041<br>786	0.9078514<br>92	0.363956<br>689	NA
Glycyrrhiza_uralensis_Fisch0<br>033410.1	3.577872124	3.944112<br>118	0.9071426<br>01	0.364331<br>393	NA
Glycyrrhiza_uralensis_Fisch0<br>190500.1	3.577872124	3.944112<br>118	0.9071426<br>01	0.364331<br>393	NA
Glycyrrhiza_uralensis_Fisch0<br>249070.1	3.56860835	1.180537<br>808	3.0228666<br>35	0.002503<br>926	0.117944<br>208
Glycyrrhiza_uralensis_Fisch0<br>019450.1	3.558020017	3.944627<br>145	0.9019914<br>65	0.367061<br>402	NA
Glycyrrhiza_uralensis_Fisch0<br>286110.1	3.537767398	3.945159<br>845	0.8967361<br>37	0.369859<br>733	NA
Glycyrrhiza_uralensis_Fisch0<br>260240.1	3.537767398	3.945159<br>845	0.8967361<br>37	0.369859<br>733	NA
Glycyrrhiza_uralensis_Fisch0<br>279180.1	3.537767398	3.945159<br>845	0.8967361<br>37	0.369859<br>733	NA
Glycyrrhiza_uralensis_Fisch0<br>047700.1	3.537767398	3.945159<br>845	0.8967361<br>37	0.369859<br>733	NA
Glycyrrhiza_uralensis_Fisch0<br>131590.1	3.537767398	3.945159<br>845	0.8967361<br>37	0.369859<br>733	NA
Glycyrrhiza_uralensis_Fisch0<br>218330.1	3.537767398	3.945159<br>845	0.8967361<br>37	0.369859<br>733	NA
Glycyrrhiza_uralensis_Fisch0<br>277230.1	3.526204098	3.945467<br>329	0.8937354<br>7	0.371463<br>443	NA
Glycyrrhiza_uralensis_Fisch0<br>206430.1	3.524866026	0.526845<br>27	6.6905147<br>03	2.22E-11	4.51E-08
Glycyrrhiza_uralensis_Fisch0<br>098380.1	3.520390339	3.945622<br>849	0.8922267<br>72	0.372271<br>395	NA
Glycyrrhiza_uralensis_Fisch0<br>244780.1	3.514554982	3.945779<br>572	0.8907124<br>48	0.373083<br>455	NA
Glycyrrhiza_uralensis_Fisch0	3.514554982	3.945779	0.8907124	0.373083	NA

023350.1		572	48	455	
Glycyrrhiza_uralensis_Fisch0036770.1	3.500050471	3.273479585	1.069214082	0.284973215	NA
Glycyrrhiza_uralensis_Fisch0030750.1	3.496917826	3.946257095	0.886135328	0.375544608	NA
Glycyrrhiza_uralensis_Fisch0038810.1	3.496917826	3.946257095	0.886135328	0.375544608	NA
Glycyrrhiza_uralensis_Fisch0049610.1	3.488790159	1.058174756	3.29698865	0.000977275	0.065341659
Glycyrrhiza_uralensis_Fisch0254650.1	3.433203123	0.728914387	4.710022441	2.48E-06	0.000764344
Glycyrrhiza_uralensis_Fisch0155820.1	3.421382675	1.033782874	3.309575697	0.000934375	0.064075229
Glycyrrhiza_uralensis_Fisch0155830.1	3.418263057	1.768798256	1.932534164	0.05329361	0.544010679
Glycyrrhiza_uralensis_Fisch0021190.1	3.415281926	1.37710698	2.480041112	0.013136723	0.282914881
Glycyrrhiza_uralensis_Fisch0153990.1	3.413055748	3.948608401	0.864369267	0.38738507	NA
Glycyrrhiza_uralensis_Fisch0219400.1	3.413055748	3.948608401	0.864369267	0.38738507	NA
Glycyrrhiza_uralensis_Fisch0044200.1	3.413055748	3.948608401	0.864369267	0.38738507	NA
Glycyrrhiza_uralensis_Fisch0147710.1	3.413055748	3.948608401	0.864369267	0.38738507	NA
Glycyrrhiza_uralensis_Fisch0046460.1	3.413055748	3.948608401	0.864369267	0.38738507	NA
Glycyrrhiza_uralensis_Fisch0110000.1	3.413055748	3.948608401	0.864369267	0.38738507	NA
Glycyrrhiza_uralensis_Fisch0152600.1	3.413055748	3.948608401	0.864369267	0.38738507	NA
Glycyrrhiza_uralensis_Fisch0152390.1	3.413055748	3.948608401	0.864369267	0.38738507	NA
Glycyrrhiza_uralensis_Fisch0002480.1	3.413055748	3.948608401	0.864369267	0.38738507	NA
Glycyrrhiza_uralensis_Fisch0175210.1	3.413055748	3.948608401	0.864369267	0.38738507	NA
Glycyrrhiza_uralensis_Fisch0152580.1	3.413055748	3.948608401	0.864369267	0.38738507	NA
Glycyrrhiza_uralensis_Fisch0088650.1	3.413055748	3.948608401	0.864369267	0.38738507	NA
Glycyrrhiza_uralensis_Fisch0224790.1	3.402471235	1.923269405	1.769107971	0.076875858	0.623502421
Glycyrrhiza_uralensis_Fisch0	3.397207223	3.949068	0.8602554	0.389648	NA

135910.1		154	05	27	
Glycyrrhiza_uralensis_Fisch0228920.1	3.379997251	0.407279853	8.298955201	1.05E-16	4.21E-13
Glycyrrhiza_uralensis_Fisch0089470.1	3.377425199	1.878652827	1.797791029	0.07221013	0.606540739
Glycyrrhiza_uralensis_Fisch0054070.1	3.368336963	3.949918605	0.852761107	0.393791796	NA
Glycyrrhiza_uralensis_Fisch0266620.1	3.35821754	3.542959922	0.947856485	0.34320252	0.894315373
Glycyrrhiza_uralensis_Fisch0032970.1	3.351982107	1.43780694	2.331315849	0.019736711	0.34325335
Glycyrrhiza_uralensis_Fisch0002260.1	3.34533659	0.497736749	6.721096233	1.80E-11	4.34E-08
Glycyrrhiza_uralensis_Fisch0240010.1	3.335222551	2.369894084	1.407329794	0.159329619	0.763804804
Glycyrrhiza_uralensis_Fisch0097970.1	3.332134996	1.024783827	3.251549165	0.001147779	0.07323834
Glycyrrhiza_uralensis_Fisch0155320.1	3.332076646	2.601029485	1.281060697	0.200172347	0.806142846
Glycyrrhiza_uralensis_Fisch0193130.1	3.327004444	0.748979463	4.442050295	NA	NA
Glycyrrhiza_uralensis_Fisch0245500.1	3.322325076	3.951309281	0.840816256	0.400450879	NA
Glycyrrhiza_uralensis_Fisch0263280.1	3.315515381	3.95151885	0.839048352	0.401442177	NA
Glycyrrhiza_uralensis_Fisch0238410.1	3.315515381	3.95151885	0.839048352	0.401442177	NA
Glycyrrhiza_uralensis_Fisch0237190.1	3.315515381	3.95151885	0.839048352	0.401442177	NA
Glycyrrhiza_uralensis_Fisch0111980.1	3.315515381	3.95151885	0.839048352	0.401442177	NA
Glycyrrhiza_uralensis_Fisch0108870.1	3.315515381	3.95151885	0.839048352	0.401442177	NA
Glycyrrhiza_uralensis_Fisch0117520.1	3.315515381	3.95151885	0.839048352	0.401442177	NA
Glycyrrhiza_uralensis_Fisch0047170.1	3.315515381	3.95151885	0.839048352	0.401442177	NA
Glycyrrhiza_uralensis_Fisch0018250.1	3.315515381	3.95151885	0.839048352	0.401442177	NA
Glycyrrhiza_uralensis_Fisch0272770.1	3.314136346	0.672286278	4.929650438	8.24E-07	0.000319808
Glycyrrhiza_uralensis_Fisch0087410.1	3.310949942	1.240184951	2.669722721	NA	NA
Glycyrrhiza_uralensis_Fisch0	3.302108334	3.951934	0.8355676	0.403398	NA

116790.1		322	15	194	
Glycyrrhiza_uralensis_Fisch0224480.1	3.297313866	1.265106406	2.606352993	0.00915121	0.236747677
Glycyrrhiza_uralensis_Fisch0270180.1	3.295577459	0.692047297	4.76206969	1.92E-06	0.000623275
Glycyrrhiza_uralensis_Fisch0069390.1	3.29536102	3.952144863	0.833815848	0.404384763	NA
Glycyrrhiza_uralensis_Fisch0087750.1	3.287879993	1.047266136	3.139488501	0.001692431	0.093433036
Glycyrrhiza_uralensis_Fisch0116660.1	3.274949101	3.952787748	0.828516305	0.407378165	NA
Glycyrrhiza_uralensis_Fisch0154330.1	3.274949101	3.952787748	0.828516305	0.407378165	NA
Glycyrrhiza_uralensis_Fisch0228330.1	3.274949101	3.952787748	0.828516305	0.407378165	NA
Glycyrrhiza_uralensis_Fisch0113440.1	3.274949101	3.952787748	0.828516305	0.407378165	NA
Glycyrrhiza_uralensis_Fisch0112600.1	3.274949101	3.952787748	0.828516305	0.407378165	NA
Glycyrrhiza_uralensis_Fisch0257760.1	3.274949101	3.952787748	0.828516305	0.407378165	NA
Glycyrrhiza_uralensis_Fisch0155720.1	3.274949101	3.952787748	0.828516305	0.407378165	NA
Glycyrrhiza_uralensis_Fisch0220520.1	3.274949101	3.952787748	0.828516305	0.407378165	NA
Glycyrrhiza_uralensis_Fisch0258400.1	3.274949101	3.952787748	0.828516305	0.407378165	NA
Glycyrrhiza_uralensis_Fisch0131550.1	3.274949101	3.952787748	0.828516305	0.407378165	NA
Glycyrrhiza_uralensis_Fisch0056410.1	3.26690973	0.763942539	4.276381483	1.90E-05	0.003544366
Glycyrrhiza_uralensis_Fisch0049560.1	3.252792288	1.305150483	2.49227375	0.012692817	0.278755565
Glycyrrhiza_uralensis_Fisch0015110.1	3.248134209	1.301269704	2.496126822	0.01255577	0.277327763
Glycyrrhiza_uralensis_Fisch0078000.1	3.239217402	1.848837883	1.752028899	0.079768839	0.632839799
Glycyrrhiza_uralensis_Fisch0023750.1	3.234029833	1.259586188	2.56753358	0.010242487	0.250037179
Glycyrrhiza_uralensis_Fisch0231100.1	3.229662971	1.067721018	3.024819139	0.002487818	0.117415276
Glycyrrhiza_uralensis_Fisch0014760.1	3.20389474	0.323522221	9.903167492	4.03E-23	2.43E-19
Glycyrrhiza_uralensis_Fisch0	3.202707767	2.312774	1.3847905	0.166116	0.772572

018760.1		212	04	535	421
Glycyrrhiza_uralensis_Fisch0 241850.1	3.199703961	2.216934 227	1.4433012 59	0.148935 625	0.750917 574
Glycyrrhiza_uralensis_Fisch0 101630.1	3.199577708	2.057850 631	1.5548153 3	0.119990 057	0.711449 612
Glycyrrhiza_uralensis_Fisch0 040690.1	3.183946341	1.746877 407	1.8226501 35	0.068356 397	0.597002 351
Glycyrrhiza_uralensis_Fisch0 094260.1	3.182226616	1.115759 963	2.8520709 85	0.004343 54	0.162595 657
Glycyrrhiza_uralensis_Fisch0 243140.1	3.17848653	1.214682 016	2.6167231 33	NA	NA
Glycyrrhiza_uralensis_Fisch0 223710.1	3.169617079	0.725487 704	4.3689466 55	1.25E-05	0.002584 674
Glycyrrhiza_uralensis_Fisch0 027170.1	3.163339175	0.673405 36	4.6975259 81	2.63E-06	0.000792 299
Glycyrrhiza_uralensis_Fisch0 131690.1	3.160463227	2.745355 165	1.1512037 74	0.249648 413	0.844321 666
Glycyrrhiza_uralensis_Fisch0 236390.1	3.149306848	3.956949 104	0.7958926 85	0.426094 413	NA
Glycyrrhiza_uralensis_Fisch0 236750.1	3.149306848	3.956949 104	0.7958926 85	0.426094 413	NA
Glycyrrhiza_uralensis_Fisch0 179740.1	3.149306848	3.956949 104	0.7958926 85	0.426094 413	NA
Glycyrrhiza_uralensis_Fisch0 054550.1	3.149306848	3.956949 104	0.7958926 85	0.426094 413	NA
Glycyrrhiza_uralensis_Fisch0 227920.1	3.149306848	3.956949 104	0.7958926 85	0.426094 413	NA
Glycyrrhiza_uralensis_Fisch0 172510.1	3.149306848	3.956949 104	0.7958926 85	0.426094 413	NA
Glycyrrhiza_uralensis_Fisch0 132660.1	3.141336276	1.866502 826	1.6830064 4	0.092373 845	0.661782 054
Glycyrrhiza_uralensis_Fisch0 273920.1	3.140516651	3.456180 196	0.9086669 31	0.363525 965	NA
Glycyrrhiza_uralensis_Fisch0 005110.1	3.123481753	1.016538 245	3.0726652 64	0.002121 563	0.107734 233
Glycyrrhiza_uralensis_Fisch0 172090.1	3.120449333	1.189261 208	2.6238553 08	0.008694 071	0.232899 664
Glycyrrhiza_uralensis_Fisch0 009320.1	3.10185333	2.483606 997	1.2489308 23	0.211690 377	0.813314 506
Glycyrrhiza_uralensis_Fisch0 227860.1	3.066339885	0.905671 316	3.3857093 97	0.000709 945	0.053069 508
Glycyrrhiza_uralensis_Fisch0 108550.1	3.063694746	0.642612 644	4.7675606 37	1.86E-06	0.000623 275
Glycyrrhiza_uralensis_Fisch0	3.052673967	3.960402	0.7707989	0.440826	NA

117940.1		133	91	086	
Glycyrrhiza_uralensis_Fisch0004130.1	3.052673967	3.960402133	0.770798991	0.440826086	NA
Glycyrrhiza_uralensis_Fisch0083670.1	3.052673967	3.960402133	0.770798991	0.440826086	NA
Glycyrrhiza_uralensis_Fisch0117490.1	3.052673967	3.960402133	0.770798991	0.440826086	NA
Glycyrrhiza_uralensis_Fisch0036440.1	3.052673967	3.960402133	0.770798991	0.440826086	NA
Glycyrrhiza_uralensis_Fisch0226010.1	3.052673967	3.960402133	0.770798991	0.440826086	NA
Glycyrrhiza_uralensis_Fisch0151460.1	3.052673967	3.960402133	0.770798991	0.440826086	NA
Glycyrrhiza_uralensis_Fisch0150830.1	3.052673967	3.960402133	0.770798991	0.440826086	NA
Glycyrrhiza_uralensis_Fisch0177050.1	3.052673967	3.960402133	0.770798991	0.440826086	NA
Glycyrrhiza_uralensis_Fisch0213010.1	3.043952552	1.228156098	2.478473671	0.013194585	0.283565766
Glycyrrhiza_uralensis_Fisch0186580.1	3.036722533	3.9609944	0.766656608	0.443285709	NA
Glycyrrhiza_uralensis_Fisch0080040.1	3.028687921	3.961295174	0.764570118	0.444527568	NA
Glycyrrhiza_uralensis_Fisch0173460.1	3.028687921	3.961295174	0.764570118	0.444527568	NA
Glycyrrhiza_uralensis_Fisch0024230.1	3.01249976	3.961906216	0.760366247	0.447035693	NA
Glycyrrhiza_uralensis_Fisch0131260.1	3.01249976	3.961906216	0.760366247	0.447035693	NA
Glycyrrhiza_uralensis_Fisch0063600.1	3.01249976	3.961906216	0.760366247	0.447035693	NA
Glycyrrhiza_uralensis_Fisch0212960.1	3.01249976	3.961906216	0.760366247	0.447035693	NA
Glycyrrhiza_uralensis_Fisch0038390.1	3.01249976	3.961906216	0.760366247	0.447035693	NA
Glycyrrhiza_uralensis_Fisch0042240.1	3.01249976	3.961906216	0.760366247	0.447035693	NA
Glycyrrhiza_uralensis_Fisch0088600.1	3.01249976	3.961906216	0.760366247	0.447035693	NA
Glycyrrhiza_uralensis_Fisch0181730.1	3.010931838	3.676503671	0.818966091	0.412805761	0.911978305
Glycyrrhiza_uralensis_Fisch0235320.1	3.01092085	2.717567861	1.107946886	0.267884755	0.85529651
Glycyrrhiza_uralensis_Fisch0	3.001871106	0.775132	3.8727195	0.000107	0.013779

023650.1		592	03	628	777
Glycyrrhiza_uralensis_Fisch0106620.1	2.985738409	2.794830072	1.068307673	0.285381749	0.862094057
Glycyrrhiza_uralensis_Fisch0153660.1	2.985632788	2.045595171	1.459542352	0.144415893	0.744212491
Glycyrrhiza_uralensis_Fisch0167070.1	2.985445178	1.413892274	2.111508234	0.034728651	0.454303602
Glycyrrhiza_uralensis_Fisch0078610.1	2.96655663	0.839445517	3.533947791	0.000409402	0.036229061
Glycyrrhiza_uralensis_Fisch0178930.1	2.963192623	0.466244641	6.355445972	2.08E-10	2.94E-07
Glycyrrhiza_uralensis_Fisch0151170.1	2.95800933	3.725216266	0.794050363	0.427166112	0.916400488
Glycyrrhiza_uralensis_Fisch0179580.1	2.945593928	2.974271082	0.990358258	0.32199904	NA
Glycyrrhiza_uralensis_Fisch0033800.1	2.922074229	1.167580296	2.502675182	0.012325861	0.274652222
Glycyrrhiza_uralensis_Fisch0184350.1	2.917862916	2.125700986	1.372659154	0.169858333	0.778611707
Glycyrrhiza_uralensis_Fisch0209360.1	2.910252224	2.022392291	1.439014694	0.150146359	0.753863762
Glycyrrhiza_uralensis_Fisch0272800.1	2.909458047	1.983110601	1.467118397	0.14234385	0.743052804
Glycyrrhiza_uralensis_Fisch0097650.1	2.900741304	1.420011109	2.042759585	0.041076243	0.492628387
Glycyrrhiza_uralensis_Fisch0207140.1	2.892656161	2.703689223	1.069892255	0.284667809	0.862094057
Glycyrrhiza_uralensis_Fisch0118090.1	2.891960369	1.274896979	2.268387498	0.023305599	0.371430842
Glycyrrhiza_uralensis_Fisch0135840.1	2.875658351	0.832719876	3.453332186	0.000553707	0.045332382
Glycyrrhiza_uralensis_Fisch0126120.1	2.869813129	1.968975742	1.457515737	0.144974071	0.744212491
Glycyrrhiza_uralensis_Fisch0022240.1	2.849518609	0.862329461	3.304443066	0.000951653	0.06470703
Glycyrrhiza_uralensis_Fisch0122160.1	2.848252947	1.997559249	1.425866566	0.153906862	0.758333908
Glycyrrhiza_uralensis_Fisch0080690.1	2.846831369	2.009394343	1.416760916	0.15655284	0.760775523
Glycyrrhiza_uralensis_Fisch0221590.1	2.84575921	1.890941071	1.50494336	0.132338639	0.727923
Glycyrrhiza_uralensis_Fisch0093250.1	2.841862886	2.994104281	0.949152941	0.342542832	0.894315373
Glycyrrhiza_uralensis_Fisch0	2.828174935	1.762787	1.6043764	0.108631	0.694857

160330.1		618	47	099	976
Glycyrrhiza_uralensis_Fisch0111550.1	2.826331346	3.969441226	0.71202247	0.476450862	NA
Glycyrrhiza_uralensis_Fisch0167550.1	2.826331346	3.969441226	0.71202247	0.476450862	NA
Glycyrrhiza_uralensis_Fisch0142680.1	2.826331346	3.969441226	0.71202247	0.476450862	NA
Glycyrrhiza_uralensis_Fisch0180040.1	2.826331346	3.969441226	0.71202247	0.476450862	NA
Glycyrrhiza_uralensis_Fisch0013970.1	2.826331346	3.969441226	0.71202247	0.476450862	NA
Glycyrrhiza_uralensis_Fisch0237170.1	2.826331346	3.969441226	0.71202247	0.476450862	NA
Glycyrrhiza_uralensis_Fisch0170470.1	2.826331346	3.969441226	0.71202247	0.476450862	NA
Glycyrrhiza_uralensis_Fisch0085820.1	2.826331346	3.969441226	0.71202247	0.476450862	NA
Glycyrrhiza_uralensis_Fisch0171280.1	2.826331346	3.969441226	0.71202247	0.476450862	NA
Glycyrrhiza_uralensis_Fisch0058870.1	2.826331346	3.969441226	0.71202247	0.476450862	NA
Glycyrrhiza_uralensis_Fisch0036320.1	2.826331346	3.969441226	0.71202247	0.476450862	NA
Glycyrrhiza_uralensis_Fisch0286760.1	2.826331346	3.969441226	0.71202247	0.476450862	NA
Glycyrrhiza_uralensis_Fisch0171540.1	2.826331346	3.969441226	0.71202247	0.476450862	NA
Glycyrrhiza_uralensis_Fisch0105060.1	2.826331346	3.969441226	0.71202247	0.476450862	NA
Glycyrrhiza_uralensis_Fisch0004530.1	2.826331346	3.969441226	0.71202247	0.476450862	NA
Glycyrrhiza_uralensis_Fisch0132530.1	2.826331346	3.969441226	0.71202247	0.476450862	NA
Glycyrrhiza_uralensis_Fisch0095680.1	2.826331346	3.969441226	0.71202247	0.476450862	NA
Glycyrrhiza_uralensis_Fisch0237280.1	2.826331346	3.969441226	0.71202247	0.476450862	NA
Glycyrrhiza_uralensis_Fisch0286630.1	2.826331346	3.969441226	0.71202247	0.476450862	NA
Glycyrrhiza_uralensis_Fisch0234890.1	2.826331346	3.969441226	0.71202247	0.476450862	NA
Glycyrrhiza_uralensis_Fisch0286160.1	2.826331346	3.969441226	0.71202247	0.476450862	NA
Glycyrrhiza_uralensis_Fisch0	2.826331346	3.969441	0.7120224	0.476450	NA

186560.1		226	7	862	
Glycyrrhiza_uralensis_Fisch0 043630.1	2.826331346	3.969441 226	0.7120224 7	0.476450 862	NA
Glycyrrhiza_uralensis_Fisch0 014890.1	2.826331346	3.969441 226	0.7120224 7	0.476450 862	NA
Glycyrrhiza_uralensis_Fisch0 205720.1	2.826331346	3.969441 226	0.7120224 7	0.476450 862	NA
Glycyrrhiza_uralensis_Fisch0 137210.1	2.826331346	3.969441 226	0.7120224 7	0.476450 862	NA
Glycyrrhiza_uralensis_Fisch0 051290.1	2.826331346	3.969441 226	0.7120224 7	0.476450 862	NA
Glycyrrhiza_uralensis_Fisch0 079700.1	2.826331346	3.969441 226	0.7120224 7	0.476450 862	NA
Glycyrrhiza_uralensis_Fisch0 209850.1	2.826331346	3.969441 226	0.7120224 7	0.476450 862	NA
Glycyrrhiza_uralensis_Fisch0 055900.1	2.826331346	3.969441 226	0.7120224 7	0.476450 862	NA
Glycyrrhiza_uralensis_Fisch0 286260.1	2.826331346	3.969441 226	0.7120224 7	0.476450 862	NA
Glycyrrhiza_uralensis_Fisch0 218290.1	2.826331346	3.969441 226	0.7120224 7	0.476450 862	NA
Glycyrrhiza_uralensis_Fisch0 215150.1	2.826331346	3.969441 226	0.7120224 7	0.476450 862	NA
Glycyrrhiza_uralensis_Fisch0 033070.1	2.826331346	3.969441 226	0.7120224 7	0.476450 862	NA
Glycyrrhiza_uralensis_Fisch0 110930.1	2.826331346	3.969441 226	0.7120224 7	0.476450 862	NA
Glycyrrhiza_uralensis_Fisch0 209740.1	2.826331346	3.969441 226	0.7120224 7	0.476450 862	NA
Glycyrrhiza_uralensis_Fisch0 070730.1	2.826331346	3.969441 226	0.7120224 7	0.476450 862	NA
Glycyrrhiza_uralensis_Fisch0 247710.1	2.826331346	3.969441 226	0.7120224 7	0.476450 862	NA
Glycyrrhiza_uralensis_Fisch0 112230.1	2.826331346	3.969441 226	0.7120224 7	0.476450 862	NA
Glycyrrhiza_uralensis_Fisch0 248160.1	2.826331346	3.969441 226	0.7120224 7	0.476450 862	NA
Glycyrrhiza_uralensis_Fisch0 025080.1	2.826331346	3.969441 226	0.7120224 7	0.476450 862	NA
Glycyrrhiza_uralensis_Fisch0 097290.1	2.826331346	3.969441 226	0.7120224 7	0.476450 862	NA
Glycyrrhiza_uralensis_Fisch0 175430.1	2.826331346	3.969441 226	0.7120224 7	0.476450 862	NA
Glycyrrhiza_uralensis_Fisch0	2.826331346	3.969441	0.7120224	0.476450	NA

228810.1		226	7	862	
Glycyrrhiza_uralensis_Fisch0 079030.1	2.826331346	3.969441 226	0.7120224 7	0.476450 862	NA
Glycyrrhiza_uralensis_Fisch0 173530.1	2.826331346	3.969441 226	0.7120224 7	0.476450 862	NA
Glycyrrhiza_uralensis_Fisch0 200080.1	2.823649168	3.731816 059	0.7566421 07	0.449264 307	0.922628 42
Glycyrrhiza_uralensis_Fisch0 059060.1	2.821920093	1.470636 211	1.9188430 64	0.055004 198	0.549803 889
Glycyrrhiza_uralensis_Fisch0 109350.1	2.815893266	1.361581 29	2.0681051 42	0.038630 137	0.481526 362
Glycyrrhiza_uralensis_Fisch0 129300.1	2.81558903	0.917025 38	3.0703501 68	0.002138 079	0.107974 882
Glycyrrhiza_uralensis_Fisch0 251220.1	2.814648859	0.973414 5	2.8915214 02	0.003833 815	0.151030 969
Glycyrrhiza_uralensis_Fisch0 008500.1	2.813091348	1.295541 048	2.1713641 21	0.029903 659	0.425598 337
Glycyrrhiza_uralensis_Fisch0 115220.1	2.797395036	1.280092 308	2.1853072 77	0.028866 324	0.418058 016
Glycyrrhiza_uralensis_Fisch0 273690.1	2.784661786	1.349972 812	2.0627539 77	0.039136 009	0.481530 651
Glycyrrhiza_uralensis_Fisch0 124070.1	2.780441048	0.608184 409	4.5717072 08	4.84E-06	0.001252 071
Glycyrrhiza_uralensis_Fisch0 015260.1	2.768022774	1.784406 476	1.5512288 32	0.120846 855	0.712110 373
Glycyrrhiza_uralensis_Fisch0 244940.1	2.7602339	3.972355 228	0.6948607 92	0.487142 546	NA
Glycyrrhiza_uralensis_Fisch0 031600.1	2.758921466	0.835737 873	3.3011803 75	0.000962 79	0.065096 479
Glycyrrhiza_uralensis_Fisch0 252340.1	2.751413042	1.832304 938	1.5016130 69	0.133197 066	0.729217 799
Glycyrrhiza_uralensis_Fisch0 083590.1	2.748942313	0.754830 992	3.6417984 19	0.000270 74	0.027381 146
Glycyrrhiza_uralensis_Fisch0 055400.1	2.74653665	2.215917 403	1.2394580 44	0.215175 917	0.814737 191
Glycyrrhiza_uralensis_Fisch0 047080.1	2.746240101	1.199634 703	2.2892302 91	0.022065 975	0.364287 823
Glycyrrhiza_uralensis_Fisch0 031510.1	2.744425776	1.463233 28	1.8755900 47	0.060711 594	0.571804 506
Glycyrrhiza_uralensis_Fisch0 032020.1	2.734013486	2.485963 983	1.0997800 07	0.271427 985	NA
Glycyrrhiza_uralensis_Fisch0 152550.1	2.731026187	3.973685 32	0.6872779 21	0.491907 609	NA
Glycyrrhiza_uralensis_Fisch0	2.731026187	3.973685	0.6872779	0.491907	NA

278560.1		32	21	609	
Glycyrrhiza_uralensis_Fisch0 052940.1	2.731026187	3.973685 32	0.6872779 21	0.491907 609	NA
Glycyrrhiza_uralensis_Fisch0 001120.1	2.731026187	3.973685 32	0.6872779 21	0.491907 609	NA
Glycyrrhiza_uralensis_Fisch0 181100.1	2.731026187	3.973685 32	0.6872779 21	0.491907 609	NA
Glycyrrhiza_uralensis_Fisch0 274400.1	2.731026187	3.973685 32	0.6872779 21	0.491907 609	NA
Glycyrrhiza_uralensis_Fisch0 226380.1	2.731026187	3.973685 32	0.6872779 21	0.491907 609	NA
Glycyrrhiza_uralensis_Fisch0 230990.1	2.731026187	3.973685 32	0.6872779 21	0.491907 609	NA
Glycyrrhiza_uralensis_Fisch0 057900.1	2.731026187	3.973685 32	0.6872779 21	0.491907 609	NA
Glycyrrhiza_uralensis_Fisch0 283170.1	2.731026187	3.973685 32	0.6872779 21	0.491907 609	NA
Glycyrrhiza_uralensis_Fisch0 272650.1	2.731026187	3.973685 32	0.6872779 21	0.491907 609	NA
Glycyrrhiza_uralensis_Fisch0 241390.1	2.731026187	3.973685 32	0.6872779 21	0.491907 609	NA
Glycyrrhiza_uralensis_Fisch0 249210.1	2.731026187	3.973685 32	0.6872779 21	0.491907 609	NA
Glycyrrhiza_uralensis_Fisch0 026740.1	2.731026187	3.973685 32	0.6872779 21	0.491907 609	NA
Glycyrrhiza_uralensis_Fisch0 223300.1	2.731026187	3.973685 32	0.6872779 21	0.491907 609	NA
Glycyrrhiza_uralensis_Fisch0 058760.1	2.731026187	3.973685 32	0.6872779 21	0.491907 609	NA
Glycyrrhiza_uralensis_Fisch0 006140.1	2.731026187	3.973685 32	0.6872779 21	0.491907 609	NA
Glycyrrhiza_uralensis_Fisch0 231740.1	2.731026187	3.973685 32	0.6872779 21	0.491907 609	NA
Glycyrrhiza_uralensis_Fisch0 278960.1	2.731026187	3.973685 32	0.6872779 21	0.491907 609	NA
Glycyrrhiza_uralensis_Fisch0 260140.1	2.731026187	3.973685 32	0.6872779 21	0.491907 609	NA
Glycyrrhiza_uralensis_Fisch0 035370.1	2.731026187	3.973685 32	0.6872779 21	0.491907 609	NA
Glycyrrhiza_uralensis_Fisch0 116890.1	2.731026187	3.973685 32	0.6872779 21	0.491907 609	NA
Glycyrrhiza_uralensis_Fisch0 102590.1	2.731026187	3.973685 32	0.6872779 21	0.491907 609	NA
Glycyrrhiza_uralensis_Fisch0	2.731026187	3.973685	0.6872779	0.491907	NA

110350.1		32	21	609	
Glycyrrhiza_uralensis_Fisch0051740.1	2.731026187	3.97368532	0.687277921	0.491907609	NA
Glycyrrhiza_uralensis_Fisch0216970.1	2.731026187	3.97368532	0.687277921	0.491907609	NA
Glycyrrhiza_uralensis_Fisch0240700.1	2.731026187	3.97368532	0.687277921	0.491907609	NA
Glycyrrhiza_uralensis_Fisch0006070.1	2.731026187	3.97368532	0.687277921	0.491907609	NA
Glycyrrhiza_uralensis_Fisch0117500.1	2.731026187	3.97368532	0.687277921	0.491907609	NA
Glycyrrhiza_uralensis_Fisch0101850.1	2.731026187	3.97368532	0.687277921	0.491907609	NA
Glycyrrhiza_uralensis_Fisch0102810.1	2.731026187	3.97368532	0.687277921	0.491907609	NA
Glycyrrhiza_uralensis_Fisch0028490.1	2.731026187	3.97368532	0.687277921	0.491907609	NA
Glycyrrhiza_uralensis_Fisch0225610.1	2.731026187	3.97368532	0.687277921	0.491907609	NA
Glycyrrhiza_uralensis_Fisch0159350.1	2.731026187	3.97368532	0.687277921	0.491907609	NA
Glycyrrhiza_uralensis_Fisch0279970.1	2.731026187	3.97368532	0.687277921	0.491907609	NA
Glycyrrhiza_uralensis_Fisch0018790.1	2.731026187	3.97368532	0.687277921	0.491907609	NA
Glycyrrhiza_uralensis_Fisch0118540.1	2.731026187	3.97368532	0.687277921	0.491907609	NA
Glycyrrhiza_uralensis_Fisch0231620.1	2.731026187	3.97368532	0.687277921	0.491907609	NA
Glycyrrhiza_uralensis_Fisch0236180.1	2.731026187	3.97368532	0.687277921	0.491907609	NA
Glycyrrhiza_uralensis_Fisch0064540.1	2.731026187	3.97368532	0.687277921	0.491907609	NA
Glycyrrhiza_uralensis_Fisch0036420.1	2.731026187	3.97368532	0.687277921	0.491907609	NA
Glycyrrhiza_uralensis_Fisch0100390.1	2.731026187	3.97368532	0.687277921	0.491907609	NA
Glycyrrhiza_uralensis_Fisch0206950.1	2.72898712	1.389157744	1.964490449	NA	NA
Glycyrrhiza_uralensis_Fisch0095800.1	2.724176104	0.596539377	4.566632497	4.96E-06	0.001269108
Glycyrrhiza_uralensis_Fisch0140200.1	2.719899768	2.050048919	1.326748714	0.184591821	0.792545039
Glycyrrhiza_uralensis_Fisch0	2.71900971	0.632344	4.2998861	1.71E-05	0.003284

078720.1		585	26		255
Glycyrrhiza_uralensis_Fisch0 235300.1	2.713197835	1.930007 267	1.4057966 93	0.159784 504	0.764089 972
Glycyrrhiza_uralensis_Fisch0 177840.1	2.711342004	3.974596 77	0.6821678 17	0.495132 842	NA
Glycyrrhiza_uralensis_Fisch0 282050.1	2.710323295	1.097997 071	2.4684248 87	0.013570 913	0.288157 19
Glycyrrhiza_uralensis_Fisch0 043510.1	2.696979504	1.178481 546	2.2885207 77	0.022107 212	0.364287 823
Glycyrrhiza_uralensis_Fisch0 045050.1	2.691423478	3.975531 597	0.6769971 29	0.498407 776	NA
Glycyrrhiza_uralensis_Fisch0 217150.1	2.691423478	3.975531 597	0.6769971 29	0.498407 776	NA
Glycyrrhiza_uralensis_Fisch0 134550.1	2.691423478	3.975531 597	0.6769971 29	0.498407 776	NA
Glycyrrhiza_uralensis_Fisch0 222910.1	2.691423478	3.975531 597	0.6769971 29	0.498407 776	NA
Glycyrrhiza_uralensis_Fisch0 102780.1	2.691423478	3.975531 597	0.6769971 29	0.498407 776	NA
Glycyrrhiza_uralensis_Fisch0 189210.1	2.691423478	3.975531 597	0.6769971 29	0.498407 776	NA
Glycyrrhiza_uralensis_Fisch0 254480.1	2.691423478	3.975531 597	0.6769971 29	0.498407 776	NA
Glycyrrhiza_uralensis_Fisch0 193440.1	2.691423478	3.975531 597	0.6769971 29	0.498407 776	NA
Glycyrrhiza_uralensis_Fisch0 036000.1	2.691423478	3.975531 597	0.6769971 29	0.498407 776	NA
Glycyrrhiza_uralensis_Fisch0 110960.1	2.691423478	3.975531 597	0.6769971 29	0.498407 776	NA
Glycyrrhiza_uralensis_Fisch0 278820.1	2.691423478	3.975531 597	0.6769971 29	0.498407 776	NA
Glycyrrhiza_uralensis_Fisch0 212900.1	2.691423478	3.975531 597	0.6769971 29	0.498407 776	NA
Glycyrrhiza_uralensis_Fisch0 276490.1	2.691423478	3.975531 597	0.6769971 29	0.498407 776	NA
Glycyrrhiza_uralensis_Fisch0 139530.1	2.691423478	3.975531 597	0.6769971 29	0.498407 776	NA
Glycyrrhiza_uralensis_Fisch0 204180.1	2.691423478	3.975531 597	0.6769971 29	0.498407 776	NA
Glycyrrhiza_uralensis_Fisch0 252060.1	2.691423478	3.975531 597	0.6769971 29	0.498407 776	NA
Glycyrrhiza_uralensis_Fisch0 241100.1	2.691423478	3.975531 597	0.6769971 29	0.498407 776	NA
Glycyrrhiza_uralensis_Fisch0	2.691423478	3.975531	0.6769971	0.498407	NA

052240.1		597	29	776	
Glycyrrhiza_uralensis_Fisch0112260.1	2.691423478	3.975531597	0.676997129	0.498407776	NA
Glycyrrhiza_uralensis_Fisch0087870.1	2.691423478	3.975531597	0.676997129	0.498407776	NA
Glycyrrhiza_uralensis_Fisch0149000.1	2.691423478	3.975531597	0.676997129	0.498407776	NA
Glycyrrhiza_uralensis_Fisch0037280.1	2.691423478	3.975531597	0.676997129	0.498407776	NA
Glycyrrhiza_uralensis_Fisch0110040.1	2.691423478	3.975531597	0.676997129	0.498407776	NA
Glycyrrhiza_uralensis_Fisch0263340.1	2.691423478	3.975531597	0.676997129	0.498407776	NA
Glycyrrhiza_uralensis_Fisch0155030.1	2.691423478	3.975531597	0.676997129	0.498407776	NA
Glycyrrhiza_uralensis_Fisch0120430.1	2.691423478	3.975531597	0.676997129	0.498407776	NA
Glycyrrhiza_uralensis_Fisch0139280.1	2.691423478	3.975531597	0.676997129	0.498407776	NA
Glycyrrhiza_uralensis_Fisch0125160.1	2.691423478	3.975531597	0.676997129	0.498407776	NA
Glycyrrhiza_uralensis_Fisch0226170.1	2.691423478	3.975531597	0.676997129	0.498407776	NA
Glycyrrhiza_uralensis_Fisch0037420.1	2.691423478	3.975531597	0.676997129	0.498407776	NA
Glycyrrhiza_uralensis_Fisch0006540.1	2.691423478	3.975531597	0.676997129	0.498407776	NA
Glycyrrhiza_uralensis_Fisch0281360.1	2.691423478	3.975531597	0.676997129	0.498407776	NA
Glycyrrhiza_uralensis_Fisch0253740.1	2.691423478	3.975531597	0.676997129	0.498407776	NA
Glycyrrhiza_uralensis_Fisch0114960.1	2.691423478	3.975531597	0.676997129	0.498407776	NA
Glycyrrhiza_uralensis_Fisch0236910.1	2.691423478	3.975531597	0.676997129	0.498407776	NA
Glycyrrhiza_uralensis_Fisch0106830.1	2.691423478	3.975531597	0.676997129	0.498407776	NA
Glycyrrhiza_uralensis_Fisch0271420.1	2.691423478	3.975531597	0.676997129	0.498407776	NA
Glycyrrhiza_uralensis_Fisch0064440.1	2.691423478	3.975531597	0.676997129	0.498407776	NA
Glycyrrhiza_uralensis_Fisch0269730.1	2.691423478	3.975531597	0.676997129	0.498407776	NA
Glycyrrhiza_uralensis_Fisch0	2.691423478	3.975531	0.6769971	0.498407	NA

200110.1		597	29	776	
Glycyrrhiza_uralensis_Fisch0267380.1	2.691423478	3.975531597	0.676997129	0.498407776	NA
Glycyrrhiza_uralensis_Fisch0004630.1	2.691423478	3.975531597	0.676997129	0.498407776	NA
Glycyrrhiza_uralensis_Fisch0020360.1	2.691423478	3.975531597	0.676997129	0.498407776	NA
Glycyrrhiza_uralensis_Fisch0245610.1	2.691423478	3.975531597	0.676997129	0.498407776	NA
Glycyrrhiza_uralensis_Fisch0035690.1	2.691423478	3.975531597	0.676997129	0.498407776	NA
Glycyrrhiza_uralensis_Fisch0140150.1	2.691423478	3.975531597	0.676997129	0.498407776	NA
Glycyrrhiza_uralensis_Fisch0099710.1	2.691423478	3.975531597	0.676997129	0.498407776	NA
Glycyrrhiza_uralensis_Fisch0249080.1	2.691423478	3.975531597	0.676997129	0.498407776	NA
Glycyrrhiza_uralensis_Fisch0041750.1	2.691423478	3.975531597	0.676997129	0.498407776	NA
Glycyrrhiza_uralensis_Fisch0007720.1	2.691423478	3.975531597	0.676997129	0.498407776	NA
Glycyrrhiza_uralensis_Fisch0041000.1	2.691423478	3.975531597	0.676997129	0.498407776	NA
Glycyrrhiza_uralensis_Fisch0046660.1	2.691423478	3.975531597	0.676997129	0.498407776	NA
Glycyrrhiza_uralensis_Fisch0054520.1	2.691423478	3.975531597	0.676997129	0.498407776	NA
Glycyrrhiza_uralensis_Fisch0215070.1	2.691423478	3.975531597	0.676997129	0.498407776	NA
Glycyrrhiza_uralensis_Fisch0075480.1	2.686498718	2.098617687	1.28012774	0.200500214	0.806142846
Glycyrrhiza_uralensis_Fisch0145710.1	2.685606679	0.263021161	10.21061068	1.78E-24	1.43E-20
Glycyrrhiza_uralensis_Fisch0174100.1	2.68217702	0.586225169	4.575335827	4.75E-06	0.001252053
Glycyrrhiza_uralensis_Fisch0236050.1	2.676783431	1.043924106	2.564155206	0.010342728	0.251647408
Glycyrrhiza_uralensis_Fisch0142210.1	2.655030881	2.795658906	0.949697717	0.34226587	0.894158318
Glycyrrhiza_uralensis_Fisch0248140.1	2.650443486	1.473475498	1.798769976	0.072055075	0.606209592
Glycyrrhiza_uralensis_Fisch0049580.1	2.648705269	1.114154683	2.377322744	0.017438822	0.323883064
Glycyrrhiza_uralensis_Fisch0	2.635256258	1.189966	2.2145634	0.026790	0.401151

077320.1		475	46	043	458
Glycyrrhiza_uralensis_Fisch0 005210.1	2.63347969	1.014099 515	2.5968651 49	0.009407 885	0.237615 733
Glycyrrhiza_uralensis_Fisch0 170970.1	2.626167072	3.016701 228	0.8705426 47	0.384003 924	NA
Glycyrrhiza_uralensis_Fisch0 232870.1	2.61883783	0.697009 669	3.7572474 93	0.000171 793	0.019784 915
Glycyrrhiza_uralensis_Fisch0 210450.1	2.617398085	2.020956 642	1.2951282 73	0.195275 983	0.802371 614
Glycyrrhiza_uralensis_Fisch0 043530.1	2.615063102	0.588375 997	4.4445441 6	8.81E-06	0.002015 265
Glycyrrhiza_uralensis_Fisch0 161230.1	2.61050179	1.981534 711	1.3174141 11	0.187699 853	0.797392 744
Glycyrrhiza_uralensis_Fisch0 250640.1	2.601994878	0.745782 754	3.4889448 22	0.000484 931	0.040924 599
Glycyrrhiza_uralensis_Fisch0 246370.1	2.601933873	1.614516 031	1.6115875 12	0.107051 73	0.692097 679
Glycyrrhiza_uralensis_Fisch0 031610.1	2.595195112	0.626800 253	4.1403861 92	3.47E-05	0.005877 175
Glycyrrhiza_uralensis_Fisch0 045870.1	2.591425436	0.580443 788	4.4645588 26	8.02E-06	0.001874 981
Glycyrrhiza_uralensis_Fisch0 156720.1	2.585549525	0.635523 766	4.0683758 26	4.73E-05	0.007212 161
Glycyrrhiza_uralensis_Fisch0 004540.1	2.57327811	1.576207 082	1.6325761 63	0.102558 168	0.687549 103
Glycyrrhiza_uralensis_Fisch0 272220.1	2.570730798	0.732465 57	3.5096950 66	0.000448 621	0.038428 128
Glycyrrhiza_uralensis_Fisch0 108290.1	2.569321754	0.503615 258	5.1017551 81	3.37E-07	0.00015
Glycyrrhiza_uralensis_Fisch0 127890.1	2.566202181	2.959745 416	0.8670347 68	0.385922 96	0.907171 672
Glycyrrhiza_uralensis_Fisch0 016060.1	2.565723707	2.132488 282	1.2031595 81	0.228914 573	0.826303 164
Glycyrrhiza_uralensis_Fisch0 041220.1	2.565407277	1.848997 206	1.3874587 09	0.165301 934	0.771334 974
Glycyrrhiza_uralensis_Fisch0 265480.1	2.564002127	1.926230 625	1.3310982 05	0.183156 699	0.791962 817
Glycyrrhiza_uralensis_Fisch0 059540.1	2.562778498	1.903018 27	1.3466914 84	0.178079 616	0.788665 611
Glycyrrhiza_uralensis_Fisch0 010720.1	2.550798694	0.478136 635	5.3348739 86	9.56E-08	5.41E-05
Glycyrrhiza_uralensis_Fisch0 047250.1	2.547046068	3.847229 694	0.6620467 9	0.507941 236	NA
Glycyrrhiza_uralensis_Fisch0	2.545061413	0.499615	5.0940374	3.51E-07	0.000153

249730.1		765	41		399
Glycyrrhiza_uralensis_Fisch0091090.1	2.534500911	1.306315557	1.940190407	0.052356554	0.538096607
Glycyrrhiza_uralensis_Fisch0171500.1	2.527213529	3.880563936	0.651249038	0.51488574	NA
Glycyrrhiza_uralensis_Fisch0264280.1	2.524748075	0.534183314	4.72637016	2.29E-06	0.0007145
Glycyrrhiza_uralensis_Fisch0104570.1	2.518672497	1.462380779	1.722309629	0.085013444	0.643781241
Glycyrrhiza_uralensis_Fisch0083600.1	2.518389556	1.912831514	1.31657678	0.187980526	0.797474632
Glycyrrhiza_uralensis_Fisch0053930.1	2.517489684	1.16369911	2.163351043	0.03051419	0.428423478
Glycyrrhiza_uralensis_Fisch0151660.1	2.514787595	3.28336221	0.76591842	0.443724845	NA
Glycyrrhiza_uralensis_Fisch0235980.1	2.510737332	1.468811569	1.709366528	0.087383077	0.648769483
Glycyrrhiza_uralensis_Fisch0287050.1	2.50664808	2.443974233	1.025644234	0.305059309	0.875571878
Glycyrrhiza_uralensis_Fisch0049750.1	2.50386125	0.507644351	4.932313823	8.13E-07	0.000319808
Glycyrrhiza_uralensis_Fisch0017070.1	2.503712577	2.555143919	0.97987145	0.327149577	NA
Glycyrrhiza_uralensis_Fisch0100530.1	2.495472858	1.099156155	2.27035335	0.023186153	0.37082438
Glycyrrhiza_uralensis_Fisch0202350.1	2.481406845	2.095864662	1.183953759	0.236431368	0.832730451
Glycyrrhiza_uralensis_Fisch0058960.1	2.478889767	0.693590616	3.573995537	0.000351575	0.032547725
Glycyrrhiza_uralensis_Fisch0241780.1	2.478698956	2.886284035	0.858785527	0.390458854	0.908672653
Glycyrrhiza_uralensis_Fisch0252680.1	2.478071498	1.30281708	1.902087051	0.057159775	0.558221775
Glycyrrhiza_uralensis_Fisch0257100.1	2.477410593	0.4527346	5.472103511	4.45E-08	3.06E-05
Glycyrrhiza_uralensis_Fisch0187640.1	2.47694975	1.370709305	1.80705693	0.070753403	0.602417549
Glycyrrhiza_uralensis_Fisch0192370.1	2.474351238	1.408116826	1.757205931	0.078882715	0.629619411
Glycyrrhiza_uralensis_Fisch0268660.1	2.473117913	0.794697071	3.112025959	0.001858082	0.099678948
Glycyrrhiza_uralensis_Fisch0143410.1	2.467776314	2.822927506	0.874190467	0.382014537	0.906340083
Glycyrrhiza_uralensis_Fisch0	2.466432747	2.672459	0.9229076	0.356055	0.897807

100780.1			1	355	124
Glycyrrhiza_uralensis_Fisch0232440.1	2.461270712	3.490883579	0.705056659	0.480774977	0.93190416
Glycyrrhiza_uralensis_Fisch0085450.1	2.446456241	0.941708732	2.597890576	0.009379838	0.237405572
Glycyrrhiza_uralensis_Fisch0028670.1	2.436798054	2.056767344	1.184770879	0.236108049	0.832447744
Glycyrrhiza_uralensis_Fisch0156020.1	2.434590869	0.691059949	3.522980706	0.000426722	0.03734985
Glycyrrhiza_uralensis_Fisch0280920.1	2.433593046	1.071010427	2.272240293	0.023072001	0.369719763
Glycyrrhiza_uralensis_Fisch0035290.1	2.431268243	0.614615116	3.955757318	7.63E-05	0.010493499
Glycyrrhiza_uralensis_Fisch0213000.1	2.431076138	1.406634187	1.728293084	0.083935692	0.64198669
Glycyrrhiza_uralensis_Fisch0046100.1	2.428732117	1.34666946	1.803510207	0.071308124	0.603936151
Glycyrrhiza_uralensis_Fisch0145820.1	2.410685668	2.242989762	1.074764455	0.282480198	0.86117142
Glycyrrhiza_uralensis_Fisch0114650.1	2.409606712	3.990210057	0.603879665	0.545923649	NA
Glycyrrhiza_uralensis_Fisch0241050.1	2.409606712	3.990210057	0.603879665	0.545923649	NA
Glycyrrhiza_uralensis_Fisch0109550.1	2.409606712	3.990210057	0.603879665	0.545923649	NA
Glycyrrhiza_uralensis_Fisch0222210.1	2.409606712	3.990210057	0.603879665	0.545923649	NA
Glycyrrhiza_uralensis_Fisch0183370.1	2.409606712	3.990210057	0.603879665	0.545923649	NA
Glycyrrhiza_uralensis_Fisch0139480.1	2.409606712	3.990210057	0.603879665	0.545923649	NA
Glycyrrhiza_uralensis_Fisch0256760.1	2.409606712	3.990210057	0.603879665	0.545923649	NA
Glycyrrhiza_uralensis_Fisch0264770.1	2.409606712	3.990210057	0.603879665	0.545923649	NA
Glycyrrhiza_uralensis_Fisch0209660.1	2.409606712	3.990210057	0.603879665	0.545923649	NA
Glycyrrhiza_uralensis_Fisch0123540.1	2.409606712	3.990210057	0.603879665	0.545923649	NA
Glycyrrhiza_uralensis_Fisch0102120.1	2.409606712	3.990210057	0.603879665	0.545923649	NA
Glycyrrhiza_uralensis_Fisch0276220.1	2.409606712	3.990210057	0.603879665	0.545923649	NA
Glycyrrhiza_uralensis_Fisch0	2.409606712	3.990210	0.6038796	0.545923	NA

112620.1		057	65	649	
Glycyrrhiza_uralensis_Fisch0 145340.1	2.409606712	3.990210 057	0.6038796 65	0.545923 649	NA
Glycyrrhiza_uralensis_Fisch0 153530.1	2.409606712	3.990210 057	0.6038796 65	0.545923 649	NA
Glycyrrhiza_uralensis_Fisch0 049740.1	2.409606712	3.990210 057	0.6038796 65	0.545923 649	NA
Glycyrrhiza_uralensis_Fisch0 221200.1	2.406342404	1.273979 028	1.8888398 88	0.058913 286	0.564515 005
Glycyrrhiza_uralensis_Fisch0 057050.1	2.400983806	2.318732 16	1.0354726 81	0.300448 218	0.872611 273
Glycyrrhiza_uralensis_Fisch0 023640.1	2.399322902	0.522327 251	4.5935242 65	4.36E-06	0.001192 073
Glycyrrhiza_uralensis_Fisch0 232610.1	2.394952645	0.658874 844	3.6349128 6	0.000278 075	0.027888 566
Glycyrrhiza_uralensis_Fisch0 034910.1	2.393585181	1.170723 345	2.0445352 79	0.040900 696	0.491748 127
Glycyrrhiza_uralensis_Fisch0 175940.1	2.390499379	1.074161 704	2.2254557 86	0.026050 656	0.394628 607
Glycyrrhiza_uralensis_Fisch0 154240.1	2.386061273	2.900649 594	0.8225954 9	0.410738 064	0.911783 198
Glycyrrhiza_uralensis_Fisch0 126970.1	2.384253034	1.176238 224	2.0270154 34	0.042660 834	0.498469 069
Glycyrrhiza_uralensis_Fisch0 071320.1	2.371813526	1.211798 94	1.9572665 46	0.050316 139	0.530561 439
Glycyrrhiza_uralensis_Fisch0 276410.1	2.370054028	0.898455 588	2.6379200 71	0.008341 624	0.230210 17
Glycyrrhiza_uralensis_Fisch0 177300.1	2.362896857	2.105056 117	1.1224863 97	0.261655 688	0.850912 419
Glycyrrhiza_uralensis_Fisch0 178090.1	2.361926187	1.385038 172	1.7053148 68	0.088135 716	0.650432 137
Glycyrrhiza_uralensis_Fisch0 022120.1	2.361271938	0.712748 268	3.3129115 05	0.000923 302	0.063496 79
Glycyrrhiza_uralensis_Fisch0 096510.1	2.360058192	2.228249 663	1.0591533 93	0.289529 927	0.866142 846
Glycyrrhiza_uralensis_Fisch0 254370.1	2.359157762	0.534390 884	4.4146669 27	1.01E-05	0.002205 848
Glycyrrhiza_uralensis_Fisch0 196300.1	2.349984476	1.118672 295	2.1006906 91	0.035668 127	0.459346 501
Glycyrrhiza_uralensis_Fisch0 247490.1	2.349519563	0.813198 825	2.8892313 8	0.003861 848	0.151886 733
Glycyrrhiza_uralensis_Fisch0 037050.1	2.349169547	1.876989 054	1.2515627 31	0.210729 242	0.812109 091
Glycyrrhiza_uralensis_Fisch0	2.348040247	3.993808	0.5879201	0.556585	NA

180720.1		189	34	901	
Glycyrrhiza_uralensis_Fisch0166560.1	2.346135995	1.439949157	1.629318634	0.103245583	0.689354006
Glycyrrhiza_uralensis_Fisch0163840.1	2.343229182	2.510395404	0.933410402	0.350608112	0.897130588
Glycyrrhiza_uralensis_Fisch0082090.1	2.343194182	0.913917108	2.563902306	0.010350267	0.251647408
Glycyrrhiza_uralensis_Fisch0129980.1	2.342666039	1.745586532	1.342050936	0.179579481	0.789067991
Glycyrrhiza_uralensis_Fisch0178710.1	2.342393344	0.584092087	4.010315145	6.06E-05	0.008739825
Glycyrrhiza_uralensis_Fisch0151280.1	2.340334661	3.951934104	0.592199819	0.553716791	NA
Glycyrrhiza_uralensis_Fisch0006080.1	2.338636793	1.118952188	2.09002388	0.036615655	0.466810809
Glycyrrhiza_uralensis_Fisch0265610.1	2.33622928	0.522950636	4.467399249	7.92E-06	0.001868405
Glycyrrhiza_uralensis_Fisch0180540.1	2.335466994	3.994561664	0.584661645	0.558775249	NA
Glycyrrhiza_uralensis_Fisch0163210.1	2.327222361	1.063067684	2.189157282	0.02858541	0.416736426
Glycyrrhiza_uralensis_Fisch0257510.1	2.321227509	0.682490478	3.401113398	0.00067112	0.051282076
Glycyrrhiza_uralensis_Fisch0223360.1	2.320880443	1.49915402	1.548126752	0.121591782	0.713911572
Glycyrrhiza_uralensis_Fisch0235430.1	2.320840633	1.039731226	2.232154402	0.02560476	0.392224196
Glycyrrhiza_uralensis_Fisch0223040.1	2.316985712	2.885997874	0.802836944	0.422068986	0.913773112
Glycyrrhiza_uralensis_Fisch0030070.1	2.316435643	3.995714439	0.579730028	0.56209669	NA
Glycyrrhiza_uralensis_Fisch0156160.1	2.316435643	3.995714439	0.579730028	0.56209669	NA
Glycyrrhiza_uralensis_Fisch0035870.1	2.316435643	3.995714439	0.579730028	0.56209669	NA
Glycyrrhiza_uralensis_Fisch0232880.1	2.316435643	3.995714439	0.579730028	0.56209669	NA
Glycyrrhiza_uralensis_Fisch0222710.1	2.316435643	3.995714439	0.579730028	0.56209669	NA
Glycyrrhiza_uralensis_Fisch0232390.1	2.316435643	3.995714439	0.579730028	0.56209669	NA
Glycyrrhiza_uralensis_Fisch0276040.1	2.316435643	3.995714439	0.579730028	0.56209669	NA
Glycyrrhiza_uralensis_Fisch0	2.316435643	3.995714	0.5797300	0.562096	NA

078760.1		439	28	69	
Glycyrrhiza_uralensis_Fisch0019870.1	2.316435643	3.995714439	0.579730028	0.56209669	NA
Glycyrrhiza_uralensis_Fisch0113570.1	2.316435643	3.995714439	0.579730028	0.56209669	NA
Glycyrrhiza_uralensis_Fisch0124910.1	2.316435643	3.995714439	0.579730028	0.56209669	NA
Glycyrrhiza_uralensis_Fisch0180430.1	2.316435643	3.995714439	0.579730028	0.56209669	NA
Glycyrrhiza_uralensis_Fisch0281210.1	2.316435643	3.995714439	0.579730028	0.56209669	NA
Glycyrrhiza_uralensis_Fisch0202020.1	2.316435643	3.995714439	0.579730028	0.56209669	NA
Glycyrrhiza_uralensis_Fisch0138920.1	2.316435643	3.995714439	0.579730028	0.56209669	NA
Glycyrrhiza_uralensis_Fisch0032580.1	2.316435643	3.995714439	0.579730028	0.56209669	NA
Glycyrrhiza_uralensis_Fisch0046090.1	2.316435643	3.995714439	0.579730028	0.56209669	NA
Glycyrrhiza_uralensis_Fisch0268760.1	2.316435643	3.995714439	0.579730028	0.56209669	NA
Glycyrrhiza_uralensis_Fisch0168560.1	2.316435643	3.995714439	0.579730028	0.56209669	NA
Glycyrrhiza_uralensis_Fisch0046740.1	2.316435643	3.995714439	0.579730028	0.56209669	NA
Glycyrrhiza_uralensis_Fisch0037460.1	2.307393926	0.344957588	6.688920626	2.25E-11	4.51E-08
Glycyrrhiza_uralensis_Fisch0092730.1	2.306934238	0.671236806	3.436841093	0.000588541	0.047537506
Glycyrrhiza_uralensis_Fisch0077610.1	2.305980925	3.390387238	0.680152668	0.496407799	NA
Glycyrrhiza_uralensis_Fisch0002150.1	2.302829537	1.711336759	1.345632019	0.17842122	0.788665611
Glycyrrhiza_uralensis_Fisch0073900.1	2.298304776	1.19185817	1.928337477	0.053813163	0.546162726
Glycyrrhiza_uralensis_Fisch0174690.1	2.298108489	1.072789447	2.142180364	0.032178971	0.440334185
Glycyrrhiza_uralensis_Fisch0189230.1	2.295902796	3.062940275	0.749574784	NA	NA
Glycyrrhiza_uralensis_Fisch0207180.1	2.294645038	1.426036001	1.60910737	0.107592866	0.692449274
Glycyrrhiza_uralensis_Fisch0168730.1	2.293084933	0.650042328	3.527593258	0.000419356	0.036973989
Glycyrrhiza_uralensis_Fisch0	2.291946919	0.779458	2.9404346	0.003277	0.138403

248700.1		553	26	522	41
Glycyrrhiza_uralensis_Fisch0109110.1	2.290739369	3.997294702	0.573072425	0.566595657	NA
Glycyrrhiza_uralensis_Fisch0268650.1	2.289564942	0.807117462	2.836718383	0.00455798	0.166987166
Glycyrrhiza_uralensis_Fisch0132490.1	2.289536329	0.827061088	2.768279588	0.005635309	0.18735067
Glycyrrhiza_uralensis_Fisch0039460.1	2.288287183	1.193167687	1.917825305	0.055133166	0.550416966
Glycyrrhiza_uralensis_Fisch0124060.1	2.283574556	0.985381	2.317453407	0.020479046	0.349348431
Glycyrrhiza_uralensis_Fisch0082560.1	2.283346135	0.930200257	2.45468233	0.014100914	0.292341937
Glycyrrhiza_uralensis_Fisch0138830.1	2.2812368	0.61771172	3.693044386	0.000221585	0.023599818
Glycyrrhiza_uralensis_Fisch0103700.1	2.278543153	0.563541165	4.043259472	5.27E-05	0.007784095
Glycyrrhiza_uralensis_Fisch0044780.1	2.277749413	3.998104085	0.569707382	0.568876182	NA
Glycyrrhiza_uralensis_Fisch0248250.1	2.277749413	3.998104085	0.569707382	0.568876182	NA
Glycyrrhiza_uralensis_Fisch0058030.1	2.277749413	3.998104085	0.569707382	0.568876182	NA
Glycyrrhiza_uralensis_Fisch0279030.1	2.277749413	3.998104085	0.569707382	0.568876182	NA
Glycyrrhiza_uralensis_Fisch0018870.1	2.277749413	3.998104085	0.569707382	0.568876182	NA
Glycyrrhiza_uralensis_Fisch0151160.1	2.277749413	3.998104085	0.569707382	0.568876182	NA
Glycyrrhiza_uralensis_Fisch0245860.1	2.277749413	3.998104085	0.569707382	0.568876182	NA
Glycyrrhiza_uralensis_Fisch0071710.1	2.277749413	3.998104085	0.569707382	0.568876182	NA
Glycyrrhiza_uralensis_Fisch0114200.1	2.277749413	3.998104085	0.569707382	0.568876182	NA
Glycyrrhiza_uralensis_Fisch0028260.1	2.277749413	3.998104085	0.569707382	0.568876182	NA
Glycyrrhiza_uralensis_Fisch0114870.1	2.277749413	3.998104085	0.569707382	0.568876182	NA
Glycyrrhiza_uralensis_Fisch0157420.1	2.277749413	3.998104085	0.569707382	0.568876182	NA
Glycyrrhiza_uralensis_Fisch0036720.1	2.277749413	3.998104085	0.569707382	0.568876182	NA
Glycyrrhiza_uralensis_Fisch0	2.277749413	3.998104	0.5697073	0.568876	NA

150590.1		085	82	182	
Glycyrrhiza_uralensis_Fisch0271430.1	2.277749413	3.998104085	0.569707382	0.568876182	NA
Glycyrrhiza_uralensis_Fisch0176840.1	2.277749413	3.998104085	0.569707382	0.568876182	NA
Glycyrrhiza_uralensis_Fisch0182910.1	2.277749413	3.998104085	0.569707382	0.568876182	NA
Glycyrrhiza_uralensis_Fisch0181000.1	2.277749413	3.998104085	0.569707382	0.568876182	NA
Glycyrrhiza_uralensis_Fisch0229580.1	2.277749413	3.998104085	0.569707382	0.568876182	NA
Glycyrrhiza_uralensis_Fisch0034450.1	2.277749413	3.998104085	0.569707382	0.568876182	NA
Glycyrrhiza_uralensis_Fisch0108930.1	2.277749413	3.998104085	0.569707382	0.568876182	NA
Glycyrrhiza_uralensis_Fisch0151220.1	2.277749413	3.998104085	0.569707382	0.568876182	NA
Glycyrrhiza_uralensis_Fisch0232600.1	2.277749413	3.998104085	0.569707382	0.568876182	NA
Glycyrrhiza_uralensis_Fisch0183050.1	2.277749413	3.998104085	0.569707382	0.568876182	NA
Glycyrrhiza_uralensis_Fisch0110460.1	2.277749413	3.998104085	0.569707382	0.568876182	NA
Glycyrrhiza_uralensis_Fisch0213660.1	2.277749413	3.998104085	0.569707382	0.568876182	NA
Glycyrrhiza_uralensis_Fisch0026030.1	2.27064227	2.260822117	1.00434362	0.315213015	0.882780387
Glycyrrhiza_uralensis_Fisch0043240.1	2.265334654	1.563061254	1.449293589	0.147255611	0.747275789
Glycyrrhiza_uralensis_Fisch0279960.1	2.264577982	0.610633698	3.708570274	0.000208433	0.022497659
Glycyrrhiza_uralensis_Fisch0158080.1	2.264230599	0.606840094	3.731181608	0.000190584	0.021139868
Glycyrrhiza_uralensis_Fisch0066450.1	2.263824891	1.92296026	1.177260361	0.239091603	0.834895529
Glycyrrhiza_uralensis_Fisch0166550.1	2.262498473	0.650359652	3.478842	0.000503585	0.042135102
Glycyrrhiza_uralensis_Fisch0224020.1	2.257070979	1.046866769	2.156025051	0.031081709	0.431053322
Glycyrrhiza_uralensis_Fisch0072900.1	2.255945011	3.832255991	0.588672838	0.556080762	NA
Glycyrrhiza_uralensis_Fisch0163620.1	2.253225321	0.379022958	5.944825442	2.77E-09	2.83E-06
Glycyrrhiza_uralensis_Fisch0	2.251242529	0.659505	3.4135295	0.000641	0.049743

209860.1		791	87	272	04
Glycyrrhiza_uralensis_Fisch0171090.1	2.249606109	1.053104635	2.136165803	0.032665902	0.442207404
Glycyrrhiza_uralensis_Fisch0031070.1	2.248378044	0.819976155	2.742004178	0.006106556	0.194366451
Glycyrrhiza_uralensis_Fisch0001920.1	2.245426873	1.212964472	1.851189317	0.064142322	0.58182822
Glycyrrhiza_uralensis_Fisch0057630.1	2.245236731	1.406035261	1.596856632	0.110297669	0.697894385
Glycyrrhiza_uralensis_Fisch0150600.1	2.245046613	2.913283053	0.770624266	0.440929674	0.919447046
Glycyrrhiza_uralensis_Fisch0018500.1	2.243683209	2.135244689	1.050785055	0.293357321	0.868229887
Glycyrrhiza_uralensis_Fisch0276820.1	2.237301383	1.442306982	1.55119639	0.120854627	0.712110373
Glycyrrhiza_uralensis_Fisch0020200.1	2.237274147	0.376691808	5.939269453	2.86E-09	2.83E-06
Glycyrrhiza_uralensis_Fisch0216740.1	2.232005459	1.307321341	1.707312035	0.08776407	0.649994208
Glycyrrhiza_uralensis_Fisch0275540.1	2.231547716	1.166882354	1.912401631	0.055824699	0.553093989
Glycyrrhiza_uralensis_Fisch0188590.1	2.228929164	2.945444511	0.756737788	0.449206971	0.92262842
Glycyrrhiza_uralensis_Fisch0026470.1	2.226615851	3.807979949	0.584723628	0.558733564	0.949902309
Glycyrrhiza_uralensis_Fisch0043580.1	2.226203888	2.424696286	0.918137212	0.358547035	NA
Glycyrrhiza_uralensis_Fisch0157580.1	2.223872773	2.959892054	0.751335769	0.452450608	0.922710496
Glycyrrhiza_uralensis_Fisch0027470.1	2.221766955	0.921729161	2.410433616	0.015933571	0.313410208
Glycyrrhiza_uralensis_Fisch0284570.1	2.218746944	0.641111082	3.460783954	0.000538605	0.044246648
Glycyrrhiza_uralensis_Fisch0284080.1	2.216607966	0.646859709	3.426721334	0.000610916	0.048458834
Glycyrrhiza_uralensis_Fisch0276390.1	2.214556335	2.922640371	0.757724541	0.448615904	0.922606358
Glycyrrhiza_uralensis_Fisch0079600.1	2.214396106	0.555618577	3.985460883	6.73E-05	0.00959229
Glycyrrhiza_uralensis_Fisch0174490.1	2.210031706	3.408690637	0.648352092	0.516757249	NA
Glycyrrhiza_uralensis_Fisch0282420.1	2.209875695	0.559719211	3.948186254	7.87E-05	0.010708502
Glycyrrhiza_uralensis_Fisch0	2.206480313	2.032240	1.0857380	0.277594	0.859826

147020.1		047	34	908	609
Glycyrrhiza_uralensis_Fisch0 175930.1	2.204374065	1.360523 6	1.6202394 91	0.105180 841	0.692097 679
Glycyrrhiza_uralensis_Fisch0 266990.1	2.202728623	1.221947 549	1.8026376 21	0.071445 145	0.604224 35
Glycyrrhiza_uralensis_Fisch0 118460.1	2.198237237	0.742538 71	2.9604345 28	0.003072 054	0.135277 149
Glycyrrhiza_uralensis_Fisch0 038490.1	2.193900187	2.947684 445	0.7442791 89	0.456707 589	0.924102 621
Glycyrrhiza_uralensis_Fisch0 088930.1	2.188494487	1.269386 604	1.7240567 07	0.084697 607	0.643723 846
Glycyrrhiza_uralensis_Fisch0 068550.1	2.187980737	1.237505 098	1.7680579 59	0.077051 215	0.623757 367
Glycyrrhiza_uralensis_Fisch0 166230.1	2.182846128	0.472574 811	4.6190488 31	3.86E-06	0.001066 559
Glycyrrhiza_uralensis_Fisch0 097960.1	2.181889141	1.878488 058	1.1615134 48	0.245433 158	0.840577 135
Glycyrrhiza_uralensis_Fisch0 014400.1	2.176458157	2.714757 12	0.8017137 67	0.422718 55	0.913852 659
Glycyrrhiza_uralensis_Fisch0 018080.1	2.175848059	1.820435 761	1.1952347 38	0.231995 33	0.828477 62
Glycyrrhiza_uralensis_Fisch0 079960.1	2.171275066	1.196553 989	1.8146068 51	0.069584 349	0.600603 436
Glycyrrhiza_uralensis_Fisch0 243150.1	2.170760509	0.491848 21	4.4134764 84	1.02E-05	0.002205 848
Glycyrrhiza_uralensis_Fisch0 152970.1	2.168756502	0.561314 098	3.8637128 61	0.000111 676	0.014147 648
Glycyrrhiza_uralensis_Fisch0 007280.1	2.168401104	0.634345 812	3.4183265 09	0.000630 075	0.049561 76
Glycyrrhiza_uralensis_Fisch0 260440.1	2.167505632	3.892800 523	0.5567985 36	0.577665 084	NA
Glycyrrhiza_uralensis_Fisch0 205180.1	2.163887282	1.721077 686	1.2572862 34	0.208650 007	0.811966 832
Glycyrrhiza_uralensis_Fisch0 254360.1	2.163101188	0.647376 727	3.3413329 47	0.000833 772	0.059227 469
Glycyrrhiza_uralensis_Fisch0 064770.1	2.162044167	3.806550 083	0.5679799 61	0.570048 575	0.952518 696
Glycyrrhiza_uralensis_Fisch0 040960.1	2.161103208	2.240334 075	0.9646343 52	0.334727 987	0.888988 497
Glycyrrhiza_uralensis_Fisch0 240650.1	2.156724887	2.066765 448	1.0435266 8	0.296704 431	0.869761 143
Glycyrrhiza_uralensis_Fisch0 008490.1	2.156598472	0.560500 238	3.8476316 79	0.000119 265	0.014948 504
Glycyrrhiza_uralensis_Fisch0	2.152060679	0.799102	2.6930955	0.007079	0.209854

246960.1		993	05	199	834
Glycyrrhiza_uralensis_Fisch0204910.1	2.151018433	0.773352518	2.781420354	0.005412161	0.183738664
Glycyrrhiza_uralensis_Fisch0203540.1	2.149708783	0.493059474	4.359938093	1.30E-05	0.002653804
Glycyrrhiza_uralensis_Fisch0268750.1	2.149045045	0.375635031	5.721098594	1.06E-08	8.49E-06
Glycyrrhiza_uralensis_Fisch0038190.1	2.143490992	2.153289288	0.995449615	0.319517638	0.885321694
Glycyrrhiza_uralensis_Fisch0057130.1	2.143219645	1.2342463	1.736460255	0.082482486	0.639269575
Glycyrrhiza_uralensis_Fisch0152410.1	2.139348449	1.330271855	1.608203949	0.107790519	0.692784222
Glycyrrhiza_uralensis_Fisch0044240.1	2.136255618	0.726249964	2.941488087	0.003266394	0.138175932
Glycyrrhiza_uralensis_Fisch0154200.1	2.133372641	0.983767181	2.168574722	0.030114986	0.426204537
Glycyrrhiza_uralensis_Fisch0054740.1	2.130303732	1.104612586	1.928552834	0.053786399	0.546162726
Glycyrrhiza_uralensis_Fisch0019440.1	2.130221349	0.554526835	3.841511747	0.000122279	0.015171406
Glycyrrhiza_uralensis_Fisch0208460.1	2.120924551	1.29366209	1.639473373	0.101114712	0.682893128
Glycyrrhiza_uralensis_Fisch0082100.1	2.115070494	0.772502973	2.737944795	0.006182445	0.19580455
Glycyrrhiza_uralensis_Fisch0084740.1	2.11447742	3.257478941	0.649114686	0.51626425	NA
Glycyrrhiza_uralensis_Fisch0051530.1	2.11241436	0.512173195	4.124414129	3.72E-05	0.006085938
Glycyrrhiza_uralensis_Fisch0012370.1	2.112310726	1.327216346	1.591534592	0.111489317	0.698786349
Glycyrrhiza_uralensis_Fisch0121480.1	2.102399587	0.845692255	2.486010217	0.012918426	0.281145123
Glycyrrhiza_uralensis_Fisch0141220.1	2.100852494	0.501409439	4.189894189	2.79E-05	0.004975974
Glycyrrhiza_uralensis_Fisch0222700.1	2.099040426	0.953109905	2.202306801	0.027643644	0.407959849
Glycyrrhiza_uralensis_Fisch0064870.1	2.096085477	1.583941071	1.323335518	0.185723825	0.794168142
Glycyrrhiza_uralensis_Fisch0136150.1	2.093819771	1.28319114	1.631728669	0.102736658	0.687864076
Glycyrrhiza_uralensis_Fisch0047090.1	2.092300746	1.145925324	1.825861338	0.067871151	0.594613222
Glycyrrhiza_uralensis_Fisch0	2.091469824	2.266960	0.9225878	0.356222	0.897807

054280.1		049	62	024	124
Glycyrrhiza_uralensis_Fisch0184590.1	2.087094116	0.948653672	2.200059072	0.027802704	0.409303419
Glycyrrhiza_uralensis_Fisch0027140.1	2.076983305	0.938486219	2.21312073	0.026889323	0.40150497
Glycyrrhiza_uralensis_Fisch0275720.1	2.071426927	1.33454584	1.552158693	0.120624257	0.711449612
Glycyrrhiza_uralensis_Fisch0150440.1	2.069428239	3.184758964	0.649791165	0.515827126	NA
Glycyrrhiza_uralensis_Fisch0215610.1	2.068323803	1.826445946	1.132430887	0.257453325	0.84740043
Glycyrrhiza_uralensis_Fisch0153210.1	2.063225177	1.158562124	1.780849844	0.074936984	0.618589034
Glycyrrhiza_uralensis_Fisch0142730.1	2.062147129	0.721140546	2.859563423	0.004242246	0.159994804
Glycyrrhiza_uralensis_Fisch0123420.1	2.060470559	0.382875768	5.381564293	7.38E-08	4.56E-05
Glycyrrhiza_uralensis_Fisch0140060.1	2.05997484	0.253537869	8.124919756	4.48E-16	1.54E-12
Glycyrrhiza_uralensis_Fisch0214050.1	2.054621578	0.678071721	3.030094774	0.00244477	0.116986155
Glycyrrhiza_uralensis_Fisch0060660.1	2.054594912	0.943705258	2.177157426	0.029468824	0.422462527
Glycyrrhiza_uralensis_Fisch0040480.1	2.052626326	2.531776006	0.810745628	0.417511765	0.913280981
Glycyrrhiza_uralensis_Fisch0068920.1	2.051737848	0.461800046	4.442913911	8.87E-06	0.002015265
Glycyrrhiza_uralensis_Fisch0177520.1	2.05163273	1.983671863	1.034260136	0.301014568	0.872983114
Glycyrrhiza_uralensis_Fisch0180830.1	2.050726153	3.961905947	0.517611014	0.604729696	NA
Glycyrrhiza_uralensis_Fisch0257410.1	2.046935954	0.50229492	4.075167538	4.60E-05	0.007049479
Glycyrrhiza_uralensis_Fisch0211570.1	2.039491233	2.775213888	0.734895152	0.462403351	0.924964086
Glycyrrhiza_uralensis_Fisch0092950.1	2.029148744	0.512388864	3.960173389	7.49E-05	0.010420413
Glycyrrhiza_uralensis_Fisch0038270.1	2.024639386	0.442643336	4.573974624	4.79E-06	0.001252053
Glycyrrhiza_uralensis_Fisch0201780.1	2.021409152	0.597772338	3.381570248	0.000720728	0.053708741
Glycyrrhiza_uralensis_Fisch0258740.1	2.021298758	0.581296068	3.477227647	0.000506627	0.042135102
Glycyrrhiza_uralensis_Fisch0	2.019648823	0.613259	3.2933045	0.000990	0.066020

142780.1		054	35	172	585
Glycyrrhiza_uralensis_Fisch0086440.1	2.019038737	1.446278362	1.396023607	0.162707386	0.768517816
Glycyrrhiza_uralensis_Fisch0109460.1	2.016793075	0.989710901	2.037759787	0.041573962	0.49475144
Glycyrrhiza_uralensis_Fisch0259510.1	2.015269478	0.404250657	4.985197775	6.19E-07	0.000252525
Glycyrrhiza_uralensis_Fisch0120580.1	2.010078229	0.630544147	3.187846937	0.001433364	0.084769211
Glycyrrhiza_uralensis_Fisch0070250.1	2.009814592	0.446174796	4.504545324	6.65E-06	0.001633695
Glycyrrhiza_uralensis_Fisch0123510.1	2.008696672	1.349895353	1.488038808	0.136740659	0.733728967
Glycyrrhiza_uralensis_Fisch0091660.1	2.007053652	2.026208935	0.990546245	0.321907197	0.886479038
Glycyrrhiza_uralensis_Fisch0281710.1	2.005053325	0.687445426	2.916672726	0.003537868	0.146276169
Glycyrrhiza_uralensis_Fisch0249720.1	1.999466396	0.510745648	3.914798696	9.05E-05	0.011966184
Glycyrrhiza_uralensis_Fisch0144690.1	1.998614155	1.298060574	1.539692519	0.123635321	0.717084863
Glycyrrhiza_uralensis_Fisch0054780.1	1.998480554	1.519222387	1.315462812	0.188354408	0.797474632
Glycyrrhiza_uralensis_Fisch0026790.1	1.99807022	0.940565918	2.124327687	0.033642752	0.449486331
Glycyrrhiza_uralensis_Fisch0243480.1	1.997878451	2.067931166	0.966124252	0.33398201	0.888735657
Glycyrrhiza_uralensis_Fisch0163110.1	1.996685474	1.992429296	1.002136175	0.316277828	0.883160394
Glycyrrhiza_uralensis_Fisch0221630.1	1.991877113	2.292705571	0.868788884	0.384962614	0.906957344
Glycyrrhiza_uralensis_Fisch0160860.1	1.991604707	1.015382638	1.961432698	0.049828569	0.529504363
Glycyrrhiza_uralensis_Fisch0014350.1	1.987052223	0.610083166	3.257018607	0.001125891	0.07226716
Glycyrrhiza_uralensis_Fisch0092330.1	1.986530636	0.957653803	2.074372419	0.038044737	0.476946258
Glycyrrhiza_uralensis_Fisch0209250.1	1.985629208	1.645409856	1.206768758	0.227521211	0.82488862
Glycyrrhiza_uralensis_Fisch0098270.1	1.9851138	1.735060628	1.144117829	0.252574774	0.845775428
Glycyrrhiza_uralensis_Fisch0124630.1	1.984801966	0.417429971	4.754814231	1.99E-06	0.000629079
Glycyrrhiza_uralensis_Fisch0	1.983140701	1.559584	1.2715825	0.203521	0.808522

049970.1		76	08	495	144
Glycyrrhiza_uralensis_Fisch0082890.1	1.977678435	2.081839939	0.949966612	0.342129218	0.894158318
Glycyrrhiza_uralensis_Fisch0039250.1	1.975169105	0.66634922	2.964165102	0.003035054	0.135034658
Glycyrrhiza_uralensis_Fisch0282500.1	1.970418151	0.48499282	4.062777979	4.85E-05	0.007340918
Glycyrrhiza_uralensis_Fisch0156470.1	1.970268337	0.419095308	4.7012417	2.59E-06	0.000787864
Glycyrrhiza_uralensis_Fisch0055910.1	1.969920058	2.009382618	0.980360853	0.326908026	0.887225647
Glycyrrhiza_uralensis_Fisch0067220.1	1.968422188	0.333493912	5.902423155	3.58E-09	3.32E-06
Glycyrrhiza_uralensis_Fisch0077850.1	1.967996973	1.773682067	1.10955453	0.267191034	0.854585363
Glycyrrhiza_uralensis_Fisch0154440.1	1.967544555	0.884575136	2.224282003	0.026129477	0.395308929
Glycyrrhiza_uralensis_Fisch0172000.1	1.964878154	1.883753502	1.043065429	0.296917991	0.869761143
Glycyrrhiza_uralensis_Fisch0194780.1	1.96395189	2.037315305	0.963990151	0.335050864	0.889039773
Glycyrrhiza_uralensis_Fisch0054800.1	1.957401137	0.65748771	2.977091599	0.002909971	0.13122759
Glycyrrhiza_uralensis_Fisch0073480.1	1.954949829	1.292862612	1.512109493	0.13050601	0.725270532
Glycyrrhiza_uralensis_Fisch0174030.1	1.95459783	0.43145473	4.53025009	5.89E-06	0.001477143
Glycyrrhiza_uralensis_Fisch0257800.1	1.953336929	1.465051134	1.333289251	0.1824369	0.791863958
Glycyrrhiza_uralensis_Fisch0025380.1	1.95330017	0.365740377	5.340674124	9.26E-08	5.41E-05
Glycyrrhiza_uralensis_Fisch0209070.1	1.952093025	0.515753333	3.784935354	0.000153749	0.017964696
Glycyrrhiza_uralensis_Fisch0121680.1	1.94768976	1.48499569	1.311579403	0.189662086	0.797474632
Glycyrrhiza_uralensis_Fisch0210160.1	1.947678499	1.03120494	1.888740466	0.058926614	0.564515005
Glycyrrhiza_uralensis_Fisch0095560.1	1.945249624	0.354080847	5.493800746	3.93E-08	2.78E-05
Glycyrrhiza_uralensis_Fisch0261010.1	1.943804222	0.688406025	2.823630461	0.00474831	0.170943755
Glycyrrhiza_uralensis_Fisch0053510.1	1.942126505	3.026440907	0.641719619	0.521055248	0.944144726
Glycyrrhiza_uralensis_Fisch0	1.941292165	0.676765	2.8684841	0.004124	0.157330

153330.1		867	53	439	011
Glycyrrhiza_uralensis_Fisch0041290.1	1.94024755	0.973639518	1.992778142	0.046285746	0.515126062
Glycyrrhiza_uralensis_Fisch0114510.1	1.936840021	0.805090469	2.40574208	0.016139655	0.314814824
Glycyrrhiza_uralensis_Fisch0150160.1	1.928850061	2.04726352	0.942160128	0.346110664	0.895255665
Glycyrrhiza_uralensis_Fisch0052170.1	1.922754411	2.112757997	0.910068457	0.362786408	0.899306781
Glycyrrhiza_uralensis_Fisch0222180.1	1.922293849	0.850051032	2.261386407	0.02373534	0.376109038
Glycyrrhiza_uralensis_Fisch0150150.1	1.919852686	2.519568712	0.761976713	0.446073903	0.920840013
Glycyrrhiza_uralensis_Fisch0100990.1	1.919434941	1.510885584	1.270403901	0.203940794	0.808522144
Glycyrrhiza_uralensis_Fisch0246260.1	1.919370705	1.192363628	1.609719267	0.107459157	0.692329204
Glycyrrhiza_uralensis_Fisch0100310.1	1.91932347	0.774498924	2.478148658	0.013206611	0.283571027
Glycyrrhiza_uralensis_Fisch0259670.1	1.917811996	0.890856331	2.152773605	0.031336476	0.433660376
Glycyrrhiza_uralensis_Fisch0072300.1	1.917615747	0.564956604	3.394270876	0.000688116	0.051759221
Glycyrrhiza_uralensis_Fisch0171160.1	1.915921743	0.57919976	3.307877306	0.00094006	0.064281934
Glycyrrhiza_uralensis_Fisch0094280.1	1.915733731	0.731920698	2.617406143	0.008860085	0.233840184
Glycyrrhiza_uralensis_Fisch0254440.1	1.913739691	0.739760786	2.586970989	0.009682374	0.242512749
Glycyrrhiza_uralensis_Fisch0271000.1	1.91021265	1.280648863	1.491597506	0.135804692	0.731522758
Glycyrrhiza_uralensis_Fisch0258500.1	1.908723458	0.320989722	5.946369402	2.74E-09	2.83E-06
Glycyrrhiza_uralensis_Fisch0115900.1	1.908436339	0.739403813	2.581047467	0.009850104	0.244676983
Glycyrrhiza_uralensis_Fisch0184240.1	1.90839454	0.443778307	4.300333092	1.71E-05	0.003284255
Glycyrrhiza_uralensis_Fisch0070280.1	1.905397015	0.465945359	4.089314288	4.33E-05	0.006762268
Glycyrrhiza_uralensis_Fisch0257920.1	1.90475285	2.00285575	0.951018489	0.34159499	0.894105199
Glycyrrhiza_uralensis_Fisch0028410.1	1.904161633	1.092711518	1.742602327	0.081403103	0.636559454
Glycyrrhiza_uralensis_Fisch0	1.903436715	2.079699	0.9152459	0.360062	0.898154

155570.1		654	64	52	454
Glycyrrhiza_uralensis_Fisch0 110520.1	1.902983156	0.359408 17	5.2947687 72	1.19E-07	6.10E-05
Glycyrrhiza_uralensis_Fisch0 138650.1	1.902832386	1.237105 396	1.5381327 99	0.124016 148	0.718026 594
Glycyrrhiza_uralensis_Fisch0 183850.1	1.899529508	3.841646 32	0.4944571 55	0.620983 358	NA
Glycyrrhiza_uralensis_Fisch0 261030.1	1.89885099	1.522925 927	1.2468439 58	0.212454 719	0.813575 823
Glycyrrhiza_uralensis_Fisch0 040830.1	1.897527065	1.027129 26	1.8474082 47	0.064687 995	0.583161 062
Glycyrrhiza_uralensis_Fisch0 211380.1	1.892224353	3.043698 74	0.6216858 22	0.534148 476	0.945943 27
Glycyrrhiza_uralensis_Fisch0 038310.1	1.885461847	1.501929 985	1.2553593 49	0.209348 339	0.811966 832
Glycyrrhiza_uralensis_Fisch0 127230.1	1.88277239	0.702356 487	2.6806506 74	0.007347 918	0.215163 477
Glycyrrhiza_uralensis_Fisch0 135710.1	1.882383484	1.218321 789	1.5450626 43	0.122331 112	0.714482 657
Glycyrrhiza_uralensis_Fisch0 029340.1	1.881716612	2.343042 573	0.8031081 61	0.421912 221	0.913755 795
Glycyrrhiza_uralensis_Fisch0 096280.1	1.881190516	0.394894 678	4.7637778 38	1.90E-06	0.000623 275
Glycyrrhiza_uralensis_Fisch0 166720.1	1.880808706	0.727848 897	2.5840647 88	0.009764 345	0.243299 985
Glycyrrhiza_uralensis_Fisch0 248360.1	1.878783818	0.574516 959	3.2701973 18	0.001074 725	0.070104 683
Glycyrrhiza_uralensis_Fisch0 040980.1	1.876510251	3.896495 946	0.4815891 71	0.630097 821	NA
Glycyrrhiza_uralensis_Fisch0 259430.1	1.876425932	3.811712 506	0.4922789 77	0.622522 14	0.962923 206
Glycyrrhiza_uralensis_Fisch0 089420.1	1.876082898	0.848593 043	2.2108157 89	0.027048 596	0.402634 327
Glycyrrhiza_uralensis_Fisch0 180920.1	1.867958703	1.879800 921	0.9937002 81	0.320368 806	NA
Glycyrrhiza_uralensis_Fisch0 212480.1	1.865575012	2.919168 345	0.6390775 7	0.522772 471	0.944537 86
Glycyrrhiza_uralensis_Fisch0 212470.1	1.861909553	0.556658 579	3.3447962 95	0.000823 43	0.058987 994
Glycyrrhiza_uralensis_Fisch0 191780.1	1.860690629	0.844800 856	2.2025198 18	0.027628 611	0.407959 849
Glycyrrhiza_uralensis_Fisch0 174530.1	1.858204682	3.813351 516	0.4872891 14	0.626053 461	NA
Glycyrrhiza_uralensis_Fisch0	1.858204682	3.813351	0.4872891	0.626053	NA

106170.1		516	14	461	
Glycyrrhiza_uralensis_Fisch0 171110.1	1.856175684	0.996170 248	1.8633117 06	0.062418 424	0.575020 79
Glycyrrhiza_uralensis_Fisch0 238120.1	1.851392164	2.009437 089	0.9213486 57	0.356868 425	0.897807 124
Glycyrrhiza_uralensis_Fisch0 203760.1	1.845675025	3.072777 321	0.6006536 86	0.548070 672	0.948727 873
Glycyrrhiza_uralensis_Fisch0 138170.1	1.840197671	3.810014 498	0.4829896 77	0.629103 066	0.963563 011
Glycyrrhiza_uralensis_Fisch0 140500.1	1.837303642	0.374272 671	4.9089976	9.15E-07	0.000349 753
Glycyrrhiza_uralensis_Fisch0 009670.1	1.833057187	0.620434 366	2.9544739 75	0.003132 026	0.135346 245
Glycyrrhiza_uralensis_Fisch0 197880.1	1.832260009	1.082786 615	1.6921709 08	0.090613 392	0.656551 577
Glycyrrhiza_uralensis_Fisch0 064420.1	1.831753462	1.169581 286	1.5661617 4	0.117310 757	0.706303 999
Glycyrrhiza_uralensis_Fisch0 034070.1	1.831439094	0.936700 407	1.9552026 24	NA	NA
Glycyrrhiza_uralensis_Fisch0 055860.1	1.831303496	0.680537 578	2.6909660 18	0.007124 545	0.210672 975
Glycyrrhiza_uralensis_Fisch0 169280.1	1.829604196	0.533352 215	3.4303864 19	0.000602 722	0.048197 756
Glycyrrhiza_uralensis_Fisch0 039110.1	1.825901482	1.353259 488	1.3492619 11	0.177252 855	0.788311 562
Glycyrrhiza_uralensis_Fisch0 020920.1	1.825445072	2.558603 704	0.7134536 19	0.475565 104	0.929731 323
Glycyrrhiza_uralensis_Fisch0 105560.1	1.823936095	1.639041 451	1.1128065 7	0.265791 513	0.854047 617
Glycyrrhiza_uralensis_Fisch0 221540.1	1.823865798	1.232713 647	1.4795535 05	0.138992 442	0.737015 71
Glycyrrhiza_uralensis_Fisch0 051080.1	1.823240056	2.914411 357	0.6255946 17	0.531580 871	0.945897 21
Glycyrrhiza_uralensis_Fisch0 112380.1	1.821955209	3.102884 156	0.5871811 89	0.557082 024	0.949576 115
Glycyrrhiza_uralensis_Fisch0 011020.1	1.821457413	4.031550 674	0.4518006 99	0.651412 566	NA
Glycyrrhiza_uralensis_Fisch0 257910.1	1.821457413	4.031550 674	0.4518006 99	0.651412 566	NA
Glycyrrhiza_uralensis_Fisch0 174130.1	1.821457413	4.031550 674	0.4518006 99	0.651412 566	NA
Glycyrrhiza_uralensis_Fisch0 170940.1	1.821457413	4.031550 674	0.4518006 99	0.651412 566	NA
Glycyrrhiza_uralensis_Fisch0	1.821457413	4.031550	0.4518006	0.651412	NA

041380.1		674	99	566	
Glycyrrhiza_uralensis_Fisch0 015510.1	1.821457413	4.031550 674	0.4518006 99	0.651412 566	NA
Glycyrrhiza_uralensis_Fisch0 105840.1	1.821457413	4.031550 674	0.4518006 99	0.651412 566	NA
Glycyrrhiza_uralensis_Fisch0 174760.1	1.821457413	4.031550 674	0.4518006 99	0.651412 566	NA
Glycyrrhiza_uralensis_Fisch0 070070.1	1.821457413	4.031550 674	0.4518006 99	0.651412 566	NA
Glycyrrhiza_uralensis_Fisch0 246510.1	1.821457413	4.031550 674	0.4518006 99	0.651412 566	NA
Glycyrrhiza_uralensis_Fisch0 233730.1	1.821457413	4.031550 674	0.4518006 99	0.651412 566	NA
Glycyrrhiza_uralensis_Fisch0 141070.1	1.821457413	4.031550 674	0.4518006 99	0.651412 566	NA
Glycyrrhiza_uralensis_Fisch0 052930.1	1.821457413	4.031550 674	0.4518006 99	0.651412 566	NA
Glycyrrhiza_uralensis_Fisch0 231200.1	1.821457413	4.031550 674	0.4518006 99	0.651412 566	NA
Glycyrrhiza_uralensis_Fisch0 279920.1	1.821457413	4.031550 674	0.4518006 99	0.651412 566	NA
Glycyrrhiza_uralensis_Fisch0 221640.1	1.821457413	4.031550 674	0.4518006 99	0.651412 566	NA
Glycyrrhiza_uralensis_Fisch0 014390.1	1.81439276	2.584434 565	0.7020463 14	0.482650 281	NA
Glycyrrhiza_uralensis_Fisch0 189440.1	1.811832772	2.283444 588	0.7934647 43	0.427507 103	0.916400 488
Glycyrrhiza_uralensis_Fisch0 034750.1	1.811119832	1.758652 892	1.0298335 96	0.303088 127	0.873493 229
Glycyrrhiza_uralensis_Fisch0 208230.1	1.811109599	0.663957 388	2.7277497 48	0.006376 797	0.199596 218
Glycyrrhiza_uralensis_Fisch0 009310.1	1.809943026	1.160361 162	1.5598100 7	0.118804 771	0.710114 437
Glycyrrhiza_uralensis_Fisch0 173660.1	1.809942004	3.303924 216	0.5478158 35	0.583818 364	NA
Glycyrrhiza_uralensis_Fisch0 261660.1	1.80738782	1.652242 168	1.0939000 68	0.273998 809	0.858000 161
Glycyrrhiza_uralensis_Fisch0 054440.1	1.806544647	1.507159 155	1.1986422 55	0.230667 081	0.828030 507
Glycyrrhiza_uralensis_Fisch0 153730.1	1.801419601	1.211572 415	1.4868443 51	0.137055 925	0.734402 518
Glycyrrhiza_uralensis_Fisch0 178960.1	1.800527999	2.698314 581	0.6672787 57	0.504594 093	0.939945 395
Glycyrrhiza_uralensis_Fisch0	1.800199231	0.747426	2.4085316	0.016016	0.313435

257660.1		018	64	837	173
Glycyrrhiza_uralensis_Fisch0081830.1	1.797589457	1.36552636	1.316407731	0.188037229	0.797474632
Glycyrrhiza_uralensis_Fisch0157160.1	1.79746054	0.574556915	3.128429045	0.001757434	0.096799646
Glycyrrhiza_uralensis_Fisch0178310.1	1.797109493	1.369134915	1.312587586	0.189321955	0.797474632
Glycyrrhiza_uralensis_Fisch0154960.1	1.791390912	1.736338621	1.031705965	0.302209882	0.87347594
Glycyrrhiza_uralensis_Fisch0108540.1	1.790513374	0.632348011	2.831531597	0.004632566	0.168948264
Glycyrrhiza_uralensis_Fisch0004310.1	1.789965754	0.462815542	3.867557575	0.000109931	0.014000192
Glycyrrhiza_uralensis_Fisch0097350.1	1.786177529	0.407459379	4.383694724	1.17E-05	0.002463654
Glycyrrhiza_uralensis_Fisch0107960.1	1.785945053	1.888182379	0.945854104	0.344223005	0.894405752
Glycyrrhiza_uralensis_Fisch0246610.1	1.783780931	1.737528447	1.026619699	0.304599574	0.875114795
Glycyrrhiza_uralensis_Fisch0277450.1	1.783616648	1.871079034	0.953255643	0.34046056	0.893467693
Glycyrrhiza_uralensis_Fisch0216540.1	1.781795903	0.328952601	5.416573391	6.08E-08	3.85E-05
Glycyrrhiza_uralensis_Fisch0128820.1	1.780712459	1.398722474	1.273099197	0.202982844	0.808104045
Glycyrrhiza_uralensis_Fisch0074780.1	1.780545976	1.022811186	1.740835454	0.081712425	0.637335735
Glycyrrhiza_uralensis_Fisch0120730.1	1.78032779	0.562745764	3.163644942	0.001558067	0.089934495
Glycyrrhiza_uralensis_Fisch0271090.1	1.778810489	0.674706128	2.636422606	0.008378531	0.23021017
Glycyrrhiza_uralensis_Fisch0100250.1	1.77843536	0.968209148	1.836829743	0.066235034	0.589381612
Glycyrrhiza_uralensis_Fisch0050480.1	1.777388845	4.035358917	0.44045372	0.659608525	NA
Glycyrrhiza_uralensis_Fisch0002800.1	1.777075507	1.527612531	1.16330252	0.244706784	0.839393558
Glycyrrhiza_uralensis_Fisch0075470.1	1.776292346	1.316029253	1.349736217	0.177100611	0.787950406
Glycyrrhiza_uralensis_Fisch0157860.1	1.773451104	1.080705298	1.641012685	0.100794782	0.681807835
Glycyrrhiza_uralensis_Fisch0234840.1	1.773420154	2.256303073	0.785984904	0.431876331	0.916951181
Glycyrrhiza_uralensis_Fisch0	1.770983703	0.574086	3.0848699	0.002036	0.105784

261530.1		998	04	412	917
Glycyrrhiza_uralensis_Fisch0 237020.1	1.769956514	0.428701 51	4.1286453 96	3.65E-05	0.006015 961
Glycyrrhiza_uralensis_Fisch0 027220.1	1.769096877	1.416464 428	1.2489525 62	0.211682 425	0.813314 506
Glycyrrhiza_uralensis_Fisch0 232420.1	1.768427559	0.840125 606	2.1049561 47	0.035295 126	0.456503 854
Glycyrrhiza_uralensis_Fisch0 078830.1	1.760218488	0.428539 919	4.1074784 63	4.00E-05	0.006376 192
Glycyrrhiza_uralensis_Fisch0 181290.1	1.759686415	0.771672 882	2.2803527 97	0.022586 773	0.366993 26
Glycyrrhiza_uralensis_Fisch0 098090.1	1.758656971	1.271954 024	1.3826419 33	0.166774 685	0.773428 812
Glycyrrhiza_uralensis_Fisch0 012480.1	1.757986	0.685337 364	2.5651395 84	0.010313 431	0.251259 387
Glycyrrhiza_uralensis_Fisch0 262270.1	1.755670678	0.972235 303	1.8058084 01	0.070948 273	0.603649 673
Glycyrrhiza_uralensis_Fisch0 173510.1	1.755163193	0.638863 62	2.7473206 13	0.006008 437	0.194366 451
Glycyrrhiza_uralensis_Fisch0 176920.1	1.753128434	1.449085 794	1.2098168 66	0.226349 173	0.823953 464
Glycyrrhiza_uralensis_Fisch0 000680.1	1.752699158	2.033470 435	0.8619250 75	0.388728 749	0.908584 712
Glycyrrhiza_uralensis_Fisch0 235520.1	1.752413732	0.462654 675	3.7877359 19	0.000152 026	0.017859 218
Glycyrrhiza_uralensis_Fisch0 026350.1	1.751883676	0.725395 62	2.4150734 12	0.015732 039	0.312434 131
Glycyrrhiza_uralensis_Fisch0 285820.1	1.750967173	3.842266 799	0.4557120 22	0.648597 076	NA
Glycyrrhiza_uralensis_Fisch0 275990.1	1.750775083	1.189924 693	1.4713326 76	0.141201 171	0.741661 163
Glycyrrhiza_uralensis_Fisch0 014580.1	1.743949426	2.752067 062	0.6336871 11	0.526285 038	0.944927 971
Glycyrrhiza_uralensis_Fisch0 195000.1	1.741056782	0.747798 695	2.3282426 06	0.019899 225	0.345331 179
Glycyrrhiza_uralensis_Fisch0 030690.1	1.740836457	3.298843 395	0.5277111 55	0.597699 83	NA
Glycyrrhiza_uralensis_Fisch0 247890.1	1.736656339	0.783839 405	2.2155767 23	0.026720 505	0.400474 809
Glycyrrhiza_uralensis_Fisch0 100520.1	1.736524453	2.008848 216	0.8644378 6	0.387347 402	0.908010 516
Glycyrrhiza_uralensis_Fisch0 272550.1	1.734133842	1.116560 968	1.5531026 89	0.120398 604	0.711449 612
Glycyrrhiza_uralensis_Fisch0	1.73387801	0.488497	3.5494099	0.000386	0.034487

050040.1		529	91	095	819
Glycyrrhiza_uralensis_Fisch0 064810.1	1.733509694	1.394403 222	1.2431911 13	0.213797 417	0.814082 192
Glycyrrhiza_uralensis_Fisch0 062720.1	1.732298455	4.039373 817	0.4288532 17	0.668030 048	NA
Glycyrrhiza_uralensis_Fisch0 036810.1	1.732298455	4.039373 817	0.4288532 17	0.668030 048	NA
Glycyrrhiza_uralensis_Fisch0 094940.1	1.732298455	4.039373 817	0.4288532 17	0.668030 048	NA
Glycyrrhiza_uralensis_Fisch0 113300.1	1.732298455	4.039373 817	0.4288532 17	0.668030 048	NA
Glycyrrhiza_uralensis_Fisch0 088630.1	1.732298455	4.039373 817	0.4288532 17	0.668030 048	NA
Glycyrrhiza_uralensis_Fisch0 126250.1	1.732298455	4.039373 817	0.4288532 17	0.668030 048	NA
Glycyrrhiza_uralensis_Fisch0 165600.1	1.732298455	4.039373 817	0.4288532 17	0.668030 048	NA
Glycyrrhiza_uralensis_Fisch0 098160.1	1.732298455	4.039373 817	0.4288532 17	0.668030 048	NA
Glycyrrhiza_uralensis_Fisch0 278160.1	1.732298455	4.039373 817	0.4288532 17	0.668030 048	NA
Glycyrrhiza_uralensis_Fisch0 238590.1	1.732298455	4.039373 817	0.4288532 17	0.668030 048	NA
Glycyrrhiza_uralensis_Fisch0 207260.1	1.732298455	4.039373 817	0.4288532 17	0.668030 048	NA
Glycyrrhiza_uralensis_Fisch0 056000.1	1.732298455	4.039373 817	0.4288532 17	0.668030 048	NA
Glycyrrhiza_uralensis_Fisch0 004030.1	1.732298455	4.039373 817	0.4288532 17	0.668030 048	NA
Glycyrrhiza_uralensis_Fisch0 055550.1	1.732298455	4.039373 817	0.4288532 17	0.668030 048	NA
Glycyrrhiza_uralensis_Fisch0 036730.1	1.732298455	4.039373 817	0.4288532 17	0.668030 048	NA
Glycyrrhiza_uralensis_Fisch0 210080.1	1.732298455	4.039373 817	0.4288532 17	0.668030 048	NA
Glycyrrhiza_uralensis_Fisch0 231810.1	1.732298455	4.039373 817	0.4288532 17	0.668030 048	NA
Glycyrrhiza_uralensis_Fisch0 052630.1	1.732298455	4.039373 817	0.4288532 17	0.668030 048	NA
Glycyrrhiza_uralensis_Fisch0 188020.1	1.730566484	0.431270 617	4.0127159 53	6.00E-05	0.008703 498
Glycyrrhiza_uralensis_Fisch0 135090.1	1.729883845	0.261346 053	6.6191313 1	3.61E-11	6.21E-08
Glycyrrhiza_uralensis_Fisch0	1.726741321	0.514180	3.3582372	0.000784	0.057563

222890.1		857	74	412	441
Glycyrrhiza_uralensis_Fisch0225360.1	1.726569681	0.425702243	4.055815329	5.00E-05	0.007515816
Glycyrrhiza_uralensis_Fisch0162410.1	1.726076905	0.878407956	1.965006001	0.049413543	0.528259591
Glycyrrhiza_uralensis_Fisch0267310.1	1.725988685	1.05537373	1.635428888	0.101959171	0.685194799
Glycyrrhiza_uralensis_Fisch0035740.1	1.724807438	0.632717847	2.726029376	0.006410129	0.199773779
Glycyrrhiza_uralensis_Fisch0020000.1	1.721732898	1.328247725	1.296243815	0.194891499	0.801825957
Glycyrrhiza_uralensis_Fisch0105670.1	1.721640899	0.62025463	2.775700198	0.005508298	0.185328029
Glycyrrhiza_uralensis_Fisch0057970.1	1.719279192	0.398956976	4.309435086	1.64E-05	0.003255857
Glycyrrhiza_uralensis_Fisch0211370.1	1.718566858	0.563491544	3.049853857	0.002289527	0.112238138
Glycyrrhiza_uralensis_Fisch0087160.1	1.713778769	0.487623034	3.514556636	0.000440489	0.037948295
Glycyrrhiza_uralensis_Fisch0096500.1	1.712548428	0.447383102	3.827923807	0.000129229	0.015870081
Glycyrrhiza_uralensis_Fisch0227830.1	1.7111274	1.988353063	0.860575233	0.389472032	0.908649002
Glycyrrhiza_uralensis_Fisch0140510.1	1.710500691	0.519607115	3.291911602	0.000995089	0.066165162
Glycyrrhiza_uralensis_Fisch0052260.1	1.709720401	2.765947539	0.618131898	0.536488397	0.94685993
Glycyrrhiza_uralensis_Fisch0114670.1	1.709708547	1.155491022	1.479638107	0.138969851	0.73701571
Glycyrrhiza_uralensis_Fisch0156840.1	1.709624811	3.034966559	0.563309275	0.573224304	0.952518696
Glycyrrhiza_uralensis_Fisch0235310.1	1.709482397	3.842995297	0.444830728	0.656442086	NA
Glycyrrhiza_uralensis_Fisch0020940.1	1.708472902	1.925862045	0.887121124	0.375013692	0.90447444
Glycyrrhiza_uralensis_Fisch0185350.1	1.708459719	0.614992744	2.778016059	0.005469192	0.184644923
Glycyrrhiza_uralensis_Fisch0153120.1	1.705853206	3.794492774	0.449560273	0.653027539	0.965935919
Glycyrrhiza_uralensis_Fisch0222690.1	1.703547293	0.630800488	2.700611881	0.006921205	0.20798179
Glycyrrhiza_uralensis_Fisch0126540.1	1.703113543	0.371500597	4.584416706	4.55E-06	0.001221279
Glycyrrhiza_uralensis_Fisch0	1.702152804	1.665067	1.0222728	0.306651	0.876615

135780.1		06	23	792	969
Glycyrrhiza_uralensis_Fisch0190410.1	1.699558795	0.386685906	4.39519198	1.11E-05	0.002357473
Glycyrrhiza_uralensis_Fisch0186170.1	1.695860403	0.666892989	2.542927324	0.010992809	0.261717014
Glycyrrhiza_uralensis_Fisch0212890.1	1.695317354	4.042758481	0.419346682	0.674962785	NA
Glycyrrhiza_uralensis_Fisch0231190.1	1.695317354	4.042758481	0.419346682	0.674962785	NA
Glycyrrhiza_uralensis_Fisch0048610.1	1.695317354	4.042758481	0.419346682	0.674962785	NA
Glycyrrhiza_uralensis_Fisch0235290.1	1.695317354	4.042758481	0.419346682	0.674962785	NA
Glycyrrhiza_uralensis_Fisch0037650.1	1.695317354	4.042758481	0.419346682	0.674962785	NA
Glycyrrhiza_uralensis_Fisch0231310.1	1.695317354	4.042758481	0.419346682	0.674962785	NA
Glycyrrhiza_uralensis_Fisch0129220.1	1.695317354	4.042758481	0.419346682	0.674962785	NA
Glycyrrhiza_uralensis_Fisch0147760.1	1.695317354	4.042758481	0.419346682	0.674962785	NA
Glycyrrhiza_uralensis_Fisch0027570.1	1.695317354	4.042758481	0.419346682	0.674962785	NA
Glycyrrhiza_uralensis_Fisch0086580.1	1.695317354	4.042758481	0.419346682	0.674962785	NA
Glycyrrhiza_uralensis_Fisch0273990.1	1.695317354	4.042758481	0.419346682	0.674962785	NA
Glycyrrhiza_uralensis_Fisch0201410.1	1.695317354	4.042758481	0.419346682	0.674962785	NA
Glycyrrhiza_uralensis_Fisch0243910.1	1.695317354	4.042758481	0.419346682	0.674962785	NA
Glycyrrhiza_uralensis_Fisch0164180.1	1.695317354	4.042758481	0.419346682	0.674962785	NA
Glycyrrhiza_uralensis_Fisch0156590.1	1.695317354	4.042758481	0.419346682	0.674962785	NA
Glycyrrhiza_uralensis_Fisch0119210.1	1.695317354	4.042758481	0.419346682	0.674962785	NA
Glycyrrhiza_uralensis_Fisch0276060.1	1.695317354	4.042758481	0.419346682	0.674962785	NA
Glycyrrhiza_uralensis_Fisch0000070.1	1.695317354	4.042758481	0.419346682	0.674962785	NA
Glycyrrhiza_uralensis_Fisch0138850.1	1.695317354	4.042758481	0.419346682	0.674962785	NA
Glycyrrhiza_uralensis_Fisch0	1.695317354	4.042758	0.4193466	0.674962	NA

286550.1		481	82	785	
Glycyrrhiza_uralensis_Fisch0 148050.1	1.695317354	4.042758 481	0.4193466 82	0.674962 785	NA
Glycyrrhiza_uralensis_Fisch0 072920.1	1.695317354	4.042758 481	0.4193466 82	0.674962 785	NA
Glycyrrhiza_uralensis_Fisch0 050100.1	1.69371924	0.821514 272	2.0617039 74	0.039235 928	0.481595 506
Glycyrrhiza_uralensis_Fisch0 027400.1	1.693680747	1.859325 045	0.9109115 98	0.362341 954	0.899040 488
Glycyrrhiza_uralensis_Fisch0 007300.1	1.693094758	1.223177 548	1.3841774 33	0.166304 132	0.772572 421
Glycyrrhiza_uralensis_Fisch0 275740.1	1.690978276	2.795037 835	0.6049929 82	0.545183 66	0.947866 184
Glycyrrhiza_uralensis_Fisch0 093280.1	1.69056509	0.804934 594	2.1002515 03	0.035706 723	0.459346 501
Glycyrrhiza_uralensis_Fisch0 284110.1	1.686926242	2.149132 588	0.7849335 36	0.432492 538	0.916951 181
Glycyrrhiza_uralensis_Fisch0 156610.1	1.686437809	0.726548 719	2.3211627 32	0.020278 063	0.347651 918
Glycyrrhiza_uralensis_Fisch0 191290.1	1.684346176	0.645743 21	2.6083838 73	0.009097 087	0.236747 677
Glycyrrhiza_uralensis_Fisch0 225390.1	1.683946129	1.865578 623	0.9026401 29	0.366716 921	0.900974 301
Glycyrrhiza_uralensis_Fisch0 124380.1	1.683800433	3.784680 947	0.4448989 11	0.656392 809	0.966321 504
Glycyrrhiza_uralensis_Fisch0 227130.1	1.681200328	1.236011 348	1.3601819 52	0.173772 352	0.782672 669
Glycyrrhiza_uralensis_Fisch0 052850.1	1.679343224	0.318767 863	5.2682325 26	1.38E-07	6.77E-05
Glycyrrhiza_uralensis_Fisch0 062320.1	1.67567175	0.610415 206	2.7451343 51	0.006048 613	0.194366 451
Glycyrrhiza_uralensis_Fisch0 155310.1	1.674706214	0.566018 542	2.9587479 72	0.003088 916	0.135277 149
Glycyrrhiza_uralensis_Fisch0 011740.1	1.673622276	1.593091 395	1.0505500 7	0.293465 283	0.868229 887
Glycyrrhiza_uralensis_Fisch0 115580.1	1.672469131	0.848460 544	1.9711807 98	0.048703 2	0.525688 795
Glycyrrhiza_uralensis_Fisch0 272930.1	1.671922153	1.443671 155	1.1581045 64	0.246821 368	0.842120 208
Glycyrrhiza_uralensis_Fisch0 097950.1	1.671422254	2.470431 634	0.6765709 41	0.498678 222	0.938408 631
Glycyrrhiza_uralensis_Fisch0 219150.1	1.669885179	2.390484 084	0.6985552 38	0.484830 026	0.933077 93
Glycyrrhiza_uralensis_Fisch0	1.669757577	0.647541	2.5786090	0.009919	0.245729

055220.1		967	49	898	
Glycyrrhiza_uralensis_Fisch0263790.1	1.668832463	0.909827584	1.834229357	0.066619955	0.591169659
Glycyrrhiza_uralensis_Fisch0128200.1	1.667991539	1.121038942	1.487897946	0.13677781	0.733728967
Glycyrrhiza_uralensis_Fisch0197400.1	1.667325891	2.869679111	0.58101475	0.561230512	0.950814695
Glycyrrhiza_uralensis_Fisch0049730.1	1.666068269	0.990425705	1.682173897	0.092535124	0.661782054
Glycyrrhiza_uralensis_Fisch0200710.1	1.664771554	1.031745422	1.613548768	0.106625337	0.692097679
Glycyrrhiza_uralensis_Fisch0061640.1	1.664429353	1.328842096	1.252541109	0.210372759	0.812109091
Glycyrrhiza_uralensis_Fisch0237510.1	1.663324616	3.214005221	0.517523931	0.604790468	NA
Glycyrrhiza_uralensis_Fisch0016490.1	1.663265387	0.399354381	4.164885792	3.12E-05	0.005394251
Glycyrrhiza_uralensis_Fisch0209130.1	1.660504665	1.299668512	1.277637067	0.201377425	0.806142846
Glycyrrhiza_uralensis_Fisch0265360.1	1.657869482	0.526712119	3.147581804	0.00164627	0.092152854
Glycyrrhiza_uralensis_Fisch0021570.1	1.657464695	0.799709085	2.072584553	0.03821096	0.477785872
Glycyrrhiza_uralensis_Fisch0161750.1	1.657025914	2.646600887	0.626095881	0.531252054	0.94589721
Glycyrrhiza_uralensis_Fisch0026200.1	1.654223795	0.801405846	2.064152393	0.03900327	0.481530651
Glycyrrhiza_uralensis_Fisch0280630.1	1.653728684	3.360760636	0.492069761	0.622670028	NA
Glycyrrhiza_uralensis_Fisch0075560.1	1.649931456	0.565880377	2.91568947	0.003549035	0.146276169
Glycyrrhiza_uralensis_Fisch0115740.1	1.648573641	0.569588753	2.89432267	0.003799775	0.150181571
Glycyrrhiza_uralensis_Fisch0032820.1	1.646858924	1.434543431	1.148002136	0.250967682	0.845365739
Glycyrrhiza_uralensis_Fisch0073720.1	1.64473182	2.337212718	0.703715074	0.481610231	0.932067114
Glycyrrhiza_uralensis_Fisch0154320.1	1.644635317	1.127489565	1.458670101	0.144655929	0.744212491
Glycyrrhiza_uralensis_Fisch0040470.1	1.644450814	1.057487013	1.555055327	0.119932893	0.711449612
Glycyrrhiza_uralensis_Fisch0145420.1	1.642872645	0.6133697	2.678437889	0.007396645	0.216064615
Glycyrrhiza_uralensis_Fisch0	1.64138483	1.359730	1.2071402	0.227378	0.824618

109280.1		058	12	151	366
Glycyrrhiza_uralensis_Fisch0091990.1	1.641305665	0.927971918	1.768701868	0.07694364	0.623502421
Glycyrrhiza_uralensis_Fisch0145530.1	1.640379042	0.605199358	2.710477169	0.006718647	0.205906576
Glycyrrhiza_uralensis_Fisch0000920.1	1.640227454	0.294509002	5.56936272	2.56E-08	1.92E-05
Glycyrrhiza_uralensis_Fisch0173680.1	1.639877662	0.418441109	3.919016623	8.89E-05	0.011823687
Glycyrrhiza_uralensis_Fisch0219740.1	1.639574967	2.058918071	0.796328416	0.425841173	0.916078027
Glycyrrhiza_uralensis_Fisch0038740.1	1.639519389	2.680334323	0.61168466	0.540746413	0.947110698
Glycyrrhiza_uralensis_Fisch0098860.1	1.639462372	0.948211109	1.729005657	0.083808083	0.641860957
Glycyrrhiza_uralensis_Fisch0283880.1	1.639385074	0.797534336	2.055566764	0.039824294	0.485844278
Glycyrrhiza_uralensis_Fisch0041180.1	1.638645138	1.372445298	1.193960256	0.232493515	0.828686349
Glycyrrhiza_uralensis_Fisch0113210.1	1.638119822	0.459968094	3.561377072	0.000368915	0.033763421
Glycyrrhiza_uralensis_Fisch0204960.1	1.637643795	0.437035775	3.747161875	0.000178847	0.020305847
Glycyrrhiza_uralensis_Fisch0153110.1	1.637021573	1.315710593	1.244210985	0.213421922	0.814082192
Glycyrrhiza_uralensis_Fisch0010360.1	1.636599188	3.846221167	0.425508341	0.670466142	NA
Glycyrrhiza_uralensis_Fisch0087420.1	1.636278325	1.096649199	1.49207087	0.135680567	0.731522758
Glycyrrhiza_uralensis_Fisch0207810.1	1.636256896	1.06766621	1.532554726	0.125385604	0.720086693
Glycyrrhiza_uralensis_Fisch0024900.1	1.635843674	2.098096227	0.779680004	0.435579252	0.917310907
Glycyrrhiza_uralensis_Fisch0171120.1	1.635555426	0.596384881	2.74244951	0.006098282	0.194366451
Glycyrrhiza_uralensis_Fisch0045380.1	1.632031262	0.872546219	1.870423854	0.061424982	0.572797664
Glycyrrhiza_uralensis_Fisch0012670.1	1.63073612	0.341663921	4.772924557	1.82E-06	0.000615547
Glycyrrhiza_uralensis_Fisch0155610.1	1.630314177	2.291712993	0.711395442	0.476839223	0.930559437
Glycyrrhiza_uralensis_Fisch0184950.1	1.63028018	1.624901501	1.003310157	0.315711237	0.882780387
Glycyrrhiza_uralensis_Fisch0	1.629712044	0.322203	5.0580182	4.24E-07	0.000179

117310.1		667	97		289
Glycyrrhiza_uralensis_Fisch0264390.1	1.629578432	1.990412459	0.818713943	0.41294964	0.911978305
Glycyrrhiza_uralensis_Fisch0092960.1	1.62843102	0.950384509	1.713444405	0.086630813	0.647355071
Glycyrrhiza_uralensis_Fisch0279730.1	1.62779914	0.45644833	3.566228709	0.000362155	0.033271294
Glycyrrhiza_uralensis_Fisch0142630.1	1.627280296	1.607514947	1.012295593	0.311396742	0.880015095
Glycyrrhiza_uralensis_Fisch0039790.1	1.626067805	0.557644745	2.915956477	0.003546	0.146276169
Glycyrrhiza_uralensis_Fisch0178700.1	1.625848118	2.19944846	0.739207191	0.459781195	0.92444875
Glycyrrhiza_uralensis_Fisch0170490.1	1.624146902	2.485677127	0.653402199	0.513497021	NA
Glycyrrhiza_uralensis_Fisch0243770.1	1.624068945	1.412073801	1.150130357	0.250090185	0.845097273
Glycyrrhiza_uralensis_Fisch0092670.1	1.622912571	0.51376143	3.158883633	0.001583747	0.090920841
Glycyrrhiza_uralensis_Fisch0245880.1	1.621897884	1.182207587	1.371923089	0.170087382	0.778850891
Glycyrrhiza_uralensis_Fisch0129500.1	1.620516818	0.435146662	3.724070433	0.000196036	0.021644914
Glycyrrhiza_uralensis_Fisch0007310.1	1.619909145	0.379649328	4.26685635	1.98E-05	0.003670614
Glycyrrhiza_uralensis_Fisch0121290.1	1.619550664	1.016215533	1.593707841	0.111001487	0.698786349
Glycyrrhiza_uralensis_Fisch0244980.1	1.618963268	0.562432158	2.87850409	0.003995661	0.154755979
Glycyrrhiza_uralensis_Fisch0061070.1	1.617751329	2.083001802	0.776644229	0.437368699	0.918289828
Glycyrrhiza_uralensis_Fisch0009300.1	1.614597861	1.511449047	1.068244983	0.285410019	0.862094057
Glycyrrhiza_uralensis_Fisch0147920.1	1.614536489	1.604338308	1.006356627	0.31424405	0.882272787
Glycyrrhiza_uralensis_Fisch0197960.1	1.614475763	0.454027638	3.555897544	0.000376691	0.034214903
Glycyrrhiza_uralensis_Fisch0220120.1	1.61342212	0.886499112	1.819992935	0.068760081	0.597968345
Glycyrrhiza_uralensis_Fisch0144940.1	1.612640181	0.269814581	5.976845941	2.27E-09	2.61E-06
Glycyrrhiza_uralensis_Fisch0236780.1	1.611450984	3.047899091	0.528708771	0.597007491	0.95798827
Glycyrrhiza_uralensis_Fisch0	1.610018069	0.842192	1.9116987	0.055914	0.553093

096990.1		335	91	839	989
Glycyrrhiza_uralensis_Fisch0 041260.1	1.609405341	0.652417 849	2.4668321 73	0.013631 423	0.288157 19
Glycyrrhiza_uralensis_Fisch0 004360.1	1.608656069	2.085506 451	0.7713503 2	0.440499 313	0.919303 851
Glycyrrhiza_uralensis_Fisch0 104280.1	1.606725241	1.300255 955	1.2356991 99	0.216570 396	0.816005 312
Glycyrrhiza_uralensis_Fisch0 012430.1	1.606585653	0.948071 269	1.6945832 08	0.090154 515	0.655594 916
Glycyrrhiza_uralensis_Fisch0 252100.1	1.604002994	1.920314 515	0.8352813 99	0.403559 288	0.910612 334
Glycyrrhiza_uralensis_Fisch0 037690.1	1.603466717	0.529100 327	3.0305532 55	0.002441 061	0.116986 155
Glycyrrhiza_uralensis_Fisch0 192130.1	1.603369477	1.530252 311	1.0477811 18	0.294739 464	0.869454 662
Glycyrrhiza_uralensis_Fisch0 272600.1	1.595243398	1.062421 241	1.5015168 53	0.133221 931	0.729217 799
Glycyrrhiza_uralensis_Fisch0 277320.1	1.595000883	2.331049 701	0.6842414 74	0.493822 697	NA
Glycyrrhiza_uralensis_Fisch0 001020.1	1.59472855	1.244908 202	1.2810009 18	0.200193 344	0.806142 846
Glycyrrhiza_uralensis_Fisch0 170120.1	1.592526614	2.114985 393	0.7529728 66	0.451466 219	0.922710 496
Glycyrrhiza_uralensis_Fisch0 071600.1	1.58978801	0.460572 712	3.4517633 55	0.000556 936	0.045442 198
Glycyrrhiza_uralensis_Fisch0 122480.1	1.589625978	0.529117 702	3.0042955 86	0.002661 966	0.122511 499
Glycyrrhiza_uralensis_Fisch0 236070.1	1.589257035	0.712324 899	2.2310844 91	0.025675 533	0.392365 156
Glycyrrhiza_uralensis_Fisch0 074920.1	1.589171342	1.985322 911	0.8004598 81	0.423444 399	0.914436 271
Glycyrrhiza_uralensis_Fisch0 011040.1	1.586064521	1.458202 879	1.0876843 98	0.276734 458	0.859692 133
Glycyrrhiza_uralensis_Fisch0 273760.1	1.58513586	0.983867 67	1.6111270 94	0.107152 024	0.692097 679
Glycyrrhiza_uralensis_Fisch0 009650.1	1.58434793	0.437079 69	3.6248491 21	0.000289 13	0.028521 983
Glycyrrhiza_uralensis_Fisch0 055360.1	1.58305639	0.648712 375	2.4403055 19	0.014674 845	0.300454 876
Glycyrrhiza_uralensis_Fisch0 158100.1	1.582328225	0.544878 562	2.9040016 17	0.003684 263	0.148900 684
Glycyrrhiza_uralensis_Fisch0 217950.1	1.58215025	1.959478 312	0.8074344 28	0.419416 238	0.913601 698
Glycyrrhiza_uralensis_Fisch0	1.579899611	1.016100	1.5548658	0.119978	0.711449

197950.1		297	1	032	612
Glycyrrhiza_uralensis_Fisch0035500.1	1.579515582	0.391795513	4.03147951	5.54E-05	0.008085599
Glycyrrhiza_uralensis_Fisch0002610.1	1.578255312	0.229225222	6.885173008	5.77E-12	1.74E-08
Glycyrrhiza_uralensis_Fisch0240210.1	1.578110159	3.842567915	0.410691546	0.681298726	NA
Glycyrrhiza_uralensis_Fisch0044660.1	1.578034349	0.414978526	3.802689179	0.000143134	0.017226164
Glycyrrhiza_uralensis_Fisch0226830.1	1.577863218	3.164859745	0.498557075	0.618091451	NA
Glycyrrhiza_uralensis_Fisch0196540.1	1.577399919	1.062982728	1.483937487	0.137825506	0.735920766
Glycyrrhiza_uralensis_Fisch0150820.1	1.575184332	0.908730351	1.733390251	0.083026329	0.640113947
Glycyrrhiza_uralensis_Fisch0125970.1	1.574600695	0.542846731	2.900635859	0.003724064	0.148900684
Glycyrrhiza_uralensis_Fisch0218020.1	1.573878714	0.59430918	2.648249038	0.00809099	0.226611014
Glycyrrhiza_uralensis_Fisch0054810.1	1.573428375	1.163759105	1.352022397	0.176368151	0.786144701
Glycyrrhiza_uralensis_Fisch0248720.1	1.573035732	0.474089898	3.3180115	0.000906608	0.062887732
Glycyrrhiza_uralensis_Fisch0076140.1	1.569624671	3.032534276	0.517595031	0.60474085	0.959659299
Glycyrrhiza_uralensis_Fisch0159750.1	1.569195155	2.245398318	0.69884935	0.484646184	0.933010769
Glycyrrhiza_uralensis_Fisch0179230.1	1.567878773	3.53406543	0.443647353	0.657297564	NA
Glycyrrhiza_uralensis_Fisch0151790.1	1.567702048	0.475217448	3.298915168	0.000970592	0.065267636
Glycyrrhiza_uralensis_Fisch0248120.1	1.56761246	1.68620192	0.929670665	0.352541627	0.897195704
Glycyrrhiza_uralensis_Fisch0016270.1	1.567272166	0.689250764	2.273878026	0.022973321	0.369137411
Glycyrrhiza_uralensis_Fisch0246030.1	1.567118314	1.575241621	0.994843136	0.319812563	0.885515059
Glycyrrhiza_uralensis_Fisch0040700.1	1.564698579	0.825043605	1.8965041	0.057893418	0.562073263
Glycyrrhiza_uralensis_Fisch0145000.1	1.563727148	0.85889078	1.82063562	0.068662265	0.597572186
Glycyrrhiza_uralensis_Fisch0048960.1	1.563065703	0.350624965	4.457941848	8.28E-06	0.001915193
Glycyrrhiza_uralensis_Fisch0	1.562751959	3.438347	0.4545066	0.649464	0.965935

277660.1		677	72	191	919
Glycyrrhiza_uralensis_Fisch0226580.1	1.561718181	0.777740014	2.00802087	0.044641074	0.508412002
Glycyrrhiza_uralensis_Fisch0037840.1	1.561397725	3.81593445	0.40917834	0.682408789	NA
Glycyrrhiza_uralensis_Fisch0271730.1	1.561176793	0.779217285	2.003519201	0.045121589	0.509005012
Glycyrrhiza_uralensis_Fisch0057200.1	1.56081499	0.67758911	2.303482991	0.021251683	0.357461923
Glycyrrhiza_uralensis_Fisch0004700.1	1.559415387	1.144936023	1.362010938	0.173194433	0.780672286
Glycyrrhiza_uralensis_Fisch0019140.1	1.554575524	0.756760515	2.054250313	0.039951471	0.486678992
Glycyrrhiza_uralensis_Fisch0201100.1	1.554052291	0.756938835	2.053075124	0.040065292	0.486678992
Glycyrrhiza_uralensis_Fisch0101000.1	1.553245163	1.063969882	1.459858206	0.144329048	0.744212491
Glycyrrhiza_uralensis_Fisch0033680.1	1.552683455	1.598518599	0.971326487	0.331385723	0.887837307
Glycyrrhiza_uralensis_Fisch0028910.1	1.551119031	3.216531203	0.482233478	0.629640097	NA
Glycyrrhiza_uralensis_Fisch0039770.1	1.550883775	1.838669243	0.843481655	0.398959122	0.910230427
Glycyrrhiza_uralensis_Fisch0063680.1	1.550131007	1.42322785	1.089165735	0.276080806	0.859487559
Glycyrrhiza_uralensis_Fisch0131510.1	1.549950697	0.344625332	4.497494971	6.88E-06	0.001671741
Glycyrrhiza_uralensis_Fisch0012810.1	1.549573433	1.210193494	1.280434443	0.200392387	0.806142846
Glycyrrhiza_uralensis_Fisch0019540.1	1.549162616	3.812550251	0.40633238	0.684498397	NA
Glycyrrhiza_uralensis_Fisch0128130.1	1.549100482	0.344793825	4.492831283	7.03E-06	0.001691699
Glycyrrhiza_uralensis_Fisch0200020.1	1.548715198	1.135634654	1.363744222	0.172648081	0.779564903
Glycyrrhiza_uralensis_Fisch0237290.1	1.547387173	1.144618978	1.351879711	0.176413799	0.786202581
Glycyrrhiza_uralensis_Fisch0177020.1	1.544494334	0.465069103	3.320999658	0.000896956	0.062398099
Glycyrrhiza_uralensis_Fisch0093360.1	1.542976747	2.550863674	0.604884049	0.545256043	0.947866184
Glycyrrhiza_uralensis_Fisch0168130.1	1.542074498	1.941105315	0.794431135	0.426944485	0.916400488
Glycyrrhiza_uralensis_Fisch0	1.541141977	1.711435	0.9004965	0.367856	0.900974

022440.1		777	29	072	301
Glycyrrhiza_uralensis_Fisch0051390.1	1.539016828	1.267556939	1.214159917	0.224686663	0.821475428
Glycyrrhiza_uralensis_Fisch0242840.1	1.538746446	2.073768636	0.742004879	0.458084379	0.924102621
Glycyrrhiza_uralensis_Fisch0200660.1	1.537904935	0.479553735	3.206950179	0.001341502	0.080323282
Glycyrrhiza_uralensis_Fisch0218840.1	1.536564842	1.020447804	1.505775049	0.13212493	0.727923
Glycyrrhiza_uralensis_Fisch0168180.1	1.535700508	1.508913348	1.017752617	0.308795535	0.877815097
Glycyrrhiza_uralensis_Fisch0251210.1	1.535596668	0.43936218	3.49505883	0.000473957	0.040169548
Glycyrrhiza_uralensis_Fisch0016790.1	1.533456894	2.877044836	0.532997218	0.594035506	0.957698234
Glycyrrhiza_uralensis_Fisch0065290.1	1.533301879	0.904324932	1.695520963	0.089976637	0.655331602
Glycyrrhiza_uralensis_Fisch0241440.1	1.532699277	0.472511846	3.243726674	0.001179769	0.074384456
Glycyrrhiza_uralensis_Fisch0072130.1	1.532311321	0.708654004	2.162284152	0.030596281	0.429168107
Glycyrrhiza_uralensis_Fisch0040900.1	1.532123982	0.74281188	2.062600266	0.039150622	0.481530651
Glycyrrhiza_uralensis_Fisch0260570.1	1.531637507	0.497821697	3.076678892	0.002093207	0.1069713
Glycyrrhiza_uralensis_Fisch0228390.1	1.53112846	2.01932736	0.758236871	0.448309192	0.922342258
Glycyrrhiza_uralensis_Fisch0022850.1	1.530560449	1.072860035	1.426617078	0.153690299	0.758333908
Glycyrrhiza_uralensis_Fisch0106230.1	1.530368952	0.885820511	1.727628715	0.084054811	0.64208365
Glycyrrhiza_uralensis_Fisch0224530.1	1.52898848	2.835600268	0.539211573	0.589740876	0.956703673
Glycyrrhiza_uralensis_Fisch0280590.1	1.52820618	3.216407493	0.475128286	0.634695538	NA
Glycyrrhiza_uralensis_Fisch0266600.1	1.527417814	1.524749228	1.001750181	0.316464264	0.883160394
Glycyrrhiza_uralensis_Fisch0265970.1	1.527319575	1.466634836	1.041376856	0.297700679	0.870247146
Glycyrrhiza_uralensis_Fisch0086720.1	1.527026308	1.930204739	0.791121417	0.428873142	0.916516843
Glycyrrhiza_uralensis_Fisch0208100.1	1.526127921	0.58379219	2.614162961	0.008944636	0.235243816
Glycyrrhiza_uralensis_Fisch0	1.525169465	3.814238	0.3998621	0.689258	NA

134060.1		318	32	065	
Glycyrrhiza_uralensis_Fisch0256300.1	1.524333428	2.887479183	0.527911487	0.597560772	0.95798827
Glycyrrhiza_uralensis_Fisch0070150.1	1.523966803	1.137312237	1.33997222	0.180254377	0.789145661
Glycyrrhiza_uralensis_Fisch0194150.1	1.523804522	0.670056282	2.274144074	0.022957325	0.369126803
Glycyrrhiza_uralensis_Fisch0050020.1	1.522431394	1.575666223	0.9662144	0.333936909	0.888735657
Glycyrrhiza_uralensis_Fisch0028430.1	1.520671084	0.862124557	1.763864713	0.077754759	0.626567477
Glycyrrhiza_uralensis_Fisch0118320.1	1.520499041	0.664292339	2.28890046	0.022085137	0.364287823
Glycyrrhiza_uralensis_Fisch0231220.1	1.520359007	1.522368656	0.998679919	0.317949771	0.884483859
Glycyrrhiza_uralensis_Fisch0181830.1	1.518670995	2.268667347	0.669411052	0.503233304	0.93941259
Glycyrrhiza_uralensis_Fisch0016730.1	1.518574166	1.382890302	1.098116145	0.272153776	0.857255693
Glycyrrhiza_uralensis_Fisch0262290.1	1.518496368	0.572317735	2.653240106	0.007972314	0.225415683
Glycyrrhiza_uralensis_Fisch0184600.1	1.518321456	0.745510444	2.036619966	0.04168814	0.49475144
Glycyrrhiza_uralensis_Fisch0269160.1	1.518135749	0.462959008	3.279201233	0.001041014	0.068275752
Glycyrrhiza_uralensis_Fisch0195370.1	1.517287839	0.519968516	2.918037905	0.003522415	0.146180234
Glycyrrhiza_uralensis_Fisch0173340.1	1.516710715	1.187401759	1.277335749	0.201483738	0.806142846
Glycyrrhiza_uralensis_Fisch0059230.1	1.514781617	3.732092279	0.405880001	NA	NA
Glycyrrhiza_uralensis_Fisch0074030.1	1.512725779	3.798510381	0.398241844	0.690451924	0.970066415
Glycyrrhiza_uralensis_Fisch0007260.1	1.512294732	0.40980336	3.690293637	0.000223995	0.023647227
Glycyrrhiza_uralensis_Fisch0006740.1	1.510635123	0.368480473	4.099634132	4.14E-05	0.006509972
Glycyrrhiza_uralensis_Fisch0157850.1	1.510241719	1.00142221	1.508096889	0.131529731	0.727359417
Glycyrrhiza_uralensis_Fisch0028840.1	1.509509963	1.497775744	1.00783443	0.313533954	0.882272787
Glycyrrhiza_uralensis_Fisch0193280.1	1.508293858	0.364195587	4.14143914	3.45E-05	0.005877175
Glycyrrhiza_uralensis_Fisch0	1.508083723	2.014577	0.7485857	0.454106	0.923405

051510.1		096	58	919	722
Glycyrrhiza_uralensis_Fisch0255450.1	1.505675845	0.675811432	2.227952612	0.025883675	0.393569212
Glycyrrhiza_uralensis_Fisch0250800.1	1.505560312	1.367645592	1.100840979	0.270965873	0.856487008
Glycyrrhiza_uralensis_Fisch0072730.1	1.505233178	0.740889509	2.031656758	0.042188416	0.49646066
Glycyrrhiza_uralensis_Fisch0142710.1	1.50429957	0.904590886	1.662961227	0.096320197	0.672537019
Glycyrrhiza_uralensis_Fisch0241810.1	1.503979552	0.632904171	2.376314805	0.017486537	0.324518845
Glycyrrhiza_uralensis_Fisch0151380.1	1.503846788	0.464043755	3.240743513	0.001192184	0.074642604
Glycyrrhiza_uralensis_Fisch0155990.1	1.501682499	0.625180407	2.401998657	0.016305768	0.315198656
Glycyrrhiza_uralensis_Fisch0085360.1	1.501134707	2.336020265	0.642603461	0.520481437	0.944123382
Glycyrrhiza_uralensis_Fisch0117420.1	1.499535684	2.366063833	0.633768059	0.526232201	0.944927971
Glycyrrhiza_uralensis_Fisch0008700.1	1.498850489	0.633427581	2.366253908	0.017969116	0.328213607
Glycyrrhiza_uralensis_Fisch0035180.1	1.498097455	1.309487883	1.144033079	0.252609919	0.845775428
Glycyrrhiza_uralensis_Fisch0193050.1	1.497956296	0.25637557	5.842819956	5.13E-09	4.58E-06
Glycyrrhiza_uralensis_Fisch0178150.1	1.497571815	0.755681947	1.98174883	0.047507359	0.521432801
Glycyrrhiza_uralensis_Fisch0150390.1	1.496508818	0.525612591	2.847170797	0.004410969	0.163426587
Glycyrrhiza_uralensis_Fisch0284740.1	1.495554037	0.843392167	1.773260526	0.076185547	0.622616391
Glycyrrhiza_uralensis_Fisch0187180.1	1.495543785	0.752719889	1.98685302	0.046938687	0.517862275
Glycyrrhiza_uralensis_Fisch0078470.1	1.495345	1.701530042	0.878823743	0.379496851	0.904607774
Glycyrrhiza_uralensis_Fisch0021900.1	1.494789933	0.619271694	2.41378695	0.015787691	0.312817878
Glycyrrhiza_uralensis_Fisch0061540.1	1.494403382	0.875261985	1.70737837	0.087751748	0.649994208
Glycyrrhiza_uralensis_Fisch0152070.1	1.493799328	0.533001134	2.802619418	0.005068945	0.177597523
Glycyrrhiza_uralensis_Fisch0262600.1	1.493040982	3.216155658	0.464231567	0.642481847	NA
Glycyrrhiza_uralensis_Fisch0	1.49174732	0.566735	2.6321749	0.008484	0.231268

093660.1		635	11	017	737
Glycyrrhiza_uralensis_Fisch0210290.1	1.491637839	3.81003798	0.391502092	0.695426143	NA
Glycyrrhiza_uralensis_Fisch0109970.1	1.488152258	1.581752695	0.94082486	0.346794619	0.895541946
Glycyrrhiza_uralensis_Fisch0088450.1	1.487391451	0.41901417	3.549740219	0.000385611	0.034487819
Glycyrrhiza_uralensis_Fisch0245830.1	1.486490334	3.765097881	0.394807886	0.692984675	0.970778182
Glycyrrhiza_uralensis_Fisch0034400.1	1.485196431	1.151258149	1.290063772	0.197028517	0.805858352
Glycyrrhiza_uralensis_Fisch0213620.1	1.484799732	0.959363528	1.547692495	0.12169635	0.714046956
Glycyrrhiza_uralensis_Fisch0265090.1	1.484361872	2.409166349	0.616130917	0.537808118	0.947110698
Glycyrrhiza_uralensis_Fisch0081690.1	1.483836736	0.58969014	2.516299048	0.011859451	0.269299039
Glycyrrhiza_uralensis_Fisch0138010.1	1.481367422	0.398978907	3.71289659	0.000204901	0.022216027
Glycyrrhiza_uralensis_Fisch0002470.1	1.481185241	0.429290527	3.450309638	0.000559944	0.045533277
Glycyrrhiza_uralensis_Fisch0113390.1	1.479997392	2.539627844	0.582761524	0.56005385	NA
Glycyrrhiza_uralensis_Fisch0046900.1	1.478181706	0.62041742	2.382559965	0.017192732	0.320739521
Glycyrrhiza_uralensis_Fisch0267000.1	1.476289288	0.65214201	2.263754313	0.02358923	0.375024047
Glycyrrhiza_uralensis_Fisch0093960.1	1.474263941	1.561466592	0.944153368	0.345091281	0.894851545
Glycyrrhiza_uralensis_Fisch0132470.1	1.473945969	0.86002414	1.713842554	0.086557646	0.647355071
Glycyrrhiza_uralensis_Fisch0163420.1	1.473700239	2.037178693	0.723402539	0.469432612	0.928837698
Glycyrrhiza_uralensis_Fisch0165530.1	1.471574291	0.912819796	1.612119169	0.10693601	0.692097679
Glycyrrhiza_uralensis_Fisch0082940.1	1.469288148	0.389004206	3.777049515	0.000158697	0.018364622
Glycyrrhiza_uralensis_Fisch0023880.1	1.468560526	3.232103285	0.454366831	0.649564822	NA
Glycyrrhiza_uralensis_Fisch0001170.1	1.468499106	0.34162691	4.298546343	1.72E-05	0.003284255
Glycyrrhiza_uralensis_Fisch0210410.1	1.467907394	0.631816762	2.323311889	0.020162404	0.347491768
Glycyrrhiza_uralensis_Fisch0	1.466176448	0.709272	2.0671556	0.038719	0.481530

084320.1		388	84	486	651
Glycyrrhiza_uralensis_Fisch0219340.1	1.465664327	1.501662279	0.976027931	0.329050623	0.887225647
Glycyrrhiza_uralensis_Fisch0196840.1	1.46486177	0.494643791	2.961447808	0.003061964	0.135232055
Glycyrrhiza_uralensis_Fisch0169170.1	1.464020851	0.767032204	1.908682378	0.056303078	0.554280198
Glycyrrhiza_uralensis_Fisch0174040.1	1.463619786	0.361412087	4.049725617	5.13E-05	0.007618856
Glycyrrhiza_uralensis_Fisch0126950.1	1.463014223	2.042040119	0.716447346	0.473715169	0.92896529
Glycyrrhiza_uralensis_Fisch0176100.1	1.462640061	2.138696901	0.683893103	0.494042669	0.937222783
Glycyrrhiza_uralensis_Fisch0167970.1	1.459595343	0.300534231	4.856669199	1.19E-06	0.000442061
Glycyrrhiza_uralensis_Fisch0000420.1	1.457854188	0.840009492	1.735521089	0.082648549	0.639868313
Glycyrrhiza_uralensis_Fisch0060160.1	1.456774108	0.680923063	2.139410732	0.032402419	0.440911127
Glycyrrhiza_uralensis_Fisch0146540.1	1.456191142	1.135673956	1.282226413	0.199763233	0.806142846
Glycyrrhiza_uralensis_Fisch0247470.1	1.456017803	1.602874261	0.908379302	0.363677857	0.899580325
Glycyrrhiza_uralensis_Fisch0265570.1	1.455114802	0.629822804	2.310355854	0.020868461	0.352741472
Glycyrrhiza_uralensis_Fisch0131570.1	1.454928057	0.663550844	2.192639902	0.028333334	0.414076106
Glycyrrhiza_uralensis_Fisch0220830.1	1.454076257	0.513708309	2.83054845	0.004646827	0.169212
Glycyrrhiza_uralensis_Fisch0033810.1	1.454042578	0.543297879	2.676326621	0.007443407	0.216904114
Glycyrrhiza_uralensis_Fisch0211620.1	1.452819434	1.541763006	0.942310477	0.346033706	0.895255665
Glycyrrhiza_uralensis_Fisch0209230.1	1.451998457	0.581955204	2.495034749	0.01259448	0.277600804
Glycyrrhiza_uralensis_Fisch0123210.1	1.450664925	1.145354987	1.26656359	0.205311378	0.81040421
Glycyrrhiza_uralensis_Fisch0035300.1	1.450420784	0.43975356	3.298258195	0.000972866	0.065267636
Glycyrrhiza_uralensis_Fisch0132070.1	1.4503274	0.674106542	2.151480973	0.031438256	0.434646078
Glycyrrhiza_uralensis_Fisch0132390.1	1.448774757	0.925449556	1.565482146	0.117469901	0.706303999
Glycyrrhiza_uralensis_Fisch0	1.44824173	0.256740	5.6408875	1.69E-08	1.31E-05

051880.1		045	85		
Glycyrrhiza_uralensis_Fisch0161190.1	1.448096657	1.275887663	1.13497191	0.256387088	0.84740043
Glycyrrhiza_uralensis_Fisch0149840.1	1.448016529	3.237581232	0.447252571	0.654692708	NA
Glycyrrhiza_uralensis_Fisch0039960.1	1.445743267	0.854018755	1.692870629	0.090480095	0.656094692
Glycyrrhiza_uralensis_Fisch0079520.1	1.44322387	3.911097135	0.369007422	0.712122193	NA
Glycyrrhiza_uralensis_Fisch0170460.1	1.441339306	1.900107634	0.758556663	0.448117805	0.922133502
Glycyrrhiza_uralensis_Fisch0137650.1	1.441206666	0.475565227	3.03051313	0.002441386	0.116986155
Glycyrrhiza_uralensis_Fisch0173610.1	1.440770623	0.447765939	3.217686958	0.001292288	0.078351048
Glycyrrhiza_uralensis_Fisch0176610.1	1.440366477	0.87735123	1.641721613	0.10064771	0.681807835
Glycyrrhiza_uralensis_Fisch0259780.1	1.439539987	0.819915973	1.755716481	0.079136831	0.630836091
Glycyrrhiza_uralensis_Fisch0032230.1	1.439287321	1.856965176	0.77507502	0.438295331	0.91828988
Glycyrrhiza_uralensis_Fisch0180570.1	1.438541694	0.370800219	3.879559994	0.000104646	0.013542039
Glycyrrhiza_uralensis_Fisch0057760.1	1.438143449	0.650121789	2.212113905	0.026958796	0.4020435
Glycyrrhiza_uralensis_Fisch0024640.1	1.437269227	0.757768744	1.896712207	0.057865932	0.562073263
Glycyrrhiza_uralensis_Fisch0245680.1	1.436793074	3.076260749	0.467058287	0.640458177	0.965035372
Glycyrrhiza_uralensis_Fisch0092660.1	1.435953376	0.581759518	2.468293738	0.013575887	0.28815719
Glycyrrhiza_uralensis_Fisch0009960.1	1.435784075	1.253769344	1.145174016	0.25213708	0.845727356
Glycyrrhiza_uralensis_Fisch0147410.1	1.435419704	0.791977226	1.812450734	0.06991658	0.600931266
Glycyrrhiza_uralensis_Fisch0257090.1	1.432565163	0.499995147	2.865158136	0.00416801	0.158239765
Glycyrrhiza_uralensis_Fisch0060230.1	1.431606472	0.657413582	2.177634463	0.029433262	0.422462527
Glycyrrhiza_uralensis_Fisch0038400.1	1.431447732	0.418621055	3.419435582	0.000627512	0.049522003
Glycyrrhiza_uralensis_Fisch0030000.1	1.429723155	0.550033529	2.599338186	0.00934037	0.237026417
Glycyrrhiza_uralensis_Fisch0	1.428768283	0.556193	2.5688341	0.010204	0.249861

212640.1		287	02	129	034
Glycyrrhiza_uralensis_Fisch0215990.1	1.426637712	0.528609379	2.698850547	0.006957942	0.208047509
Glycyrrhiza_uralensis_Fisch0253840.1	1.424292538	1.398487488	1.018452114	0.308463147	0.877728803
Glycyrrhiza_uralensis_Fisch0218440.1	1.42376683	1.457698673	0.97672232	0.328706642	0.887225647
Glycyrrhiza_uralensis_Fisch0041240.1	1.42290319	0.695560206	2.045693786	0.040786508	0.491323029
Glycyrrhiza_uralensis_Fisch0212790.1	1.420990883	1.525356445	0.931579559	0.351553851	0.897130588
Glycyrrhiza_uralensis_Fisch0022860.1	1.420923875	0.843089449	1.685377366	0.091915788	0.66060154
Glycyrrhiza_uralensis_Fisch0285640.1	1.420686802	0.611802828	2.322131798	0.02022584	0.347491768
Glycyrrhiza_uralensis_Fisch0254930.1	1.419308824	0.506865793	2.800166913	0.005107619	0.178174474
Glycyrrhiza_uralensis_Fisch0258620.1	1.415508282	0.30880817	4.583778597	4.57E-06	0.001221279
Glycyrrhiza_uralensis_Fisch0273430.1	1.41546703	0.543132469	2.606117498	0.009157504	0.236747677
Glycyrrhiza_uralensis_Fisch0135760.1	1.415115635	0.816382035	1.733398794	0.083024812	0.640113947
Glycyrrhiza_uralensis_Fisch0167990.1	1.414696042	0.265876564	5.320875289	1.03E-07	5.64E-05
Glycyrrhiza_uralensis_Fisch0149790.1	1.414188533	0.403359395	3.506026015	0.000454851	0.038704929
Glycyrrhiza_uralensis_Fisch0002570.1	1.413948493	1.54651461	0.914280721	0.360569358	0.89825134
Glycyrrhiza_uralensis_Fisch0160750.1	1.413520805	3.850807345	0.367071286	0.713565845	NA
Glycyrrhiza_uralensis_Fisch0021080.1	1.413509697	0.806263389	1.753161208	0.07957434	0.63213015
Glycyrrhiza_uralensis_Fisch0095810.1	1.412949554	0.477700959	2.957811844	0.003098312	0.135300221
Glycyrrhiza_uralensis_Fisch0105250.1	1.412314793	1.506130865	0.937710544	0.348393184	0.896081753
Glycyrrhiza_uralensis_Fisch0254830.1	1.411304786	0.514188526	2.74472244	0.00605621	0.194366451
Glycyrrhiza_uralensis_Fisch0002680.1	1.41114785	1.894780517	0.744755309	0.456419656	0.92402224
Glycyrrhiza_uralensis_Fisch0205240.1	1.409410042	0.294455349	4.78649835	1.70E-06	0.000592039
Glycyrrhiza_uralensis_Fisch0	1.408826385	2.386466	0.5903398	0.554962	0.949166

140920.1		744	35	838	55
Glycyrrhiza_uralensis_Fisch0184160.1	1.404811662	3.141435068	0.447187872	0.654739418	0.965983804
Glycyrrhiza_uralensis_Fisch0222460.1	1.403924815	0.42230273	3.324451197	0.000885927	0.061809484
Glycyrrhiza_uralensis_Fisch0063590.1	1.40375294	0.503125991	2.790062461	0.005269787	0.18142708
Glycyrrhiza_uralensis_Fisch0018800.1	1.400464318	0.472259371	2.965455857	0.003022347	0.134949303
Glycyrrhiza_uralensis_Fisch0236590.1	1.399469422	3.816196912	0.366718347	0.71382912	NA
Glycyrrhiza_uralensis_Fisch0172970.1	1.398333387	1.51719219	0.921658704	0.356706627	0.897807124
Glycyrrhiza_uralensis_Fisch0164400.1	1.397853311	3.120399182	0.447972593	0.654172977	0.965983804
Glycyrrhiza_uralensis_Fisch0249830.1	1.396634356	0.643259943	2.171181919	0.029917424	0.425598337
Glycyrrhiza_uralensis_Fisch0105190.1	1.395761846	0.77903759	1.791648907	0.073189224	0.610477279
Glycyrrhiza_uralensis_Fisch0156920.1	1.395733987	0.799793487	1.745117971	0.080964336	0.63655336
Glycyrrhiza_uralensis_Fisch0184520.1	1.395511165	0.876994651	1.591242504	0.111555011	0.698786349
Glycyrrhiza_uralensis_Fisch0113320.1	1.3954385	2.86156441	0.487648817	0.625798611	0.963502294
Glycyrrhiza_uralensis_Fisch0167910.1	1.394627996	1.422718789	0.980255555	0.326959988	0.887225647
Glycyrrhiza_uralensis_Fisch0199800.1	1.394148382	0.587655004	2.372392598	0.0176733	0.325724612
Glycyrrhiza_uralensis_Fisch0098310.1	1.393237498	1.494471067	0.932261272	0.351201517	0.897130588
Glycyrrhiza_uralensis_Fisch0255930.1	1.393107739	0.256239334	5.436744304	5.43E-08	3.53E-05
Glycyrrhiza_uralensis_Fisch0252500.1	1.392760946	1.15444797	1.206430244	0.22765164	0.824920193
Glycyrrhiza_uralensis_Fisch0182000.1	1.391554069	0.507718038	2.740800928	0.006128963	0.194366451
Glycyrrhiza_uralensis_Fisch0107770.1	1.391396192	0.473935906	2.935831986	0.003326544	0.139494642
Glycyrrhiza_uralensis_Fisch0168090.1	1.39126555	2.01671389	0.68986759	0.490277459	0.936011543
Glycyrrhiza_uralensis_Fisch0212800.1	1.39002897	2.92347265	0.475471857	0.634450688	0.963606249
Glycyrrhiza_uralensis_Fisch0	1.389295903	0.429098	3.2377064	0.001204	0.074943

262560.1		781	8	947	351
Glycyrrhiza_uralensis_Fisch0 049120.1	1.386994352	1.251035 812	1.1086767 77	0.267569 644	0.854958 36
Glycyrrhiza_uralensis_Fisch0 082210.1	1.385895123	1.251432 203	1.1074472 27	0.268100 617	0.855296 51
Glycyrrhiza_uralensis_Fisch0 237120.1	1.38371402	3.816454 583	0.3625653 05	0.716929 632	NA
Glycyrrhiza_uralensis_Fisch0 202640.1	1.383586093	0.661839 852	2.0905149 32	0.036571 568	0.466729 236
Glycyrrhiza_uralensis_Fisch0 184930.1	1.381934053	0.699921 839	1.9744119 65	0.048334 919	0.524360 715
Glycyrrhiza_uralensis_Fisch0 114530.1	1.379973284	0.426607 632	3.2347599 57	0.001217 45	0.075297 97
Glycyrrhiza_uralensis_Fisch0 254000.1	1.378637954	0.795610 389	1.7328053 71	0.083130 268	0.640302 576
Glycyrrhiza_uralensis_Fisch0 140450.1	1.37792045	0.906557 869	1.5199475 92	0.128524 148	0.721820 17
Glycyrrhiza_uralensis_Fisch0 028310.1	1.376862861	1.830621 561	0.7521286 16	0.451973 717	0.922710 496
Glycyrrhiza_uralensis_Fisch0 273280.1	1.37647351	1.198393 214	1.1485992 2	0.250721 279	0.845365 739
Glycyrrhiza_uralensis_Fisch0 249970.1	1.376402186	0.375855 08	3.6620555 56	0.000250 2	0.025626 825
Glycyrrhiza_uralensis_Fisch0 256360.1	1.376285983	2.081553 836	0.6611820 26	0.508495 586	0.940226 457
Glycyrrhiza_uralensis_Fisch0 191090.1	1.376079916	0.377148 084	3.6486461 82	0.000263 626	0.026774 156
Glycyrrhiza_uralensis_Fisch0 061900.1	1.375721219	2.635379 575	0.5220201 42	0.601656 307	NA
Glycyrrhiza_uralensis_Fisch0 107710.1	1.37568814	0.791467 777	1.7381480 09	0.082184 737	0.637992 565
Glycyrrhiza_uralensis_Fisch0 006780.1	1.375661893	0.771016 593	1.7842182 72	0.074388 204	0.616359 408
Glycyrrhiza_uralensis_Fisch0 265340.1	1.375410444	0.345895 565	3.9763749 07	7.00E-05	0.009849 526
Glycyrrhiza_uralensis_Fisch0 203530.1	1.37507663	0.607999 127	2.2616424 4	0.023719 504	0.376105 7
Glycyrrhiza_uralensis_Fisch0 214040.1	1.374411775	0.483532 707	2.8424380 71	0.004476 993	0.164772 503
Glycyrrhiza_uralensis_Fisch0 002880.1	1.374268711	0.486943 099	2.8222367 53	0.004768 996	0.171072 616
Glycyrrhiza_uralensis_Fisch0 195460.1	1.372563461	0.804679 829	1.7057261 93	0.088059 07	0.650432 137
Glycyrrhiza_uralensis_Fisch0	1.371426016	0.455959	3.0077827	0.002631	0.121346

138000.1		139	11	612	565
Glycyrrhiza_uralensis_Fisch0200090.1	1.370905809	3.177358395	0.431460867	0.666133301	0.966368724
Glycyrrhiza_uralensis_Fisch0090460.1	1.370001737	0.725345358	1.888757849	0.058924283	0.564515005
Glycyrrhiza_uralensis_Fisch0284910.1	1.366101605	0.699232429	1.953716031	0.050734809	0.532279959
Glycyrrhiza_uralensis_Fisch0187560.1	1.365748788	2.253540179	0.606045901	0.544484274	0.947851963
Glycyrrhiza_uralensis_Fisch0179170.1	1.365491113	0.456480705	2.991344648	0.002777518	0.126517411
Glycyrrhiza_uralensis_Fisch0136570.1	1.364226894	0.828656823	1.646311062	0.099699733	0.680826549
Glycyrrhiza_uralensis_Fisch0052830.1	1.364167787	0.432765208	3.152212243	0.001620384	0.09177095
Glycyrrhiza_uralensis_Fisch0025410.1	1.364039805	0.282186494	4.833823844	1.34E-06	0.000488457
Glycyrrhiza_uralensis_Fisch0137070.1	1.36319698	0.608747778	2.239346128	0.025133404	0.387669509
Glycyrrhiza_uralensis_Fisch0004190.1	1.362795949	0.713844315	1.909094071	0.056249958	0.554210592
Glycyrrhiza_uralensis_Fisch0235130.1	1.362647197	0.972190345	1.401625931	0.161026979	0.766294858
Glycyrrhiza_uralensis_Fisch0115280.1	1.362292471	0.801267437	1.700172012	0.089098575	0.652861515
Glycyrrhiza_uralensis_Fisch0103390.1	1.361021559	0.623163377	2.184052545	0.028958387	0.418635663
Glycyrrhiza_uralensis_Fisch0126220.1	1.36021203	0.590991672	2.301575629	0.021359116	0.35752011
Glycyrrhiza_uralensis_Fisch0068910.1	1.359413744	0.504870209	2.692600435	0.007089718	0.209900994
Glycyrrhiza_uralensis_Fisch0166580.1	1.359274346	0.552364895	2.460826816	0.013861726	0.288929335
Glycyrrhiza_uralensis_Fisch0235000.1	1.35890715	2.143571237	0.633945411	0.526116448	0.944927971
Glycyrrhiza_uralensis_Fisch0167980.1	1.357786081	0.228768951	5.93518515	2.94E-09	2.83E-06
Glycyrrhiza_uralensis_Fisch0059890.1	1.355893457	0.611088203	2.218817921	0.026499114	0.39889536
Glycyrrhiza_uralensis_Fisch0219270.1	1.355177989	0.928930067	1.458859	0.14460392	0.744212491
Glycyrrhiza_uralensis_Fisch0019430.1	1.354842619	0.424744546	3.189782262	0.0014238	0.084411013
Glycyrrhiza_uralensis_Fisch0	1.354423355	0.508326	2.6644758	0.007710	0.222009

114860.1		386	03	841	511
Glycyrrhiza_uralensis_Fisch0 043430.1	1.354421474	0.356461 875	3.7996250 63	0.000144 915	0.017267 862
Glycyrrhiza_uralensis_Fisch0 216140.1	1.354259353	0.823380 44	1.6447553 12	0.100020 281	0.681007 723
Glycyrrhiza_uralensis_Fisch0 024280.1	1.354100792	1.503868 357	0.9004117 85	0.367901 151	0.900974 301
Glycyrrhiza_uralensis_Fisch0 234430.1	1.35297124	0.396312 832	3.4138971 33	0.000640 408	0.049743 04
Glycyrrhiza_uralensis_Fisch0 035360.1	1.350837311	0.447356 292	3.0196005 64	0.002531 083	0.118477 067
Glycyrrhiza_uralensis_Fisch0 112540.1	1.350305535	2.862079 112	0.4717918 28	0.637075 385	0.964454 661
Glycyrrhiza_uralensis_Fisch0 094650.1	1.34922177	0.542238 055	2.4882461 8	0.012837 484	0.280301 476
Glycyrrhiza_uralensis_Fisch0 041110.1	1.348699612	0.886656 707	1.5211068 74	0.128233 02	0.721820 17
Glycyrrhiza_uralensis_Fisch0 116480.1	1.347844986	2.821171 171	0.4777607 97	0.632820 467	0.963563 011
Glycyrrhiza_uralensis_Fisch0 275810.1	1.346021144	0.652185 474	2.0638625 02	0.039030 755	0.481530 651
Glycyrrhiza_uralensis_Fisch0 072600.1	1.345687718	0.461728 878	2.9144543 11	0.003563 11	0.146605 21
Glycyrrhiza_uralensis_Fisch0 274570.1	1.344041136	1.419223 438	0.9470257 47	0.343625 659	0.894315 373
Glycyrrhiza_uralensis_Fisch0 200490.1	1.343621757	0.676012 891	1.9875682 47	0.046859 461	0.517388 636
Glycyrrhiza_uralensis_Fisch0 044430.1	1.343223432	0.400759 62	3.3516935 45	0.000803 189	0.058056 316
Glycyrrhiza_uralensis_Fisch0 000890.1	1.339094453	0.860276 629	1.5565858 8	0.119568 838	0.711149 478
Glycyrrhiza_uralensis_Fisch0 202040.1	1.33826859	0.568999 198	2.3519692 04	0.018674 321	0.334620 502
Glycyrrhiza_uralensis_Fisch0 161980.1	1.336066235	0.947493 015	1.4101066 85	0.158508 184	0.762308 231
Glycyrrhiza_uralensis_Fisch0 141950.1	1.336065402	1.588187 109	0.8412518 86	0.400206 838	0.910230 427
Glycyrrhiza_uralensis_Fisch0 120830.1	1.336015238	0.608121 696	2.1969537 44	0.028023 75	0.410798 818
Glycyrrhiza_uralensis_Fisch0 092260.1	1.33522479	0.414000 054	3.2251802 27	0.001258 934	0.077302 394
Glycyrrhiza_uralensis_Fisch0 120840.1	1.334746473	0.351409 378	3.7982665 17	0.000145 712	0.017277 232
Glycyrrhiza_uralensis_Fisch0	1.334637856	0.563863	2.3669505	0.017935	0.328159

244240.1		857	3	33	75
Glycyrrhiza_uralensis_Fisch0195940.1	1.334597958	0.972717925	1.372029778	0.170054168	0.778850891
Glycyrrhiza_uralensis_Fisch0045510.1	1.333505371	0.693142339	1.923855022	0.054372758	0.548459977
Glycyrrhiza_uralensis_Fisch0277340.1	1.332818665	0.7414455	1.79759492	0.072241224	0.606540739
Glycyrrhiza_uralensis_Fisch0135600.1	1.331909445	0.565729795	2.354320838	0.018556587	0.334574564
Glycyrrhiza_uralensis_Fisch0200400.1	1.330073526	1.359600334	0.97828273	0.327934509	0.887225647
Glycyrrhiza_uralensis_Fisch0285010.1	1.330035601	0.676357637	1.966467928	0.049244584	0.528259591
Glycyrrhiza_uralensis_Fisch0268240.1	1.329241059	0.631237303	2.105770764	0.03522427	0.456503854
Glycyrrhiza_uralensis_Fisch0218650.1	1.329087921	0.48487076	2.741117901	0.006123053	0.194366451
Glycyrrhiza_uralensis_Fisch0061510.1	1.328107011	0.838894612	1.583163119	0.11338429	0.701200196
Glycyrrhiza_uralensis_Fisch0152030.1	1.32756584	0.761805199	1.742657887	0.081393392	0.636559454
Glycyrrhiza_uralensis_Fisch0201770.1	1.327473701	1.255367917	1.05743797	0.29031175	0.866789345
Glycyrrhiza_uralensis_Fisch0017240.1	1.327329546	0.499084061	2.65953103	0.007824952	0.223689547
Glycyrrhiza_uralensis_Fisch0214070.1	1.326627506	0.63705124	2.082450236	0.037301364	0.472157283
Glycyrrhiza_uralensis_Fisch0199900.1	1.32608357	1.159940299	1.143234329	0.252941313	0.845987168
Glycyrrhiza_uralensis_Fisch0275800.1	1.324933314	1.363565438	0.971668302	0.331215591	0.887837307
Glycyrrhiza_uralensis_Fisch0145800.1	1.324583952	1.483200016	0.89305821	0.371826001	0.90343083
Glycyrrhiza_uralensis_Fisch0160930.1	1.324060189	1.109051435	1.193867252	0.232529899	0.828693319
Glycyrrhiza_uralensis_Fisch0044260.1	1.323166067	0.252519296	5.239861215	1.61E-07	7.74E-05
Glycyrrhiza_uralensis_Fisch0140090.1	1.322900424	0.256538077	5.156741007	2.51E-07	0.000116316
Glycyrrhiza_uralensis_Fisch0105330.1	1.321607194	0.552217225	2.39327412	0.01669876	0.317487481
Glycyrrhiza_uralensis_Fisch0184890.1	1.319922101	1.214559082	1.086750015	0.277147304	0.859762186
Glycyrrhiza_uralensis_Fisch0	1.318280263	0.434407	3.0346636	0.002408	0.115923

203070.1		37	68	041	074
Glycyrrhiza_uralensis_Fisch0225510.1	1.318017333	0.368180348	3.579814461	0.000343838	0.032203061
Glycyrrhiza_uralensis_Fisch0005320.1	1.317059996	2.183228494	0.603262553	0.54633404	0.948067715
Glycyrrhiza_uralensis_Fisch0223900.1	1.31690258	0.475157062	2.771510064	0.005579695	0.186015585
Glycyrrhiza_uralensis_Fisch0218230.1	1.316850829	0.658458039	1.999900906	0.045510965	0.510460827
Glycyrrhiza_uralensis_Fisch0079330.1	1.316588993	0.517074933	2.546224751	0.010889505	0.260708981
Glycyrrhiza_uralensis_Fisch0074070.1	1.316389529	0.462726185	2.844856359	0.004443145	0.164028391
Glycyrrhiza_uralensis_Fisch0041080.1	1.315976073	3.998103626	0.329150066	0.742042263	NA
Glycyrrhiza_uralensis_Fisch0000910.1	1.315729022	1.023437514	1.285597805	0.198583463	0.806142846
Glycyrrhiza_uralensis_Fisch0246320.1	1.314908397	0.589417622	2.230860338	0.025690382	0.392365156
Glycyrrhiza_uralensis_Fisch0179840.1	1.314332545	0.469772518	2.797806372	0.005145095	0.178704801
Glycyrrhiza_uralensis_Fisch0203140.1	1.31428364	0.296230948	4.436685799	9.14E-06	0.002053687
Glycyrrhiza_uralensis_Fisch0058210.1	1.312913259	2.171827708	0.604519988	0.545497985	0.948067715
Glycyrrhiza_uralensis_Fisch0042770.1	1.311361386	0.599266286	2.188278262	0.028649339	0.416736426
Glycyrrhiza_uralensis_Fisch0164380.1	1.311294917	0.330542196	3.967102937	7.28E-05	0.010180991
Glycyrrhiza_uralensis_Fisch0230050.1	1.309959025	1.402042545	0.934321879	0.350137882	0.897065603
Glycyrrhiza_uralensis_Fisch0202860.1	1.30970732	0.457494003	2.862785767	0.004199344	0.158935585
Glycyrrhiza_uralensis_Fisch0153680.1	1.308783425	0.767441425	1.705385431	0.088122564	0.650432137
Glycyrrhiza_uralensis_Fisch0230130.1	1.308770971	0.281450945	4.650085554	3.32E-06	0.000962212
Glycyrrhiza_uralensis_Fisch0133840.1	1.307602562	1.298799843	1.006777579	0.314041671	0.882272787
Glycyrrhiza_uralensis_Fisch0171850.1	1.307488529	0.582771463	2.243569929	0.024860086	0.386551852
Glycyrrhiza_uralensis_Fisch0041010.1	1.30675759	1.568740073	0.832998158	0.404845768	0.910612334
Glycyrrhiza_uralensis_Fisch0	1.304021461	1.283186	1.0162365	0.309516	0.878348

266790.1		929	53	754	221
Glycyrrhiza_uralensis_Fisch0032520.1	1.303471421	0.718951476	1.813017239	0.069829162	0.600786806
Glycyrrhiza_uralensis_Fisch0216930.1	1.302179068	0.411560034	3.164007584	0.001556127	0.089934495
Glycyrrhiza_uralensis_Fisch0158410.1	1.302109321	0.554283085	2.349177444	0.018814938	0.334719555
Glycyrrhiza_uralensis_Fisch0035270.1	1.30192041	0.479585935	2.71467596	0.006634065	0.204045234
Glycyrrhiza_uralensis_Fisch0281300.1	1.300676008	0.867818311	1.498788388	0.133928538	0.730409401
Glycyrrhiza_uralensis_Fisch0286130.1	1.300672594	2.633881737	0.49382346	0.621430863	0.962594079
Glycyrrhiza_uralensis_Fisch0130940.1	1.300126476	0.397121289	3.273877558	0.001060826	0.06938607
Glycyrrhiza_uralensis_Fisch0175050.1	1.299274509	2.187227631	0.594028025	0.552493369	0.94916655
Glycyrrhiza_uralensis_Fisch0032290.1	1.29880318	0.629595014	2.062918466	0.039120375	0.481530651
Glycyrrhiza_uralensis_Fisch0018010.1	1.298731221	2.723025835	0.476944142	0.633401898	0.963563011
Glycyrrhiza_uralensis_Fisch0169220.1	1.298272744	0.191743187	6.770893728	1.28E-11	3.42E-08
Glycyrrhiza_uralensis_Fisch0188390.1	1.296345785	0.485197266	2.6717912	0.007544758	0.218534683
Glycyrrhiza_uralensis_Fisch0257580.1	1.295740628	0.568061541	2.280986362	0.022549254	0.366834848
Glycyrrhiza_uralensis_Fisch0236200.1	1.294222956	3.527295895	0.366916469	0.713681326	NA
Glycyrrhiza_uralensis_Fisch0277220.1	1.293888592	0.376669717	3.435074639	0.000592391	0.047688437
Glycyrrhiza_uralensis_Fisch0248590.1	1.293820603	0.320535805	4.036430824	5.43E-05	0.007965191
Glycyrrhiza_uralensis_Fisch0265150.1	1.29344395	1.33568589	0.968374345	0.332857447	0.888510555
Glycyrrhiza_uralensis_Fisch0274650.1	1.293215908	0.513788359	2.517020649	0.011835189	0.269150608
Glycyrrhiza_uralensis_Fisch0020780.1	1.292313842	2.447455204	0.528023491	0.597483031	0.95798827
Glycyrrhiza_uralensis_Fisch0286230.1	1.292184416	1.650416276	0.782944543	0.433659679	0.916951181
Glycyrrhiza_uralensis_Fisch0209150.1	1.291745201	3.851615698	0.335377489	0.737340347	NA
Glycyrrhiza_uralensis_Fisch0	1.291592746	0.582283	2.2181524	0.026544	0.399196

253040.1		137	16	442	096
Glycyrrhiza_uralensis_Fisch0219190.1	1.289877257	0.640626148	2.013463328	0.044065909	0.505740037
Glycyrrhiza_uralensis_Fisch0165980.1	1.289127467	1.234704523	1.044077707	0.296449439	0.869761143
Glycyrrhiza_uralensis_Fisch0038240.1	1.288556026	2.855855927	0.45119784	0.651846966	0.965935919
Glycyrrhiza_uralensis_Fisch0072950.1	1.287886032	0.489719177	2.629846029	0.008542355	0.231916014
Glycyrrhiza_uralensis_Fisch0272510.1	1.287188913	2.077198604	0.619675418	0.535471503	NA
Glycyrrhiza_uralensis_Fisch0281650.1	1.287150763	0.656397628	1.960931465	0.049887018	0.529504363
Glycyrrhiza_uralensis_Fisch0019640.1	1.286146617	0.355941804	3.613362081	0.000302252	0.029335522
Glycyrrhiza_uralensis_Fisch0111560.1	1.28564907	0.695658735	1.848103107	0.064587428	0.583086844
Glycyrrhiza_uralensis_Fisch0203820.1	1.284470879	2.989507962	0.429659628	0.667443255	0.966501962
Glycyrrhiza_uralensis_Fisch0061090.1	1.284209024	0.544266739	2.359521409	0.018298525	0.33160581
Glycyrrhiza_uralensis_Fisch0040950.1	1.28408009	0.964791025	1.330941163	0.183208371	0.791962817
Glycyrrhiza_uralensis_Fisch0110830.1	1.283856815	1.933084628	0.664149306	0.506594755	0.940226457
Glycyrrhiza_uralensis_Fisch0211720.1	1.282517016	2.224438139	0.576557735	0.564238257	0.951408356
Glycyrrhiza_uralensis_Fisch0143950.1	1.282147794	0.695701954	1.842955574	0.065335497	0.585271833
Glycyrrhiza_uralensis_Fisch0109170.1	1.281433074	3.064569994	0.418144495	0.675841474	0.968644098
Glycyrrhiza_uralensis_Fisch0042120.1	1.280893465	1.503406587	0.851994049	0.394217395	0.910003682
Glycyrrhiza_uralensis_Fisch0204080.1	1.27932844	2.45414961	0.521291952	0.602163407	0.959512721
Glycyrrhiza_uralensis_Fisch0182870.1	1.278429435	1.316401523	0.971154631	0.331471283	0.887837307
Glycyrrhiza_uralensis_Fisch0265720.1	1.277187159	0.539179459	2.368760786	0.017847793	0.32694177
Glycyrrhiza_uralensis_Fisch0248630.1	1.276241801	2.708991786	0.471113204	0.637559896	0.964482882
Glycyrrhiza_uralensis_Fisch0159580.1	1.275233236	0.814584866	1.565500771	0.117465537	0.706303999
Glycyrrhiza_uralensis_Fisch0	1.27375855	3.854060	0.3304978	0.741023	NA

201190.1		347	22	838	
Glycyrrhiza_uralensis_Fisch0128230.1	1.27271306	0.261764779	4.862048538	1.16E-06	0.000436935
Glycyrrhiza_uralensis_Fisch0010560.1	1.27228372	0.418870069	3.037418558	0.002386139	0.115257626
Glycyrrhiza_uralensis_Fisch0146720.1	1.271579116	3.525627685	0.360667441	0.718348067	NA
Glycyrrhiza_uralensis_Fisch0058980.1	1.270946964	0.509184351	2.496044823	0.012558673	0.277327763
Glycyrrhiza_uralensis_Fisch0182030.1	1.268632635	0.615911458	2.059764628	0.039421048	0.481901786
Glycyrrhiza_uralensis_Fisch0210700.1	1.267521906	1.172897575	1.080675698	0.279841397	0.86117142
Glycyrrhiza_uralensis_Fisch0123970.1	1.267402761	1.796506482	0.705481875	0.480510408	0.93190416
Glycyrrhiza_uralensis_Fisch0083080.1	1.26573196	0.405203142	3.123697298	0.001785941	0.097257005
Glycyrrhiza_uralensis_Fisch0256190.1	1.262672448	0.998474772	1.264601253	0.206014304	0.81096352
Glycyrrhiza_uralensis_Fisch0172190.1	1.261764693	0.522933094	2.412860663	0.015827869	0.313045859
Glycyrrhiza_uralensis_Fisch0227820.1	1.261649974	0.677236979	1.862937219	0.0624711	0.57502079
Glycyrrhiza_uralensis_Fisch0085190.1	1.26146856	0.869210157	1.45128143	0.14670151	0.745903116
Glycyrrhiza_uralensis_Fisch0075050.1	1.260835214	0.347614035	3.627112503	0.000286608	0.028389568
Glycyrrhiza_uralensis_Fisch0151470.1	1.260738089	1.639195233	0.769120153	0.441821985	0.919801681
Glycyrrhiza_uralensis_Fisch0120820.1	1.260502285	0.282136385	4.467705518	7.91E-06	0.001868405
Glycyrrhiza_uralensis_Fisch0260710.1	1.259993065	1.411235534	0.892829747	0.371948353	0.90343083
Glycyrrhiza_uralensis_Fisch0006020.1	1.259224832	2.904472114	0.433546883	0.664617514	0.96632848
Glycyrrhiza_uralensis_Fisch0131670.1	1.254816961	0.385518912	3.254877837	0.001134412	0.072620439
Glycyrrhiza_uralensis_Fisch0033490.1	1.254548196	1.023897392	1.225267498	0.22047445	0.817830773
Glycyrrhiza_uralensis_Fisch0144960.1	1.254177034	0.511981684	2.449652152	0.014299428	0.295186308
Glycyrrhiza_uralensis_Fisch0152660.1	1.25279717	0.604281068	2.073202747	0.038153415	0.477562504
Glycyrrhiza_uralensis_Fisch0	1.25270906	0.478176	2.6197628	0.008799	0.233353

073200.1		51	6	093	815
Glycyrrhiza_uralensis_Fisch0105490.1	1.25252667	1.092896378	1.14606169	0.251769628	0.845555314
Glycyrrhiza_uralensis_Fisch0024160.1	1.252491983	1.519275446	0.824400859	0.409711827	0.911645974
Glycyrrhiza_uralensis_Fisch0036830.1	1.252467015	1.157027793	1.082486542	0.279036392	0.860526068
Glycyrrhiza_uralensis_Fisch0036490.1	1.252467015	1.157027793	1.082486542	0.279036392	0.860526068
Glycyrrhiza_uralensis_Fisch0036980.1	1.252038551	3.319571283	0.37716875	0.706048202	NA
Glycyrrhiza_uralensis_Fisch0049160.1	1.252035937	1.692164289	0.739902116	0.459359391	0.92417089
Glycyrrhiza_uralensis_Fisch0215470.1	1.252020553	2.286499731	0.547570829	0.583986624	0.954856307
Glycyrrhiza_uralensis_Fisch0005700.1	1.251508914	0.530178655	2.360541873	0.018248258	0.331111164
Glycyrrhiza_uralensis_Fisch0098640.1	1.250845564	0.919904869	1.359755347	0.173907357	0.782981034
Glycyrrhiza_uralensis_Fisch0010680.1	1.250500586	0.470396723	2.658395616	0.007851367	0.223912795
Glycyrrhiza_uralensis_Fisch0178100.1	1.249471137	0.480549188	2.600089998	0.009319931	0.237026417
Glycyrrhiza_uralensis_Fisch0189830.1	1.24853702	2.100471624	0.594407944	0.552239296	NA
Glycyrrhiza_uralensis_Fisch0187390.1	1.248296207	0.576127168	2.166702554	0.030257542	0.427154856
Glycyrrhiza_uralensis_Fisch0013190.1	1.247952669	0.458244906	2.723331241	0.006462722	0.200325214
Glycyrrhiza_uralensis_Fisch0277590.1	1.247584707	1.439902375	0.866437009	0.386250556	0.907294904
Glycyrrhiza_uralensis_Fisch0046450.1	1.24741149	0.649916114	1.919342303	0.054941028	0.549638627
Glycyrrhiza_uralensis_Fisch0093920.1	1.247180625	0.548546918	2.273607932	0.02298957	0.36915207
Glycyrrhiza_uralensis_Fisch0103640.1	1.241746861	1.572182841	0.789823441	0.429630888	0.916516843
Glycyrrhiza_uralensis_Fisch0024430.1	1.24113518	0.296747889	4.182456648	2.88E-05	0.005066577
Glycyrrhiza_uralensis_Fisch0056730.1	1.240861496	2.115918485	0.586441068	0.557579153	0.949683712
Glycyrrhiza_uralensis_Fisch0106600.1	1.238527916	0.913799407	1.355360822	0.175302626	0.784445844
Glycyrrhiza_uralensis_Fisch0	1.238039344	1.683849	0.7352436	0.462191	0.924920

249990.1		153	18	139	246
Glycyrrhiza_uralensis_Fisch0161250.1	1.237642468	1.500622632	0.824752634	0.409512043	0.911645974
Glycyrrhiza_uralensis_Fisch0137040.1	1.235974017	0.984104934	1.255937222	0.209138732	0.811966832
Glycyrrhiza_uralensis_Fisch0268270.1	1.234841143	0.910378005	1.356404851	0.174970391	0.784165295
Glycyrrhiza_uralensis_Fisch0003130.1	1.23354021	0.772427187	1.596966329	0.110273213	0.697894385
Glycyrrhiza_uralensis_Fisch0262850.1	1.232591983	0.499443317	2.467931675	0.013589626	0.28815719
Glycyrrhiza_uralensis_Fisch0043840.1	1.231277337	1.444695745	0.852274495	0.394061759	0.910003682
Glycyrrhiza_uralensis_Fisch0247140.1	1.231231972	0.477821932	2.576759017	0.009973145	0.246208829
Glycyrrhiza_uralensis_Fisch0280230.1	1.231019401	0.457686207	2.68965807	0.007152526	0.21100414
Glycyrrhiza_uralensis_Fisch0211600.1	1.230318935	0.561670476	2.190463961	0.028490606	0.416121893
Glycyrrhiza_uralensis_Fisch0071450.1	1.230059436	2.190571836	0.561524355	0.574440132	0.952518696
Glycyrrhiza_uralensis_Fisch0226130.1	1.228748969	1.130623823	1.086788501	0.277130291	0.859762186
Glycyrrhiza_uralensis_Fisch0207640.1	1.228464676	1.3486713	0.910870333	0.362363699	0.899040488
Glycyrrhiza_uralensis_Fisch0073470.1	1.22780345	1.307651756	0.938937637	0.347762764	0.896081753
Glycyrrhiza_uralensis_Fisch0279290.1	1.227040522	2.792969138	0.43933193	NA	NA
Glycyrrhiza_uralensis_Fisch0191080.1	1.22592588	0.518765432	2.363160313	0.018119829	0.330162221
Glycyrrhiza_uralensis_Fisch0168100.1	1.225660446	0.690286675	1.775581783	0.075801875	0.621652854
Glycyrrhiza_uralensis_Fisch0144000.1	1.22524523	3.190248589	0.384059485	0.700934359	NA
Glycyrrhiza_uralensis_Fisch0196700.1	1.224955492	2.157018318	0.567892948	0.570107661	0.952518696
Glycyrrhiza_uralensis_Fisch0045710.1	1.224905514	0.482826794	2.536946024	0.011182419	0.263883172
Glycyrrhiza_uralensis_Fisch0053210.1	1.224596048	2.020567966	0.606065259	0.544471419	0.947851963
Glycyrrhiza_uralensis_Fisch0123500.1	1.224292513	1.316283947	0.930112774	0.352312698	0.897130588
Glycyrrhiza_uralensis_Fisch0	1.224054957	0.556345	2.2001694	0.027794	0.409303

052180.1		753	98	872	419
Glycyrrhiza_uralensis_Fisch0282650.1	1.223745657	0.646984536	1.891460444	0.058562906	0.56343726
Glycyrrhiza_uralensis_Fisch0231110.1	1.222684804	3.790765122	0.322543013	0.747041358	0.97735001
Glycyrrhiza_uralensis_Fisch0221500.1	1.222537023	2.599759253	0.47025009	0.638176351	NA
Glycyrrhiza_uralensis_Fisch0265700.1	1.2223643	0.364373134	3.354704796	0.000794497	0.057790453
Glycyrrhiza_uralensis_Fisch0050130.1	1.221634959	2.732475909	0.447079865	0.654817397	0.965983804
Glycyrrhiza_uralensis_Fisch0017780.1	1.221134139	2.628023382	0.46465878	0.642175831	0.965035372
Glycyrrhiza_uralensis_Fisch0048180.1	1.221015955	0.551480752	2.214068125	0.026824093	0.40127776
Glycyrrhiza_uralensis_Fisch0067680.1	1.220564403	0.67312027	1.813293193	0.069786613	0.600786806
Glycyrrhiza_uralensis_Fisch0033700.1	1.220051229	0.698280495	1.747222266	0.080598791	0.635622246
Glycyrrhiza_uralensis_Fisch0222200.1	1.220001068	0.465826035	2.619005756	0.008818646	0.233353815
Glycyrrhiza_uralensis_Fisch0192660.1	1.219802837	0.283461742	4.303236223	1.68E-05	0.003284255
Glycyrrhiza_uralensis_Fisch0061870.1	1.219716803	2.115032383	0.576689422	0.564149279	0.951408356
Glycyrrhiza_uralensis_Fisch0041270.1	1.219081766	1.432706217	0.850894449	0.394827989	0.910082902
Glycyrrhiza_uralensis_Fisch0224600.1	1.218873599	0.630391542	1.933518327	0.053172378	0.543233926
Glycyrrhiza_uralensis_Fisch0272520.1	1.218496682	0.700788207	1.738751695	0.082078449	0.637960598
Glycyrrhiza_uralensis_Fisch0045350.1	1.216994064	0.46216593	2.633240539	0.008457443	0.230867625
Glycyrrhiza_uralensis_Fisch0025070.1	1.216859924	0.433785525	2.805210997	0.005028365	0.176538318
Glycyrrhiza_uralensis_Fisch0198390.1	1.216025841	0.593718411	2.048152489	0.04054506	0.489673657
Glycyrrhiza_uralensis_Fisch0262210.1	1.215979415	1.625398701	0.748111472	0.454392923	0.923623358
Glycyrrhiza_uralensis_Fisch0183920.1	1.215597302	0.324590799	3.745014665	0.000180383	0.020384153
Glycyrrhiza_uralensis_Fisch0219750.1	1.214986758	0.318278621	3.817368421	0.000134883	0.016397101
Glycyrrhiza_uralensis_Fisch0	1.214642584	2.684562	0.4524546	0.650941	0.965935

154210.1		287	1	513	919
Glycyrrhiza_uralensis_Fisch0210280.1	1.21366807	0.469268844	2.586295862	0.009701362	0.242735735
Glycyrrhiza_uralensis_Fisch0072060.1	1.213341254	0.500155326	2.425928887	0.015269262	0.308008088
Glycyrrhiza_uralensis_Fisch0016870.1	1.211728527	1.223807498	0.990130007	0.322110578	0.886479038
Glycyrrhiza_uralensis_Fisch0108230.1	1.211340698	0.747114521	1.621358793	0.104940713	0.692097679
Glycyrrhiza_uralensis_Fisch0130690.1	1.211293457	0.988721704	1.225110618	0.220533545	0.817830773
Glycyrrhiza_uralensis_Fisch0065040.1	1.210724493	0.580594067	2.085320127	0.037040252	0.469977674
Glycyrrhiza_uralensis_Fisch0002750.1	1.209971228	1.127066244	1.073558218	0.283020733	0.86117142
Glycyrrhiza_uralensis_Fisch0219470.1	1.209918167	0.737200701	1.641233067	0.100749044	0.681807835
Glycyrrhiza_uralensis_Fisch0142660.1	1.209104405	1.273868693	0.949159369	0.342539563	0.894315373
Glycyrrhiza_uralensis_Fisch0203040.1	1.206869226	0.440203626	2.741615819	0.00611378	0.194366451
Glycyrrhiza_uralensis_Fisch0065020.1	1.206349629	0.512801265	2.352470073	0.01864919	0.334620502
Glycyrrhiza_uralensis_Fisch0229030.1	1.204861743	0.924185842	1.303700714	0.192335646	0.799842258
Glycyrrhiza_uralensis_Fisch0047670.1	1.204084309	1.352920967	0.889988653	0.373471979	0.904190987
Glycyrrhiza_uralensis_Fisch0193080.1	1.20293672	1.257502778	0.956607604	0.338765349	0.89281528
Glycyrrhiza_uralensis_Fisch0032060.1	1.201564615	1.230783769	0.976259718	0.328935776	0.887225647
Glycyrrhiza_uralensis_Fisch0225020.1	1.200854451	0.791540921	1.517109754	0.129238976	0.723101384
Glycyrrhiza_uralensis_Fisch0069560.1	1.200042407	0.419862226	2.858181406	0.004260767	0.159994804
Glycyrrhiza_uralensis_Fisch0047200.1	1.197878432	2.624364272	0.456445184	0.648069881	0.965935919
Glycyrrhiza_uralensis_Fisch0015130.1	1.196738383	0.6664422	1.79571219	0.072540302	0.607066235
Glycyrrhiza_uralensis_Fisch0115780.1	1.19652833	0.31668537	3.778287353	0.000157911	0.018361876
Glycyrrhiza_uralensis_Fisch0189730.1	1.196463689	0.793622086	1.507598781	0.131657245	0.727359417
Glycyrrhiza_uralensis_Fisch0	1.196288485	1.190898	1.0045259	0.315125	0.882780

017770.1		531	56	167	387
Glycyrrhiza_uralensis_Fisch0 279840.1	1.195801156	0.482078 133	2.4805131 65	0.013119 342	0.282914 881
Glycyrrhiza_uralensis_Fisch0 132650.1	1.195331005	2.107459 015	0.5671906 29	0.570584 674	0.952518 696
Glycyrrhiza_uralensis_Fisch0 182330.1	1.194646035	0.997190 494	1.1980118 57	0.230912 401	0.828030 507
Glycyrrhiza_uralensis_Fisch0 204030.1	1.192778394	1.576754 6	0.7564768 76	0.449363 331	0.922628 42
Glycyrrhiza_uralensis_Fisch0 135190.1	1.192616725	0.532137 461	2.2411816 74	0.025014 309	0.386983 097
Glycyrrhiza_uralensis_Fisch0 146590.1	1.192005423	1.391349 646	0.8567260 05	0.391596 328	0.908755 153
Glycyrrhiza_uralensis_Fisch0 217520.1	1.191689083	0.432966 588	2.7523811 65	0.005916 361	0.192699 972
Glycyrrhiza_uralensis_Fisch0 056130.1	1.191267201	0.946035 109	1.2592209 21	0.207950 547	0.811901 001
Glycyrrhiza_uralensis_Fisch0 206280.1	1.190925367	0.523393 901	2.2753902 26	0.022882 531	0.369126 803
Glycyrrhiza_uralensis_Fisch0 218050.1	1.190149993	0.545611 956	2.1813121 58	0.029160 336	0.420796 931
Glycyrrhiza_uralensis_Fisch0 079730.1	1.189862437	1.044566 535	1.1390968 38	0.254662 766	0.847381 568
Glycyrrhiza_uralensis_Fisch0 152370.1	1.189181519	2.315661 498	0.5135385 81	0.607574 631	0.960732 446
Glycyrrhiza_uralensis_Fisch0 213310.1	1.189063573	0.576050 864	2.0641642 02	0.039002 15	0.481530 651
Glycyrrhiza_uralensis_Fisch0 152840.1	1.188788713	1.343371 794	0.8849290 41	0.376194 904	0.904514 347
Glycyrrhiza_uralensis_Fisch0 022280.1	1.187638771	0.455049 633	2.6099104 05	0.009056 594	0.236526 869
Glycyrrhiza_uralensis_Fisch0 194930.1	1.187438608	2.117244 368	0.5608415 47	0.574905 561	0.952700 643
Glycyrrhiza_uralensis_Fisch0 055270.1	1.186965074	0.495761 82	2.3942244 57	0.016655 553	0.317487 481
Glycyrrhiza_uralensis_Fisch0 107270.1	1.186847179	1.302704 198	0.9110642 16	0.362261 539	0.899040 488
Glycyrrhiza_uralensis_Fisch0 126000.1	1.186224351	0.527153 584	2.2502443 06	0.024433 441	0.381907 034
Glycyrrhiza_uralensis_Fisch0 275620.1	1.185415282	1.343150 694	0.8825631 31	0.377472 356	0.904607 774
Glycyrrhiza_uralensis_Fisch0 073690.1	1.184340019	0.683920 173	1.7316933 54	0.083328 175	0.641118 002
Glycyrrhiza_uralensis_Fisch0	1.18426425	2.615299	0.4528215	0.650677	0.965935

115240.1		918	84	22	919
Glycyrrhiza_uralensis_Fisch0148110.1	1.183618642	0.558624529	2.118808933	0.034106617	0.451552119
Glycyrrhiza_uralensis_Fisch0128220.1	1.181670492	0.500432336	2.361299236	0.018211029	0.331111164
Glycyrrhiza_uralensis_Fisch0283630.1	1.181394008	0.676323627	1.746788019	0.080674116	0.635622246
Glycyrrhiza_uralensis_Fisch0145010.1	1.180936332	3.913054593	0.301793983	0.762809118	NA
Glycyrrhiza_uralensis_Fisch0204690.1	1.180569202	0.486946147	2.424434836	0.015332235	0.308052495
Glycyrrhiza_uralensis_Fisch0277240.1	1.178968096	0.444745796	2.65088081	0.008028217	0.225746701
Glycyrrhiza_uralensis_Fisch0041700.1	1.178707525	1.178256217	1.00038303	0.317125179	0.883788406
Glycyrrhiza_uralensis_Fisch0023150.1	1.178641526	1.27991754	0.920873017	0.357116728	0.897807124
Glycyrrhiza_uralensis_Fisch0251120.1	1.176823512	0.627160851	1.876430121	0.060596242	0.571804506
Glycyrrhiza_uralensis_Fisch0111680.1	1.175709072	0.425570494	2.762665854	0.005733143	0.189217067
Glycyrrhiza_uralensis_Fisch0037550.1	1.175000115	1.616089537	0.727063747	0.467186905	0.927438253
Glycyrrhiza_uralensis_Fisch0150780.1	1.174893045	3.917642737	0.299897955	0.764254994	NA
Glycyrrhiza_uralensis_Fisch0207820.1	1.174335096	0.465030759	2.525284777	0.011560457	0.267234253
Glycyrrhiza_uralensis_Fisch0103040.1	1.174025012	0.689743747	1.70211766	0.088733314	0.652861515
Glycyrrhiza_uralensis_Fisch0033640.1	1.173733184	0.555385862	2.113365254	0.034569518	0.45379288
Glycyrrhiza_uralensis_Fisch0144310.1	1.173364502	0.424216959	2.765953783	0.005675658	0.188172291
Glycyrrhiza_uralensis_Fisch0085520.1	1.173015036	0.492244471	2.382992812	0.01717253	0.320739521
Glycyrrhiza_uralensis_Fisch0150460.1	1.173001275	0.430554039	2.724399655	0.00644185	0.200325214
Glycyrrhiza_uralensis_Fisch0104090.1	1.172937763	1.583943123	0.740517602	0.458985984	0.924102621
Glycyrrhiza_uralensis_Fisch0081860.1	1.171308773	0.98300802	1.191555663	0.233435519	0.830232642
Glycyrrhiza_uralensis_Fisch0206590.1	1.171056724	0.45026608	2.600810446	0.009300383	0.237026417
Glycyrrhiza_uralensis_Fisch0	1.169986871	0.380466	3.0751387	0.002104	0.107297

147490.1		364	79	046	443
Glycyrrhiza_uralensis_Fisch0286450.1	1.169598936	3.612209767	0.323790425	0.746096706	NA
Glycyrrhiza_uralensis_Fisch0003060.1	1.168995109	1.019594675	1.146529241	0.251576235	0.845377633
Glycyrrhiza_uralensis_Fisch0009600.1	1.168366753	0.443226745	2.636047502	0.008387799	0.23021017
Glycyrrhiza_uralensis_Fisch0263810.1	1.168140669	2.076860434	0.562455064	0.573806011	0.952518696
Glycyrrhiza_uralensis_Fisch0056290.1	1.168093017	0.417317982	2.79904789	0.005125354	0.178276386
Glycyrrhiza_uralensis_Fisch0054700.1	1.167764743	1.555227149	0.750864428	0.45273425	0.922710496
Glycyrrhiza_uralensis_Fisch0159060.1	1.167458519	0.591792006	1.97275142	0.04852389	0.525390669
Glycyrrhiza_uralensis_Fisch0022430.1	1.166803503	0.819718375	1.423419968	0.154614447	0.758615083
Glycyrrhiza_uralensis_Fisch0142260.1	1.166603229	0.793714164	1.46980271	0.141615195	0.74193126
Glycyrrhiza_uralensis_Fisch0284780.1	1.166549583	1.524640339	0.765130998	0.444193543	0.920507951
Glycyrrhiza_uralensis_Fisch0018660.1	1.166500986	1.001633535	1.164598574	0.244181521	0.839393558
Glycyrrhiza_uralensis_Fisch0115590.1	1.166392887	0.305056491	3.823530791	0.000131554	0.016073647
Glycyrrhiza_uralensis_Fisch0233130.1	1.165910718	0.342086421	3.408234428	0.000653847	0.050442616
Glycyrrhiza_uralensis_Fisch0169720.1	1.165024587	0.614730821	1.895178421	0.058068768	0.562219369
Glycyrrhiza_uralensis_Fisch0155960.1	1.16457247	0.7871524	1.47947522	0.13901335	0.73701571
Glycyrrhiza_uralensis_Fisch0185890.1	1.164503179	0.661470448	1.7604765	0.078327049	0.627606986
Glycyrrhiza_uralensis_Fisch0255650.1	1.164345117	0.776774555	1.498948582	0.133886972	0.730409401
Glycyrrhiza_uralensis_Fisch0238230.1	1.163746803	1.115441616	1.043305886	0.296806646	0.869761143
Glycyrrhiza_uralensis_Fisch0047100.1	1.162389069	0.477824449	2.432669719	0.014987965	0.303670311
Glycyrrhiza_uralensis_Fisch0243790.1	1.162198986	1.444409183	0.80461894	0.421039609	0.913755795
Glycyrrhiza_uralensis_Fisch0148160.1	1.161940989	1.341896601	0.865894577	0.386547978	0.90734218
Glycyrrhiza_uralensis_Fisch0	1.161274037	0.665026	1.7462075	0.080774	0.635999

182960.1		339	84	887	849
Glycyrrhiza_uralensis_Fisch0260580.1	1.161097793	0.274060582	4.23664645	2.27E-05	0.00410607
Glycyrrhiza_uralensis_Fisch0178470.1	1.159637461	1.621272332	0.715263832	0.474446033	0.929128306
Glycyrrhiza_uralensis_Fisch0220850.1	1.158282365	0.535008399	2.16497978	0.030389234	0.428009864
Glycyrrhiza_uralensis_Fisch0204210.1	1.157769359	0.64988905	1.781487715	0.07483281	0.618340453
Glycyrrhiza_uralensis_Fisch0216500.1	1.155927511	0.651976865	1.772957866	0.076235689	0.622659642
Glycyrrhiza_uralensis_Fisch0145520.1	1.155587753	0.621106249	1.86053152	0.062810363	0.57702658
Glycyrrhiza_uralensis_Fisch0234480.1	1.155501193	0.37637059	3.070115533	0.00213976	0.107974882
Glycyrrhiza_uralensis_Fisch0075020.1	1.155493913	0.934862314	1.236004378	0.216456936	0.816005312
Glycyrrhiza_uralensis_Fisch0075460.1	1.155124041	0.440373973	2.62305248	0.008714585	0.232899664
Glycyrrhiza_uralensis_Fisch0247620.1	1.154549686	1.010458843	1.142599418	0.253204947	0.846082697
Glycyrrhiza_uralensis_Fisch0207290.1	1.154381704	0.803853515	1.436059784	0.15098533	0.754151361
Glycyrrhiza_uralensis_Fisch0208470.1	1.153689746	1.235827144	0.933536499	0.350543034	0.897130588
Glycyrrhiza_uralensis_Fisch0215750.1	1.153197626	0.458670384	2.514218633	0.011929645	0.270382826
Glycyrrhiza_uralensis_Fisch0165390.1	1.152981854	0.736268963	1.565979163	0.117353495	0.706303999
Glycyrrhiza_uralensis_Fisch0056780.1	1.152831679	0.566202756	2.03607571	0.041742753	0.49475144
Glycyrrhiza_uralensis_Fisch0006090.1	1.150509076	0.468855825	2.453865378	0.014132988	0.292754751
Glycyrrhiza_uralensis_Fisch0149200.1	1.149405835	0.390322945	2.944755999	0.003232094	0.137365504
Glycyrrhiza_uralensis_Fisch0195700.1	1.146674957	1.509916667	0.7594293	0.447595792	0.921789335
Glycyrrhiza_uralensis_Fisch0121300.1	1.14649886	0.615372972	1.863095899	0.062448775	0.57502079
Glycyrrhiza_uralensis_Fisch0248030.1	1.146339212	0.397368847	2.88482406	0.003916325	0.15314671
Glycyrrhiza_uralensis_Fisch0277580.1	1.146280811	0.886985401	1.292333345	0.196241729	0.80474948
Glycyrrhiza_uralensis_Fisch0	1.146075866	1.047568	1.0940338	0.273940	0.858000

210840.1		922	54	131	161
Glycyrrhiza_uralensis_Fisch0240500.1	1.145741686	3.82311	0.299688391	0.764414854	NA
Glycyrrhiza_uralensis_Fisch0237650.1	1.145438444	0.45980023	2.491165443	0.012732482	0.279271618
Glycyrrhiza_uralensis_Fisch0142910.1	1.145242393	0.440568968	2.599462232	0.009336995	0.237026417
Glycyrrhiza_uralensis_Fisch0280660.1	1.144877701	0.440245358	2.600544631	0.009307591	0.237026417
Glycyrrhiza_uralensis_Fisch0107450.1	1.142718473	0.702183764	1.62737809	0.103656822	0.690599974
Glycyrrhiza_uralensis_Fisch0192360.1	1.142659507	1.58551397	0.720687127	0.471102035	0.928837698
Glycyrrhiza_uralensis_Fisch0125290.1	1.142409008	0.697222257	1.63851483	0.101314343	0.683644598
Glycyrrhiza_uralensis_Fisch0033780.1	1.141337819	0.571009685	1.998806412	0.045629304	0.511080553
Glycyrrhiza_uralensis_Fisch0012650.1	1.141089767	0.450172894	2.534781151	0.01125176	0.264482278
Glycyrrhiza_uralensis_Fisch0257680.1	1.140878091	0.746753818	1.527783405	0.126566324	0.72105359
Glycyrrhiza_uralensis_Fisch0259370.1	1.140512378	0.583271134	1.955372573	0.050539111	0.530980532
Glycyrrhiza_uralensis_Fisch0155290.1	1.140494083	0.58654798	1.944417373	0.051845141	0.536044903
Glycyrrhiza_uralensis_Fisch0100150.1	1.140199778	0.499136332	2.284345387	0.02235124	0.365243031
Glycyrrhiza_uralensis_Fisch0122900.1	1.140185938	0.356864858	3.195007608	0.001398272	0.083307911
Glycyrrhiza_uralensis_Fisch0222880.1	1.14014733	0.604886316	1.884895227	0.059443985	0.566660084
Glycyrrhiza_uralensis_Fisch0144850.1	1.139638154	3.08826771	0.369021815	0.712111465	0.974327393
Glycyrrhiza_uralensis_Fisch0136440.1	1.138831184	3.336622603	0.341312555	0.732868298	NA
Glycyrrhiza_uralensis_Fisch0173330.1	1.138815797	2.131266662	0.534337545	0.59310802	0.957183077
Glycyrrhiza_uralensis_Fisch0098080.1	1.138595038	0.726471285	1.567295311	0.117045681	0.706303999
Glycyrrhiza_uralensis_Fisch0075760.1	1.138030081	0.917429474	1.240455112	0.214807107	0.814737191
Glycyrrhiza_uralensis_Fisch0116270.1	1.137465457	0.650113503	1.749641334	0.080180223	0.634047973
Glycyrrhiza_uralensis_Fisch0	1.137376913	1.429489	0.7956525	0.426234	0.916286

259600.1		431	51	013	886
Glycyrrhiza_uralensis_Fisch0061930.1	1.13647874	0.43091285	2.637374912	0.008355043	0.23021017
Glycyrrhiza_uralensis_Fisch0010650.1	1.136166921	0.654425699	1.736128216	0.082541166	0.639269575
Glycyrrhiza_uralensis_Fisch0046440.1	1.135950724	0.746210105	1.522293409	0.127935579	0.72182017
Glycyrrhiza_uralensis_Fisch0215160.1	1.13591538	0.750194873	1.514160415	0.129985159	0.724285196
Glycyrrhiza_uralensis_Fisch0067890.1	1.135359786	0.744811291	1.524358988	0.127419059	0.72182017
Glycyrrhiza_uralensis_Fisch0102770.1	1.135010535	0.626298326	1.812252225	0.069947233	0.600931266
Glycyrrhiza_uralensis_Fisch0040580.1	1.133879933	1.485510743	0.763292988	0.445288683	0.920535473
Glycyrrhiza_uralensis_Fisch0002650.1	1.133162808	0.478896642	2.366194931	0.017971979	0.328213607
Glycyrrhiza_uralensis_Fisch0057730.1	1.132582313	0.896072915	1.263939901	0.206251599	0.81096352
Glycyrrhiza_uralensis_Fisch0037610.1	1.131716332	0.366177656	3.090620946	0.001997384	0.104743012
Glycyrrhiza_uralensis_Fisch0033990.1	1.130911989	2.007933588	0.563221809	0.573283854	0.952518696
Glycyrrhiza_uralensis_Fisch0270540.1	1.130650796	0.446838617	2.530333665	0.01139541	0.266040274
Glycyrrhiza_uralensis_Fisch0254950.1	1.13046827	0.824348929	1.371346805	0.170266872	0.778850891
Glycyrrhiza_uralensis_Fisch0080590.1	1.130112115	0.424850596	2.660022428	0.007813545	0.223629042
Glycyrrhiza_uralensis_Fisch0028460.1	1.128670109	0.74213897	1.520833907	0.128301523	0.72182017
Glycyrrhiza_uralensis_Fisch0020740.1	1.128289389	1.873322858	0.602293077	0.546979068	0.948327603
Glycyrrhiza_uralensis_Fisch0168110.1	1.128091018	0.55936756	2.01672585	0.043724131	0.504245018
Glycyrrhiza_uralensis_Fisch0200540.1	1.126537667	0.635251871	1.773371662	0.076167142	0.622616391
Glycyrrhiza_uralensis_Fisch0150170.1	1.126004136	0.196107725	5.741763293	9.37E-09	7.78E-06
Glycyrrhiza_uralensis_Fisch0158090.1	1.125986527	0.45127232	2.495137584	0.01259083	0.277600804
Glycyrrhiza_uralensis_Fisch0266590.1	1.125619883	0.301518048	3.733175809	0.000189081	0.021139868
Glycyrrhiza_uralensis_Fisch0	1.125600941	0.654427	1.7199783	0.085436	0.643781

018690.1		371	98	368	241
Glycyrrhiza_uralensis_Fisch0 108880.1	1.125105814	0.443014 688	2.5396580 42	0.011096 09	0.262360 4
Glycyrrhiza_uralensis_Fisch0 201110.1	1.124897104	0.917186 906	1.2264644 17	0.220023 958	0.817830 773
Glycyrrhiza_uralensis_Fisch0 097740.1	1.124024909	0.629011 056	1.7869716 25	0.073942 075	0.614142 768
Glycyrrhiza_uralensis_Fisch0 049340.1	1.123682765	0.406779 514	2.7623877 93	0.005738 028	0.189217 067
Glycyrrhiza_uralensis_Fisch0 207900.1	1.122032761	1.660927 543	0.6755458 81	0.499329 012	0.938792 103
Glycyrrhiza_uralensis_Fisch0 146980.1	1.122028396	0.549354 069	2.0424503 24	0.041106 882	0.492750 327
Glycyrrhiza_uralensis_Fisch0 071500.1	1.12170377	0.447438 794	2.5069434 86	0.012178 018	0.273437 402
Glycyrrhiza_uralensis_Fisch0 187610.1	1.121587101	0.491881 383	2.2801983 18	0.022595 929	0.366993 26
Glycyrrhiza_uralensis_Fisch0 247650.1	1.121193934	0.799692 612	1.4020311 27	0.160905 952	0.766035 099
Glycyrrhiza_uralensis_Fisch0 083060.1	1.121061451	0.662257 425	1.6927880 43	0.090495 82	0.656094 692
Glycyrrhiza_uralensis_Fisch0 210870.1	1.12099069	3.821190 017	0.2933616 71	0.769245 719	NA
Glycyrrhiza_uralensis_Fisch0 118020.1	1.120358806	0.422435 56	2.6521413 25	0.007998 306	0.225432 338
Glycyrrhiza_uralensis_Fisch0 098790.1	1.119381706	1.312164 525	0.8530803	0.393614 775	0.909853 802
Glycyrrhiza_uralensis_Fisch0 211530.1	1.119261457	0.551272 46	2.0303235 49	0.042323 662	0.496648 95
Glycyrrhiza_uralensis_Fisch0 085830.1	1.119097886	2.205610 951	0.5073868 02	0.611883 444	0.961645 976
Glycyrrhiza_uralensis_Fisch0 113870.1	1.118937188	0.423587 181	2.6415747 19	0.008252 16	0.228670 17
Glycyrrhiza_uralensis_Fisch0 032200.1	1.118852317	0.295398 036	3.7876091 87	0.000152 104	0.017859 218
Glycyrrhiza_uralensis_Fisch0 021750.1	1.117241114	0.464798 123	2.4037126 19	0.016229 526	0.315198 656
Glycyrrhiza_uralensis_Fisch0 246330.1	1.116382524	0.495407 941	2.2534611 03	0.024230 091	0.380194 447
Glycyrrhiza_uralensis_Fisch0 186570.1	1.116287503	0.362815 422	3.0767366 37	0.002092 801	0.106971 3
Glycyrrhiza_uralensis_Fisch0 072190.1	1.114963526	0.774907 828	1.4388337 36	0.150197 636	0.753963 939
Glycyrrhiza_uralensis_Fisch0	1.114759811	0.590021	1.8893546	0.058844	0.564515

173670.1		477	34	325	005
Glycyrrhiza_uralensis_Fisch0153910.1	1.114040246	0.671051586	1.660140992	0.096886092	0.674129157
Glycyrrhiza_uralensis_Fisch0196070.1	1.113932373	0.674033202	1.652637243	0.098404714	0.678425608
Glycyrrhiza_uralensis_Fisch0219830.1	1.113838456	3.804201104	0.292791686	0.769681383	NA
Glycyrrhiza_uralensis_Fisch0135060.1	1.113767993	2.059998346	0.540664508	0.588738846	0.956418893
Glycyrrhiza_uralensis_Fisch0161040.1	1.112763271	0.917424203	1.212921206	0.225159947	0.822496577
Glycyrrhiza_uralensis_Fisch0150980.1	1.112681012	0.925878964	1.201756445	0.229457905	0.827098198
Glycyrrhiza_uralensis_Fisch0227850.1	1.112243596	1.301509759	0.854579528	0.392783965	0.908822369
Glycyrrhiza_uralensis_Fisch0052760.1	1.111143131	0.38344292	2.897805838	0.003757831	0.149710448
Glycyrrhiza_uralensis_Fisch0238200.1	1.11083955	0.537431842	2.066940331	0.038739777	0.481530651
Glycyrrhiza_uralensis_Fisch0283490.1	1.110224295	0.669292092	1.658803845	0.097155325	0.67528983
Glycyrrhiza_uralensis_Fisch0017120.1	1.109513428	3.821417048	0.290340838	0.7715555	NA
Glycyrrhiza_uralensis_Fisch0263060.1	1.109231754	0.461689396	2.402549776	0.016281219	0.315198656
Glycyrrhiza_uralensis_Fisch0184560.1	1.108981338	0.679527871	1.63198801	0.102682012	0.687864076
Glycyrrhiza_uralensis_Fisch0150760.1	1.108739066	3.01111978	0.368214866	0.712713026	0.974327393
Glycyrrhiza_uralensis_Fisch0201730.1	1.108497481	0.488711135	2.268205903	0.02331666	0.371430842
Glycyrrhiza_uralensis_Fisch0255210.1	1.107397319	0.575709872	1.923533664	0.054413063	0.548459977
Glycyrrhiza_uralensis_Fisch0251920.1	1.106297704	0.561004113	1.971995709	0.048610096	0.525390669
Glycyrrhiza_uralensis_Fisch0158520.1	1.10549347	0.497659473	2.221385366	0.026324871	0.396768725
Glycyrrhiza_uralensis_Fisch0142960.1	1.105359694	0.33002674	3.349303432	0.00081015	0.058308917
Glycyrrhiza_uralensis_Fisch0084750.1	1.105314882	0.956915019	1.155081549	0.248057033	0.843032358
Glycyrrhiza_uralensis_Fisch0094480.1	1.105214018	0.892595404	1.238202676	0.215640919	0.815599769
Glycyrrhiza_uralensis_Fisch0	1.105015443	0.436490	2.5315899	0.011354	0.265565

129380.1		691	42	669	821
Glycyrrhiza_uralensis_Fisch0166930.1	1.104821429	0.687037402	1.608095027	0.107814368	0.692784222
Glycyrrhiza_uralensis_Fisch0097440.1	1.104792171	0.505514332	2.185481405	0.028853568	0.418058016
Glycyrrhiza_uralensis_Fisch0158290.1	1.104076024	1.377599914	0.801448964	0.422871778	0.913935905
Glycyrrhiza_uralensis_Fisch0243760.1	1.10405971	0.726587556	1.519513651	0.128633255	0.72182017
Glycyrrhiza_uralensis_Fisch0194840.1	1.104017622	1.709238529	0.645911968	0.518336368	0.943012745
Glycyrrhiza_uralensis_Fisch0154530.1	1.103436745	2.652930809	0.415931219	0.677460334	NA
Glycyrrhiza_uralensis_Fisch0008890.1	1.102588071	3.736295428	0.295101951	0.767915999	NA
Glycyrrhiza_uralensis_Fisch0206770.1	1.102329506	0.457544344	2.409229884	0.015986225	0.313428369
Glycyrrhiza_uralensis_Fisch0197740.1	1.102194812	1.711100293	0.644143898	0.519482127	0.943621975
Glycyrrhiza_uralensis_Fisch0142130.1	1.102131904	0.448029761	2.459952442	0.013895543	0.289330203
Glycyrrhiza_uralensis_Fisch0229960.1	1.101669966	0.55101689	1.999339739	0.045571607	0.510902928
Glycyrrhiza_uralensis_Fisch0163510.1	1.100686358	0.490553813	2.243762722	0.024847672	0.386551852
Glycyrrhiza_uralensis_Fisch0248230.1	1.100215974	0.819483348	1.342572703	0.179410374	0.789067991
Glycyrrhiza_uralensis_Fisch0051900.1	1.100151663	2.7331631	0.40251958	0.687301681	0.969545301
Glycyrrhiza_uralensis_Fisch0225590.1	1.098954075	1.110571863	0.989538914	0.32239954	0.886479038
Glycyrrhiza_uralensis_Fisch0152790.1	1.098911854	0.638809108	1.72025076	0.08538687	0.643781241
Glycyrrhiza_uralensis_Fisch0207470.1	1.098836169	0.943563534	1.164559809	0.24419722	0.839393558
Glycyrrhiza_uralensis_Fisch0041120.1	1.098690327	0.889568455	1.235082383	0.216799845	0.816034982
Glycyrrhiza_uralensis_Fisch0182850.1	1.098360289	0.43452891	2.5277036	0.011481123	0.266647518
Glycyrrhiza_uralensis_Fisch0186270.1	1.098335838	0.351205423	3.127331658	0.001764008	0.096939897
Glycyrrhiza_uralensis_Fisch0119280.1	1.098320422	0.639952589	1.716252798	0.086115783	0.646325227
Glycyrrhiza_uralensis_Fisch0	1.096997485	3.264409	0.3360478	0.736834	NA

231560.1		026	04	818	
Glycyrrhiza_uralensis_Fisch0 123360.1	1.096454756	0.428865 122	2.5566424 04	0.010568 78	0.255978 083
Glycyrrhiza_uralensis_Fisch0 161030.1	1.095479537	1.006451 83	1.0884569 98	0.276393 411	0.859692 133
Glycyrrhiza_uralensis_Fisch0 096720.1	1.09547501	0.297962 561	3.6765525 45	0.000236 407	0.024317 61
Glycyrrhiza_uralensis_Fisch0 181760.1	1.095369521	2.479614 038	0.4417500 08	0.658670 118	0.966328 48
Glycyrrhiza_uralensis_Fisch0 217550.1	1.095289805	0.578981 873	1.8917514 64	0.058524 103	0.563437 26
Glycyrrhiza_uralensis_Fisch0 166190.1	1.09460807	0.619416 811	1.7671591 25	0.077201 583	0.624199 565
Glycyrrhiza_uralensis_Fisch0 174280.1	1.093885445	0.382107 259	2.8627706 45	0.004199 544	0.158935 585
Glycyrrhiza_uralensis_Fisch0 123010.1	1.092860287	0.340255 398	3.2118822 96	0.001318 684	0.079310 83
Glycyrrhiza_uralensis_Fisch0 077220.1	1.092695528	0.404504 974	2.7013154 3	0.006906 58	0.207981 79
Glycyrrhiza_uralensis_Fisch0 168140.1	1.09267617	0.556111 414	1.9648511 85	0.049431 464	0.528259 591
Glycyrrhiza_uralensis_Fisch0 063570.1	1.091574904	1.587446 423	0.6876294 46	0.491686 16	0.936770 028
Glycyrrhiza_uralensis_Fisch0 155840.1	1.090059544	0.741177 457	1.4707133	0.141368 668	0.741661 163
Glycyrrhiza_uralensis_Fisch0 054470.1	1.08929814	0.716534 037	1.5202322 34	0.128452 619	0.721820 17
Glycyrrhiza_uralensis_Fisch0 277250.1	1.089035143	0.392419 195	2.7751831 66	0.005517 063	0.185328 029
Glycyrrhiza_uralensis_Fisch0 137810.1	1.087698193	1.231161 599	0.8834731 31	0.376980 693	0.904607 774
Glycyrrhiza_uralensis_Fisch0 257000.1	1.087448839	0.785703 211	1.3840453 02	0.166344 584	0.772572 421
Glycyrrhiza_uralensis_Fisch0 131720.1	1.087439195	1.252053 386	0.8685246 23	0.385107 199	0.906957 344
Glycyrrhiza_uralensis_Fisch0 258070.1	1.087103129	0.874141 244	1.2436241 13	0.213637 937	0.814082 192
Glycyrrhiza_uralensis_Fisch0 043720.1	1.086990565	1.577498 38	0.6890597 03	0.490785 697	0.936011 543
Glycyrrhiza_uralensis_Fisch0 168700.1	1.086842441	0.520264 85	2.0890176 22	0.036706 137	0.467282 603
Glycyrrhiza_uralensis_Fisch0 130160.1	1.086664908	0.450258 266	2.4134257 82	0.015803 346	0.312817 878
Glycyrrhiza_uralensis_Fisch0	1.086585741	0.350365	3.1012963	0.001926	0.101703

271860.1		009	98	753	838
Glycyrrhiza_uralensis_Fisch0019230.1	1.086194198	1.251015655	0.868249885	0.385257551	0.906957344
Glycyrrhiza_uralensis_Fisch0174610.1	1.085527309	0.733870959	1.479180088	0.13909219	0.737108986
Glycyrrhiza_uralensis_Fisch0145410.1	1.085152355	0.720333609	1.506458038	0.131949631	0.727923
Glycyrrhiza_uralensis_Fisch0139110.1	1.085017801	0.77904105	1.392760754	0.16369215	0.769936456
Glycyrrhiza_uralensis_Fisch0180880.1	1.084322628	0.366469826	2.958831944	0.003088075	0.135277149
Glycyrrhiza_uralensis_Fisch0271010.1	1.084288803	2.575788538	0.420954122	0.673788583	0.967707506
Glycyrrhiza_uralensis_Fisch0101470.1	1.08360435	0.426159832	2.542718176	0.010999391	0.261717014
Glycyrrhiza_uralensis_Fisch0094690.1	1.082671402	1.540722471	0.702703714	0.482240413	0.932363513
Glycyrrhiza_uralensis_Fisch0148360.1	1.08156775	1.28882144	0.839191308	0.401361964	0.910612334
Glycyrrhiza_uralensis_Fisch0081290.1	1.081323009	1.502949935	0.719467085	0.471853176	0.928837698
Glycyrrhiza_uralensis_Fisch0185230.1	1.080735093	0.619733888	1.74386961	0.081181829	0.63655336
Glycyrrhiza_uralensis_Fisch0186980.1	1.080193154	1.38196341	0.78163658	0.434428183	0.917057463
Glycyrrhiza_uralensis_Fisch0041680.1	1.080159283	0.427524448	2.526543891	0.011519099	0.266647518
Glycyrrhiza_uralensis_Fisch0141100.1	1.080105747	0.552131414	1.956247587	0.050435995	0.53063215
Glycyrrhiza_uralensis_Fisch0281690.1	1.079901255	3.760997964	0.287131571	0.774011579	0.979626597
Glycyrrhiza_uralensis_Fisch0042110.1	1.079658314	0.332969996	3.242509312	0.001184821	0.07446119
Glycyrrhiza_uralensis_Fisch0231140.1	1.078868117	1.212512335	0.889779086	0.373584519	0.904190987
Glycyrrhiza_uralensis_Fisch0115130.1	1.078602777	0.805638342	1.338817581	0.180630066	0.789903344
Glycyrrhiza_uralensis_Fisch0222960.1	1.078570183	0.436054523	2.473475508	0.013380598	0.286031068
Glycyrrhiza_uralensis_Fisch0061500.1	1.07850922	0.576461085	1.870914184	0.061356977	0.572797664
Glycyrrhiza_uralensis_Fisch0027150.1	1.077830226	0.449124385	2.399847929	0.016401884	0.315330154
Glycyrrhiza_uralensis_Fisch0	1.0777791	0.349681	3.0821723	0.002054	0.105784

128320.1		649	26	958	917
Glycyrrhiza_uralensis_Fisch0 067750.1	1.077428	1.755821 662	0.6136317 96	0.539458 668	0.947110 698
Glycyrrhiza_uralensis_Fisch0 160990.1	1.076620459	2.039576 672	0.5278646 66	0.597593 27	0.957988 27
Glycyrrhiza_uralensis_Fisch0 045070.1	1.076554183	2.154336 054	0.4997150 66	0.617275 723	0.962288 147
Glycyrrhiza_uralensis_Fisch0 091230.1	1.076499011	0.474421 509	2.2690771 64	0.023263 634	0.371323 386
Glycyrrhiza_uralensis_Fisch0 020750.1	1.076391365	1.169423 684	0.9204460 11	0.357339 734	0.897807 124
Glycyrrhiza_uralensis_Fisch0 171900.1	1.076350673	0.865944 219	1.2429792 23	0.213875 49	0.814082 192
Glycyrrhiza_uralensis_Fisch0 096200.1	1.075586156	0.407871 517	2.6370710 19	0.008362 532	0.230210 17
Glycyrrhiza_uralensis_Fisch0 127160.1	1.07554799	1.459717 99	0.7368190 28	0.461232 41	0.924721 482
Glycyrrhiza_uralensis_Fisch0 231800.1	1.075278588	0.821987 518	1.3081446 67	0.190824 246	0.798375 977
Glycyrrhiza_uralensis_Fisch0 017950.1	1.075090565	2.507191 744	0.4288026 9	0.668066 821	0.966501 962
Glycyrrhiza_uralensis_Fisch0 086860.1	1.074708348	1.190798 667	0.9025105 4	0.366785 725	0.900974 301
Glycyrrhiza_uralensis_Fisch0 200930.1	1.071357304	1.163384 67	0.9208968 73	0.357104 272	0.897807 124
Glycyrrhiza_uralensis_Fisch0 127750.1	1.071270046	2.322809 499	0.4611958 26	0.644658 116	0.965587 841
Glycyrrhiza_uralensis_Fisch0 046180.1	1.071093685	0.644733 255	1.6612974 07	0.096653 731	0.673652 444
Glycyrrhiza_uralensis_Fisch0 155900.1	1.070863818	0.583740 004	1.8344876 33	0.066581 642	0.591154 599
Glycyrrhiza_uralensis_Fisch0 088120.1	1.07074239	0.903271 722	1.1854045 28	0.235857 542	0.832447 744
Glycyrrhiza_uralensis_Fisch0 097680.1	1.06973444	0.360997 347	2.9632750 77	0.003043 844	0.135175 887
Glycyrrhiza_uralensis_Fisch0 114810.1	1.069199616	1.056644 193	1.0118823 57	0.311594 306	0.880102 051
Glycyrrhiza_uralensis_Fisch0 199810.1	1.068130861	1.043287 771	1.0238123 09	0.305923 935	0.876258 338
Glycyrrhiza_uralensis_Fisch0 056560.1	1.067754411	2.493379 046	0.4282358 96	0.668479 384	0.966501 962
Glycyrrhiza_uralensis_Fisch0 051260.1	1.067503513	0.392383 106	2.7205644 12	0.006517 057	0.201367 871
Glycyrrhiza_uralensis_Fisch0	1.066563457	0.705152	1.5125283	0.130399	0.725270

067440.1		71	39	509	532
Glycyrrhiza_uralensis_Fisch0266340.1	1.065926197	0.465818671	2.288285688	0.022120889	0.364287823
Glycyrrhiza_uralensis_Fisch0081070.1	1.065164153	1.490485758	0.714642289	0.474830107	0.92913334
Glycyrrhiza_uralensis_Fisch0022070.1	1.064348481	0.591165098	1.8004251	0.071793541	0.605702951
Glycyrrhiza_uralensis_Fisch0226020.1	1.064119928	0.6357115	1.673903851	0.094149497	0.665640916
Glycyrrhiza_uralensis_Fisch0237850.1	1.063797265	3.854314784	0.276001656	0.782546794	NA
Glycyrrhiza_uralensis_Fisch0039010.1	1.063626913	0.494459094	2.15109182	0.031468953	0.434820723
Glycyrrhiza_uralensis_Fisch0259100.1	1.063570595	0.408390749	2.604296492	0.009206308	0.236747677
Glycyrrhiza_uralensis_Fisch0165790.1	1.063469168	1.503438197	0.707358088	0.479343976	0.931830837
Glycyrrhiza_uralensis_Fisch0031890.1	1.063257468	0.330026495	3.221733659	0.001274175	0.077644043
Glycyrrhiza_uralensis_Fisch0029850.1	1.062324256	0.875788226	1.212992165	0.225132816	0.822496577
Glycyrrhiza_uralensis_Fisch0270460.1	1.061711831	3.235263958	0.328168534	0.742784238	NA
Glycyrrhiza_uralensis_Fisch0169420.1	1.060928663	0.365419059	2.903320549	0.003692285	0.148900684
Glycyrrhiza_uralensis_Fisch0133590.1	1.060765616	2.028858241	0.522838705	NA	NA
Glycyrrhiza_uralensis_Fisch0038500.1	1.060511343	2.155888368	0.491913848	0.622780248	0.962948566
Glycyrrhiza_uralensis_Fisch0166940.1	1.059293921	0.720709658	1.469792875	0.141617859	0.74193126
Glycyrrhiza_uralensis_Fisch0039150.1	1.059152845	1.418691565	0.7465702	0.455323042	0.923849416
Glycyrrhiza_uralensis_Fisch0050150.1	1.058105802	0.694810363	1.522869919	0.127791253	0.72182017
Glycyrrhiza_uralensis_Fisch0195550.1	1.057475421	0.458574903	2.306003694	0.021110426	0.355582892
Glycyrrhiza_uralensis_Fisch0206860.1	1.057141947	0.521956267	2.025345826	0.042831866	0.499497585
Glycyrrhiza_uralensis_Fisch0174890.1	1.057023869	0.750450439	1.408519223	0.158977379	0.763028018
Glycyrrhiza_uralensis_Fisch0140520.1	1.05698107	0.564929121	1.87099767	0.061345404	0.572797664
Glycyrrhiza_uralensis_Fisch0	1.055783877	0.290942	3.6288371	0.000284	0.028317

002770.1		755	47	701	138
Glycyrrhiza_uralensis_Fisch0092640.1	1.054818245	0.548148614	1.924328947	0.054313364	0.548375133
Glycyrrhiza_uralensis_Fisch0088350.1	1.054490992	0.557985827	1.889816806	0.058782465	0.564515005
Glycyrrhiza_uralensis_Fisch0124050.1	1.054108389	0.760522247	1.386032286	0.165737045	0.771695423
Glycyrrhiza_uralensis_Fisch0083550.1	1.053955031	2.271651325	0.46395986	0.642676504	0.965035372
Glycyrrhiza_uralensis_Fisch0162510.1	1.053516085	0.654360157	1.609994243	0.107399113	0.692127618
Glycyrrhiza_uralensis_Fisch0159730.1	1.053104077	0.402932659	2.613598216	0.008959433	0.235243816
Glycyrrhiza_uralensis_Fisch0003110.1	1.052880013	2.18217917	0.482490177	0.629457775	0.963563011
Glycyrrhiza_uralensis_Fisch0086120.1	1.051267542	0.869359093	1.209244317	0.226568996	0.823982965
Glycyrrhiza_uralensis_Fisch0061890.1	1.05123333	0.966648222	1.087503505	0.276814351	0.859692133
Glycyrrhiza_uralensis_Fisch0095490.1	1.050683575	1.204711287	0.872145539	0.383128985	0.906519843
Glycyrrhiza_uralensis_Fisch0071540.1	1.050145376	0.241703511	4.344766739	1.39E-05	0.002820109
Glycyrrhiza_uralensis_Fisch0061400.1	1.04921612	0.609984386	1.720070456	0.085419635	0.643781241
Glycyrrhiza_uralensis_Fisch0021220.1	1.049136909	0.360577038	2.909605433	0.003618853	0.147887593
Glycyrrhiza_uralensis_Fisch0215860.1	1.048938361	0.350416166	2.993407448	0.002758812	0.126004929
Glycyrrhiza_uralensis_Fisch0213500.1	1.048484057	0.545410973	1.922374336	0.054558672	0.548778623
Glycyrrhiza_uralensis_Fisch0109420.1	1.048278944	1.14748481	0.913544942	0.360956008	0.898417073
Glycyrrhiza_uralensis_Fisch0233760.1	1.048133991	1.460406146	0.717700343	0.472942072	0.928837698
Glycyrrhiza_uralensis_Fisch0263350.1	1.047672379	0.71062983	1.474287082	0.140404312	0.740482679
Glycyrrhiza_uralensis_Fisch0146970.1	1.047274695	0.514014584	2.037441599	0.041605809	0.49475144
Glycyrrhiza_uralensis_Fisch0246870.1	1.047062103	3.614775124	0.289661754	0.772075018	NA
Glycyrrhiza_uralensis_Fisch0247990.1	1.046687281	1.270817858	0.823632808	0.410148228	0.911645974
Glycyrrhiza_uralensis_Fisch0	1.046172923	0.339233	3.0839276	0.002042	0.105784

209570.1		939	46	873	917
Glycyrrhiza_uralensis_Fisch0056210.1	1.045612888	0.547185078	1.910894375	0.056018156	0.553093989
Glycyrrhiza_uralensis_Fisch0157540.1	1.045101365	1.677171752	0.623133179	0.533197008	0.94591302
Glycyrrhiza_uralensis_Fisch0152500.1	1.043890455	2.551534905	0.409122545	0.682449732	0.969035029
Glycyrrhiza_uralensis_Fisch0106690.1	1.043391158	0.468525013	2.226970024	0.025949277	0.393837691
Glycyrrhiza_uralensis_Fisch0033730.1	1.043389913	0.686075432	1.520809323	0.128307694	0.72182017
Glycyrrhiza_uralensis_Fisch0130600.1	1.041130769	0.323764951	3.215699431	0.00130127	0.078697436
Glycyrrhiza_uralensis_Fisch0024350.1	1.040873053	1.295300045	0.803576791	0.421641432	0.913755795
Glycyrrhiza_uralensis_Fisch0218130.1	1.040113878	0.81196807	1.280978792	0.200201115	0.806142846
Glycyrrhiza_uralensis_Fisch0109290.1	1.038848922	0.398372656	2.607731492	0.009114442	0.236747677
Glycyrrhiza_uralensis_Fisch0228650.1	1.03773418	1.549646427	0.669658679	0.503075398	0.93941259
Glycyrrhiza_uralensis_Fisch0037640.1	1.037623543	2.99466604	0.34649057	0.72897407	0.976482241
Glycyrrhiza_uralensis_Fisch0017140.1	1.037290734	0.645559721	1.606808326	0.108096421	0.69307452
Glycyrrhiza_uralensis_Fisch0165300.1	1.037228092	1.239026259	0.837131646	0.402518574	0.910612334
Glycyrrhiza_uralensis_Fisch0218120.1	1.036710725	1.360668266	0.761912915	0.446111981	0.920840013
Glycyrrhiza_uralensis_Fisch0273010.1	1.036527432	0.357746858	2.897376757	0.003762976	0.149710448
Glycyrrhiza_uralensis_Fisch0250950.1	1.03642531	0.632309011	1.639112036	0.101189929	0.683209422
Glycyrrhiza_uralensis_Fisch0177610.1	1.035399168	1.345016243	0.769804211	0.441416041	0.919801681
Glycyrrhiza_uralensis_Fisch0066720.1	1.033981956	0.567778958	1.821099463	0.068591739	0.597572186
Glycyrrhiza_uralensis_Fisch0107690.1	1.033342839	0.287760743	3.590979191	0.000329438	0.031218784
Glycyrrhiza_uralensis_Fisch0101960.1	1.032992857	0.331958209	3.11181597	0.001859404	0.099678948
Glycyrrhiza_uralensis_Fisch0101140.1	1.032500443	0.355577217	2.90373059	0.003687453	0.148900684
Glycyrrhiza_uralensis_Fisch0	1.032300838	1.611946	0.6404062	0.521908	0.944252

210560.1		914	25	537	825
Glycyrrhiza_uralensis_Fisch0126660.1	1.030937753	1.417613728	0.7272346	0.467082254	0.927306982
Glycyrrhiza_uralensis_Fisch0198590.1	1.030800995	0.316265955	3.259285353	0.001116933	0.072076591
Glycyrrhiza_uralensis_Fisch0234310.1	1.030573378	1.392597405	0.740036836	0.459277643	0.924102621
Glycyrrhiza_uralensis_Fisch0056900.1	1.029631946	2.362039492	0.435908015	0.66290347	0.96632848
Glycyrrhiza_uralensis_Fisch0054250.1	1.02954185	1.674571968	0.61480896	0.538680891	0.947110698
Glycyrrhiza_uralensis_Fisch0270200.1	1.029272892	1.18713143	0.867025223	0.38592819	0.907171672
Glycyrrhiza_uralensis_Fisch0170010.1	1.028735517	0.484396856	2.123745239	0.033691452	0.449714768
Glycyrrhiza_uralensis_Fisch0072770.1	1.027938305	1.113044258	0.923537674	0.355727077	0.897807124
Glycyrrhiza_uralensis_Fisch0070980.1	1.027366608	0.240907397	4.264570618	2.00E-05	0.003680082
Glycyrrhiza_uralensis_Fisch0015250.1	1.026409101	0.448568817	2.288186479	0.022126664	0.364287823
Glycyrrhiza_uralensis_Fisch0173600.1	1.025116072	0.383276306	2.674613736	0.007481539	0.217488712
Glycyrrhiza_uralensis_Fisch0137200.1	1.024929299	0.438615472	2.336737678	0.019452828	0.341686247
Glycyrrhiza_uralensis_Fisch0006300.1	1.023546167	0.537447762	1.904457027	0.056850688	0.556711174
Glycyrrhiza_uralensis_Fisch0067780.1	1.022322673	0.233819447	4.372273927	1.23E-05	0.002573588
Glycyrrhiza_uralensis_Fisch0114850.1	1.021749128	0.505661503	2.020618779	0.043319243	0.502010217
Glycyrrhiza_uralensis_Fisch0204020.1	1.021292531	1.544769987	0.66112919	0.508529467	0.940226457
Glycyrrhiza_uralensis_Fisch0008250.1	1.021053164	0.559583961	1.824664813	0.068051626	0.595050085
Glycyrrhiza_uralensis_Fisch0007250.1	1.020179634	3.790733976	0.269124565	0.787833827	0.981741111
Glycyrrhiza_uralensis_Fisch0279110.1	1.018973964	1.071258497	0.951193355	0.341506231	0.894105199
Glycyrrhiza_uralensis_Fisch0166010.1	1.018115105	0.325672816	3.126190013	0.001770871	0.097018442
Glycyrrhiza_uralensis_Fisch0030950.1	1.017379378	3.499622533	0.290711175	0.771272224	NA
Glycyrrhiza_uralensis_Fisch0	1.015838961	0.479654	2.1178556	0.034187	0.451552

043590.1		487	39	296	119
Glycyrrhiza_uralensis_Fisch0234730.1	1.01582117	0.285788917	3.55444564	0.000378777	0.034275029
Glycyrrhiza_uralensis_Fisch0077030.1	1.013439887	0.912137251	1.111060737	0.266542207	0.854047617
Glycyrrhiza_uralensis_Fisch0256660.1	1.013102483	0.520930894	1.944792477	0.05179996	0.535990973
Glycyrrhiza_uralensis_Fisch0244600.1	1.012954189	1.587045448	0.638264134	0.523301755	0.944784972
Glycyrrhiza_uralensis_Fisch0073940.1	1.012844	0.41095332	2.464620554	0.013715841	0.28815719
Glycyrrhiza_uralensis_Fisch0114290.1	1.011893195	1.317636331	0.76796091	0.442510409	0.9200858
Glycyrrhiza_uralensis_Fisch0272970.1	1.011293731	0.49127793	2.058496156	0.03954253	0.483141469
Glycyrrhiza_uralensis_Fisch0114050.1	1.011145394	1.442606913	0.700915395	0.483355816	0.932636918
Glycyrrhiza_uralensis_Fisch0106560.1	1.011118176	0.62052169	1.629464678	0.103214687	0.68933893
Glycyrrhiza_uralensis_Fisch0266520.1	1.011103362	0.462819279	2.184661289	0.028913691	0.418240704
Glycyrrhiza_uralensis_Fisch0079620.1	1.010900251	0.421793835	2.396669099	0.016544857	0.316813618
Glycyrrhiza_uralensis_Fisch0137580.1	1.009727162	0.229139718	4.40660036	1.05E-05	0.002256684
Glycyrrhiza_uralensis_Fisch0126980.1	1.009076792	0.600545092	1.680268153	0.092905154	0.662056741
Glycyrrhiza_uralensis_Fisch0010520.1	1.008082647	0.473830279	2.127518421	0.033377032	0.447319137
Glycyrrhiza_uralensis_Fisch0144170.1	1.007860341	0.490963311	2.05282211	0.040089833	0.486678992
Glycyrrhiza_uralensis_Fisch0149800.1	1.007694868	0.803430002	1.254241023	0.209754413	0.811966832
Glycyrrhiza_uralensis_Fisch0283480.1	1.007240965	0.648373286	1.553489303	0.120306283	0.711449612
Glycyrrhiza_uralensis_Fisch0142800.1	1.007136591	1.651741007	0.60974244	0.542032437	0.94732072
Glycyrrhiza_uralensis_Fisch0233370.1	1.006432664	2.597781049	0.387420127	0.698445206	0.972014924
Glycyrrhiza_uralensis_Fisch0142120.1	1.005813003	0.551245804	1.824617975	0.068058699	0.595050085
Glycyrrhiza_uralensis_Fisch0095750.1	1.003376397	1.916226398	0.523621007	0.600542167	NA
Glycyrrhiza_uralensis_Fisch0	1.002968842	2.098921	0.4778497	0.632757	0.963563

044190.1		006	33	161	011
Glycyrrhiza_uralensis_Fisch0198050.1	1.002961319	0.519905002	1.929124197	0.053715446	0.546162726
Glycyrrhiza_uralensis_Fisch0011430.1	1.002339513	0.473307381	2.117734803	0.034197534	0.451552119
Glycyrrhiza_uralensis_Fisch0142670.1	1.002197725	0.631355855	1.587373771	0.112428016	0.699985087
Glycyrrhiza_uralensis_Fisch0082840.1	1.001359467	1.946259232	0.514504672	0.606899195	NA
Glycyrrhiza_uralensis_Fisch0184380.1	1.001321105	3.726262619	0.26871995	NA	NA
Glycyrrhiza_uralensis_Fisch0045680.1	1.001184713	1.895962792	0.528061372	0.59745674	0.95798827
Glycyrrhiza_uralensis_Fisch0102870.1	1.000445302	0.533558333	1.875043909	0.060786683	0.571804506
